# Identification of starch candidate genes using SLAF-seq and BSA strategies and development of related SNP-CAPS markers in tetraploid potato

**DOI:** 10.1371/journal.pone.0261403

**Published:** 2021-12-21

**Authors:** Jiaqi Li, Xiaoxia Yu, Sheng Zhang, Zhuo Yu, Jingwei Li, Xinghong Jin, Xia Zhang, Dongsheng Yang

**Affiliations:** Agricultural College, Inner Mongolia Agricultural University, Hohhot, Inner Mongolia, China; USDA-ARS Southern Regional Research Center, UNITED STATES

## Abstract

Potato starch is an essential nutrient for humans and is widely used worldwide. Locating relevant genomic regions, mining stable genes and developing candidate gene markers can promote the breeding of new high-starch potato varieties. A total of 106 F_1_ individuals and their parents (YSP-4 × MIN-021) were used as test materials, from which 20 plants with high starch content and 20 with low starch content were selected to construct DNA pools for site-specific amplified fragment sequencing (SLAF-seq) and bulked segregation analysis (BSA). A genomic region related to the starch traits was first identified in the 0–5.62 Mb of chromosome 2 in tetraploid potato. In this section, a total of 41 non-synonymous genes, which were considered as candidate genes related to the starch trait, were annotated through a basic local alignment search tool (BLAST) search of multiple databases. Six candidate genes for starch (PGSC0003DMG400017793, PGSC0003DMG400035245, PGSC0003DMG400036713, PGSC0003DMG400040452, PGSC0003DMG400006636 and PGSC0003DMG400044547) were further explored. In addition, cleaved amplified polymorphic sequence (CAPS) markers were developed based on single nucleotide polymorphism (SNP) sites associated with the starch candidate genes. SNP-CAPS markers chr2-CAPS6 and chr2-CAPS21 were successfully developed and validated with the F_2_ population and 24 tetraploid potato varieties (lines). Functional analysis and cloning of the candidate genes associated with potato starch will be performed in further research, and the SNP-CAPS markers chr2-CAPS6 and chr2-CAPS21 can be further used in marker-assisted selection breeding of tetraploid potato varieties with high starch content.

## Introduction

Potato (*Solanum tuberosum* L.) belongs to the solanaceae solanum family and is the fourth major food crop in the world after rice, wheat and maize. It can also be used as a vegetable and feed crop. Potato tubers are rich in starch, protein, amino acids and minerals that can maintain human life, and are widely planted in China, America, Netherlands and other countries [1, 2]. The cultivated potato is autotetraploid (2*n* = 4*x* = 48) with high heterozygosity and complex genetic characteristics, which leads to slow progress in potato breeding [3]. Breeding potato varieties with high quality, high yield, and strong disease/pest resistance is always a primary mission and challenge for potato breeders.

Potato starch, found as a carbohydrate in potato chips, is the second most produced type of starch in the world [4]. Its quality characteristics of large grain size, high gelatinization transparency, easy expansion and high viscosity are far superior to those of other types of starch, and it is widely used in food processing, agriculture, petroleum, medicine and other industries [5–8]. As one of the most important quality traits of potato starch, the quantitative trait is controlled by multiple genes. Understanding the genetic determinants of starch content is of great significance for quality improvement in potato breeding. Quantitative trait locus (QTL) mapping of potato starch has been reported. Freyre and Douches (1994) [9] used a diploid F_1_ population and their parents 84SD22 and 84510 as experimental materials to construct linkage maps using 44 restriction fragment length polymorphism (RFLP) markers and 63 random amplified polymorphic DNA (RAPD) markers. QTLs related to starch content in tubers were mapped on chromosomes 1, 2, 3, 5, 7 and 11, respectively. Li et al. (2008) [10] mapped QTLs for the starch trait on potato chromosomes 2, 3, 5, 7 and 11 by using single-stranded conformation polymorphism (SSCP), cleaved amplified polymorphic sequence (CAPS), sequence characterized amplified region (SCAR) and simple-sequence repeats (SSR) markers and candidate gene association analysis. Bradshaw et al. (2008) [11] selected 227 tetraploid potato individuals generated by Stirling (low dry matter content) × 1260 ab1 (high dry matter content) as the mapping population. Stable QTLs that control the dry matter content (closely related to starch content) were detected on linkage group 5 of Stirling based on amplified fragment length polymorphism (AFLP) and SSR. Werij et al. (2012) [12] used 16 pairs of AFLP markers and 26 pairs of SSR markers to construct a genetic linkage map, and successfully mapped the starch content, starch grain size, starch gelation temperature, amylose content and other trait-related QTLs on all chromosomes except chromosome 8 of potato. Sliwka et al. (2016) [13] mapped 30 QTLs related to the starch traits on potato chromosomes 1, 2, 3, 8, 10, 11 and 12. In general, although some QTLs that control potato starch have been identified in previous studies, the data on all of them are preliminary. Due to the lack of saturated linkage maps in potato, the starch content trait had also not yet been finely mapped in this species. The genetic mechanism for this important trait in potato is still not very clear and no genes involved in regulating the starch content of potato has been finely mapped or cloned.

Bulked segregation analysis (BSA) is a widely used gene marker localization strategy that can identify molecular markers closely linked to target trait genes [14]. The advantage of this method is that there is no need to genotype all individuals in the population, and individuals with extreme traits can be selected for mixed analysis, which greatly reduces the workload and cost of research. BSA can also be used in the research of crops, for which constructing near-isogenic lines is difficult. Site-specific amplified fragment sequencing (SLAF-seq) is a rapid and efficient large-scale genotyping technique that combines reduced-representation with high-throughput sequencing [15]. Compared with other genotyping techniques, such as genotyping-by-sequencing (GBS), restriction-site associated DNA sequencing (RAD-seq) and multiple shotgun genotyping (MSG), SLAF-seq has the advantages of high resolution, high accuracy, low cost and applicability to a large number of species. In recent years, with the rapid development of next-generation sequencing, SLAF-seq combined with BSA has been successfully applied for marker development, QTL mapping and candidate gene association analysis of a large number of species, such as rice [16], pepper [17, 18], cotton [19], melon [20], watermelon [21] and potato [22, 23] etc. and has been demonstrated as an effective strategy to identify genes or QTLs linked to important traits in different plants.

In the present study, SLAF-seq combined with BSA was first used to develop SNP loci and identify genome regions and candidate genes related to the starch trait by pooling DNAs in an F_1_ population derived from a cross between YSP-4 and MIN-021 in tetraploid potato. In addition, the SNP-CAPS markers tightly linked to the potato starch trait were subsequently developed based on the genome regions and candidate genes. These results lay a foundation for molecular assisted breeding of high starch potato varieties and functional analysis and cloning of related genes.

## Materials and methods

### Plant materials

The F_1_ and F_2_ isolated populations, their parents and 24 potato varieties (lines) were used for marker development and validation. The F_1_ population was composed of 106 individuals from a cross of YSP-4 × MIN-021, and the F_2_ segregation population comprised of 120 plants from F_1_ population self-crossing. The female parent YSP-4 is a wild species, with high starch content (about 19%), strong disease resistance, high tuber setting rate per plant and wide adaptability. The male parent, MIN-021, is a tetraploid-colored potato material with short growth period, red tuber, nearly round-shape tuber and low starch content (about 13%). All materials in this experiment were planted in the experimental farm at Inner Mongolia Agricultural University in China. The plant spacing was 30 cm and the row spacing was 90 cm.

### Determination of the starch content and genetic analysis in potato tubers

The starch content of potato material was determined by iodine-potassium iodide colorimetry [24]. The phenotypic data were statistically analyzed using SPSS 10.0 (SPSS Inc., Chicago, IL).

### DNA extraction and construction of DNA pools

The young leaves of the parents, F_1_ population, F_2_ segregation population and 24 tetraploid varieties (strains) were randomly taken as samples. DNA was extracted according to the instructions for the plant Genome Kit (Tiangen Biochemical Technology, Beijing, China). DNA was quantified with a Nanodrop 2000 UV-vis spectrophotometer (NanoDrop, Wilmington, DE, USA) and diluted to 100 ng/μL. Equal amounts of DNA from each of 20 extremely high starch individuals (H-pool) and 20 extremely low starch individuals (L-pool) identified in the F_1_ population were separately pooling to construct two extreme DNA pools. The genomic DNAs of the two DNA bulks and both parents were prepared for SLAF sequencing and BSA analysis.

### SLAF library preparation and high-throughput sequencing

The sequence of the double-haploid line DM genome was selected as the reference genome (http://solanaceae.plantbiology.msu.edu/pgsc_download.shtml), and genomic DNAs from both parents and the H-pool and L-pool were digested with the Rsa I and Hae III restriction enzymes. Afterward, specific single nucleotide A overhangs were added to the end of the resulting enzyme digestion fragment and connected to dual-index sequencing adaptors. The modified enzyme digestion fragment was amplified with polymerase chain reaction (PCR), purified and mixed by gel electrophoresis. The target fragment was selected by gluing to construct the SLAF library. To evaluate the accuracy and reliability of the SLAF library construction and sequencing, the same experimental operation was performed with *Oryza sativa japonica* as the control.

After passing the library quality test, qualified SLAF tags were sequenced on the IlluminaHiSeq^TM^ 2500 platform (Illumina, Inc., San Diego, USA) by Biomarker Company (Beijing, China). The original data files obtained by sequencing were converted into raw reads through Base Calling. Clean reads were further filtered from raw reads with an adapter, resulting in more than 10% of N content and more than 50% of bases with mass values less than 10. Finally, the GC content and Q30 score of the reads were analyzed to assess the sequencing quality.

### High-quality SNP detection

Burrows-wheeler Aligner (BWA) software [25] was used to align the reads of sequenced samples to the reference genome, and the same reads for different samples were clustered to obtain SLAF tags (sequence similarity > 90%). SNPs were mainly detected with GATK [26] and SAMtools [27] software. According to the localization of the reads on the reference genome, local realignment was carried out with the above two programs, respectively, and the intersection mutation loci were obtained as the final SNP set. Afterward, the annotation variation software SnpEff [28] was used to annotate the regions where the SNPs were located and the influence of the variation.

### Association analysis

The euclidean distance (ED) algorithm assumes that the genetic background, except for the loci, related to the target trait tends to be consistent. The algorithm then seeks SNP markers that are significantly different between the H-pool and L-pool and closely related to the target trait and determines the candidate regions according to the association threshold [29]. The formula for the ED algorithm is as follows:

$$ED=\sqrt{{\left(Amut-Awt\right)}^{2}+{\left(Cmut-Cwt\right)}^{2}+{\left(Gmut-Gwt\right)}^{2}+{\left(Tmut-Twt\right)}^{2}}$$ (1)

Amut, Cmut, Gmut and Tmut are the frequencies of the A, C, G and T bases in the L-pool respectively, while Awt, Cwt, Gwt, and Twt are the frequencies of the A, C, G, and T bases in the H-pool. Larger ED values indicate greater discrepancy in the markers between the two mixed pools. In the association analysis, ED values were multiplied to eliminate background noise, and SNPNUM was used to fit the ED value to determine the association threshold. Regions exceeding the threshold were selected as candidate gene regions for target traits [29].

SNP-index is a valuable algorithm for association analysis and performs marker association analysis by looking for genotype differences between DNA pools [30]. The diversity in genotype between mixed pools is visualized with Δ(SNP-index). Theoretically, when Δ(SNP-index) approaches 1, indicating that an allele originated from the H-pool, the associated SNP locus may be closely linked to high starch traits. On the contrary, Δ(SNP-index) approaching -1 indicates that an allele is derived from the L-pool, and that the associated SNP may be closely linked to low starch traits. The Δ(SNP-index) values were fitted to the SNPNUM method to calculate the correlation threshold, and those above the threshold were selected as the region associated with the target trait [30].

For experimental accuracy, ED and SNP-index were used to obtain the candidate interval, and their intersection was used to determine the final starch candidate interval.

### Prediction of the candidate genes

The basic local alignment search tool (BLAST) [31] was used to deeply annotate the genes in the target regions in multiple databases (non-redundant protein database (NR) [32], Swiss-Prot [33], gene ontology (GO) [34], Kyoto encyclopedia of genes and genomes (KEGG) [35], clusters of orthologous genes (COG) [36]), and the reference gene annotation could be quickly used to screen the candidate genes and predict their related functions.

### Development and validation of candidate gene SNP-CAPS markers

According to the sequencing results for the parents, SNPs linked to starch candidate genes were converted to PCR-based CAPS markers. The process was as follows: First, candidate gene sequences were obtained from the potato genome website (http://solanaceae.plantbiology.msu.edu/). Then, BioXM 2.6 was used to detect the effective restriction sites, and primers were designed 500 bp above and below the restriction sites. The design principles were in accordance with the report by Zhang et al. (2004) [37]. Finally, the primers were commissioned to be synthesized by Thermo Fisher Scientific (Nanjing, China).

PCR amplification and enzyme digestion were carried out using the DNAs of the parents and F_1_ mixed pool as templates. If the CAPS markers obtained by enzyme digestion were specific to the parents and the same bands were found in the high starch pool with YSP-4 (high starch) as well as in the low starch pool with MIN-021 (low starch), it was preliminarily concluded that CAPS was linked to potato starch traits. Extremely high and low-starch individuals from the F_2_ population and 24 varieties (lines) were further verified.

PCR reaction system (20 μL): 2.0 μL 10× buffer (Mg^2+^), 2.0 μL dNTPs, 0.4 μL Taq DNA polymerase, positive and reverse primers (10 μM) 1.0 μL each, 2.0 μL DNA (50 ng/μL), 11.6 μL ddH_2_O. PCR cycle amplification procedure: pre-denaturation at 94°C for 5 min; followed by 35 cycles at 94°C for 30 s, Tm 30 s, 72°C for 60 s; 72°C for 7 min; stopped at 4°C and stored. The restriction enzyme from NEB company was used for enzyme digestion of the PCR products. The reaction system was 0.3 μL restriction enzyme, 1 μL Tango buffer, 5 μL PCR product, and 10 μL ddH_2_O supplement. The digestion system was placed in a thermostatic water bath for 3 h to ensure that the reaction was complete, and the products were evaluated with 2% agarose gel electrophoresis.

## Results and analysis

### Genetic analysis of starch traits

**Fig 1:**
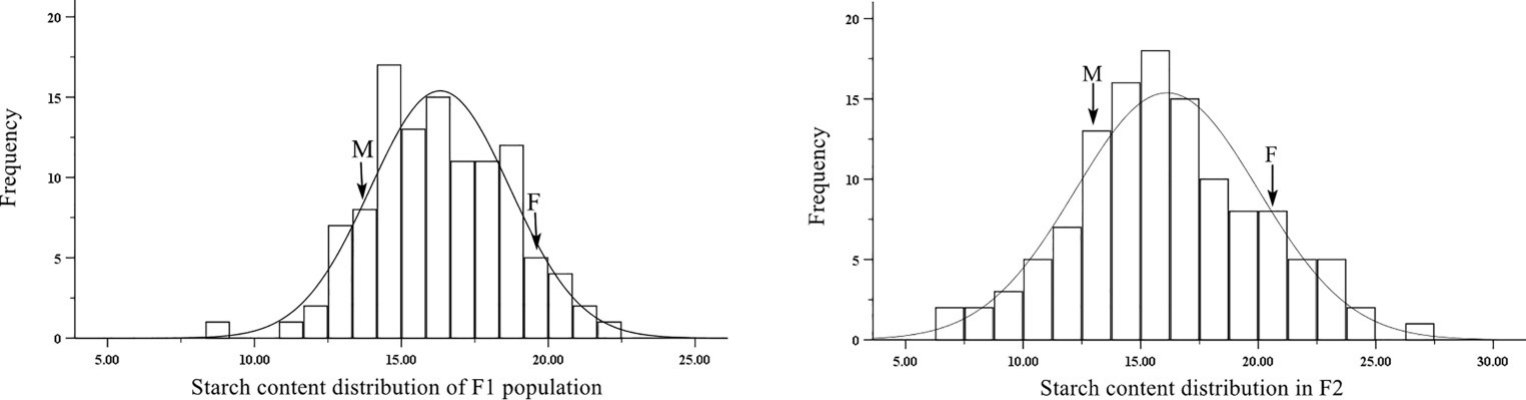
Phenotypic distributions of starch content traits in F_1_ and F_2_ potato populations.

The potato starch data measured were analyzed. As shown in Fig 1, the female starch content (YSP-4) was significantly higher than the male starch content (MIN-021). Some F_1_ individuals showed transgressive segregation, indicating that there was abundant heritable variation in the starch trait in F_1_, which was beneficial to the selection of extreme phenotypes and ensured the efficiency of the BSA strategy. In the F_2_ population, 48.33% of individual starch content was between the parents and 51.67% of individual starch content showed the transgressive phenomena, indicating that the genetic variation of starch traits in F_2_ was high. The starch contents of F_1_ and F_2_ populations had a normal distribution, indicating that the starch content trait are quantitative traits controlled by multiple genes [38, 39].

### Sequencing data analysis and SNP identification

High-throughput sequencing was performed after the SLAF library test, and a total of 2269.98 million reads were obtained. The Q30 was 95.05%, and the average GC content was 35.51%, indicating that the probability of base error identification in Base Calling was small and the quality of the sequencing base was high. On the whole, 838,604 SLAFs were identified by cluster analysis, of which 587,372 SLAFs were found in the male (MIN-021) and 612,629 in the female (YSP-4). The mean sequencing depths were 28.99× and 23.29×, respectively. There were 488,700 SLAFs in the progenitors, and the average sequencing depth was 15.36×. The detailed sequencing data can be obtained from our previous uploaded NCBI-short read archive (SRA) database (accession: PRJNA597429).

GATK and SAMtools software were used to identify 6,301,408 SNPs with intersections. In order to improve the accuracy of the experiment, firstly 135,107 SNPs with multiple alleles were filtered out; secondly, 464,306 SNPs with sequencing depths less than 4× were filtered out. Further, 70,487 SNPs with the same genotype among mixed pools were filtered out, and 4,654,022 SNPs were filtered out using the parental genes. Finally, 977,486 high-quality SNPs were obtained for further association analysis of the starch content traits.

### Association analysis of starch content in potato

**Fig 2:**
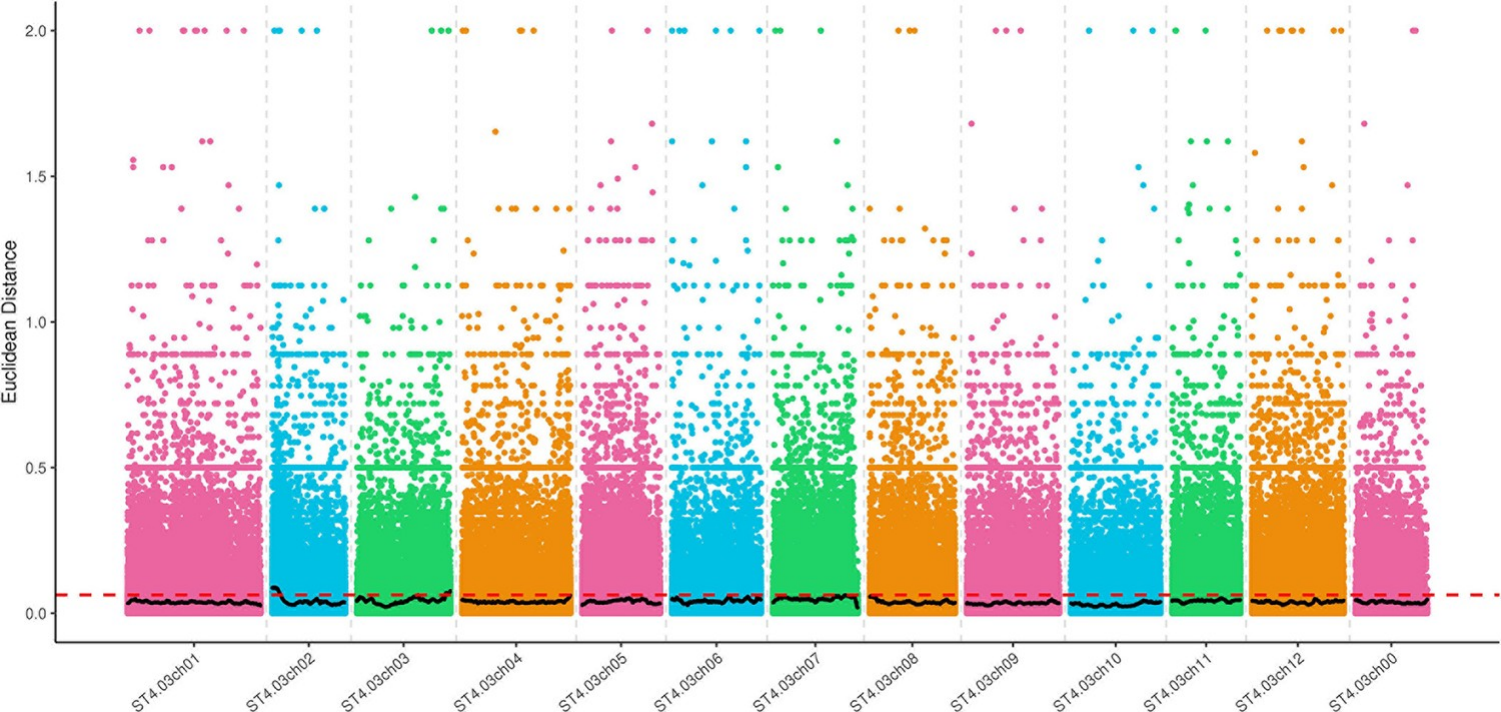
Distribution of ED correlation values on chromosomes. The abscisic coordinate is the potato chromosome abbreviation, the colored point represents the ED value of each SNP site, the black line represents the fitted ED value, and the red dotted line represents the significance correlation threshold. The higher the ED value, the better the correlation effect.

The ED values of 977,486 high-quality SNPs were calculated using the method described by Hill et al. (2013) [29]. After eliminating background noise, SNPNUM was used to fit the ED values of all sites, and the distribution of the associated values is shown in Fig 2. The median + 3SD of the fitted values of all sites was used as the association threshold between SNPs and starch traits, which was calculated to be 0.06. Fortunately, candidate regions associated with starch traits and above threshold values were detected on chromosomes 2, 3, and 7 respectively, with a total length of 8.90 Mb and 485 genes (Table 1).

**Table 1:** Statistics table for ED algorithm and SNP-index algorithm association regions.

Algorithm	Chromosome ID	Start	End	Size (Mb)	Gene number
ED	Chr02	0	5620000	5.62	123
	Chr03	59020000	62280000	3.26	355
	Chr07	48360000	48380000	0.02	7
SNP-index	Chr02	0	6370000	6.37	143
	Chr05	26160000	26380000	0.22	5
	Chr05	26400000	26440000	0.04	2
	Chr05	37170000	38150000	0.98	23
Intersection region	Chr02	0	5620000	5.62	123

**Fig 3:**
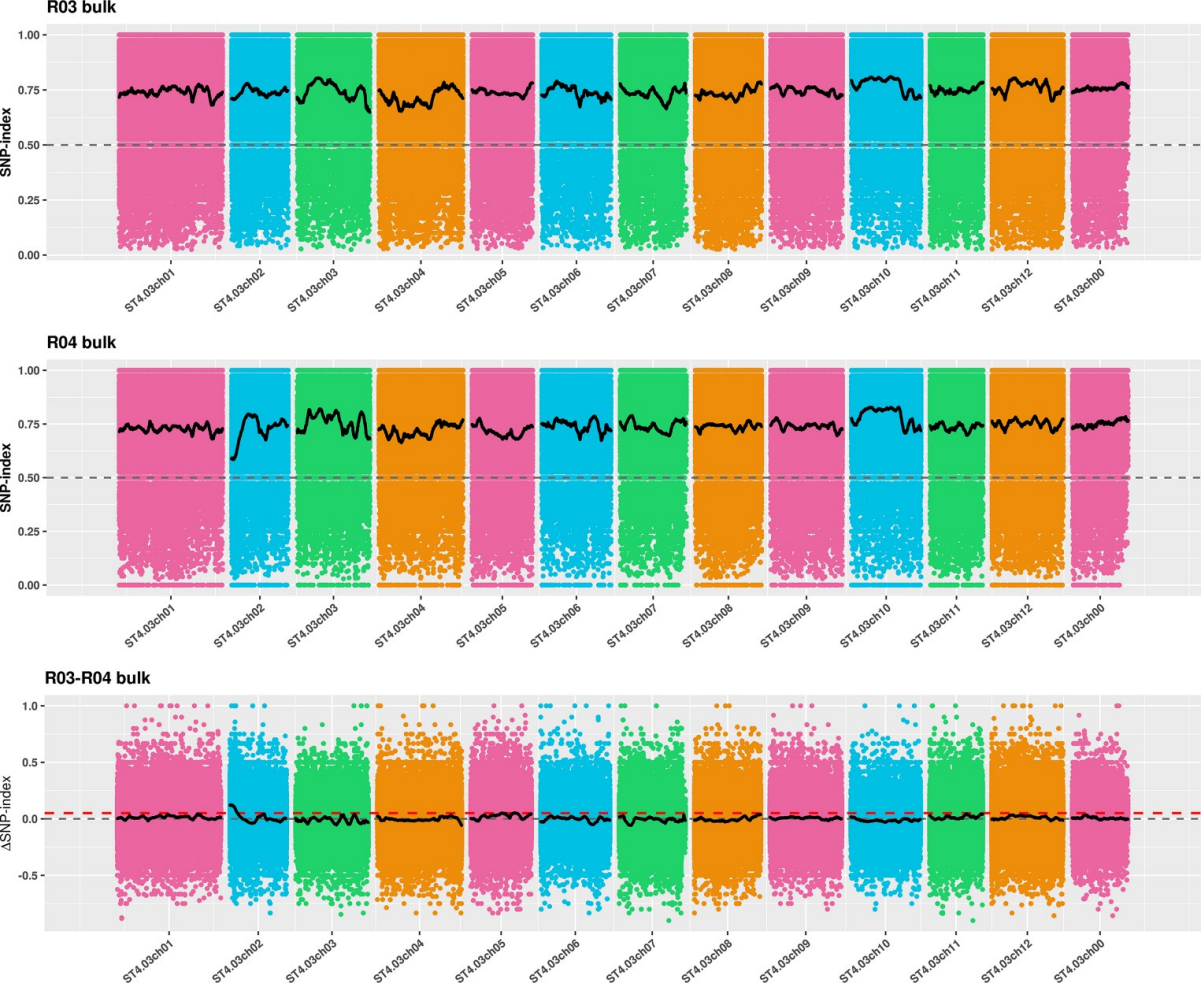
Distribution of SNP-index correlation values on chromosomes. (A) The graph shows the distribution of SNP-index values of the high starch mixing pool. (B) The graph shows the distribution of SNP-index values in the low starch mixing pool. (C) is the distribution of Δ(SNP-index) value. The abscissa is the name of potato chromosome, color dot represents calculated SNP-index (or Δ(SNP-index)), the black line for fitting the SNP-index (or Δ(SNP-index)). The red line represents the threshold line of the 99th percentile.

The SNP-index was calculated using 977,486 high-quality SNPs, and the SNP-index curves of H-pool and L-pool were plotted (Fig 3). SNPNUM method was used to fit Δ(SNP-index) values to eliminate false positive sites, and the regions above the threshold were selected as candidate regions for starch traits. However, as shown in Fig 3C, there was no high peak in the region with significant correlation, and the target site and its adjacent linkage sites did not exceed the theoretical threshold region. This demonstrated that no significant localization results could be found based on this threshold. Therefore, the threshold was lowered to find other regions. When the threshold value of the fitted SNP marker was 0.05 under the 99% confidence coefficient, one starch candidate region was detected on chromosome 2 and three starch candidate regions were detected on chromosome 5, with a total length of 7.64 Mb and 174 genes (Table 1).

The intersection of the results of the ED and SNP-index was taken as the final candidate region. The final starch candidate region was determined to be between 0 and 5.62 Mb on potato chromosome 2, and a total of 123 candidate genes were detected (Table 1).

### Functional annotations of starch candidate regions

SnpEff [28] software was used for SNP annotation in the starch candidate region between the parent and DNA pools. As shown in Table 2, the parents (YSP-4 × MIN-021) had 2,061 SNPs upstream (less than 5K), 15,160 SNPs in the intergenic region, 2,203 SNPs downstream (less than 5K), and 598 mutation sites on introns. SNP annotation of the mixed pool in the candidate region showed 1,366 SNPs upstream, 10,032 SNPs in the intergenic region, 1,314 SNPs downstream (within 5K), and 317 mutation sites on introns. According to the statistics, there were 376 non-synonymous SNPs between the parents and 256 non-synonymous SNPs between mixed pools. It is worth noting that these SNPs are likely to be directly related to potato starch traits, and the genes containing these SNPs are called non-synonymous genes.

**Table 2:** Statistics of SNP annotation results in candidate regions.

Type	R01 vs R02	R03 vs R04
Upstream (within 5K)	2061	1366
Downstream (within 5K)	2203	1314
Intron	598	317
Intergenic	15610	10032
Non synonymous coding	376	256
Synonymous coding	211	105
Total	21059	13390

**Note:** R01 represents the parent (YSP-4); R02 stands for father (MIN-021); R03 stands for high starch mixing pool; R04 stands for low starch mixing pool.

Based on the annotation information for DM in the potato reference genome, BLAST [31] was employed for deep annotation of multiple databases (NR [32], Swiss-Prot [33], GO [34], KEGG [35], COG [36]) with regard to coding genes in the candidate region. A total of 86 genes were annotated in the candidate region, among which 41 non-synonym genes were annotated among the parents. In addition, 41, 7, 2, 3 and 6 non-synonymous genes associated with starch candidate regions were annotated in NR, GO, KEGG, COG and SwissProt, respectively (Table 3).

**Table 3:** Statistics of gene function annotation results in candidate regions.

Annotated databases	NR	NT	trEMBL	SwissProt	GO	KEGG	COG	Total
Gene number	86	86	86	10	12	6	4	86
Non syn-gene number	41	41	41	6	7	2	3	41

GO analysis was performed and the differentially expressed genes for high and low starch traits in the candidate regions were classified. The genes were enriched in cellular components, molecular functions and biological processes [40]. Two differential genes were associated with cellular components, 6 with molecular function and 5 with biological processes. In the cell composition, the differential genes were mainly mapped to three subcategories: cells, organelles and cell parts. The differential genes annotated to molecular function were mainly clustered in two subcategories: catalytic activity and binding. Under biological processes, differential genes were mainly mapped into three subcategories: metabolic processes, cellular processes, and single-organism processes. These data suggest that starch formation may be related to the regulation of organelles (mitochondria and chloroplasts), or that a metabolic pathway may be involved in the regulation of starch. GO enrichment showed that there were 12 GO terms for differential genes associated with cellular components, among which the cytoplasmic part (GO: 0044444) and intracellular membrane-bounded organelle (GO: 0043231) were most significantly enriched. Under molecular functions, glucose metabolic processes (GO: 0006006) and photosynthesis (GO: 0015979) had the highest enrichment significance. Three GO terms had the most significant enrichment in biological processes, namely, oxidoreductase activity (GO: 0016491), nucleic acid binding (GO: 0003676) and cofactor binding (GO: 0048037) (S1–S4 Figs). The candidate genes had the highest enrichment significance in the bioprocess domain of glucose metabolism and photosynthesis, possibly because the genes affect starch synthesis by regulating this metabolic pathway. Analysis of the interaction pathways between different genes can help to further understand the function of the genes. Therefore, the KEGG database was used as a reference, and two genes in the starch candidate interval were found to be involved in two pathways (S5 Fig). PGSC0003DMG400006636 was involved in "carbon metabolism" (S6 Fig), and PGSC0003DMG400036713 was involved in "carbon fixation in photosynthetic organisms" (S7 Fig), indicating that these two metabolic pathways are closely related to starch synthesis.

The COG database was used for the direct homology classification of the three candidate genes. PGSC0003DMG400006636 and PGSC0003DMG400036713 were related to "metabolism", while PGSC0003DMG400037470 was related to "replication, recombination and repair" (S8 Fig).

### Prediction of potato starch candidate genes

The annotation results showed that 6 of the 41 candidate genes were most likely to be related to starch characteristics, namely PGSC0003DMG400017793, PGSC0003DMG400035245, PGSC0003DMG400036713, PGSC0003DMG400040452, PGSC0003DMG400006636 and PGSC0003DMG400044547. PGSC0003DMG400017793 encodes the pentatricopeptide repeat (PPR) family, which can maintain and regulate the normal expression of most genes in mitochondria and chloroplasts. It has been speculated that this gene may regulate starch synthesis by affecting mitochondria and chloroplasts [41]. PGSC0003DMG400035245 may control the mitochondrial protein at the end of CcmF, which is involved in the maturation of cytochrome C. Cytochrome C is the core component of the mitochondrial complex. It can affect changes in the mitochondrial ultrastructure and cause the loss of mitochondrial function, leading to changes in respiration and photosynthesis. This results in the variation of starch content [42]. PGSC0003DMG400036713 is related to "carbohydrate transport and metabolism" and may affect the production of 1, 3-diphosphoglycerate by regulating glyceraldehyde 3-phosphate dehydrogenase (NAD^+^) [43]. PGSC0003DMG400040452 may be involved in the encoding of integrase core domain proteins. PGSC0003DMG400006636 has high homology with the transporter gene ERD6-5, which encodes the sugar transporter involved in carbohydrate transport and metabolism [44]. PGSC0003DMG400044547 may regulate the mic60 protein in the MICOS complex subunit. These non-synonymous candidate genes are involved in starch metabolism pathways, regulate functional proteins, and play an important role in the synthesis of starch traits. In general, the candidate genes need to be further studied to clarify their functions.

### Development of CAPS markers related to starch content

**Fig 4:**
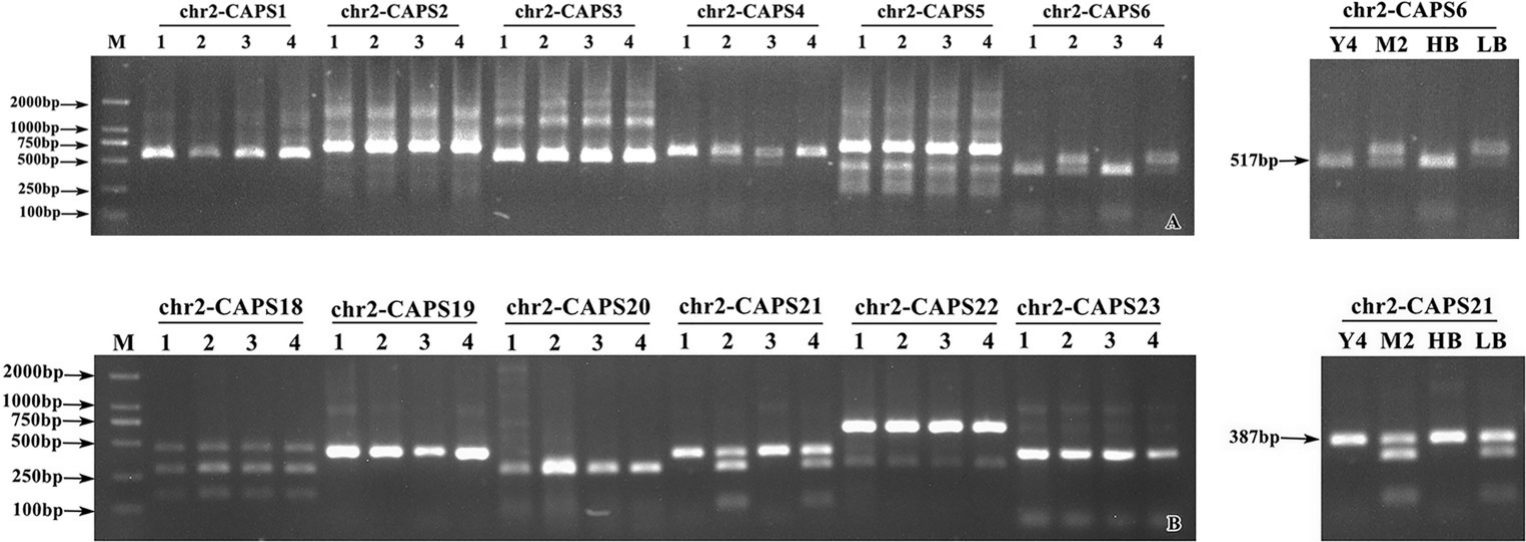
The screening of partial CAPS primers for potato. (A) chr2-CAPS6 enzyme digestion electrophoresis. (B) chr2-CAPS21 enzyme digestion electrophoresis. M. DL2000 Marker; 1. ♀ YSP-4; 2. ♂ MIN-021; 3. high starch pool; 4. low starch pool; chr2-CAPS1, chr2-CAPS2, chr2-CAPS3, chr2-CAPS4, chr2-CAPS5, chr2-CAPS6, chr2-CAPS18, chr2-CAPS19, chr2-CAPS20, chr2-CAPS21, chr2-CAPS22 and chr2-CAPS23 represents the partial primer.

Based on the results of previous sequencing and BSA analysis, the candidate range of potato starch traits was determined to be between 0–5.62 Mb on chromosome 2. In this section, 45 CAPS primers were designed based on the candidate genes (S1 Table). PCR amplification and enzyme digestion were performed using the genomic DNA of the parents and the mixed pools as templates, respectively. The specificity of enzyme digestion products was determined with 2% agarose gel electrophoresis. SNP-CAPS markers related to starch traits, named chr2-CAPS6 and chr2-CAPS21, were developed. The two markers showed restriction specificity between the parents and the mixed pool. In other words, 1 band was amplified between YSP-4 and the H-pool, and 2 bands were amplified between MIN-021 and the L-pool (Fig 4).

### Validation of markers in F2 segregation population

The 120 plants of tetraploid potato F_2_ contained 36 plants with high starch content (starch content ≥ 18%), 48 individuals with low starch content (starch content ≤ 15%) and 36 plants with medium starch content (starch content 15%-18%). Eighty-four individuals (36 with high starch and 48 with low starch) were selected to further validate the specificity of chr2-CAPS6 and chr2-CAPS21 markers.

**Fig 5:**
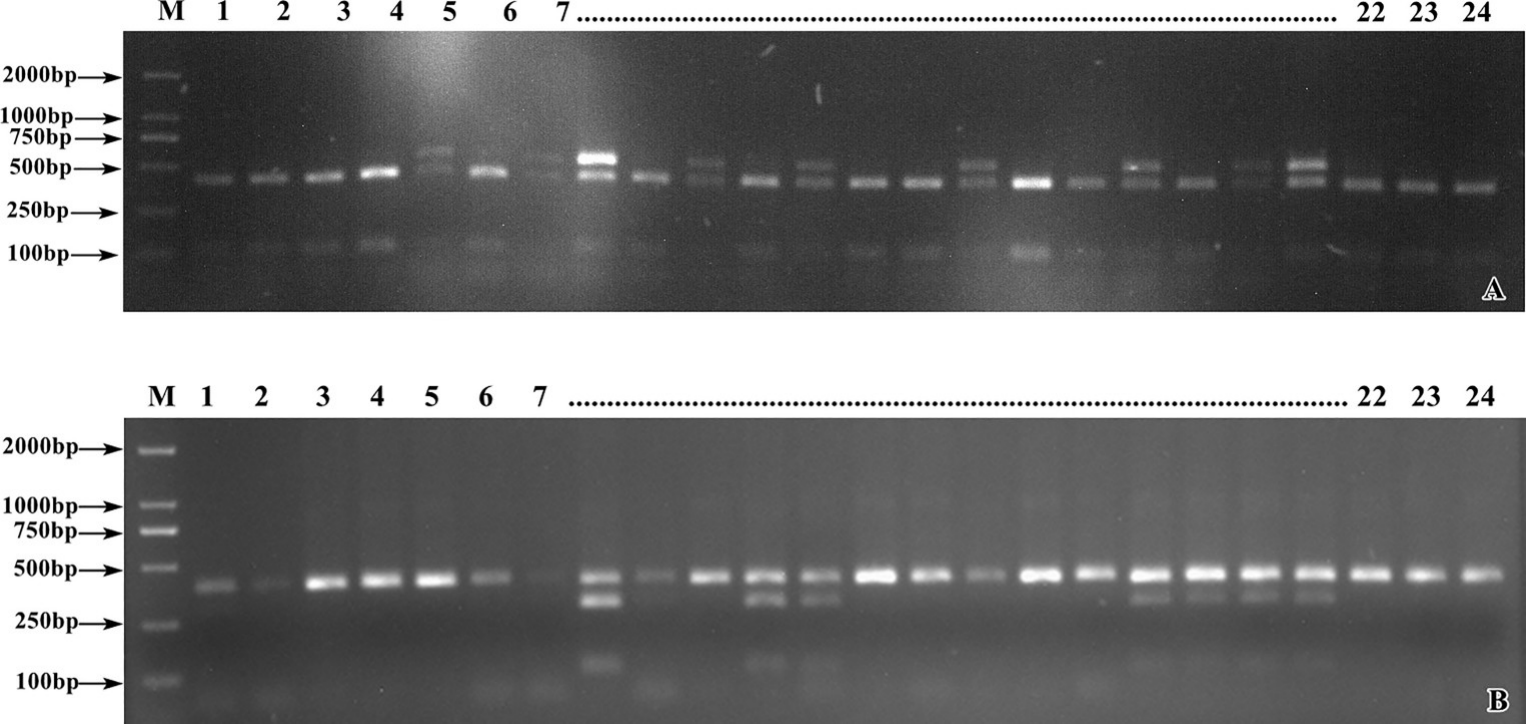
Partial results of enzyme digestion chr2-CAPS6 andchr2-CAPS21 markers for F_2_ population. (A) chr2-CAPS6 enzyme digestion electrophoresis. (B) chr2-CAPS21 enzyme digestion electrophoresis. M. DL2000 Marker; 1~24. F_2_ individuals.

The enzyme digestion results for the 84 F_2_ individual DNAs with the chr2-CAPS6 marker revealed that 32 of the 42 materials with negative labeling (1 band) had high starch content, and the correlation between the marker test results and phenotype was as high as 76.19%. Among the 42 materials with positive markers, 38 had low starch content materials, and the coincidence level between the labeling results and phenotype was 90.48%. In 84 extremely high or low-starch materials, 83.33% of the marker detection results were consistent with the starch content (Fig 5; Table 4). SPSS correlation analysis was conducted between starch content and marker detection results, and the correlation coefficient (r) was 0.61, with significant correlation at the 0.01 level (P < 0.01).

**Table 4:** Starch phenotype and marker detection of extreme materials in F_2_ population of potato.

Marker	Marker test	Number of samples	Phenotype	Number	Percentage (%)
chr2-SSR6	Positive	42	High starch content	4	90.48%
			Low starch content	38	
	Negative	42	High starch content	32	76.19%
			Low starch content	10	
chr2-SSR21	Positive	46	High starch content	7	84.78%
			Low starch content	39	
	Negative	38	High starch content	29	76.32%
			Low starch content	9	

The enzyme digestion results for chr2-CAPS21 showed that 29 of the 38 materials with negative marker (1 band) were high-starch materials, and the correlation between the marker detection results and phenotype was 76.32%. Among 46 materials with positive labels (2 bands), 39 were low-starch content materials, and the degree of correspondence between the labeling results and phenotype was 84.78%. In 84 extremely high or low-starch materials, 80.95% of the marker detection results were consistent with the starch content (Fig 5; Table 4). Correlation analysis using SPSS software revealed the correlation coefficient (r) was 0.57, with significant correlation at the 0.01 level (P < 0.01). In conclusion, chr2-CAPS6 and chr2-CAPS21 can accurately and effectively distinguish the starch content of the F_2_ segregation population, and are stable molecular markers closely linked with starch traits, which can be applied in subsequent marker-assisted breeding of high-starch potato.

### Detection of markers in tetraploid varieties (lines)

**Fig 6:**
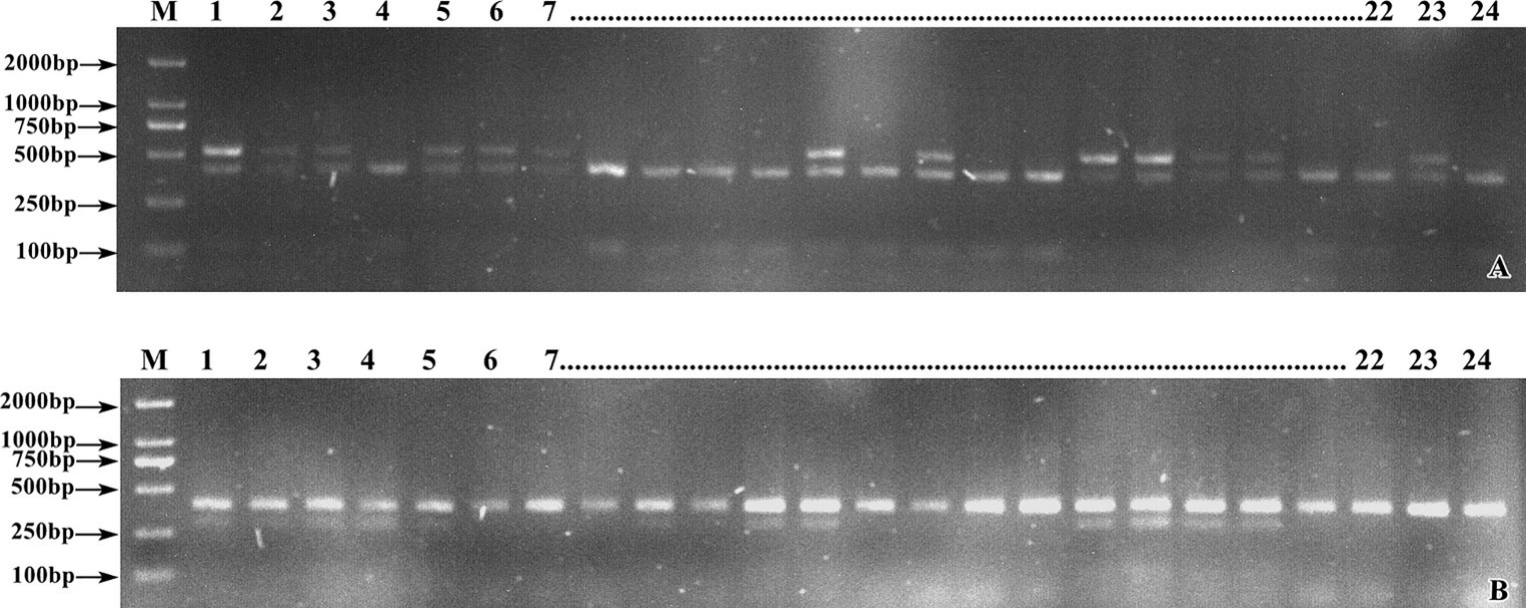
Enzymatic results marked in partial tetraploid and strains. (A) chr2-CAPS6 enzyme digestion electrophoresis. (B) chr2-CAPS21 enzyme digestion electrophoresis. M. DL2000 Marker; 1–24. Tetraploid varieties and strains.

Among the 24 tetraploid potato varieties (lines), 11 were high-starch and 13 were low-starch varieties (Table 5). The detection results of chr2-CAPS6 marker in 24 tetraploid potato varieties (strain) were as follows: Ten of the 13 positive markers showed low starch content, and the correlation between marker detection and phenotypic registration was 76.92%. Eight of the 11 cultivars with negative markers had high starch content, and the correspondence between the molecular markers and phenotypic results was 72.73% (Fig 6; Table 6). In the 24 potato varieties and strain, 75.00% of the marker detection results corresponded to the phenotypic results. Pearson’s bilateral correlation analysis was conducted between marker and phenotype, and the correlation coefficient (r) was 0.51, with significant correlation at the 0.01 level (P < 0.01).

**Table 5:** Potato varieties and strains information.

Number	Variety/strains	Starch Content (%)	Origin
1	L7×MIN-021 10#	14.71	China
2	V7	12.09	Holland
3	Burbank	18.34	America
4	Shapoti	14.73	Canada
5	Favorita	12.50	Holland
6	HM×YSP-4 6#	13.58	China
7	Jizhangshu 12#	14.90	China
8	Qingshu 9#	18.83	China
9	HM×MIN-021 5#	13.78	China
10	Huasong 7#	14.20	China
11	Houqihong	18.87	China
12	Hongmei	14.32	China
13	Longshu 3#	21.47	China
14	Longshu 6#	20.05	China
15	Longshu 7#	18.75	China
16	HM×MIN-021 9#	18.42	China
17	Heimeiren	13.65	China
18	Zicai 1#	14.89	China
19	Zicai 2#	14.87	China
20	Zaicai 3#	14.94	China
21	Neinongshu 1#	18.71	China
22	Neinongshu 2#	19.43	China
23	Neinongshu 3#	18.26	China
24	Neinongshu 4#	20.30	China

**Table 6:** Statistics of phenotype and mark detection results of starch in potato variety and strains.

Marker	Marker test	Sample size	Phenotype	Number	Percentage (%)
chr2-SSR6	Positive	13	High starch content	3	76.92%
			Low starch content	10	
	Negative	11	High starch content	8	72.73%
			Low starch content	3	
chr2-SSR21	Positive	12	High starch content	2	83.33%
			Low starch content	10	
	Negative	12	High starch content	9	75.00%
			Low starch content	3	

The detection results for chr2-CAPS21 were as follows: Ten of the 12 positive markers showed low starch content, and the correlation between marker detection and phenotypic registration was 83.33%. Nine of the 12 cultivars with negative markers had a high starch content, and the correspondence between molecular markers and phenotypic results was 75.00% (Fig 6 and Table 6). In the 24 potato varieties and strain, 79.17% of the marker detection results corresponded to the phenotypic results. Pearson’s bilateral correlation analysis was conducted between marker and phenotype, and the correlation coefficient (r) was 0.60, with significant correlation at the 0.01 level (P < 0.01). The above data indicate that chr2-CAPS6 and chr2-CAPS21 can distinguish the starch characteristics of tetraploid potato varieties and lines.

## Discussion

### Application of BSA and SLAF-seq strategies in mining candidate genes

BSA is a very practical method for gene mapping that constructs nearly isogenic mixed pools with target trait differences from isolated populations generated from parents with phenotypic differences. Appropriate primers are then selected to screen the molecular markers of the two gene pools. The molecular markers selected were identified in multiple individuals, and the markers linked to the target gene were finally determined. The purpose of this study was to test the materials for the tetraploid potato, the complex ploidy, high heterozygosity and self-cross decline; thus, it was difficult to construct stable genetic populations such as near-isogenic lines (NIL) and backcross inbred lines (BIL) to unify the genetic background. The BSA strategy can overcome this disadvantage, and does not require genotyping all individuals in the population, which can reduce the workload and cost.

The selection of extreme materials and the number of plants in the pool are important aspects of BSA technology. If the extreme phenotypes are not accurate, the analysis and judgment of the differential loci between the two extreme pools are limited, which will further affect the accuracy of the target trait genomic region [30]. Fekih et al. (2013) [45] showed that for a pool containing 20 individuals or more, the genetic background can be more effectively unified and noise can be eliminated. In this paper, 20 extreme plants were selected to construct pools based on the 2-year starch content, which could accurately locate the regions associated with the target traits.

There are a variety of molecular markers that can be combined with BSA for gene localization. SSR, inter-simple sequence repeat (ISSR), AFLP and SCAR markers are commonly used [46]. With the development of genome sequencing technology, molecular markers have also been transformed into functional markers corresponding to specific genes. The emergence of SNP has the advantages of high stability, wide distribution, high polymorphism and easy automatic analysis [47]. Site-specific amplified fragment sequencing (SLAF-seq), which can take advantage of SNP on a large scale, is cost-effective, fast, accurate, and suitable for genotyping all species. Better yet, the strategy of SLAF-seq combined with BSA allows rapid development of markers linked to the target trait, construction of genetic maps, QTL analysis, and gene mapping. At present, this method has been successfully applied to many species, such as maize [48], rice [16], pepper [17, 18] and cotton [19]. In this study, SLAF-seq combined with BSA was used for the first time to identify genomic regions and predict candidate genes for potato starch traits, and 6 important candidate genes were successfully identified.

### Identification of potato starch candidate regions

QTL mapping related to potato starch content traits has been performed for decades. As early as 1994, Freyre and Douches [9] detected tuber starch related QTLs on potato chromosomes 1, 2, 3, 5 and 7 by using traditional mapping. In 1998, Schäfer-Pregl et al. [49] used two different potato populations (K31 and LH) for localization analysis. In K31, starch QTLs were found to be located on all chromosomes except chromosome 11. Stable QTLs were detected on chromosomes 1, 2, 3 and 12 in LH under five different environmental conditions. Four years later, Menéndez et al. (2002) [50] detected QTL sites for glucose, fructose and sucrose traits, which are closely related to starch synthesis, distributed on all potato chromosomes under six different environments. In 2008, Li et al. [10] mapped starch QTLs to linkage groups 2, 3, 5, 7 and 11 using different potato genotypes as mapping populations. More recently, Sliwka et al. (2016) [13] detected a total of 12 QTLs related to starch traits in potato chromosomes 1, 2, 3, 8, 10, 11 and 12. The above studies showed that the genetic sites linked to potato starch feature may vary with the genetic background or environmental conditions of the mapped population, but potato chromosome 2 is considered to be a hot spot for controlling starch traits. For example, genes related to starch synthase (*SS* IV) [51] and starch synthase (*GBSS* II) [52] are located in this region.

There are two algorithms, ED and SNP-index, for locating sites associated with target traits. ED algorithms can remove most of the background noise without parental information and obtain high quality target character correlation regions, which are mostly used for research without parental information and difficult-to-build populations, such as forest trees [29]. On the contrary, SNP-index algorithms requires parental information to exclude SNP markers corresponding to but not linked to target traits, and then gain high-quality markers for association analysis [53]. ED and SNP-index algorithm were used for association analysis in our test, and the intersecting region for the two algorithms was selected as the final candidate region for potato starch traits. As expected, a candidate region was obtained at 0–5.62 Mb on chromosome 2, which was closely related to starch traits. The consequences were partially coincident with the interval mapped by previous researchers, suggesting that our experimental results were more accurate. It would be worth exploring new genes that regulate starch in a more systematic manner.

### Development of closely linked markers in tetraploid potato

Conventional breeding is easily affected by environmental conditions and genotypes, which results in low breeding efficiency and time consumption. However, molecular marker-assisted breeding can facilitate the combination of traditional breeding and markers, which can easily be used to select the target traits at the DNA level, shorten the breeding period and improve the efficiency. Furthermore, the key to the success of molecular assisted breeding lies in marker development. Tetraploid potatoes are characterized by complex ploidy and self-crossing decline. Therefore, diploid potato developmental molecular markers were applied in most studies to tetraploid test materials. This process tends to result in insufficient linkage between markers and traits and inaccurate correlation analysis. When applied in practice, it is easy to be affected by genetic, environmental conditions, gene interactions, etc. At present, there are few reports on the development of molecular markers related to important potato traits, and most of them focus on disease resistance. Marczewski et al. (2002) [54] successfully developed CAPS markers closely linked to the potato S virus resistant gene *Ns* using 119 F_1_ hybridization progenies of susceptible strain DW91-1187 and disease resistant DW83-3121 as test materials. Bryan et al. (2002) [55] developed the molecular marker SPUD 1636, which is closely linked to the resistance to the cyst nematode *Globodera pallida* in potatoes and can assist in the screening of potato individuals for resistance to *Globodera pallida*. Sliwka et al. (2016) [13] used 183 F_1_ populations generated by the hybridization of diploid potato DG 00–683 (male) and DG 08-28/13 (female) as test materials and developed a DArT marker related to sucrose content traits on potato chromosome 1. However, markers closely linked to potato starch have not been reported.

CAPS has been widely used in plant genotyping, location, cloning and variety identification owing to its co-dominance, site-specificity, simple operation, rapid detection and low cost [56]. SNP sites can be converted to CAPS markers because CAPS can quickly detect the SNPs that cause restriction site changes through digestion. In the development of SNP-CAPS markers, the development of CAPS based on non-synonymous SNPs in functional gene domains can not only effectively reflect the degree of influence of SNP polymorphism on the function of the whole functional gene, but also ensures the phenotypic polymorphism corresponding to functional genes is more closely correlated with the genotypic polymorphism detected by SNP-CAPS markers. In this study, the SNP-CAPS markers chr2-CAPS6 and chr2-CAPS21 were developed for the first time and confirmed using extremely high or low-starch individuals from the F_2_ population and tetraploid varieties. The high characterization rate in tetraploid parents, mixed pools and the F_2_ population confirmed the reliability of these two markers. It is of great significance for the analysis of potato germplasm resources, marker-assisted selection of functional genes and variety breeding.

## Conclusions

In this study, SLAF-seq combined with BSA strategy was used for the first time to map a genomic region related to starch traits at 0–5.62 Mb on chromosome 2 of tetraploid potato. Within this range, 376 non-synonymous SNPs and 41 non-synonymous genes were found among the parents. According to the annotations, further analysis was conducted and 6 candidate genes were found to be closely related to starch traits. The functions of these genes need to be further investigated. The development of chr2-CAPS6 and chr2-CAPS21 linked to starch traits based on candidate genes is innovative and practical. In summary, these findings will promote the progress of tetraploid potato genomics and molecular marker-assisted breeding to some extent.

## Supporting information

S1 Fig GO annotation clustering diagram of genes in the candidate region. The x-coordinate represents the contents of each GO classification, the left coordinate represents the percentage of the number of genes, and the right coordinate represents the number of genes. The figure shows the gene classification of GO secondary functions in the context of all genes in the associated region.
(TIF)

S2 Fig TopGO directed acyclic graphof biological process domain GO terms in potato starch candidate region. The boxes in the diagram represent the most significant term, and the diagram also contains the corresponding relationships between their layers. The content description and enrichment significance value of the GO term are given in each box(ellipse). Different colors represent different enrichment significance levels, and the darker the color, the higher the significance.
(TIF)

S3 Fig TopGO directed acyclic graph of cellular component domain GO terms in potato starch candidate region. The boxes in the diagram represent the most significant term, and the diagram also contains the corresponding relationships between their layers. The content description and enrichment significance value of the GO term are given in each box(ellipse). Different colors represent different enrichment significance levels, and the darker the color, the higher the significance.
(TIF)

S4 Fig TopGO directed acyclic graph of molecular function domain GO terms in potato starch candidate region. The boxes in the diagram represent the most significant term, and the diagram also contains the corresponding relationships between their layers. The content description and enrichment significance value of the GO term are given in each box(ellipse). Different colors represent different enrichment significance levels, and the darker the color, the higher the significance.
(TIF)

S5 Fig Pathway map of genes in the candidate region. The x-coordinate is the ratio of the number of genes annotated to the total number of annotated genes, and the y-coordinate is the name of the KEGG metabolic pathway.
(TIF)

S6 Fig The “carbon metabolism” pathway map associated with genes in the strach candidate region. The red box indicates the genes in the associated region, while the blue box indicates all the enzymes needed for this pathway, indicating that the corresponding genes are related to this enzyme. The genes in the associated regions associated with this pathway are highlighted in red.
(TIF)

S7 Fig The “carbon fixation in photosynthetic organisms” pathway map associated with genes in the strach candidate region. The red box indicates the genes in the associated region, while the blue box indicates all the enzymes needed for this pathway, indicating that the corresponding genes are related to this enzyme. The genes in the associated regions associated with this pathway are highlighted in red.
(TIF)

S8 Fig COG annotation classification map of SNP genes in the candidate region. The abscess is the content of COG classification, and the ordinate is the number of genes.
(TIF)

S1 Table CAPS primers and restriction endonuclease. (DOCX)

S1 Raw images (PDF)

S1 File (ZIP)

S2 File (ZIP)

S3 File (ZIP)

## References

[1] Liu YH, Wang KL, Deng C, Warran B, Wang L, Yu B, et al. Comparative transcriptome analysis of white and purple potato to identify genes involved in Anthocyanin biosynthesis. PLoS One. 2015; 10(6): e0191406. doi: 10.1371/journal.pone.0129148 26053878 PMC4459980

[2] Jackson SD. Multiple signaling pathways control tuber induction in potato. Plant Physiol. 1999; 119(1): 1–8. doi: 10.1104/pp.119.1.1 9880339 PMC1539201

[3] Spooner DM, Ghislain M, Simon R, Jansky SH, Gavrilenko T. Systematics, diversity, genetics, and evolution of wild and cultivated potatoes. Bot. Rev. 2014; 80(4): 283–383. doi: 10.1007/s12229-014-9146-y

[4] Abbasi KS, Masud T, Gulfraz M, Ali S, Imran M. Physico-chemical, functional and processing attributes of some potato varieties grown in Pakistan. Afr. J. Biotechnol. 2011; 10(84): 19570–19579. doi: 10.5897/AJB11.566

[5] Ebúrneo JM, Garcia EL, Rodrigues dos Santos TP, Soratto RP, Fernandes AM, Leonel M. Influence of nitrogen fertilization on the characteristics of potato starch. Aust J Crop Sci. 2018; 12(3): 365–373. doi: 10.21475/ajcs.18.12.03.pne680

[6] Tang HJ, Mitsunaga T, Kawamura Y. Molecular arrangement in blocklets and starch granule architecture. Carbohydr Polym. 2006; 63(4): 555–560. doi: 10.1016/j.carbpol.2005.10.016

[7] Lachman J, Hamouz K, Orsák M, Pivec V, Dvořák P. The influence of flesh colour and growing locality on polyphenolic content and antioxidant activity in potatoes. Sci Hortic. 2008; 117(2): 109–114. doi: 10.1016/j.scienta.2008.03.030

[8] Singh N, Singh J, Kaur L, Sodhi NL, Gill BS. Morphological, thermal and rheological properties of starches from different botanical sources. J Sci Food Agric. 2003; 82(12): 219–231. doi: 10.1002/jsfa.1194

[9] Freyre R, Warnke S, Sosinski B, Douches DS. Quantitative trait locus analysis of tuber dormancy in diploid potato (*Solarium spp*.). Theor Appl Genet. 1994; 89(4): 474–480. doi: 10.1007/BF00225383 24177897

[10] Li L, Paulo MJ, Strahwald J, Lübecket J, Hofferbert HR, Tacke E, et al. Natural DNA variation at candidate loci is associated with potato chip color, tuber starch content, yield and starch yield. Theor Appl Genet. 2008; 116(8): 1167–1181. doi: 10.1007/s00122-008-0746-y 18379755 PMC2358939

[11] Bradshaw JE, Hackett CA, Pande B, Waugh R, Bryan GJ. QTL mapping of yield, agronomic and quality traits in tetraploid potato (*Solanum tuberosum subsp*. *Tuberosum*). Theor Appl Genet. 2008; 116(2): 193–211. doi: 10.1007/s00122-007-0659-1 17938877

[12] Werij JS, Furrer H, Eck HJ, Visser RGF, Bachem CWB. A limited set of starch related genes explain several interrelated traits in potato. Euphytica. 2012; 186(2): 501–516. doi: 10.1007/s10681-012-0651-y

[13] Sliwka J, Soltys-Kalina D, Szajko K, Wasilewicz-Flis I, Strzelczyk-Żyta D, Zimnoch-Guzowska E, et al. Mapping of quantitative trait loci for tuber starch and leaf sucrose contents in diploid potato. Theor Appl Genet. 2016; 129(1): 131–140. doi: 10.1007/s00122-015-2615-9 26467474 PMC4703618

[14] Michelmore RW, Paran I, Kesseli RV. Identification of markers linked to disease-resistance genes by bulked segregant analysis: A rapid method to detect markers in specific genomic regions by using segregating populations. Proc Natl Acad Sci USA. 1991; 88(21): 9828–9832. doi: 10.1073/pnas.88.21.9828 1682921 PMC52814

[15] Sun XW, Liu DY, Zhang XF, Li WB, Liu H, Hong WG, et al. SLAF-seq: An efficient method of large-scale *De Novo* SNP discovery and genotyping using high-throughput sequencing. PLoS One. 2013; 8(3): e58700. doi: 10.1371/journal.pone.0058700 23527008 PMC3602454

[16] Xu FF, Sun X, Chen YL, Huang Y, Tong C, Bao JS. Rapid identification of major QTLs associated with rice grain weight and their utilization. PLoS One. 2015; 10(3): e0122206. doi: 10.1371/journal.pone.0122206 25815721 PMC4376791

[17] Zhang XF, Wang GY, Chen B, Du HS, Zhang FG, Zhang HY. et al. Candidate genes for first flower node identified in pepper using combined SLAF-seq and BSA. PLoS Ons. 2018; 13(3): e0194071. doi: 10.1371/journal.pone.0194071 29558466 PMC5860747

[18] Wang GY, Chen B, Du HS, Zhang FL, Zhang HY, Wang YQ, et al. Genetic mapping of anthocyanin accumulation-related genes in pepper fruits using a combination of SLAF-seq and BSA. PLoS One. 2018; 13(9): e0204690. doi: 10.1371/journal.pone.0204690 30261055 PMC6160195

[19] Zhao CP, Zhao GY, Zhao G, Wang ZX, Wang KH, Liu SE, et al. Physical mapping and candidate gene prediction of fertility restorer gene of cytoplasmic male sterility in cotton. BMC Genomics. 2018; 19(1): 6. doi: 10.1186/s12864-017-4406-y 29295711 PMC5751606

[20] Zhang H, Yi HP, Wu MZ, Zhang YB, Zhang XJ, Li MH, et al. Mapping the flavor contributing traits on "Fengwei melon" (*Cucumis melo* L.) chromosomes using parent resequencing and super bulked-segregant analysis. PLoS One. 2016; 11(2): e0148150. doi: 10.1371/journal.pone.0148150 26840947 PMC4739687

[21] Dong W, Wu DF, Li GS, Wu DW, Wang ZC. Next-generation sequencing from bulked segregant analysis identifies a dwarfism gene in watermelon. Sci Rep. 2018; 8(1): 2908. doi: 10.1038/s41598-018-21293-1 29440685 PMC5811605

[22] Li XC, Xu JF, Duan SG, Zhang JJ, Bian CS, Hu J, et al. Mapping and QTL analysis of early-maturity traits in tetraploid potato (*Solanum tuberosum* L.). Int J Mol Sci. 2018; 19(10): 3065. doi: 10.3390/ijms19103065 30297627 PMC6213731

[23] Xu YM, Li YM, Jia YX, Zhang CZ, Li CH, Huang SW, et al. Fine mapping and candidate genes analysis for regulatory gene of anthocyanin synthesis in red-colored tuber flesh. Zhongguo Nong Ye Ke Xue. 2019; 52(15): 2678–2685. doi: 10.3864/j.issn.0578-1752.2019.15.011

[24] Zhang YC, Tian F. Research methods of potato experiment. 1st ed. Beijing: China Agricultural Science and Technology Press; 2007.

[25] Li H, Durbin R. Fast and accurate short read alignment with Burrows-Wheeler transform. Bioinformatics. 2009; 25(14): 1754–1760. doi: 10.1093/bioinformatics/btp324 19451168 PMC2705234

[26] Mckenna A, Hanna M, Banks E, Sivachenko A, Cibulskis K, Kernytsky A, et al. The genome analysis toolkit: A map reduce framework for analyzing next-generation DNA sequencing data. Genome Res. 2010; 20(9): 1297–1303. doi: 10.1101/gr.107524.110 20644199 PMC2928508

[27] Li H, Handsaker B, Wysoker A, Fennell T, Ruan J, Homer N, et al. The sequence alignment/map (SAM) format and SAMtools. Bioinformatics. 2009; 25(16): 2078–2079. doi: 10.1093/bioinformatics/btp352 19505943 PMC2723002

[28] Cingolani P, Platts A, Wang LL, Coon M, Nguyen T, Wang L, et al. A program for annotating and predicting the effects of single nucleotide polymorphisms, SnpEff: SNPs in the genome of *Drosophila melanogaster* strain w^1118^; iso-2; iso-3. Fly (Austin). 2012; 6(2): 80–92. doi: 10.4161/fly.19695 22728672 PMC3679285

[29] Hill JT, Demarest BL, Bisgrove BW, Gorsi B, Su YC, Yost HJ. MMAPPR: mutation mapping analysis pipeline for pooled RNA-seq. Genome Res. 2013; 23(4): 687–697. doi: 10.1101/gr.146936.112 23299975 PMC3613585

[30] Takagi H, Abe A, Yoshida K, Kosugi S, Natsume S, Mitsuoka C, et al. QTL-seq: rapid mapping of quantitative trait loci in rice by whole genome resequencing of DNA from two bulked populations. Plant J. 2013; 74(1): 174–183. doi: 10.1111/tpj.12105 23289725

[31] Kent WJ. BLAT-the BLAST-like alignment tool. Genome Res. 2002; 12(4): 656–664. doi: 10.1101/gr.229202 11932250 PMC187518

[32] Deng YY, Li JQ, Wu SF, Zhu YP, Chen YW, He FC. Integrated nr database in protein annotation system and its localization. Computer Engineering. 2006; 32(5): 71–73+76. doi: 10.3969/j.issn.1000-3428.2006.05.026

[33] Yip YL, Famiglietti M, Gos A, Duek PD, David FPA, Gateau A, et al. Annotating single amino acid polymorphisms in the UniProt/Swiss-Prot knowledgebase. Hum Mutat. 2008; 29(3): 361–366. doi: 10.1002/humu.20671 18175334

[34] Ashburner M, Ball CA, Blake JA, Botstein D, Butler H, Cherry JM, et al. Gene ontology: Tool for the unification of biology. Nat Genet. 2000; 25(1): 25–29. doi: 10.1038/75556 10802651 PMC3037419

[35] Kanehisa M, Goto S, Kawashima S, Okuno Y, Hattori M. The KEGG resource for deciphering the genome. Nucleic Acids Res. 2004; 32(suppl 1): D277–D280. doi: 10.1093/nar/gkh063 14681412 PMC308797

[36] Tatusov RL, Galperin MY, Natale DA, Koonin EV. The COG database: A tool for genome-scale analysis of protein functions and evolution. Nucleic Acids Res. 2000; 28(1): 33–36. doi: 10.1093/nar/28.1.33 10592175 PMC102395

[37] Zhang XY, Gao YN. To design PCR primers with Oligo 6 and Primer Premier 5. Shengwu Xinxixue. 2004; 2(4): 15–18+46. CNKI: SUN: XXSW.0.2004-04-003

[38] McCord PH, Sosinski BR, Haynes KG, Clough ME, Yencho GC. Linkage mapping and QTL analysis of agronomic traits in tetraploid potato (*Solarium tubersum subsp*. *Tuberosum*). Crop Sci. 2011; 51(2): 771–785. doi: 10.2135/cropsci2010.02.0108

[39] Yu XX, Zhang MF, Yu Z, Yang DS, Li JW, Wu GF, et al. An SNP-based high-density genetic linkage map for tetraploid potato using specific length amplified fragment sequencing (SLAF-Seq) technology. Agronomy. 2020; 10(1): 1–14. doi: 10.3390/agronomy10010114

[40] Alexa A, Rahnenfuhrer J. topGO: Enrichment analysis forgene ontology. 2010. Available: http://www.bioconductor.org/packages/release/bioc/html/topGO.html. Accessed 2014 December 10.

[41] She KC, Kusano H, Koizumi K, Yamakawa H, Hakata M, Imamura T, et al. A novel factor *FLOURY ENDOSPERM2* is involved in regulation of rice grain size and starch quality. Plant Cell. 2010; 22(10): 3280–3294. doi: 10.1105/tpc.109.070821 20889913 PMC2990130

[42] Giegé P, Grienenberger JM, Bonnard G. Cytochrome c biogenesis in mitochondria. Mitochondrion. 2008; 8(1): 61–73. doi: 10.1016/j.mito.2007.10.001 18033741

[43] Matsuo S, Kikuchi K, Fukuda M, Honda I, Imanishi S. Roles and regulation of cytokinins in tomato fruit development. J Exp Bot. 2012; 63(15): 5569–5579. doi: 10.1093/jxb/ers207 22865911 PMC3444270

[44] Ma XL, Liu YH, Yuan ZL, Shi YS, Song YC, Wang TY, et al. Cloning of cDNAs for a novel sugar transporter gene, *ZmERD6*, from maize and its expression analysis under abiotic stresses. Zuo Wu Xue Bao. 2009; 35(8): 1410–1417. doi: 10.3724/SP.J.1006.2009.01410

[45] Fekih R, Takagi H, Tamiru M, Abe A, Natsume S, Yaegashi H, et al. MutMap+: Genetic mapping and mutant identification without crossing in rice. PloS One. 2013; 8(7): e68529. doi: 10.1371/journal.pone.0068529 23874658 PMC3707850

[46] Tiwari S, SL K, Kumar V, Singh B, Rao AR, SV AM, et al. Mapping QTLs for salt tolerance in rice (*Oryza sativa* L.) by bulked segregant analysis of recombinant inbred lines using 50K SNP Chip. PloS One. 2016; 11(4): e0153610. doi: 10.1371/journal.pone.0153610 27077373 PMC4831760

[47] Lander ES. The new genomics: global views of biology. Science. 1996; 274(5287): 536–539. doi: 10.1126/science.274.5287.536 8928008

[48] Haase NJ, Beissinger T, Hirsch CN, Vaillancourt B, Deshpande S, Barry K, et al. Shared genomic regions between derivatives of a large segregating population of Maize identified using bulked segregant analysis sequencing and traditional linkage analysis. G3 (Bethesda). 2015; 5(8): 1593–1602. doi: 10.1534/g3.115.017665 26038364 PMC4528316

[49] Schäfer-Pregl R, Ritter E, Concilio L, Hesselbach J, Lovatti L, Walkemeier B, et al. Analysis of quantitative trait loci (QTLs) and quantitative trait alleles (QTAs) for potato tuber yield and starch content. Theor Appl Genet. 1998; 97: 834–846. doi: 10.1007/s001220050963

[50] Menéndez CM, Ritter E, Schäfer-Pregl R, Walkemeier B, Kalde A, Salamini F, et al. Cold sweetening in diploid potato: mapping quantitative trait loci and candidate genes. Genetics. 2002; 162(3): 1423–1434. doi: 10.1093/genetics/162.3.1423 12454085 PMC1462350

[51] Schönhals EM, Ortega F, Barandalla L, Aragones A, Ruiz de Galarreta JI, Liao JC, et al. Identification and reproducibility of diagnostic DNA markers for tuber starch and yield optimizationin a novel association mapping population of potato (*Solarium tuberosum* L.). Theor Appl Genet. 2016; 129(4): 767–785. doi: 10.1007/s00122-016-2665-7 26825382 PMC4799268

[52] Chen X, Salamini F, Gebhardt C. A potato molecular-function map for carbohydrate metabolism and transport. Theor Appl Genet. 2001; 102(2): 284–295. doi: 10.1007/s001220051645

[53] Abe A, Kosugi S, Yoshida K, Natsume S, Takagi H, Kanzaki H, et al. Genome sequencing reveals agronomically important loci in rice using MutMap. Nat Biotechnol. 2012; 30(2): 174–178. doi: 10.1038/nbt.2095 22267009

[54] Marczewski W, Hennig J, Gebhardt C. The *Potato virus S* resistance gene *Ns* maps to potato chromosome VIII. Theor Appl Genet. 2002; 105(4): 564–567. doi: 10.1007/s00122-002-0976-3 12582505

[55] Bryan GJ, McLean K, Bradshaw JE, De Jong WS, Phillips M, Castelli L, et al. Mapping QTLs for resistance to the cyst nematode *Globodera pallida* derived from the wild potato *species Solanum vernei*. Theor Appl Genet. 2002; 105(1): 68–77. doi: 10.1007/s00122-002-0873-9 12582563

[56] Konieczny A, Ausubel FM. A procedure for mapping *Arabidopsis* mutations using co-dominant ecotype-specific PCR-based markers. Plant J. 1993; 4(2): 403–410. doi: 10.1046/j.1365-313x.1993.04020403.x 8106085

