# Supplementary figures and images for: Identification of starch candidate genes using SLAF-seq and BSA strategies and development of related SNP-CAPS markers in tetraploid potato (part 1 of 2)
Source: PLoS One. 2021 Dec 21;16(12):e0261403. doi: 10.1371/journal.pone.0261403 (PMC8691606; doi:10.1371/journal.pone.0261403)

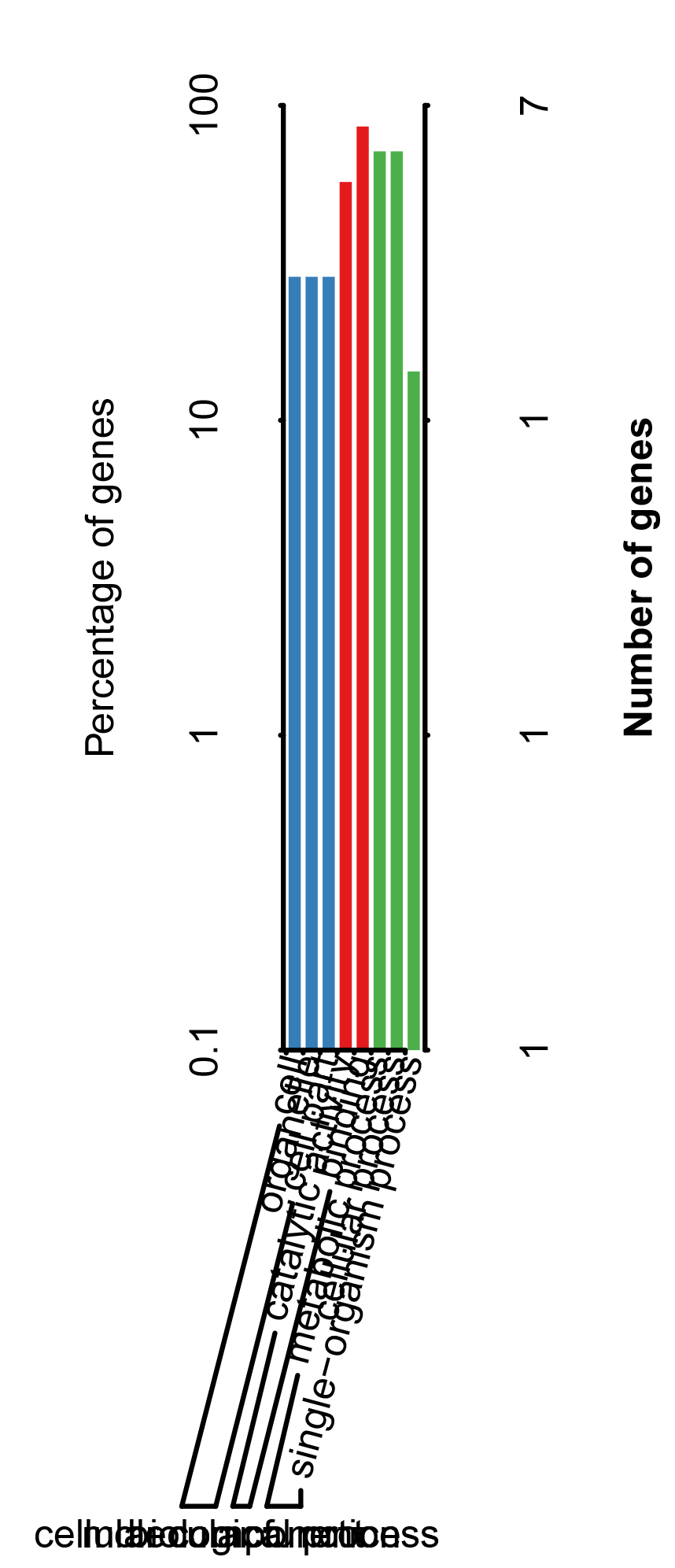

Supplement: S1 Fig — The x-coordinate represents the contents of each GO classification, the left coordinate represents the percentage of the number of genes, and the right coordinate represents the number of genes. The figure shows the gene classification of GO secondary functions in the context of all genes in the associated region. (TIF) [file pone.0261403.s001.tif]

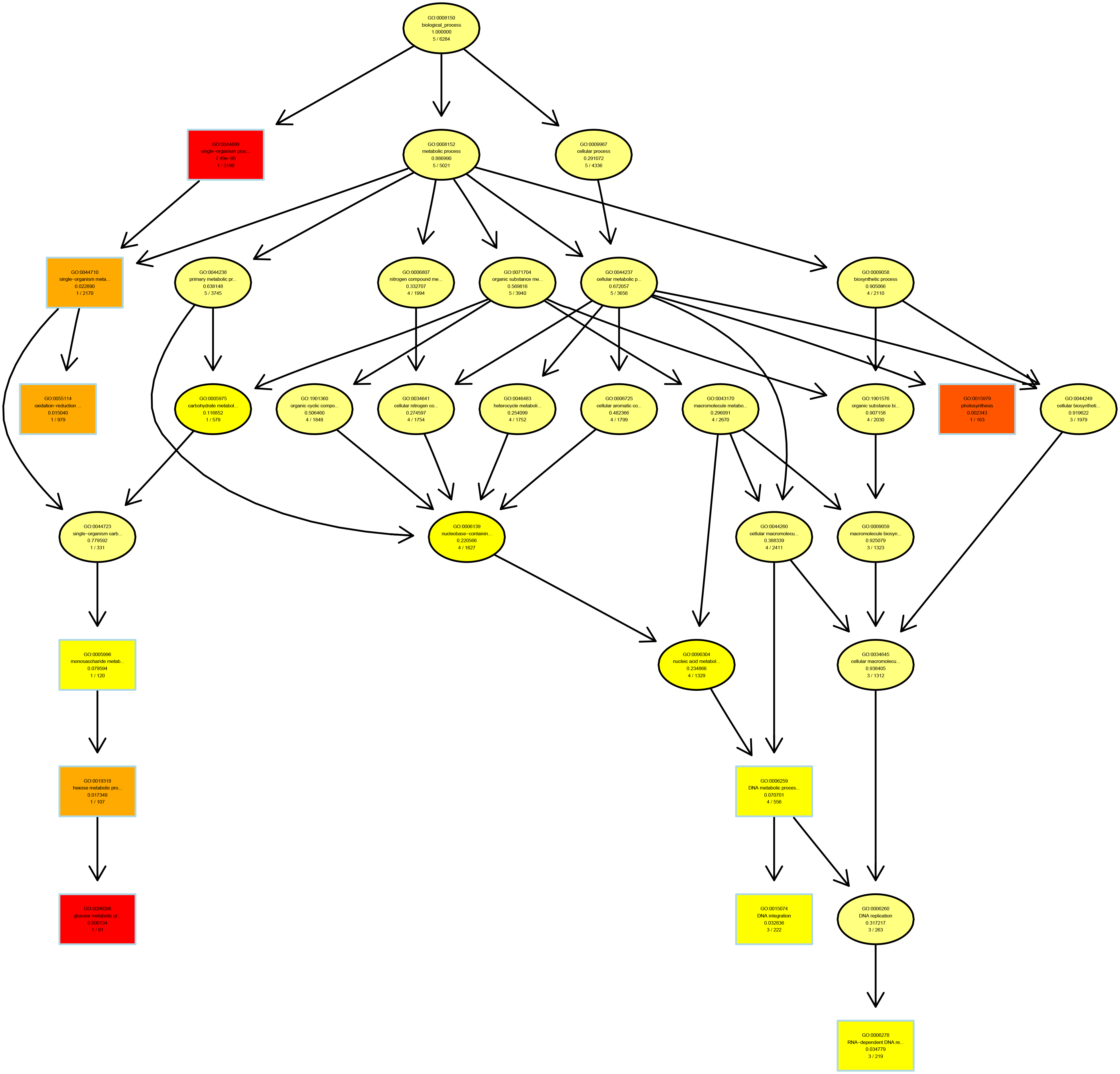

Supplement: S2 Fig — The boxes in the diagram represent the most significant term, and the diagram also contains the corresponding relationships between their layers. The content description and enrichment significance value of the GO term are given in each box(ellipse). Different colors represent different enrichment significance levels, and the darker the color, the higher the significance. (TIF) [file pone.0261403.s002.tif]

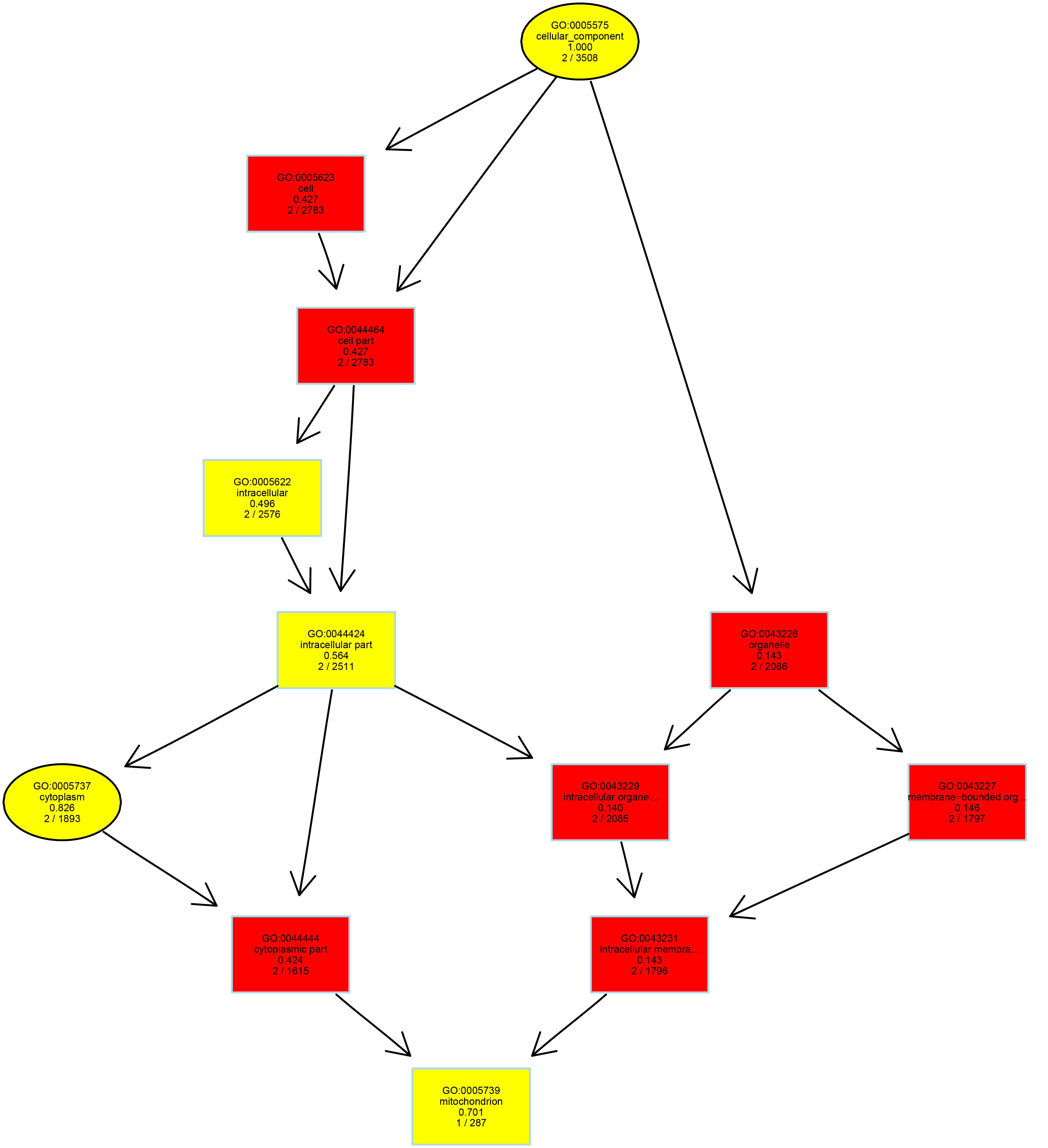

Supplement: S3 Fig — The boxes in the diagram represent the most significant term, and the diagram also contains the corresponding relationships between their layers. The content description and enrichment significance value of the GO term are given in each box(ellipse). Different colors represent different enrichment significance levels, and the darker the color, the higher the significance. (TIF) [file pone.0261403.s003.tif]

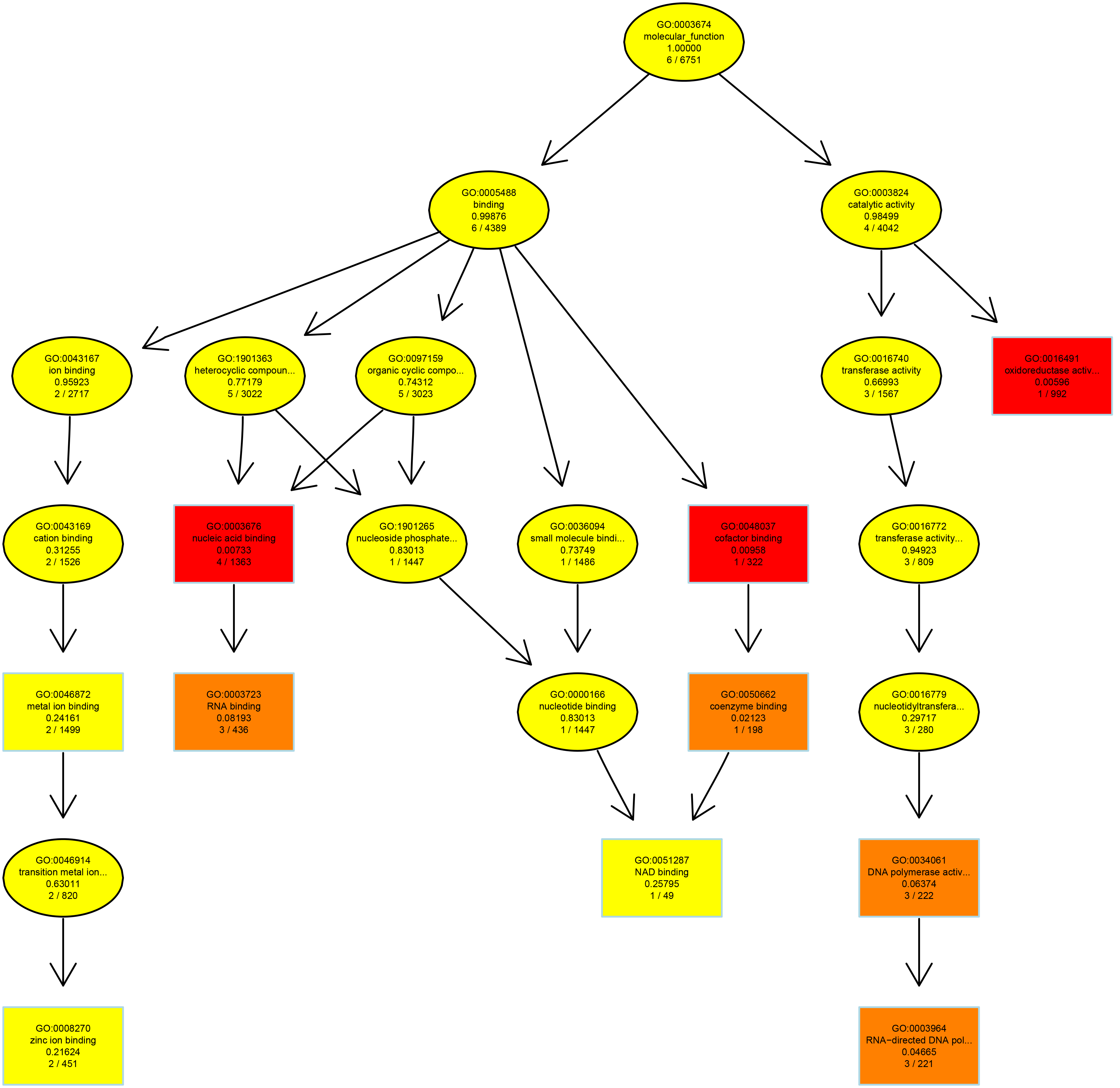

Supplement: S4 Fig — The boxes in the diagram represent the most significant term, and the diagram also contains the corresponding relationships between their layers. The content description and enrichment significance value of the GO term are given in each box(ellipse). Different colors represent different enrichment significance levels, and the darker the color, the higher the significance. (TIF) [file pone.0261403.s004.tif]

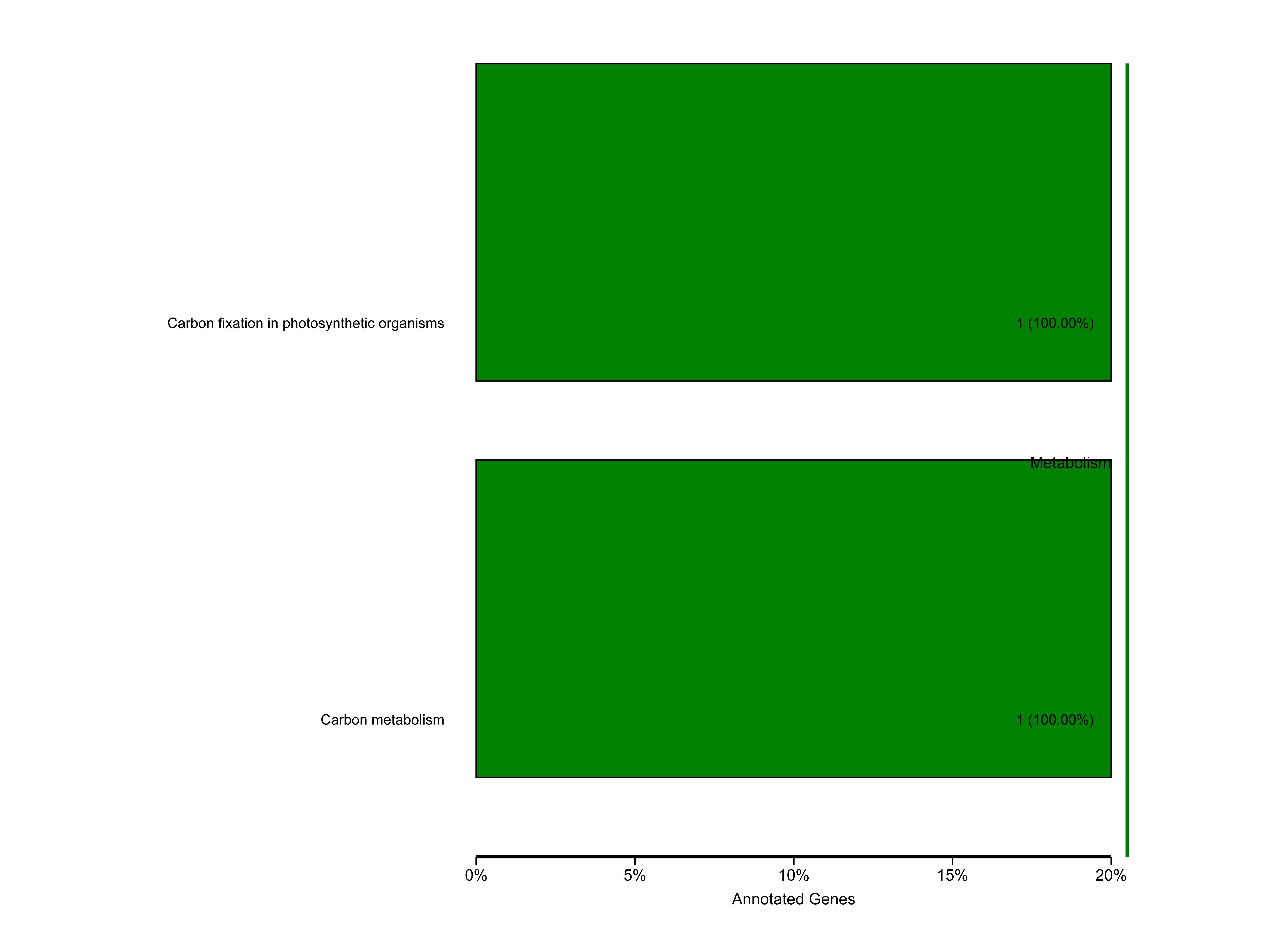

Supplement: S5 Fig — The x-coordinate is the ratio of the number of genes annotated to the total number of annotated genes, and the y-coordinate is the name of the KEGG metabolic pathway. (TIF) [file pone.0261403.s005.tif]

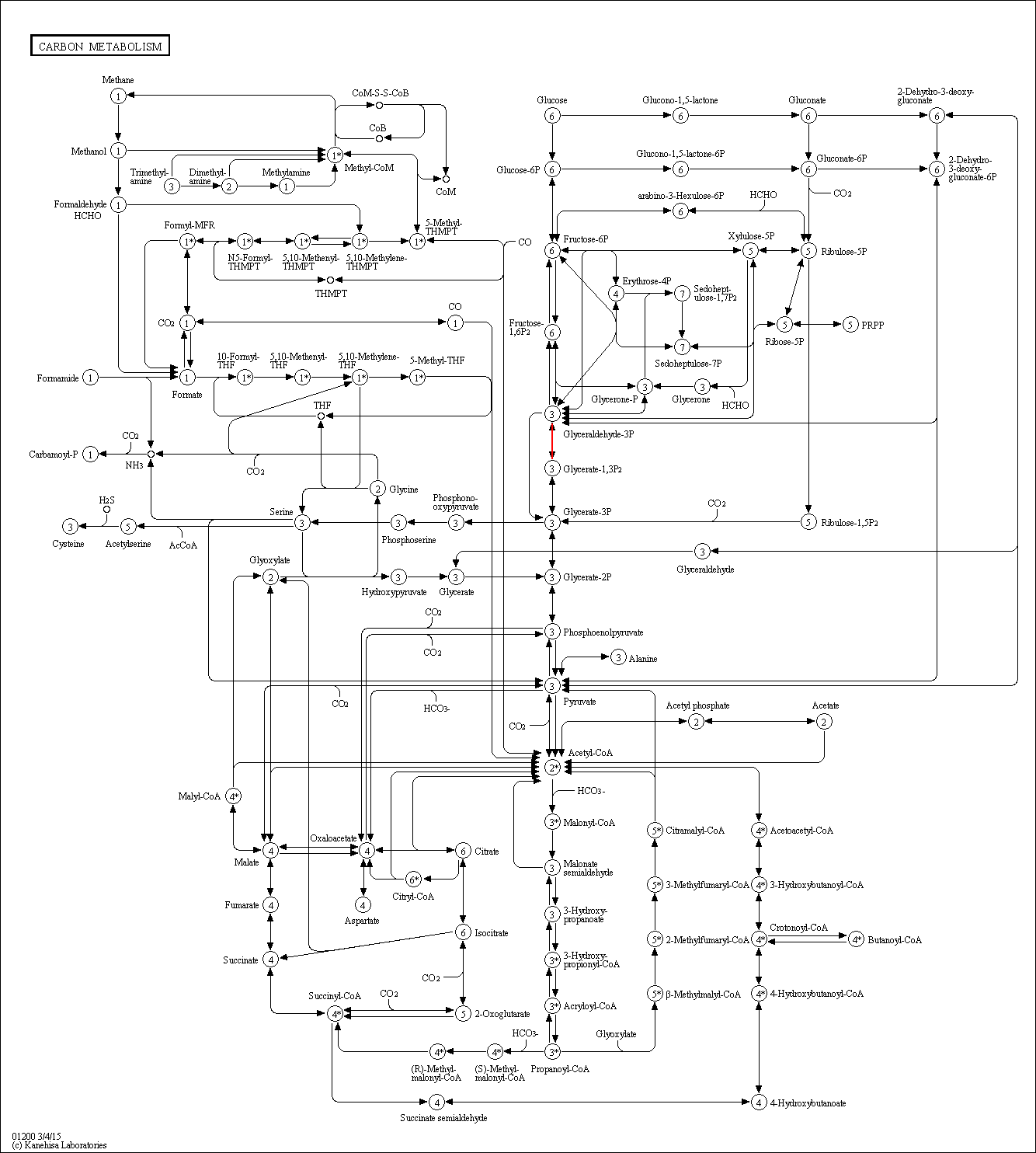

Supplement: S6 Fig — The red box indicates the genes in the associated region, while the blue box indicates all the enzymes needed for this pathway, indicating that the corresponding genes are related to this enzyme. The genes in the associated regions associated with this pathway are highlighted in red. (TIF) [file pone.0261403.s006.tif]

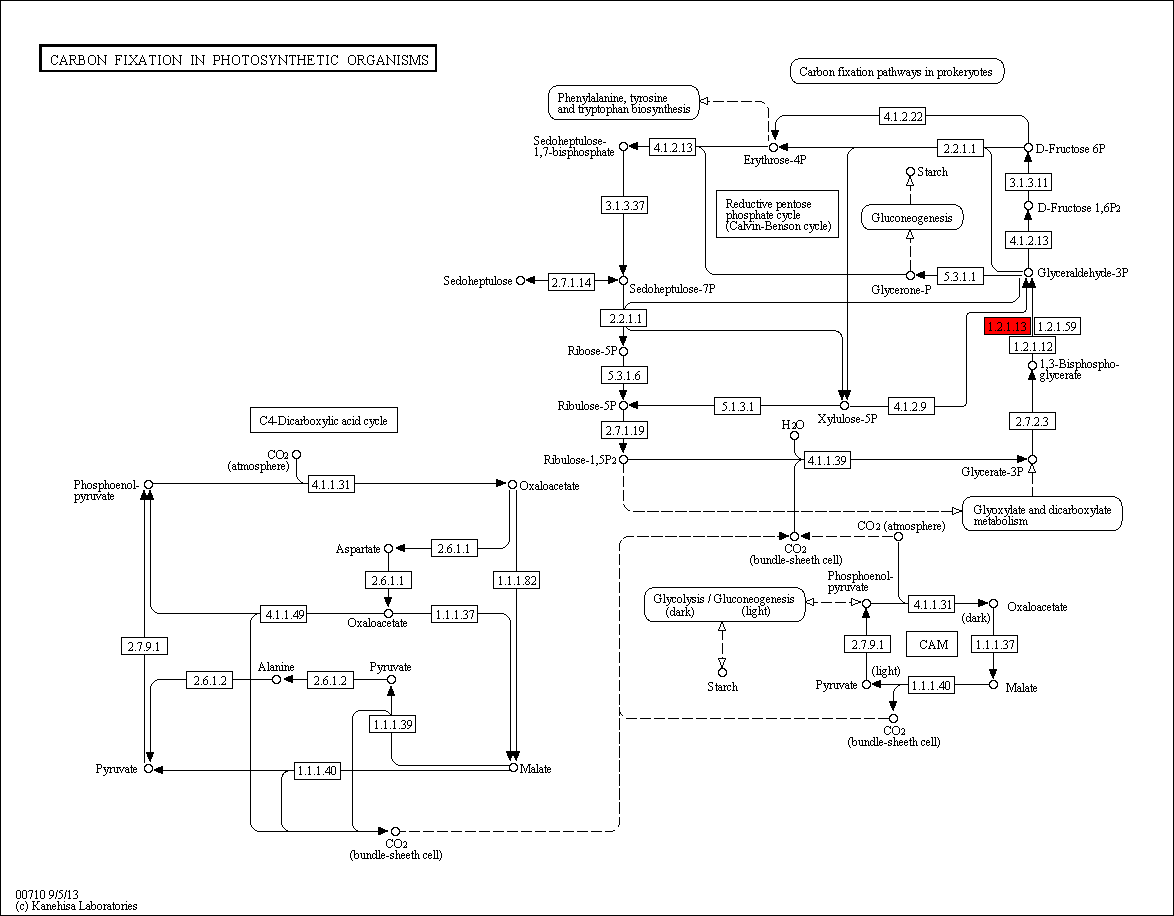

Supplement: S7 Fig — The red box indicates the genes in the associated region, while the blue box indicates all the enzymes needed for this pathway, indicating that the corresponding genes are related to this enzyme. The genes in the associated regions associated with this pathway are highlighted in red. (TIF) [file pone.0261403.s007.tif]

COG Function Classification of Consensus Sequence

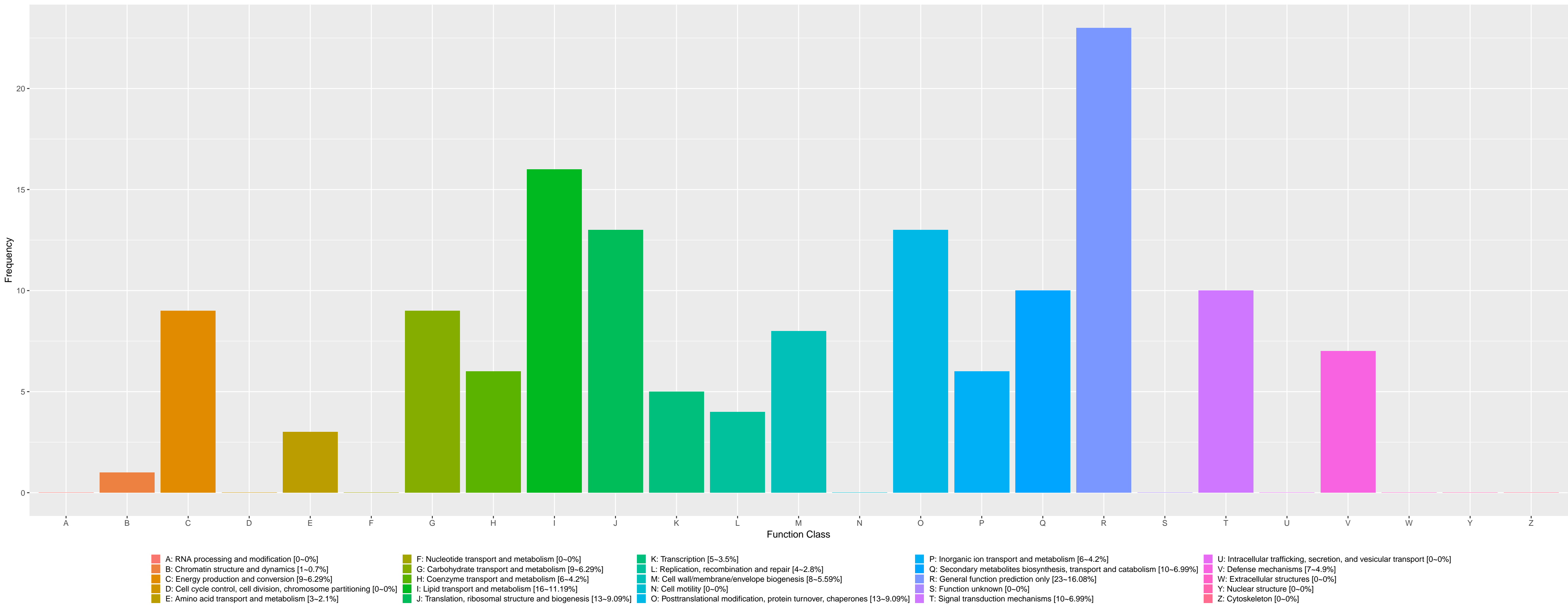

Supplement: S1 File — (ZIP) [file pone.0261403.s011.zip › ED/Anno/GeneAnno/Cog_Anno/Solanum_tuberosum_v4.03.Cog.classfy.png.pdf]

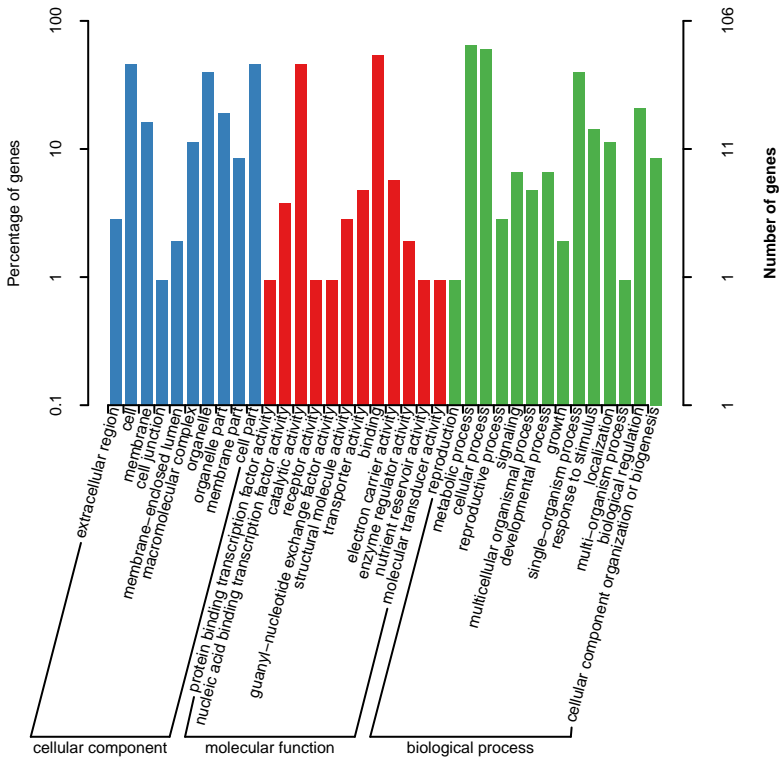

Supplement: S1 File — (ZIP) [file pone.0261403.s011.zip › ED/Anno/GeneAnno/GO_Anno/go_enrichment/Solanum_tuberosum_v4.03.GO.pdf]

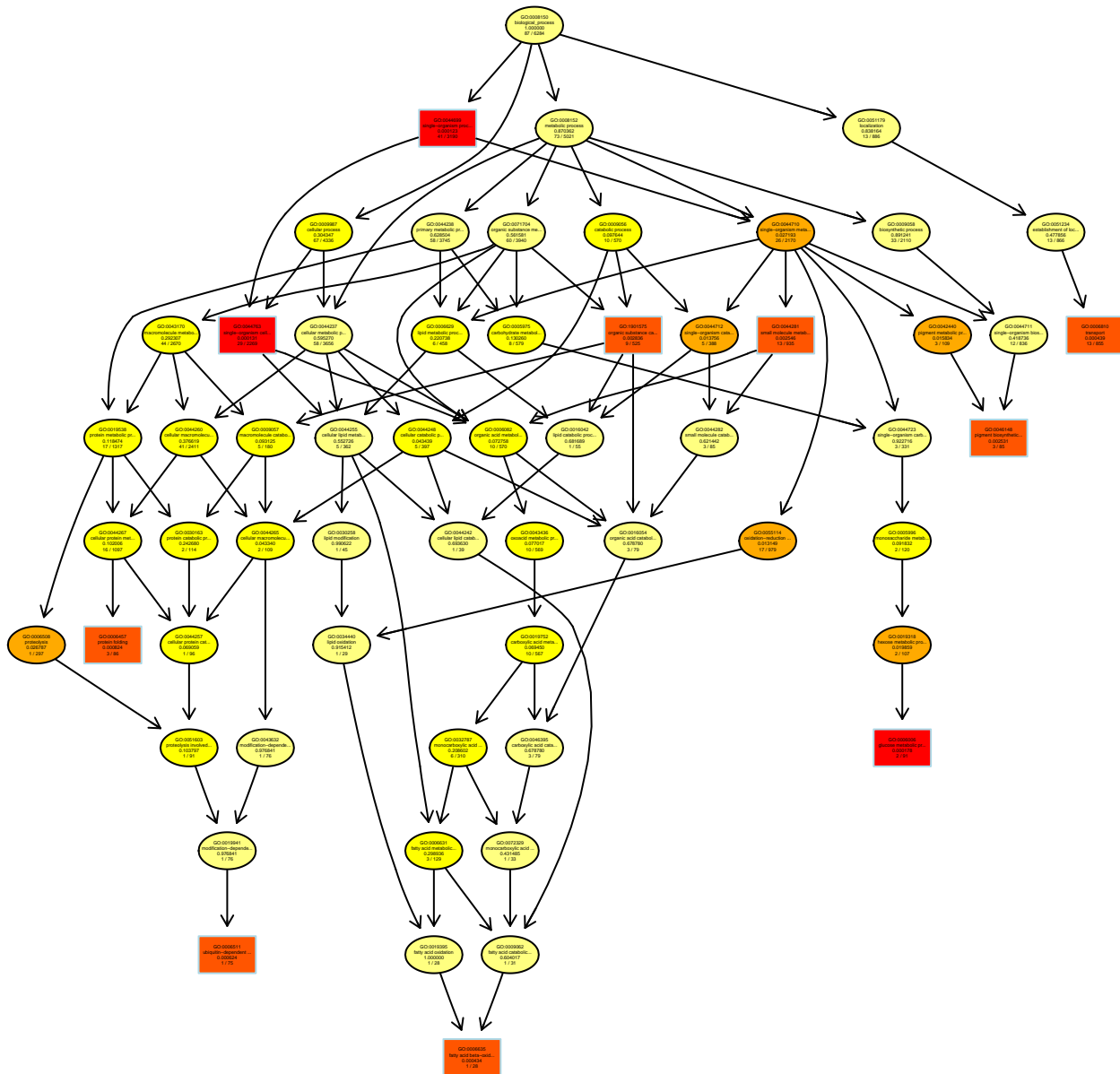

Supplement: S1 File — (ZIP) [file pone.0261403.s011.zip › ED/Anno/GeneAnno/GO_Anno/topGO/Solanum_tuberosum_v4.03.topGO_BP.pdf]

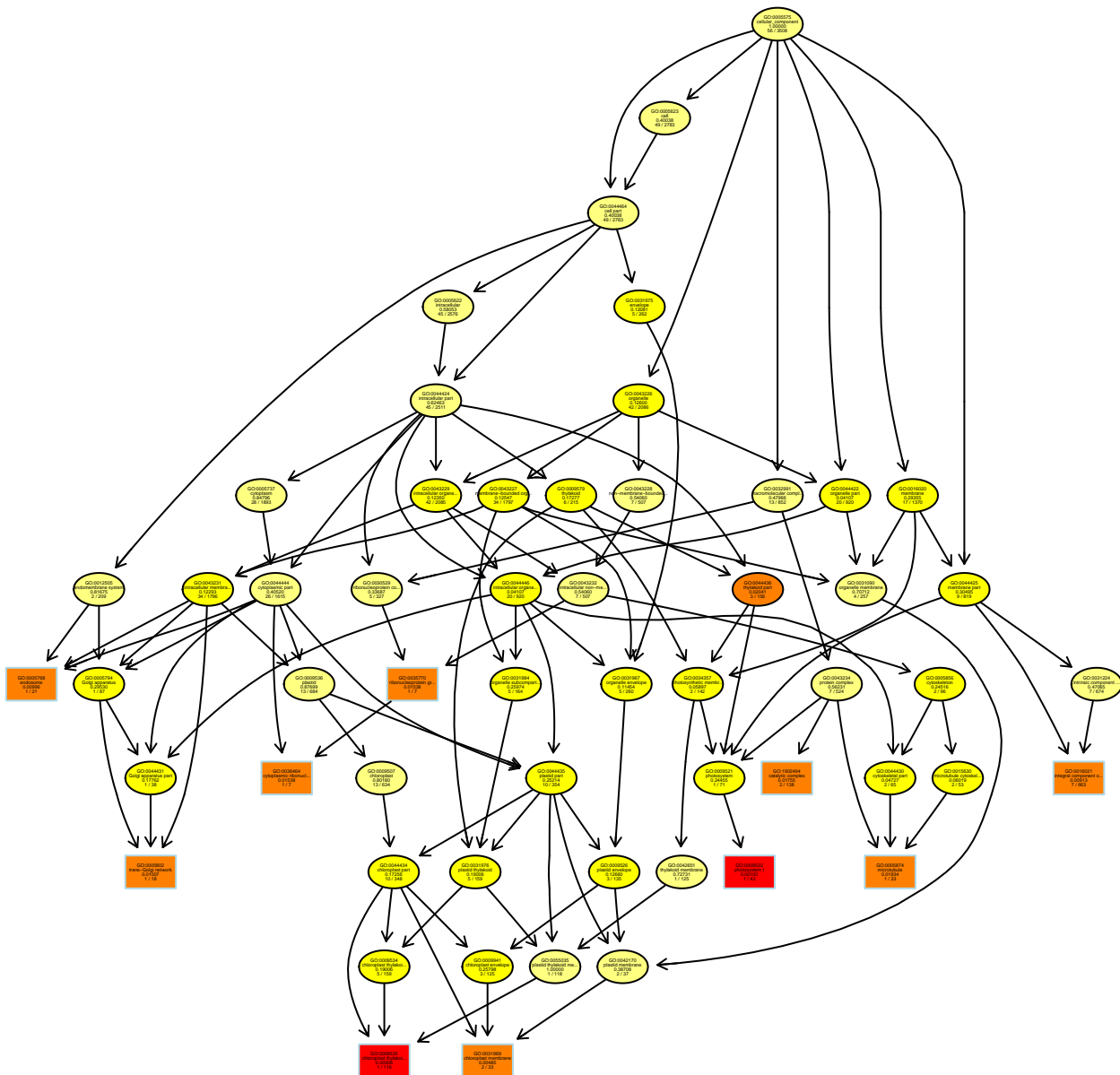

Supplement: S1 File — (ZIP) [file pone.0261403.s011.zip › ED/Anno/GeneAnno/GO_Anno/topGO/Solanum_tuberosum_v4.03.topGO_CC.pdf]

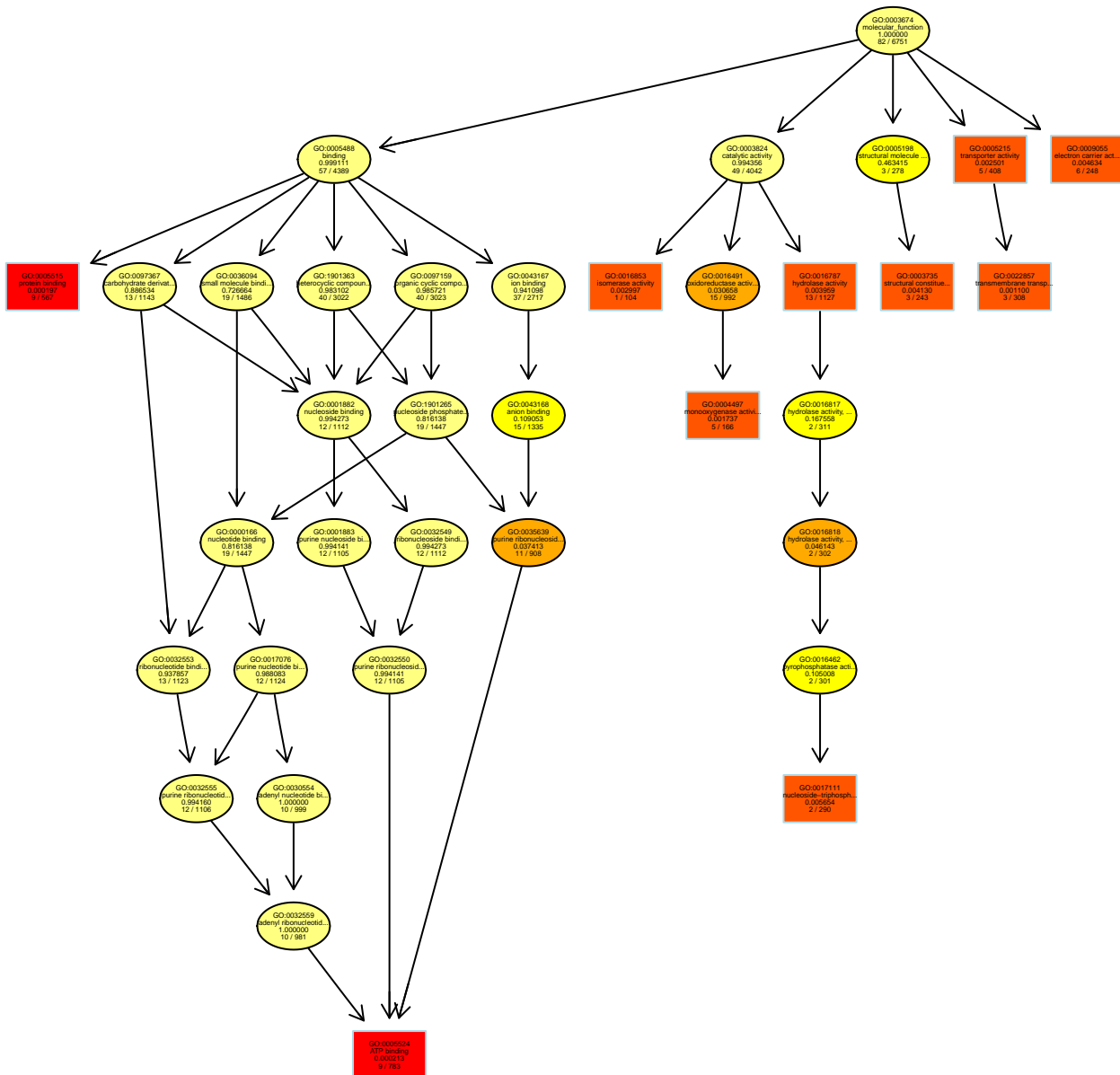

Supplement: S1 File — (ZIP) [file pone.0261403.s011.zip › ED/Anno/GeneAnno/GO_Anno/topGO/Solanum_tuberosum_v4.03.topGO_MF.pdf]

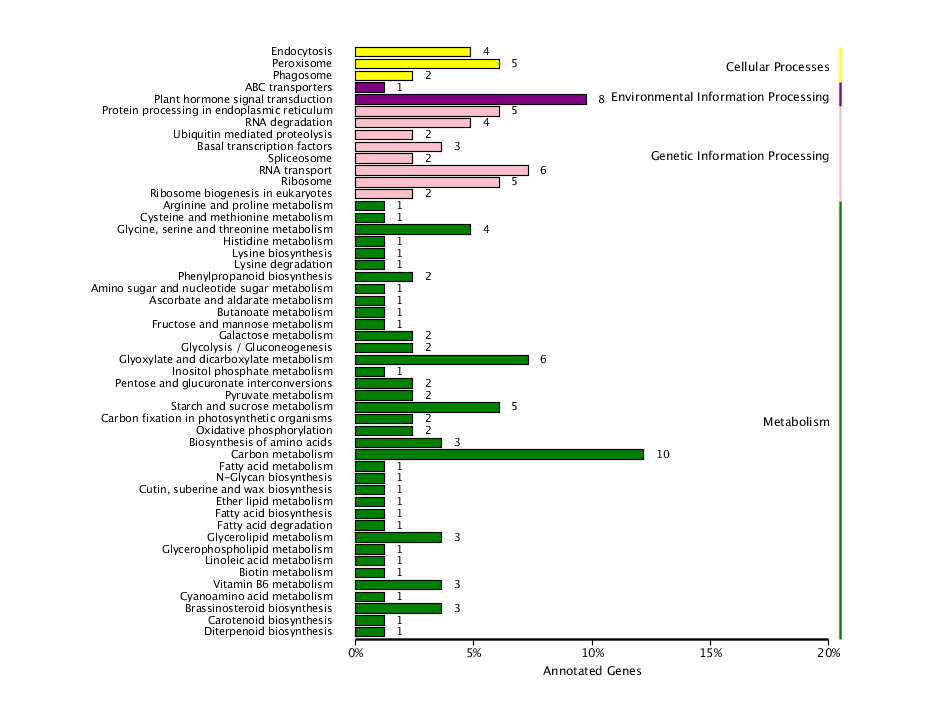

Supplement: S1 File — (ZIP) [file pone.0261403.s011.zip › ED/Anno/GeneAnno/pathway/kegg_enrichment/Solanum_tuberosum_v4.03.png]

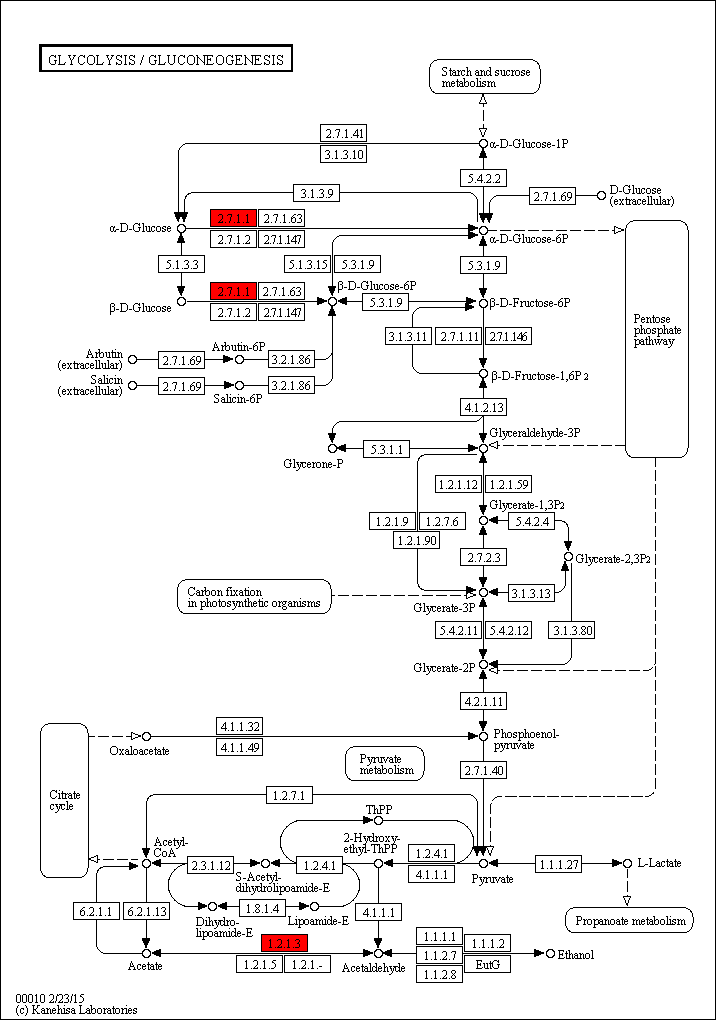

Supplement: S1 File — (ZIP) [file pone.0261403.s011.zip › ED/Anno/GeneAnno/pathway/kegg_map/ko00010.png]

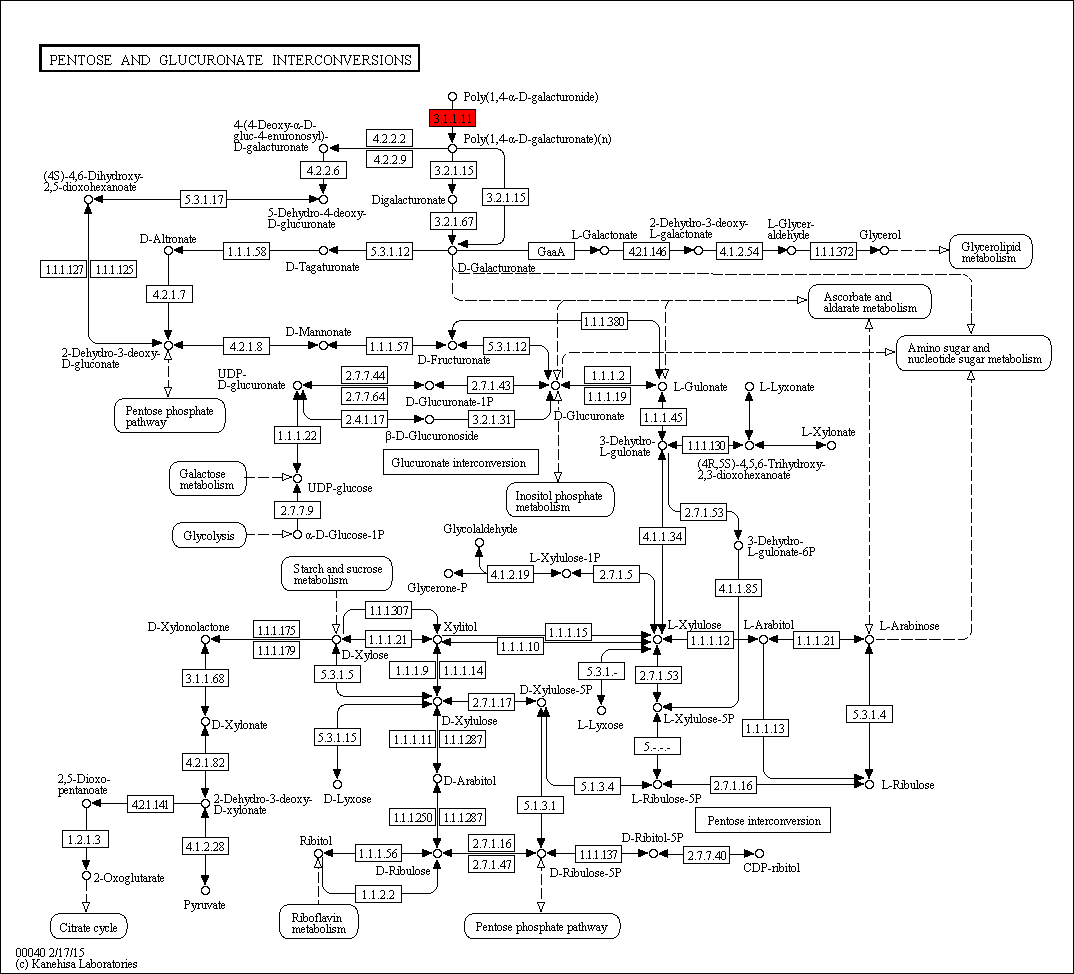

Supplement: S1 File — (ZIP) [file pone.0261403.s011.zip › ED/Anno/GeneAnno/pathway/kegg_map/ko00040.png]

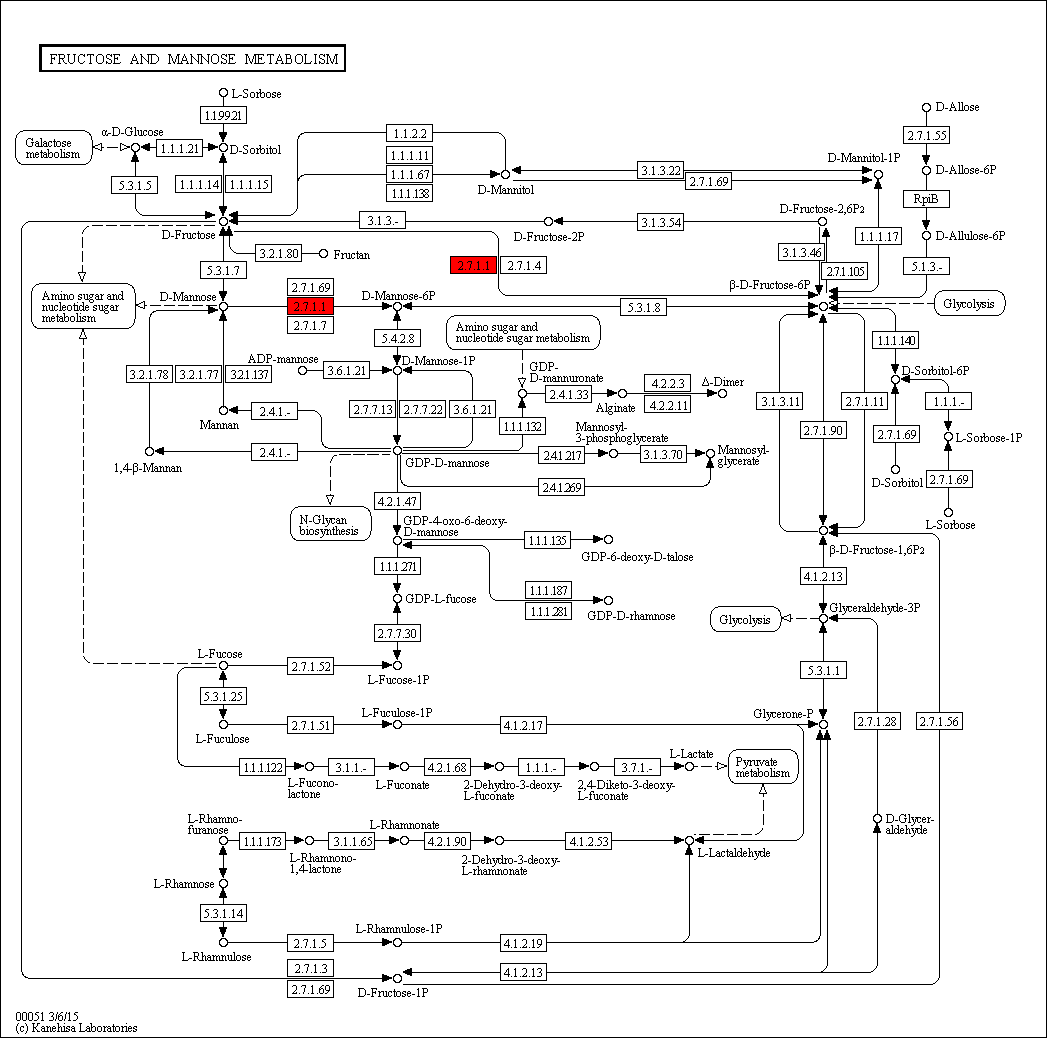

Supplement: S1 File — (ZIP) [file pone.0261403.s011.zip › ED/Anno/GeneAnno/pathway/kegg_map/ko00051.png]

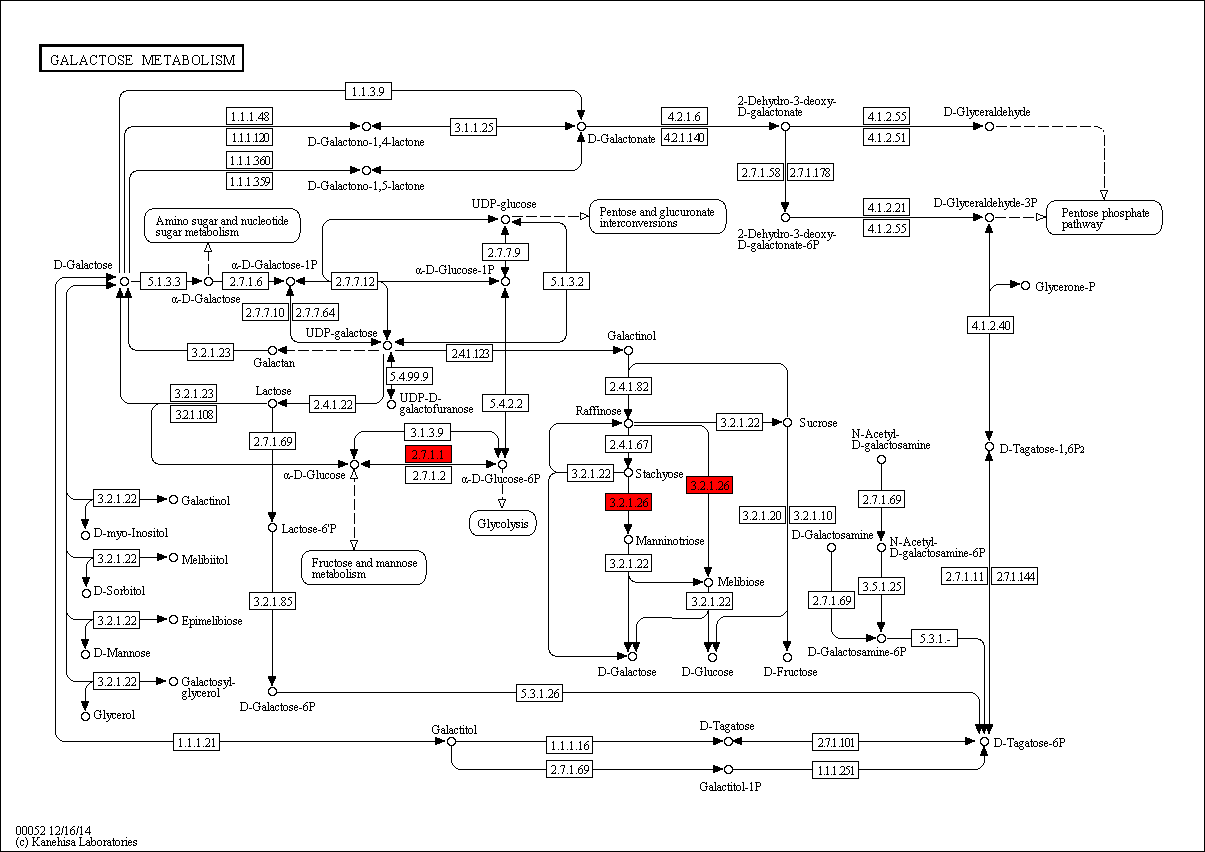

Supplement: S1 File — (ZIP) [file pone.0261403.s011.zip › ED/Anno/GeneAnno/pathway/kegg_map/ko00052.png]

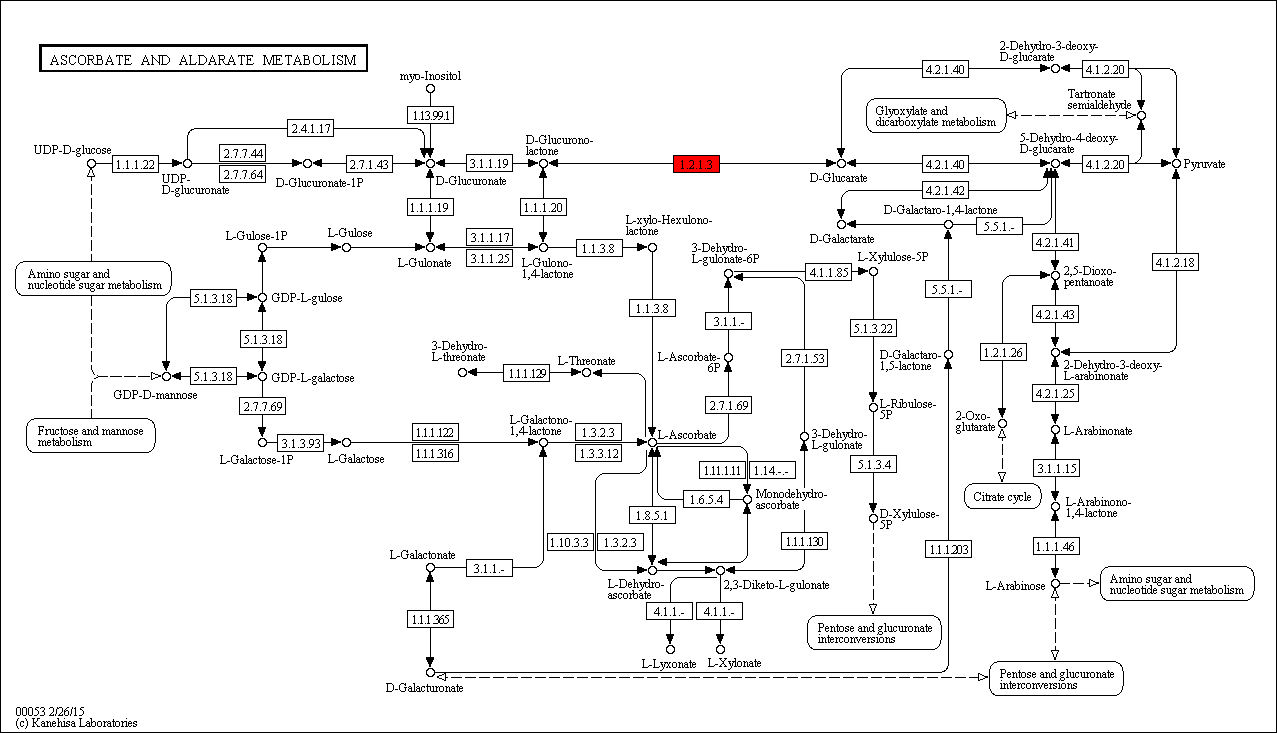

Supplement: S1 File — (ZIP) [file pone.0261403.s011.zip › ED/Anno/GeneAnno/pathway/kegg_map/ko00053.png]

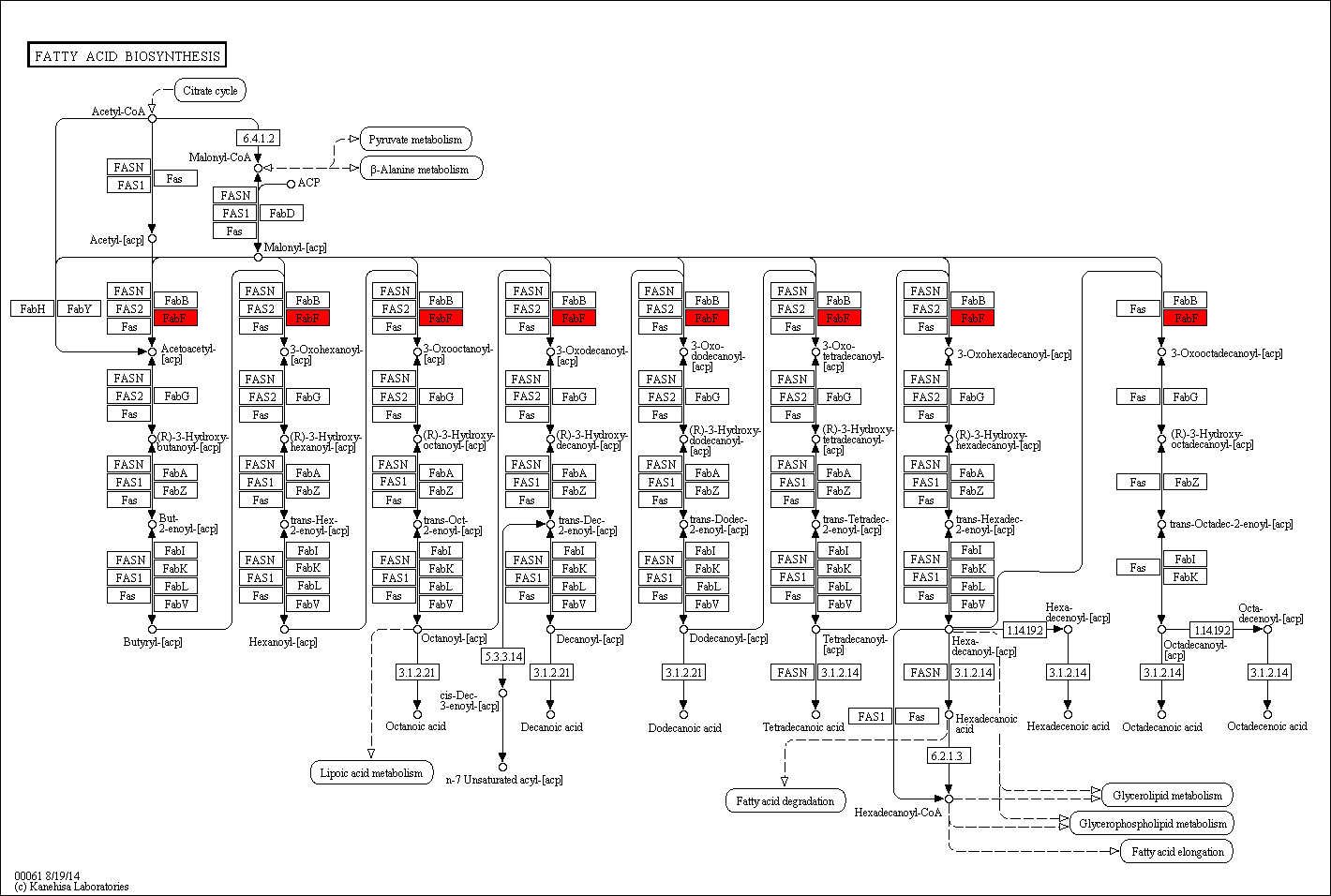

Supplement: S1 File — (ZIP) [file pone.0261403.s011.zip › ED/Anno/GeneAnno/pathway/kegg_map/ko00061.png]

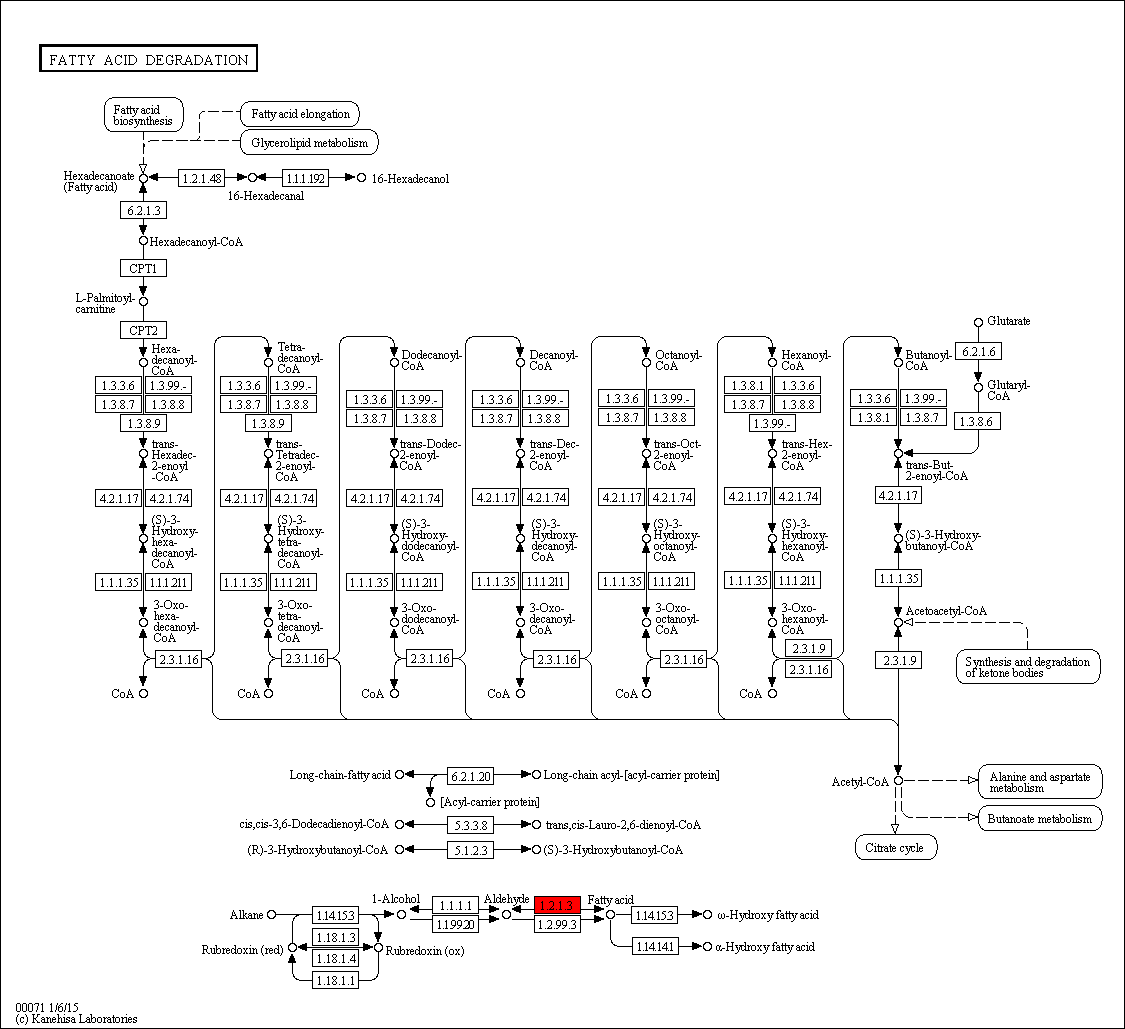

Supplement: S1 File — (ZIP) [file pone.0261403.s011.zip › ED/Anno/GeneAnno/pathway/kegg_map/ko00071.png]

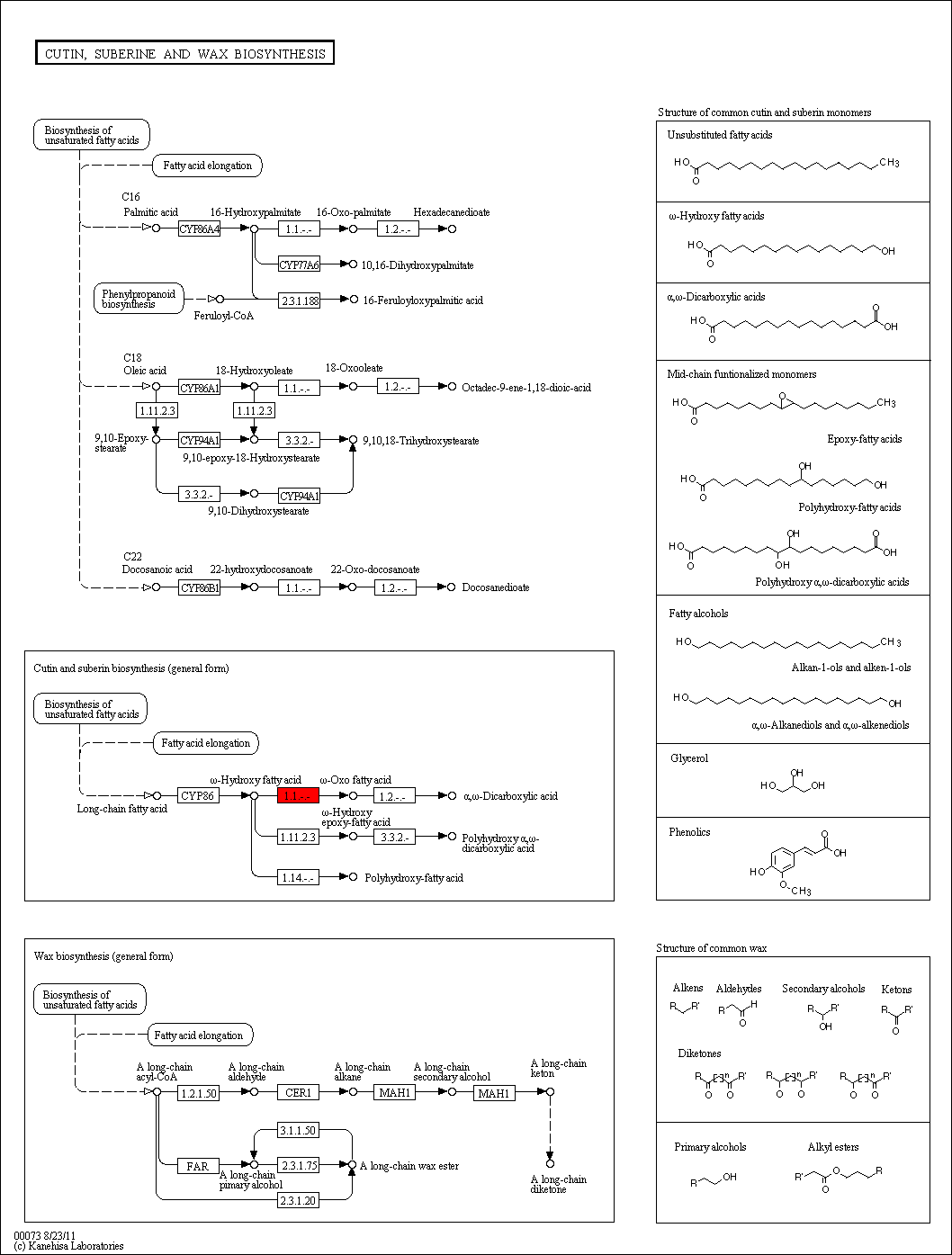

Supplement: S1 File — (ZIP) [file pone.0261403.s011.zip › ED/Anno/GeneAnno/pathway/kegg_map/ko00073.png]

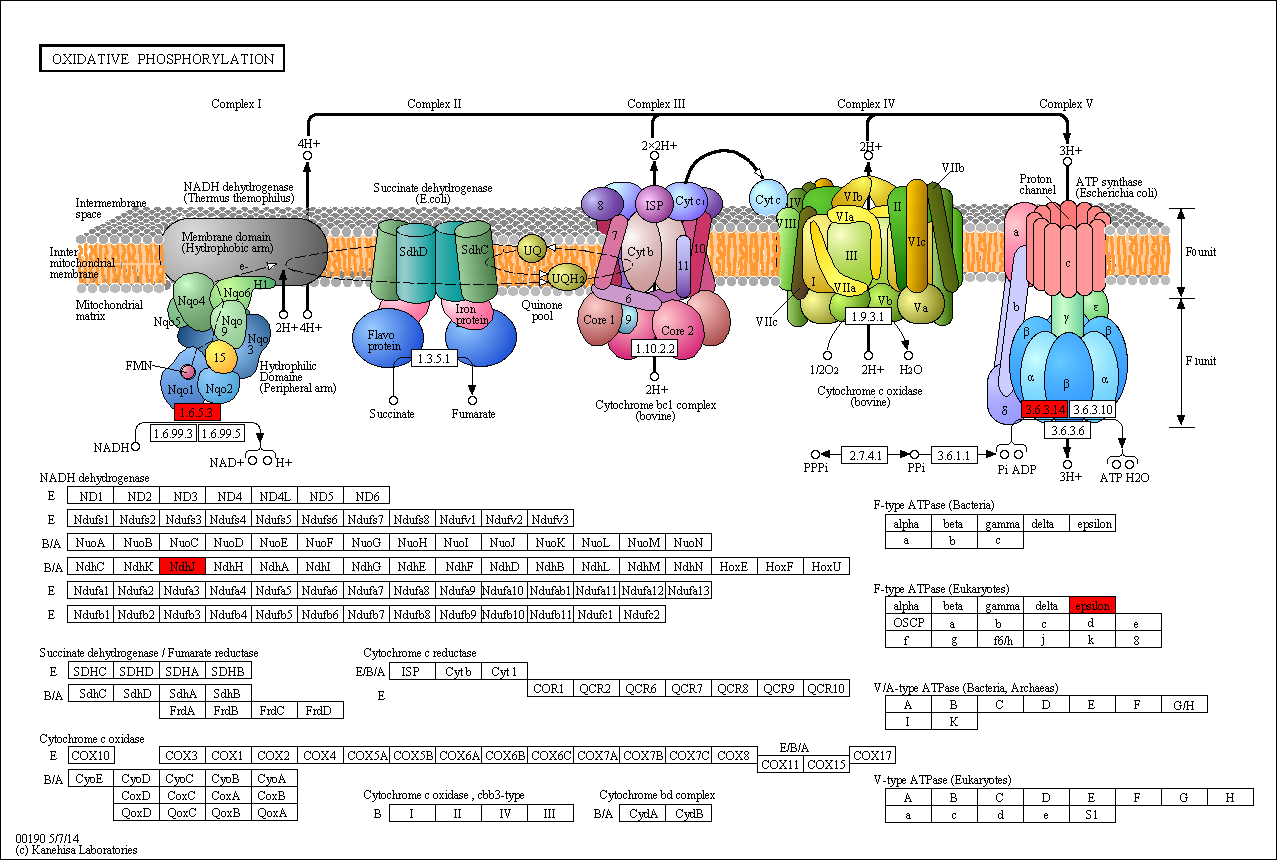

Supplement: S1 File — (ZIP) [file pone.0261403.s011.zip › ED/Anno/GeneAnno/pathway/kegg_map/ko00190.png]

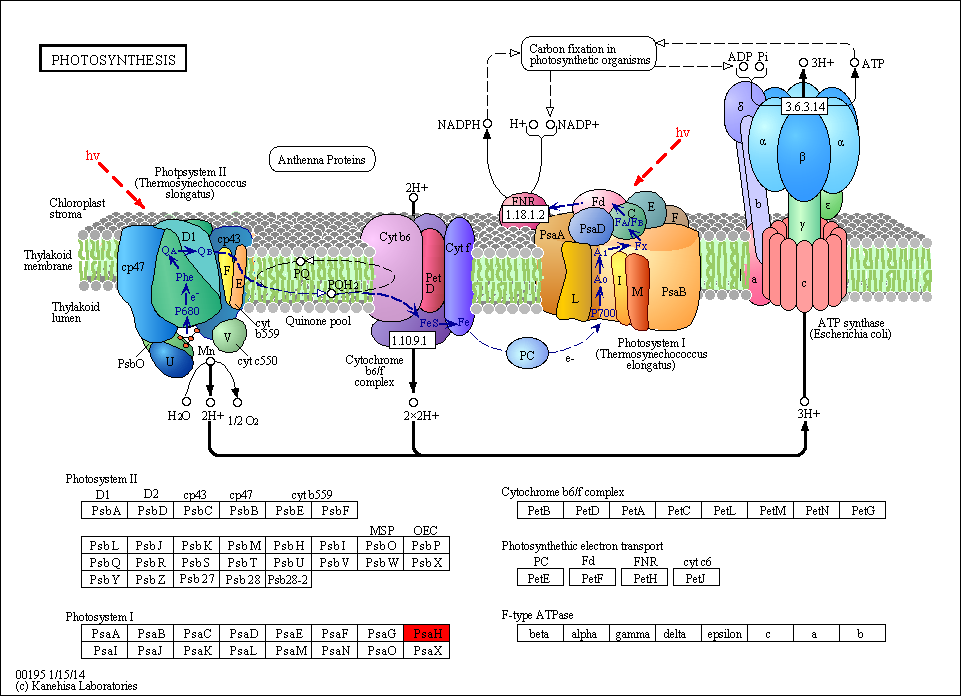

Supplement: S1 File — (ZIP) [file pone.0261403.s011.zip › ED/Anno/GeneAnno/pathway/kegg_map/ko00195.png]

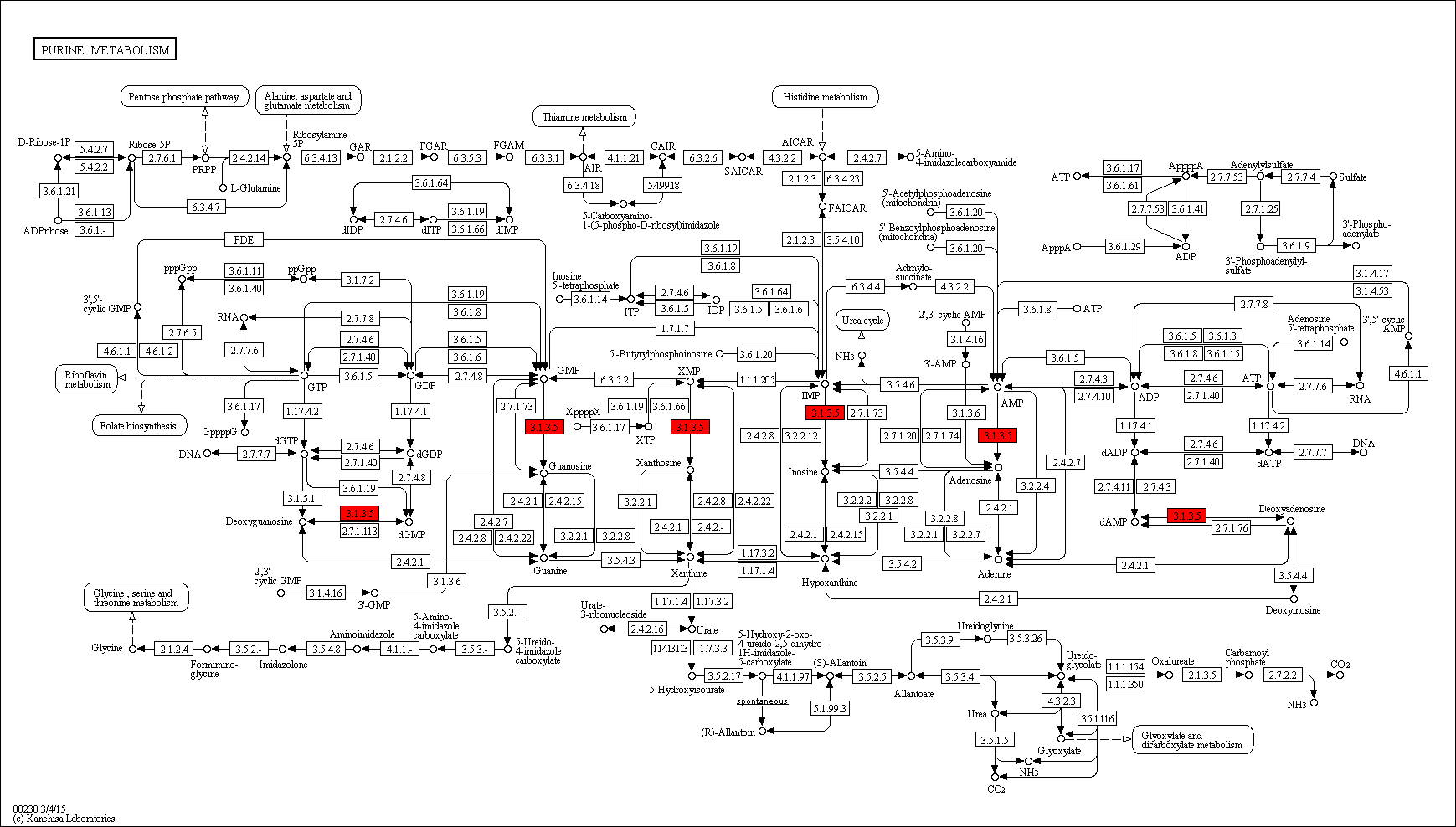

Supplement: S1 File — (ZIP) [file pone.0261403.s011.zip › ED/Anno/GeneAnno/pathway/kegg_map/ko00230.png]

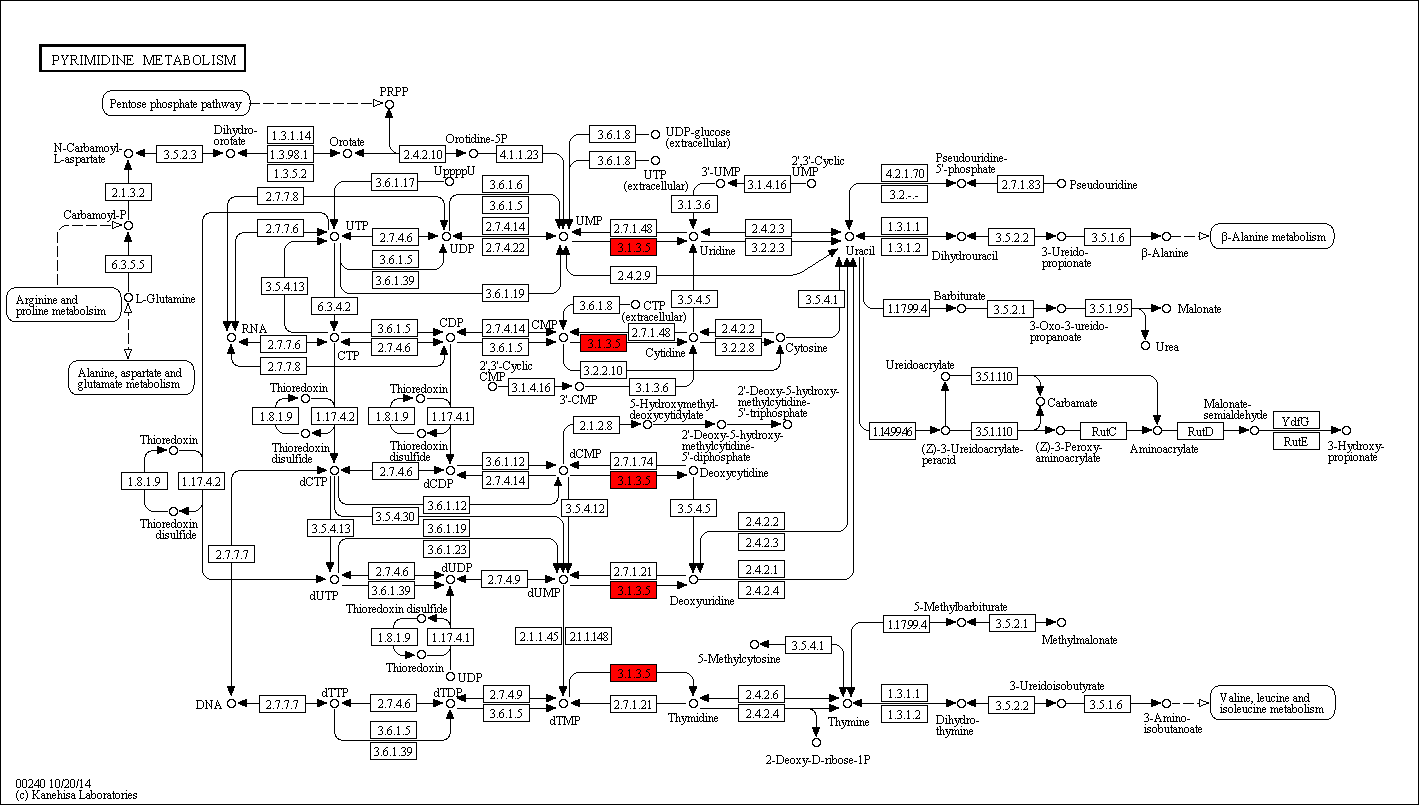

Supplement: S1 File — (ZIP) [file pone.0261403.s011.zip › ED/Anno/GeneAnno/pathway/kegg_map/ko00240.png]

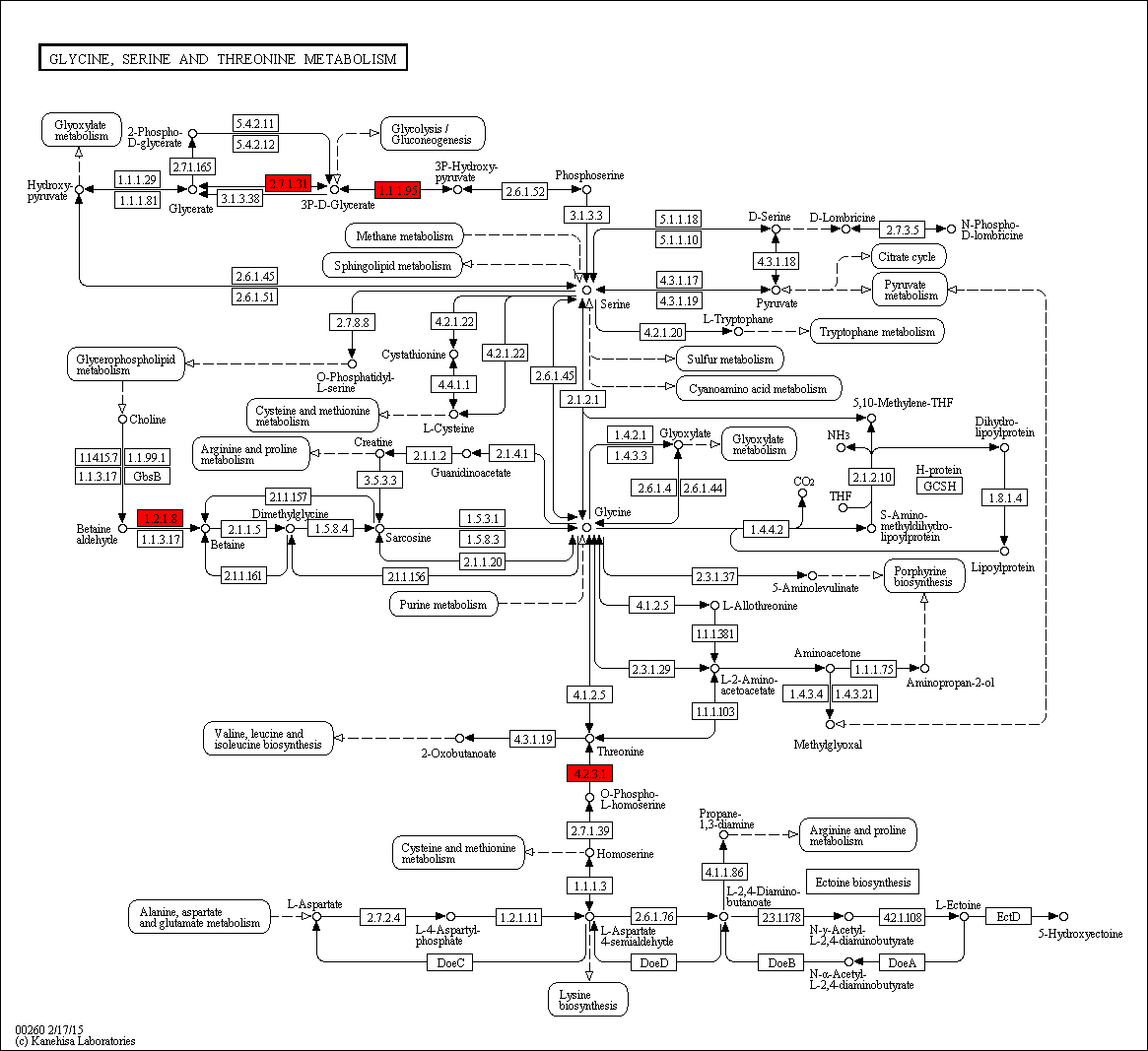

Supplement: S1 File — (ZIP) [file pone.0261403.s011.zip › ED/Anno/GeneAnno/pathway/kegg_map/ko00260.png]

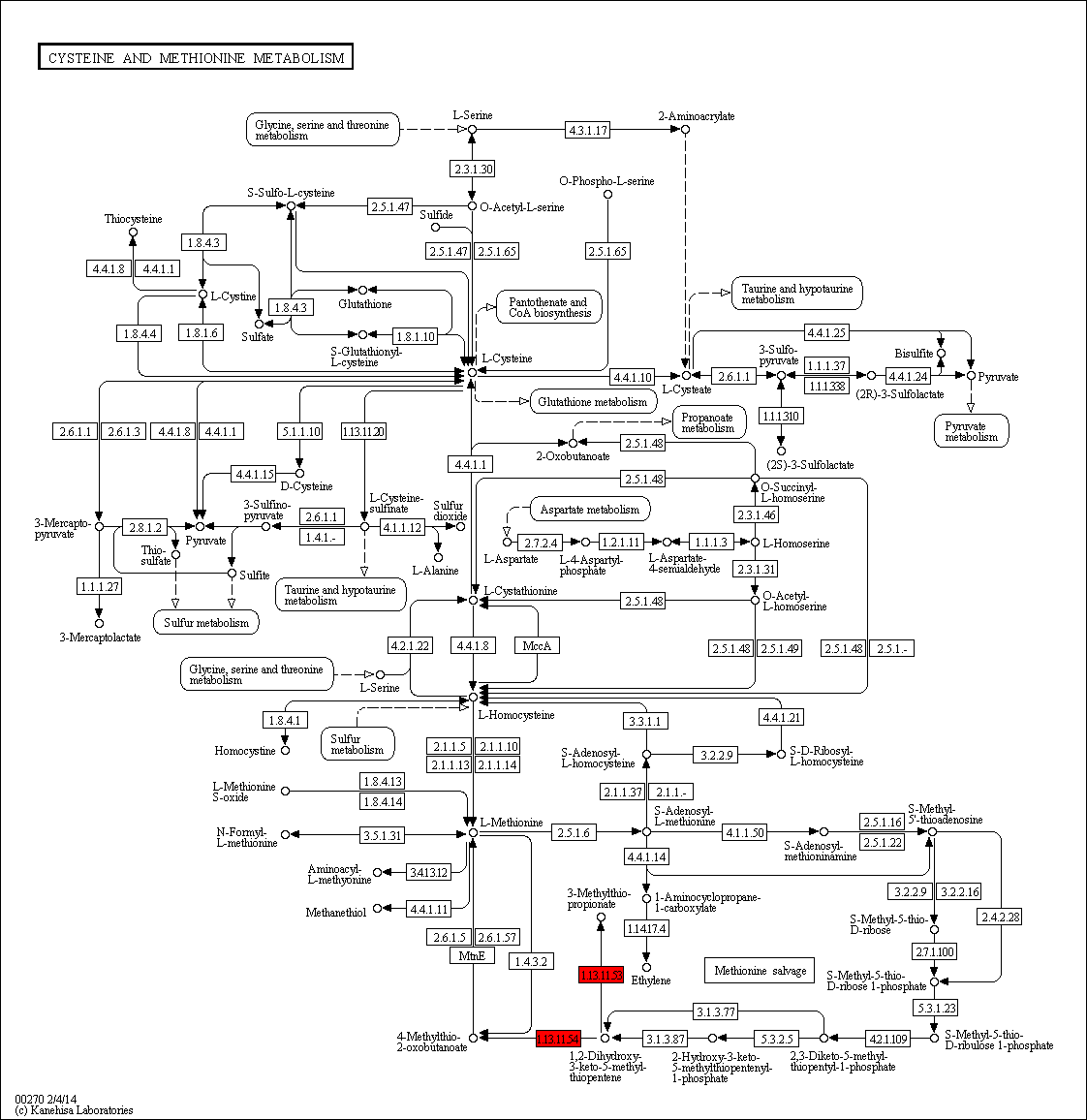

Supplement: S1 File — (ZIP) [file pone.0261403.s011.zip › ED/Anno/GeneAnno/pathway/kegg_map/ko00270.png]

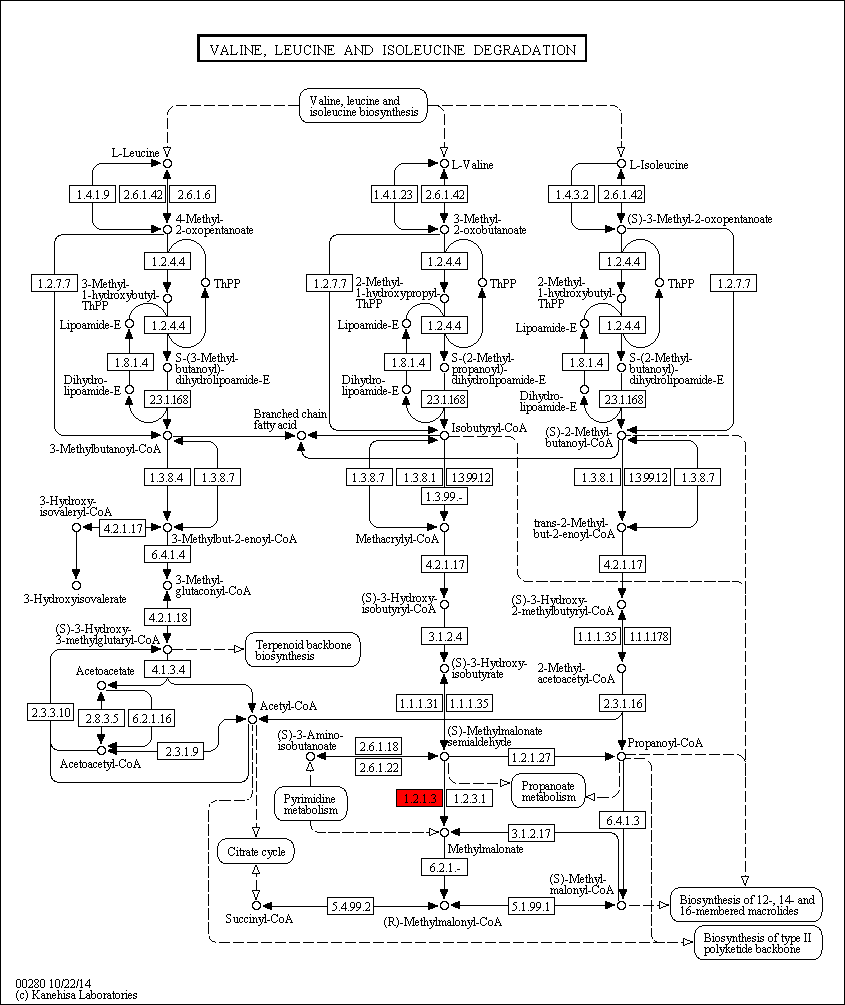

Supplement: S1 File — (ZIP) [file pone.0261403.s011.zip › ED/Anno/GeneAnno/pathway/kegg_map/ko00280.png]

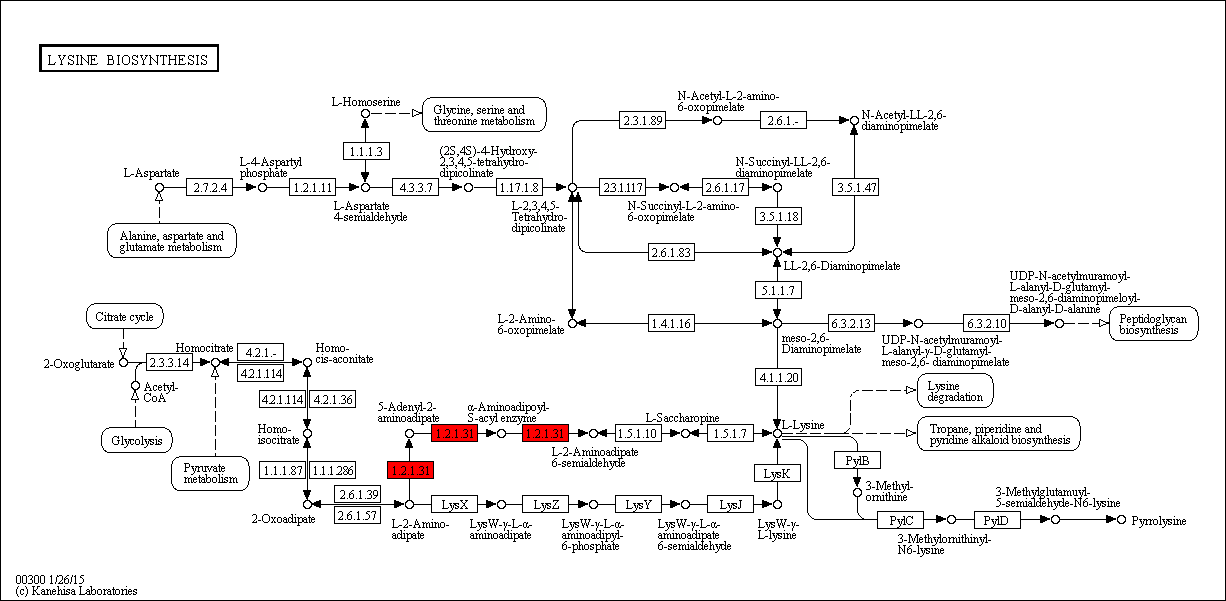

Supplement: S1 File — (ZIP) [file pone.0261403.s011.zip › ED/Anno/GeneAnno/pathway/kegg_map/ko00300.png]

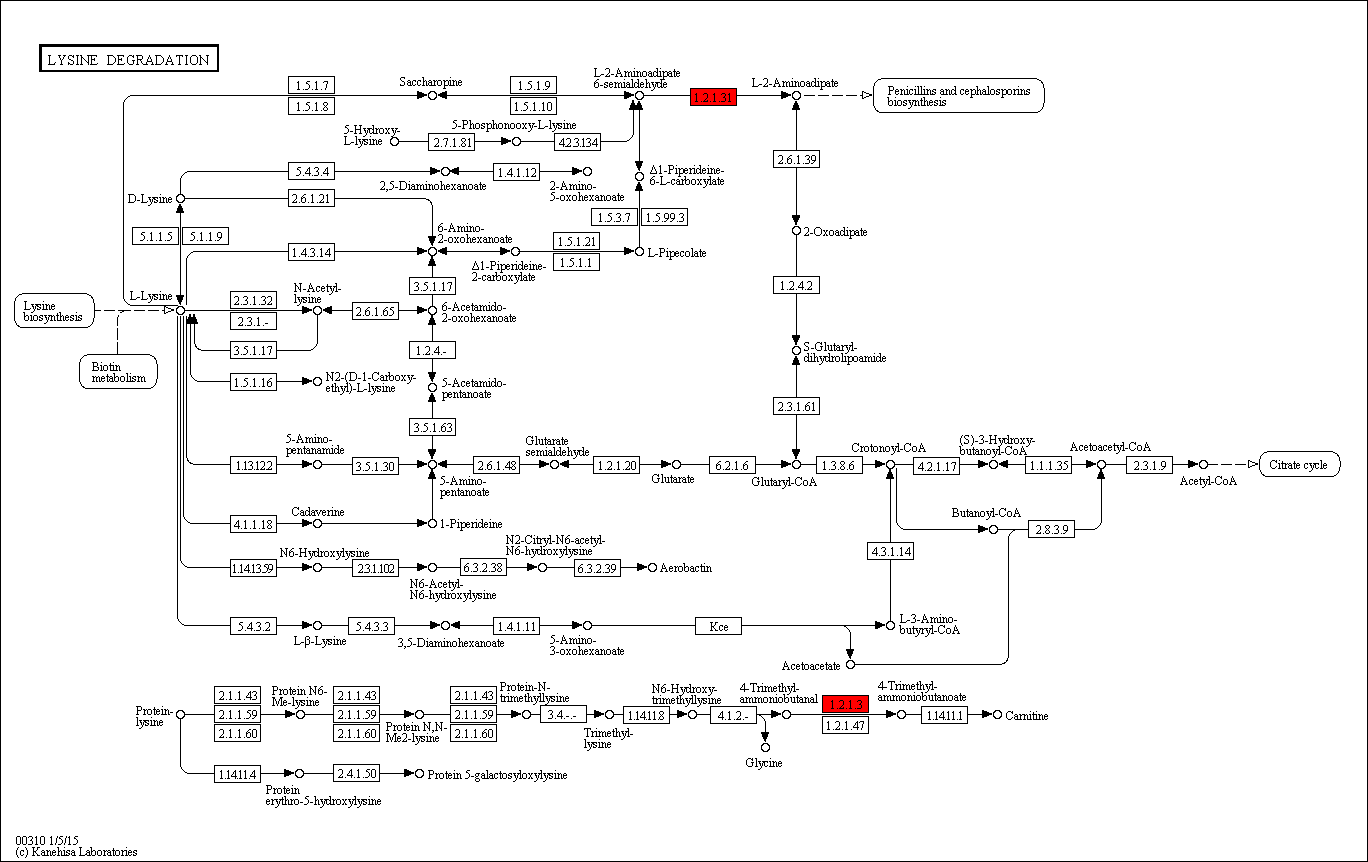

Supplement: S1 File — (ZIP) [file pone.0261403.s011.zip › ED/Anno/GeneAnno/pathway/kegg_map/ko00310.png]

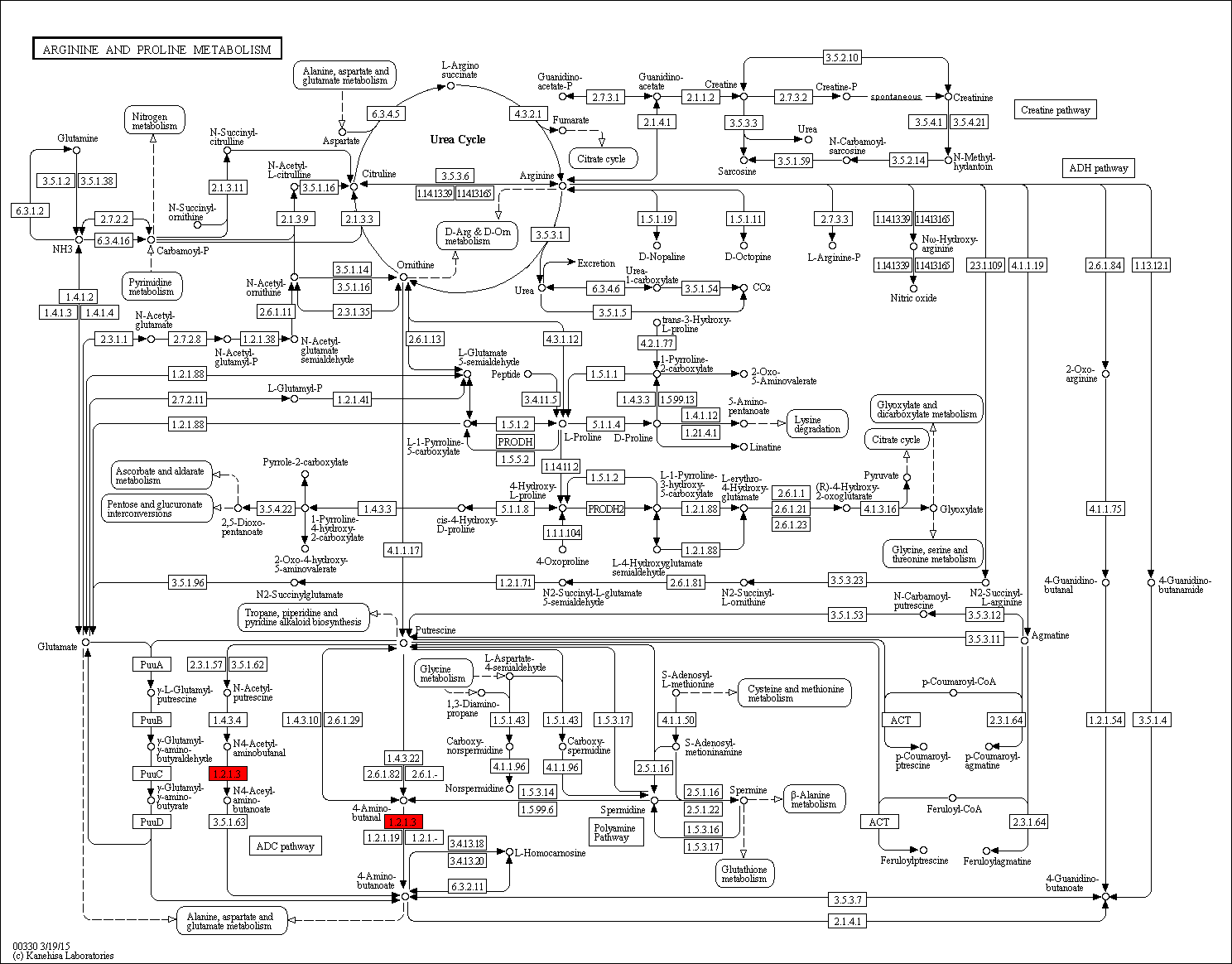

Supplement: S1 File — (ZIP) [file pone.0261403.s011.zip › ED/Anno/GeneAnno/pathway/kegg_map/ko00330.png]

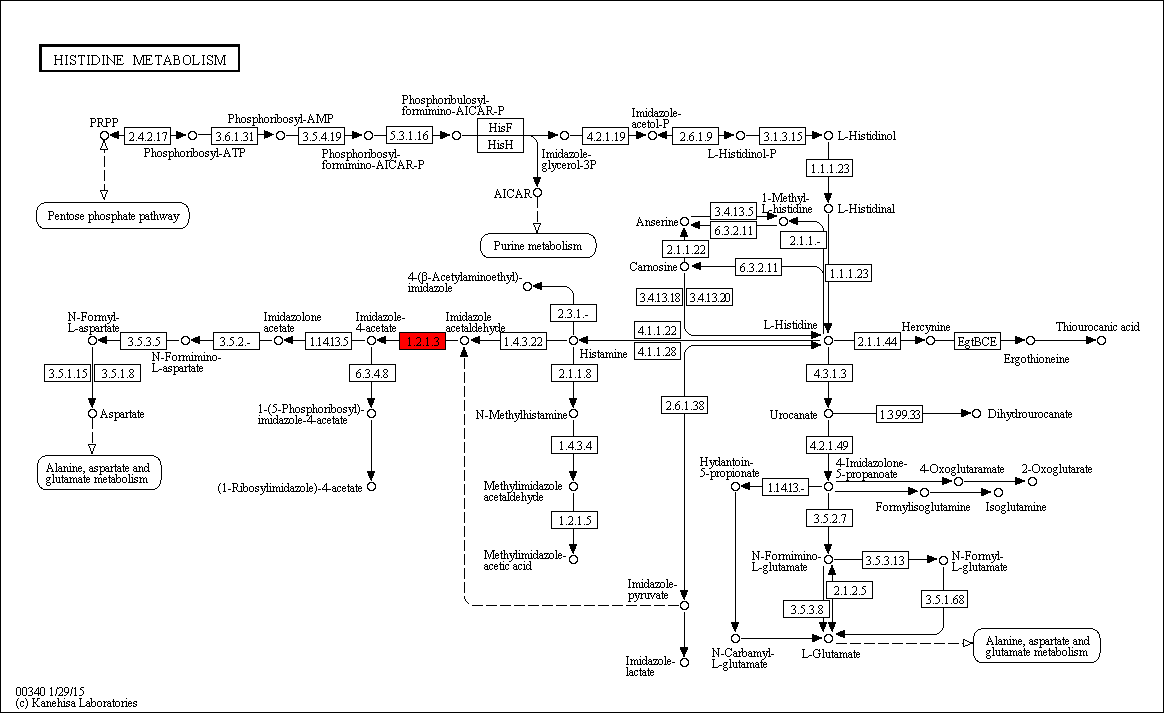

Supplement: S1 File — (ZIP) [file pone.0261403.s011.zip › ED/Anno/GeneAnno/pathway/kegg_map/ko00340.png]

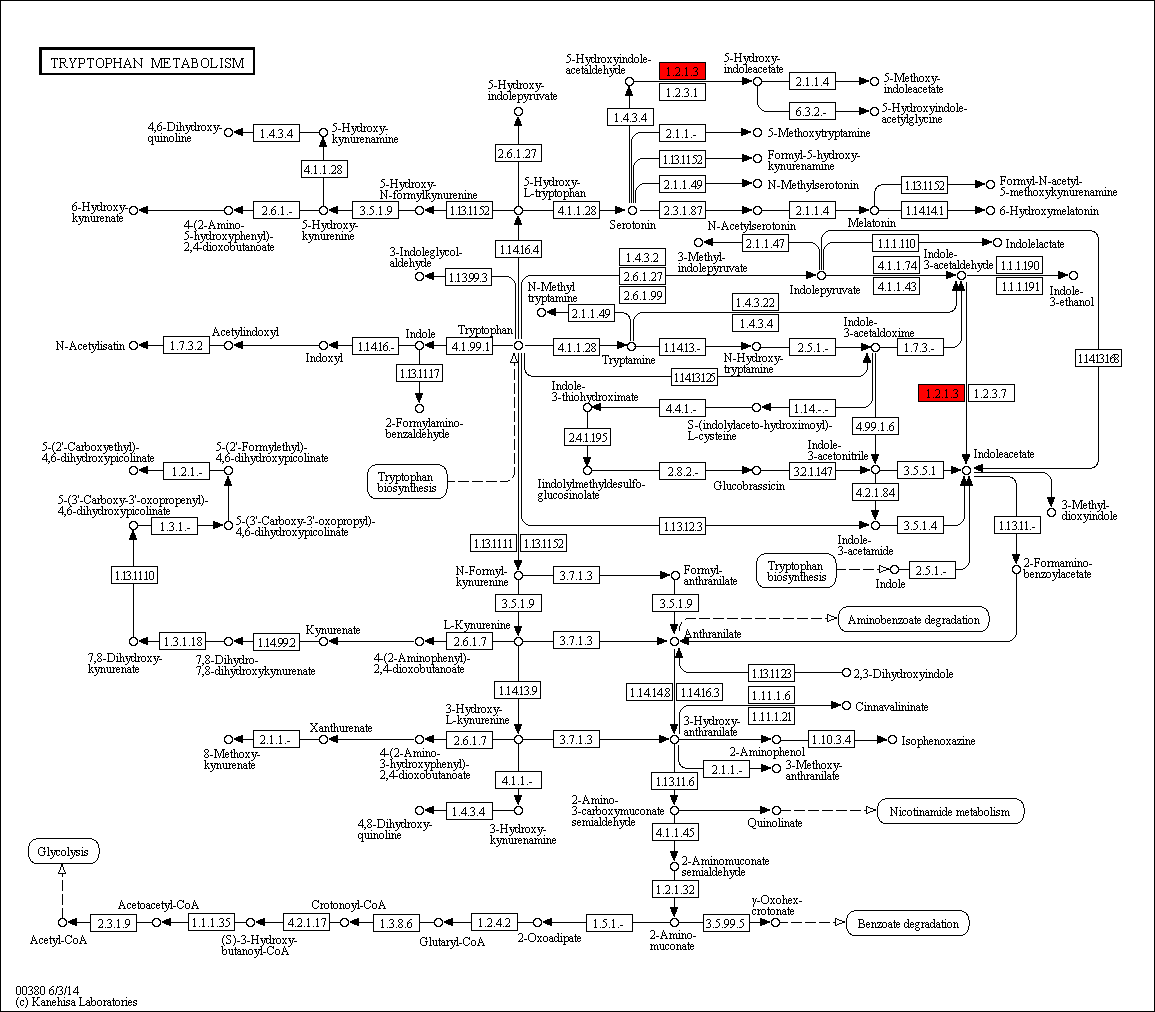

Supplement: S1 File — (ZIP) [file pone.0261403.s011.zip › ED/Anno/GeneAnno/pathway/kegg_map/ko00380.png]

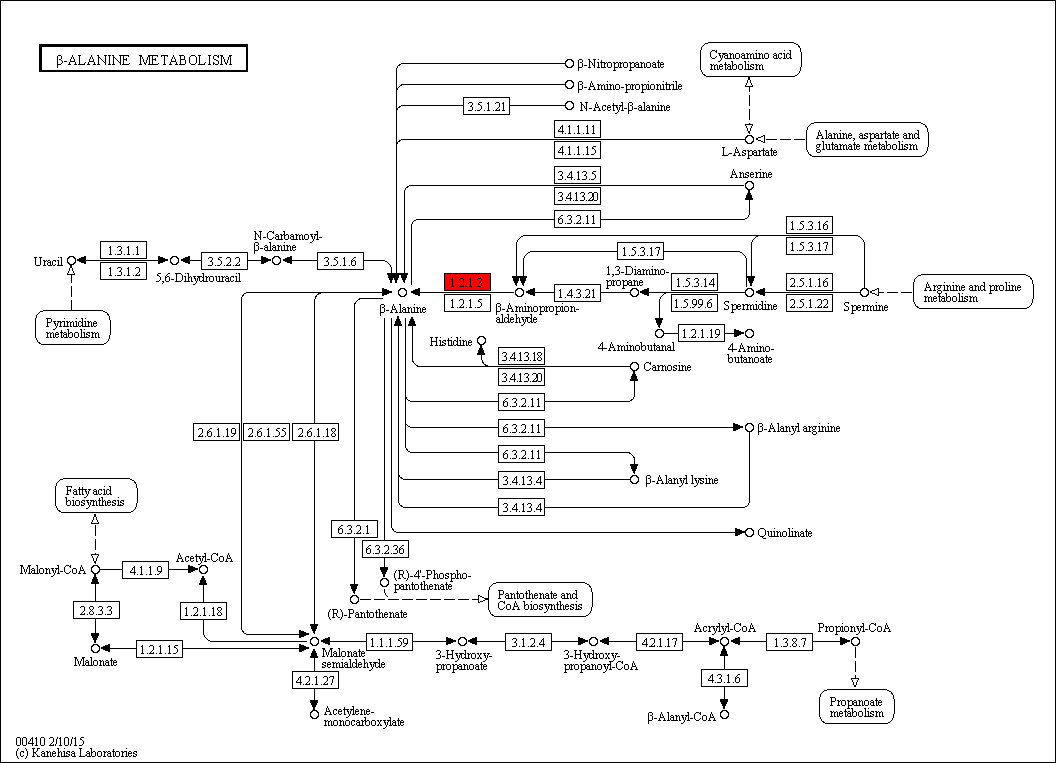

Supplement: S1 File — (ZIP) [file pone.0261403.s011.zip › ED/Anno/GeneAnno/pathway/kegg_map/ko00410.png]

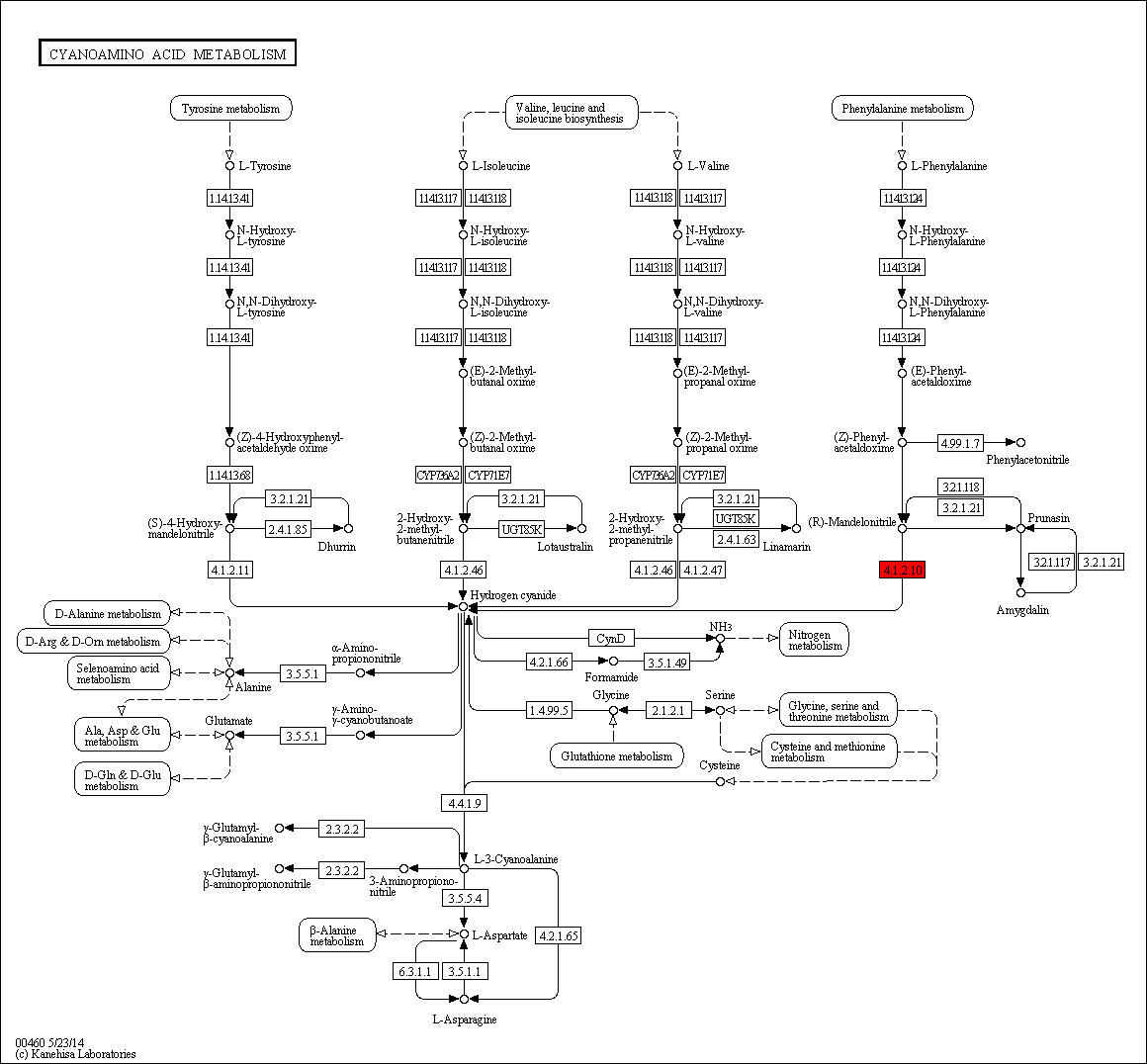

Supplement: S1 File — (ZIP) [file pone.0261403.s011.zip › ED/Anno/GeneAnno/pathway/kegg_map/ko00460.png]

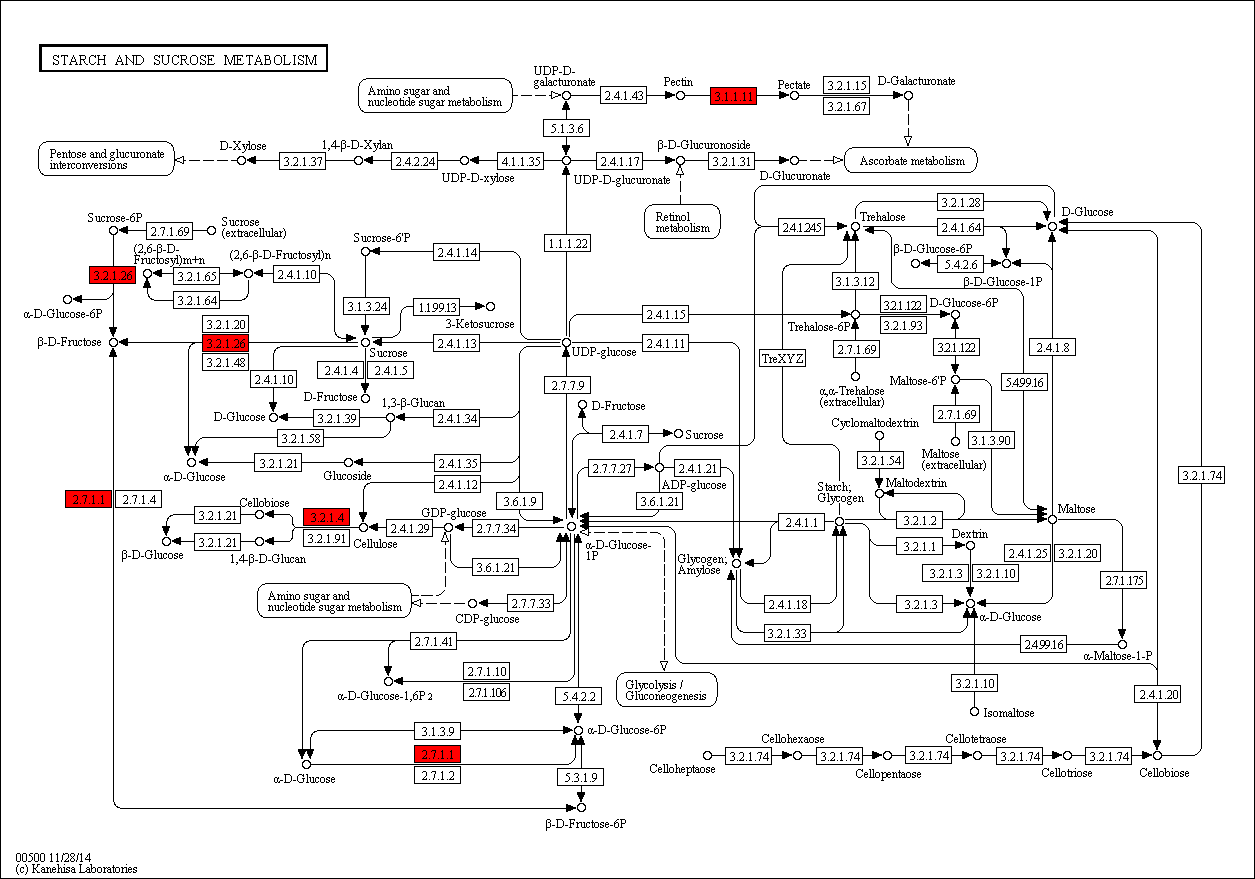

Supplement: S1 File — (ZIP) [file pone.0261403.s011.zip › ED/Anno/GeneAnno/pathway/kegg_map/ko00500.png]

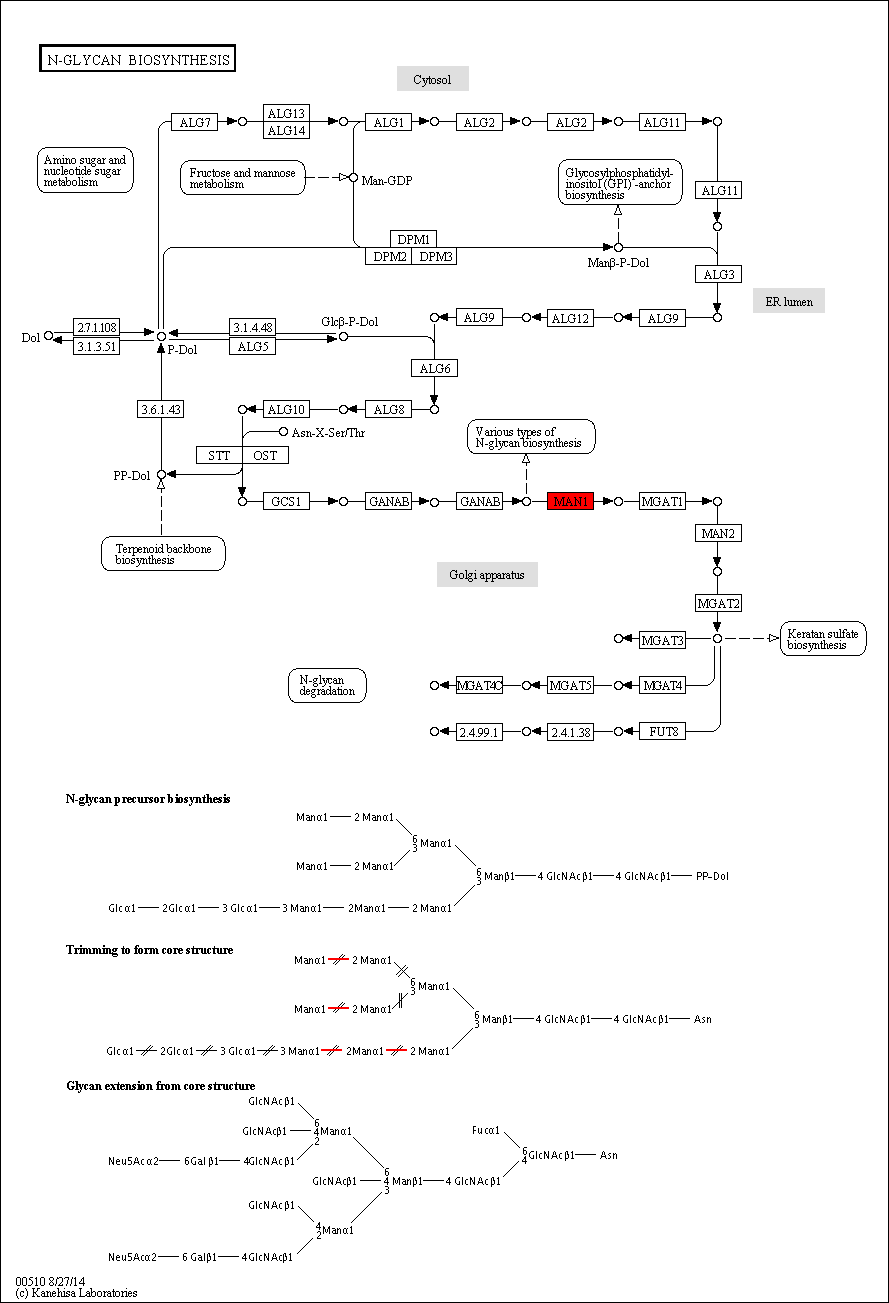

Supplement: S1 File — (ZIP) [file pone.0261403.s011.zip › ED/Anno/GeneAnno/pathway/kegg_map/ko00510.png]

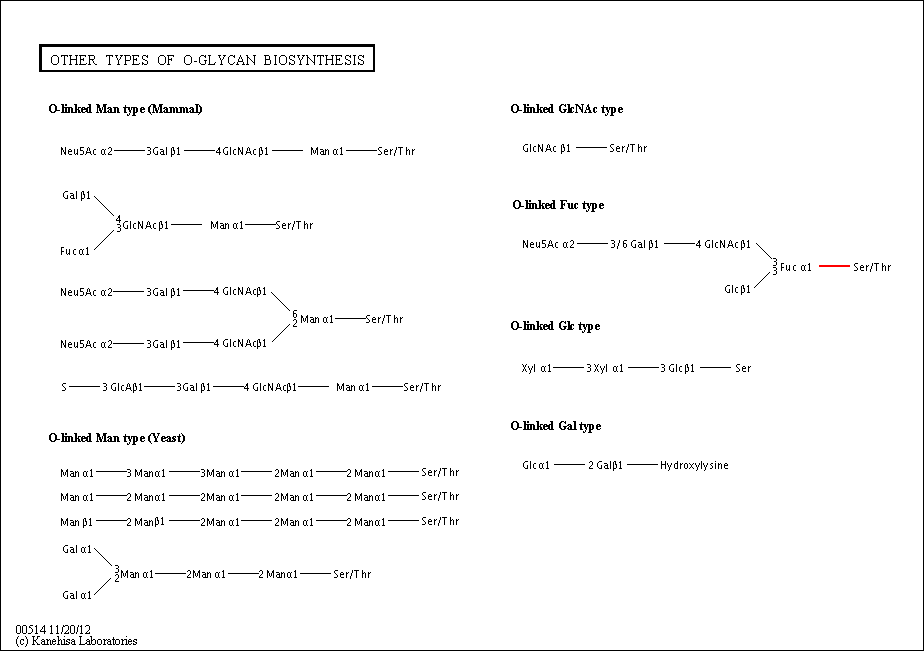

Supplement: S1 File — (ZIP) [file pone.0261403.s011.zip › ED/Anno/GeneAnno/pathway/kegg_map/ko00514.png]

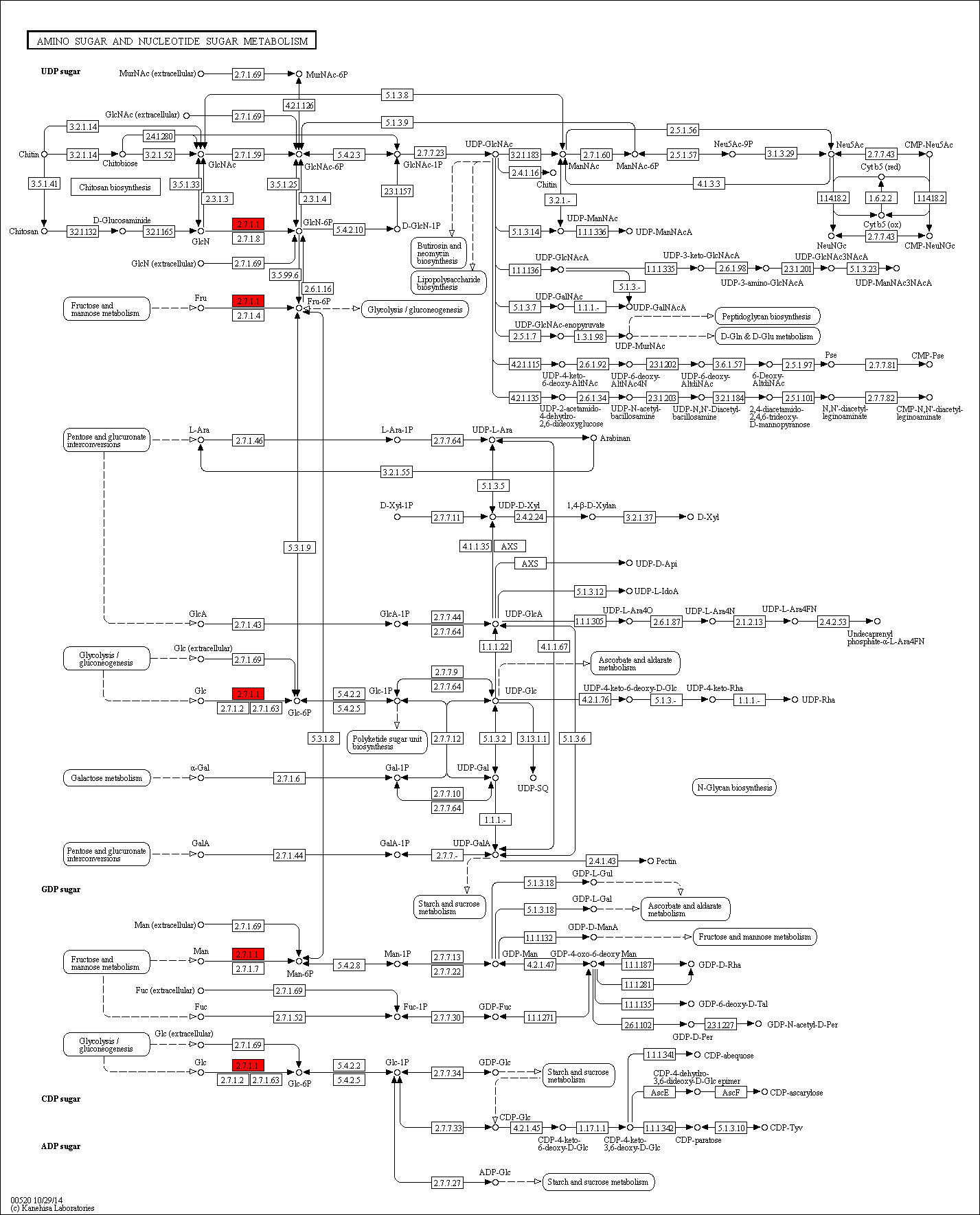

Supplement: S1 File — (ZIP) [file pone.0261403.s011.zip › ED/Anno/GeneAnno/pathway/kegg_map/ko00520.png]

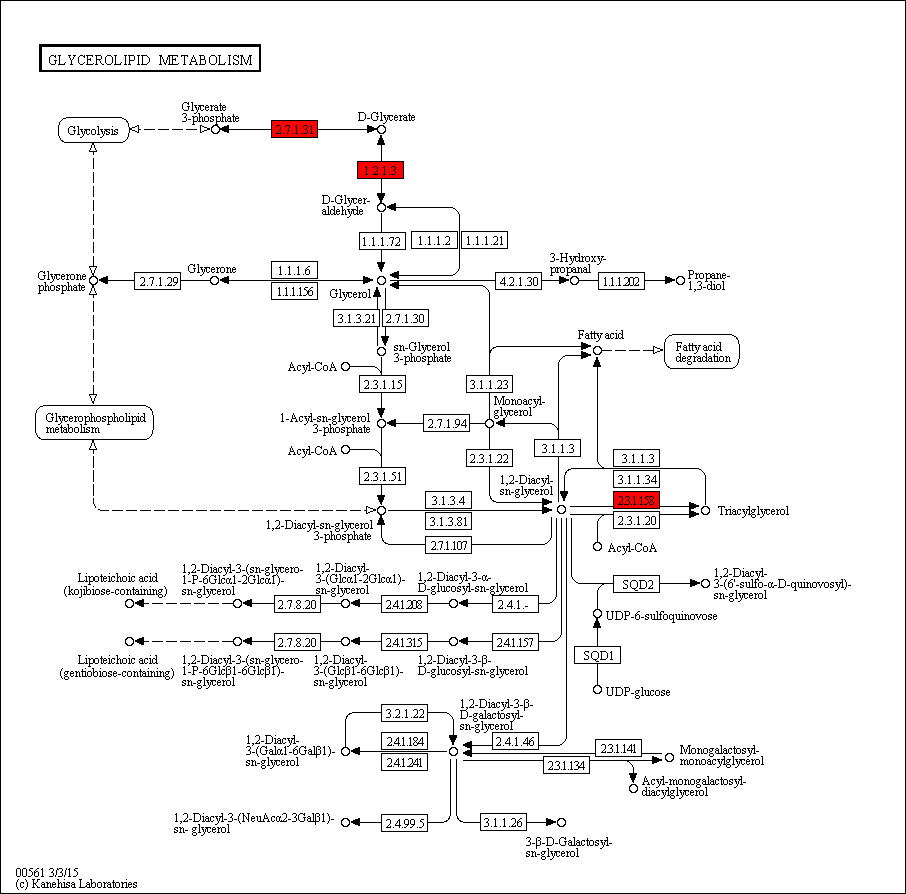

Supplement: S1 File — (ZIP) [file pone.0261403.s011.zip › ED/Anno/GeneAnno/pathway/kegg_map/ko00561.png]

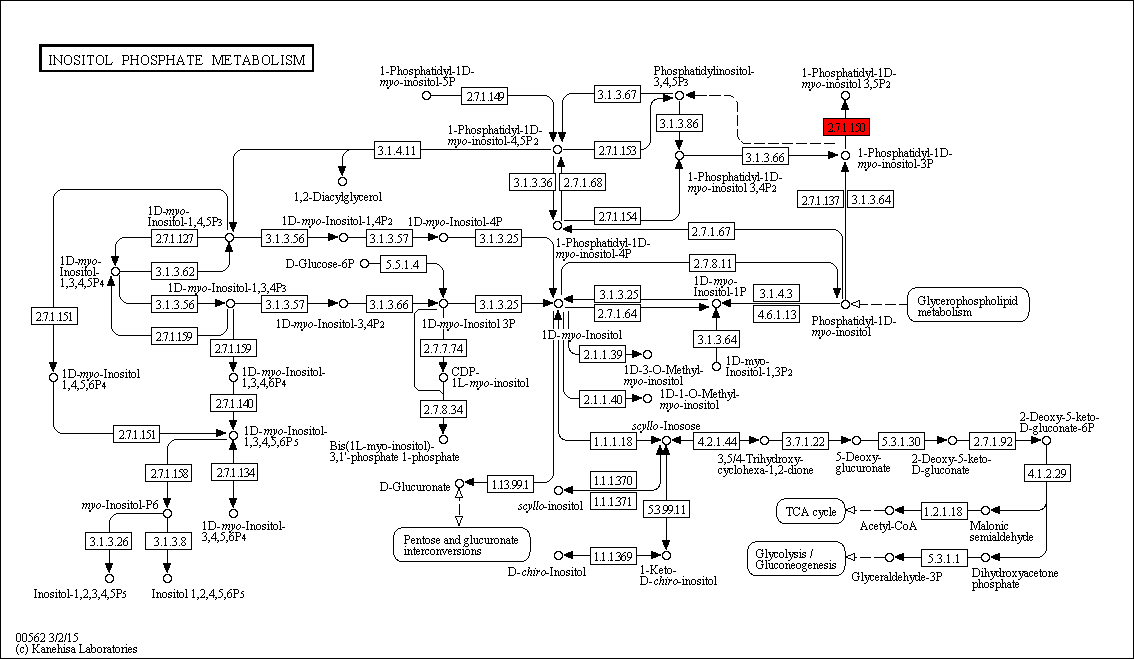

Supplement: S1 File — (ZIP) [file pone.0261403.s011.zip › ED/Anno/GeneAnno/pathway/kegg_map/ko00562.png]

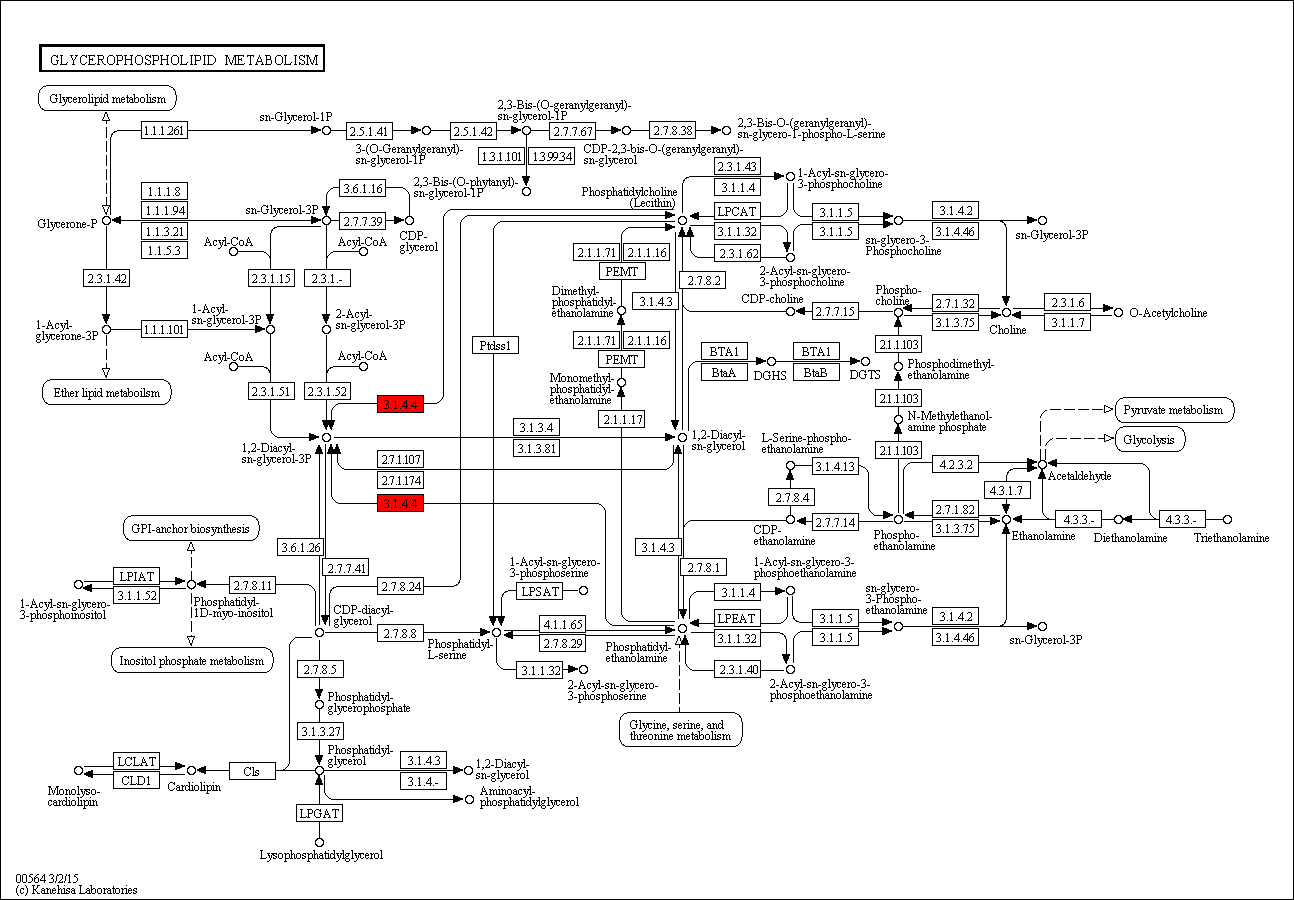

Supplement: S1 File — (ZIP) [file pone.0261403.s011.zip › ED/Anno/GeneAnno/pathway/kegg_map/ko00564.png]

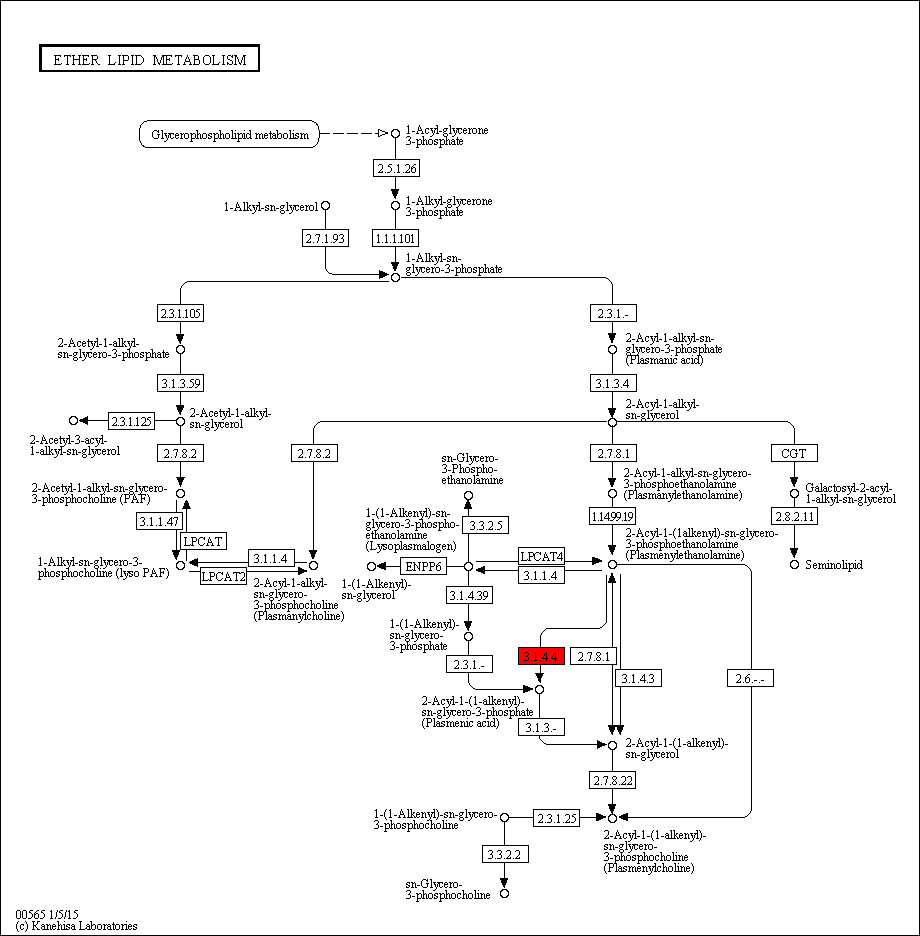

Supplement: S1 File — (ZIP) [file pone.0261403.s011.zip › ED/Anno/GeneAnno/pathway/kegg_map/ko00565.png]

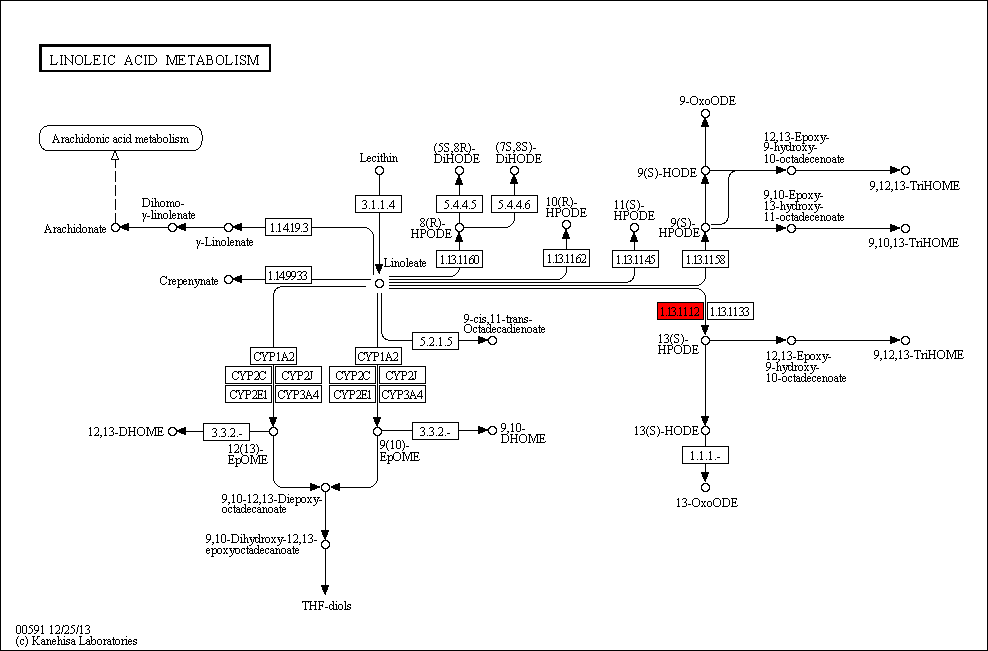

Supplement: S1 File — (ZIP) [file pone.0261403.s011.zip › ED/Anno/GeneAnno/pathway/kegg_map/ko00591.png]

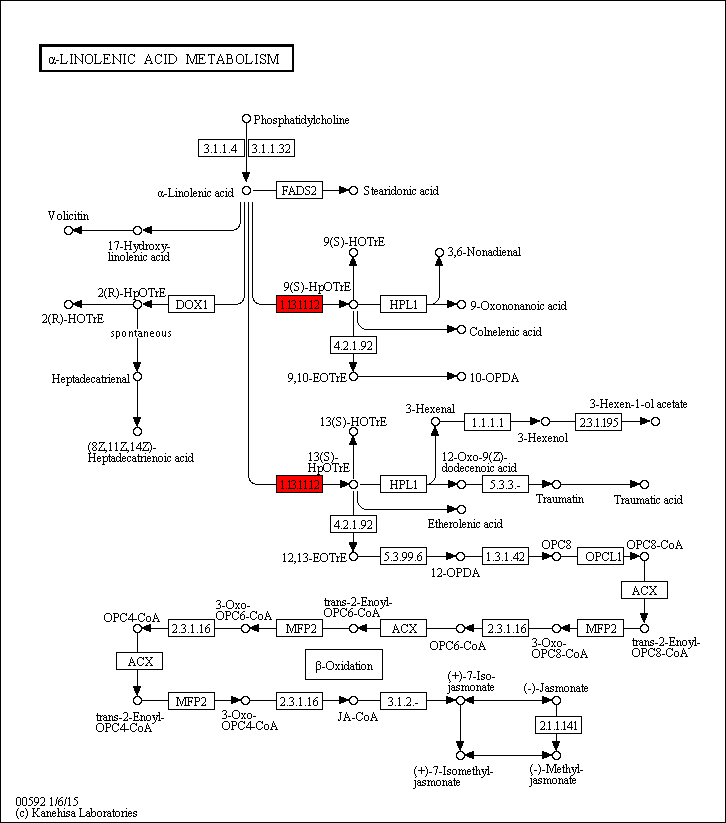

Supplement: S1 File — (ZIP) [file pone.0261403.s011.zip › ED/Anno/GeneAnno/pathway/kegg_map/ko00592.png]

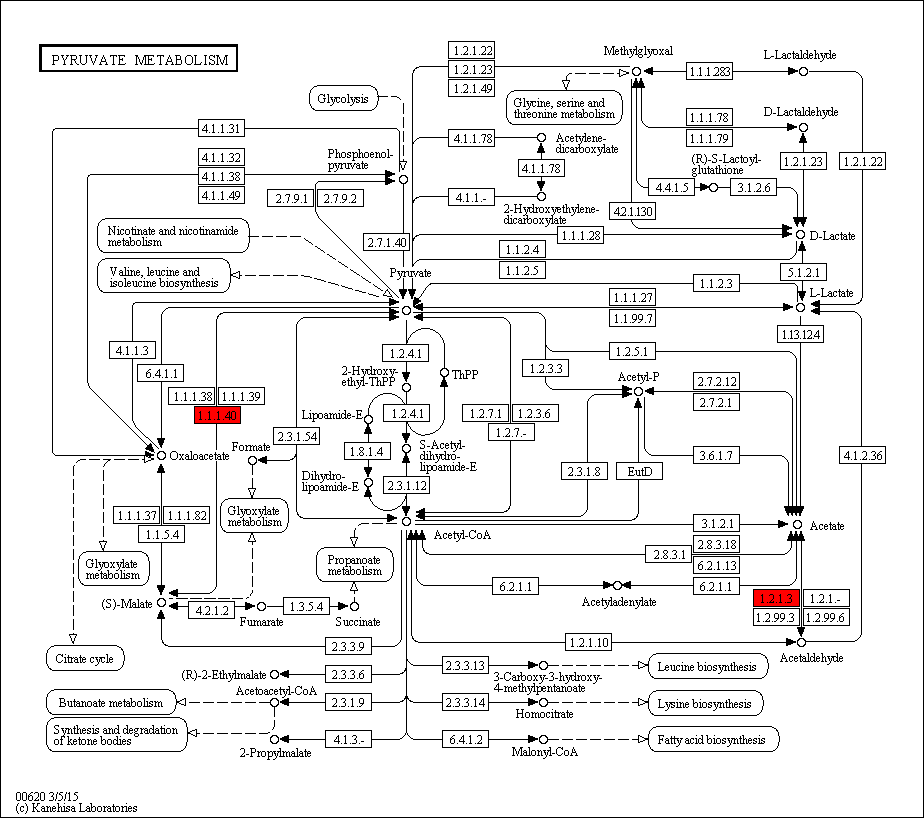

Supplement: S1 File — (ZIP) [file pone.0261403.s011.zip › ED/Anno/GeneAnno/pathway/kegg_map/ko00620.png]

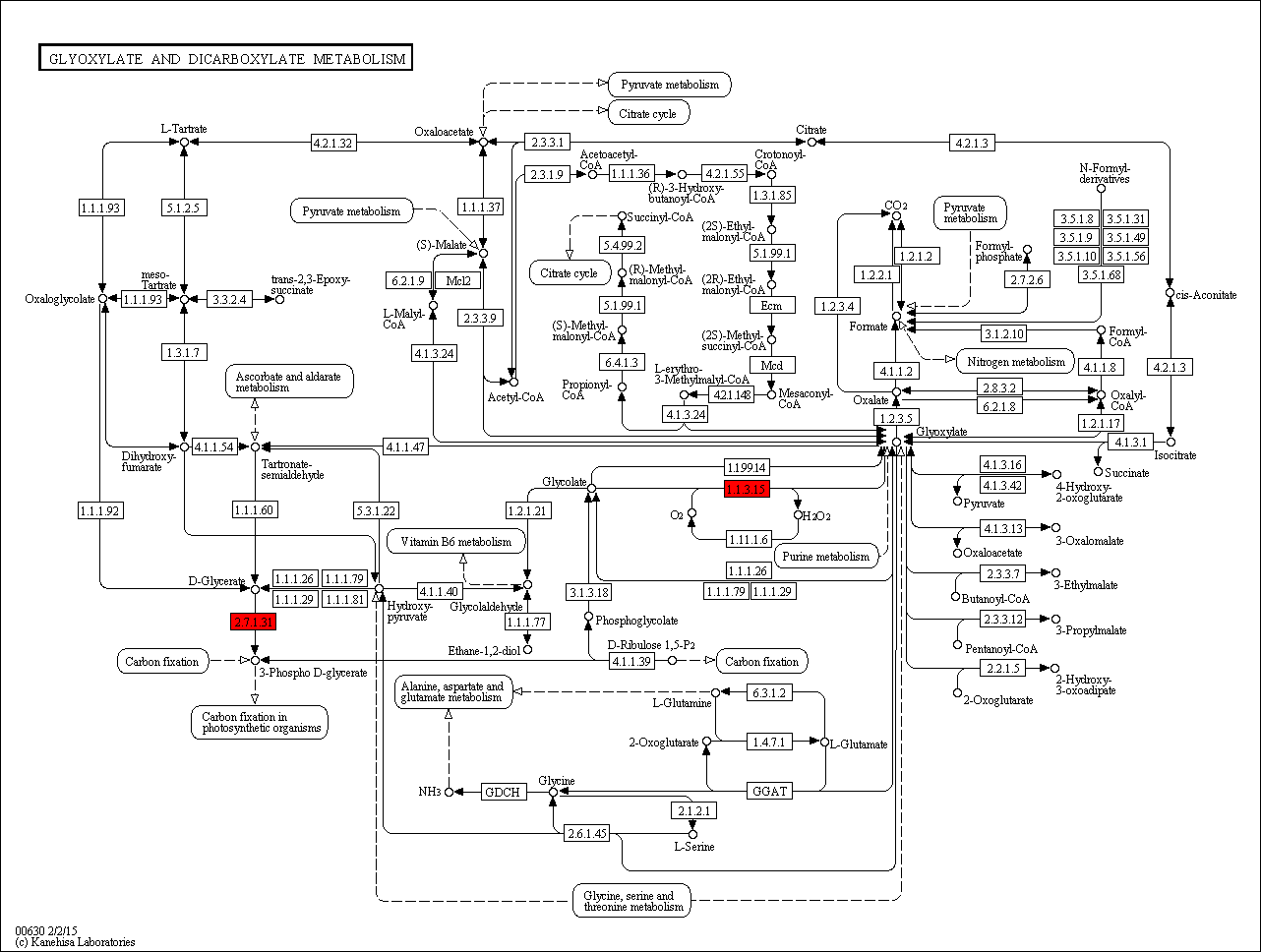

Supplement: S1 File — (ZIP) [file pone.0261403.s011.zip › ED/Anno/GeneAnno/pathway/kegg_map/ko00630.png]

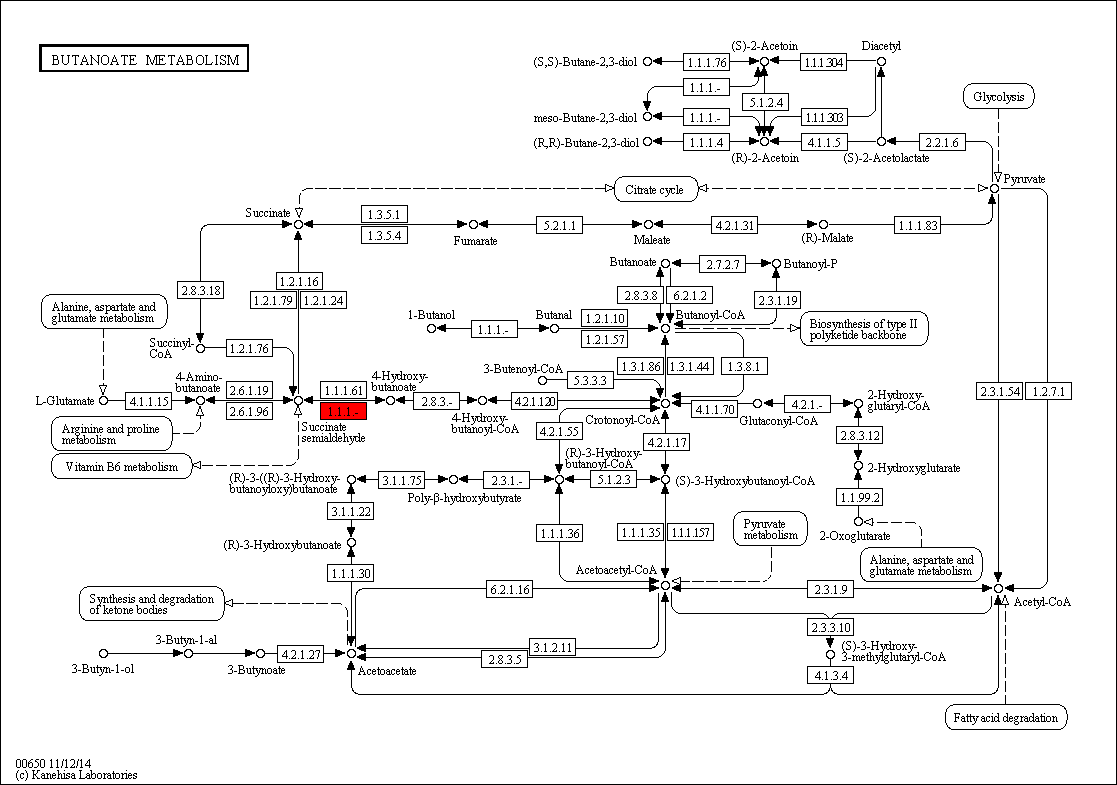

Supplement: S1 File — (ZIP) [file pone.0261403.s011.zip › ED/Anno/GeneAnno/pathway/kegg_map/ko00650.png]

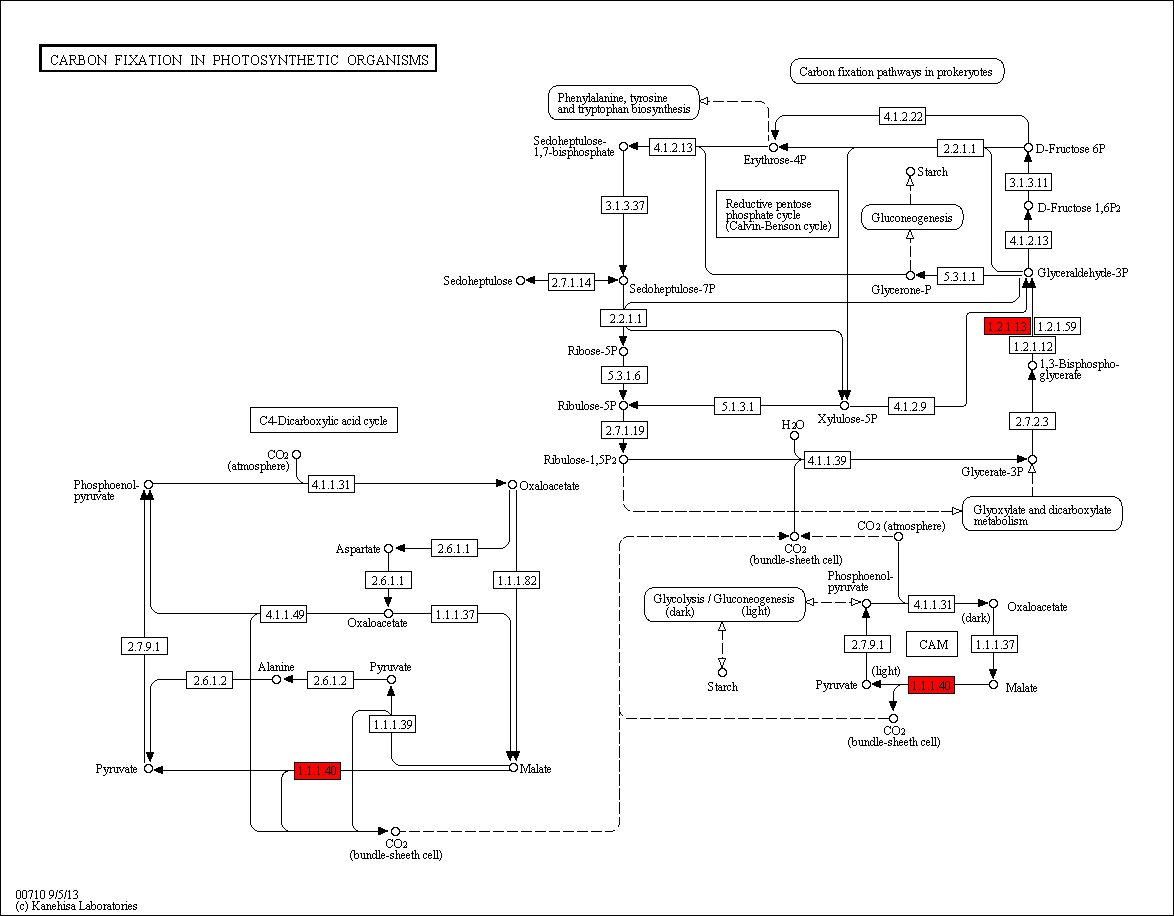

Supplement: S1 File — (ZIP) [file pone.0261403.s011.zip › ED/Anno/GeneAnno/pathway/kegg_map/ko00710.png]

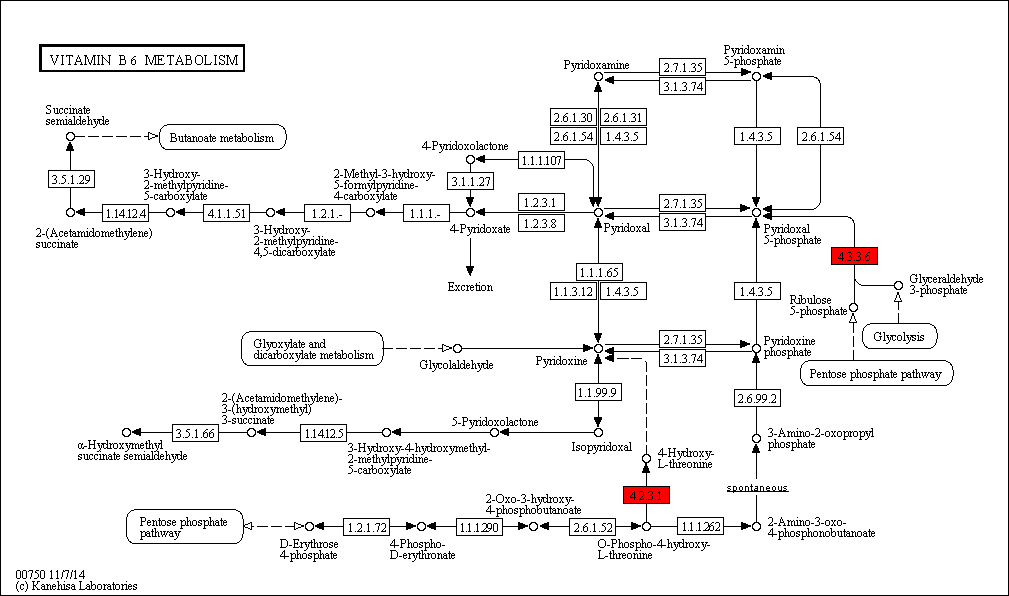

Supplement: S1 File — (ZIP) [file pone.0261403.s011.zip › ED/Anno/GeneAnno/pathway/kegg_map/ko00750.png]

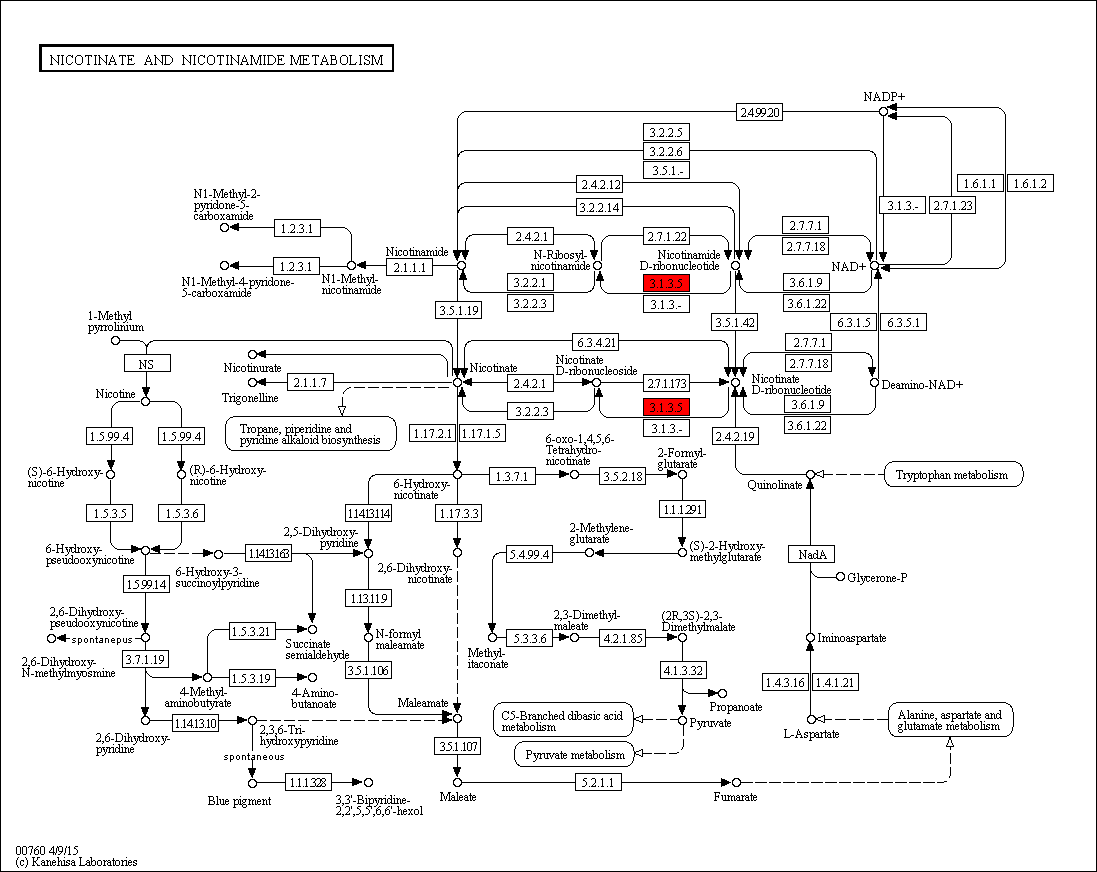

Supplement: S1 File — (ZIP) [file pone.0261403.s011.zip › ED/Anno/GeneAnno/pathway/kegg_map/ko00760.png]

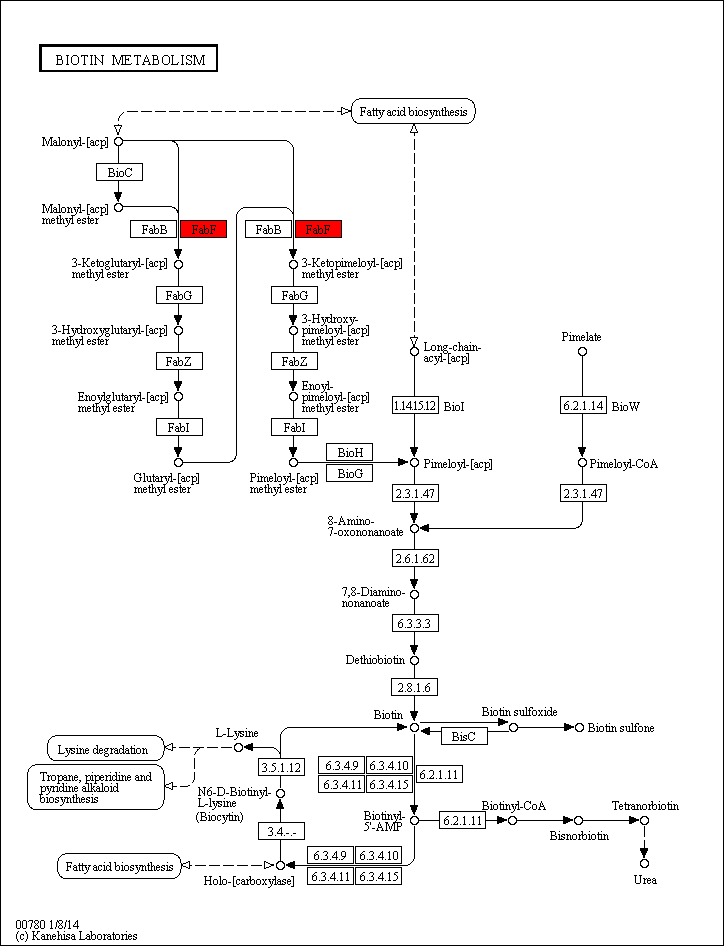

Supplement: S1 File — (ZIP) [file pone.0261403.s011.zip › ED/Anno/GeneAnno/pathway/kegg_map/ko00780.png]

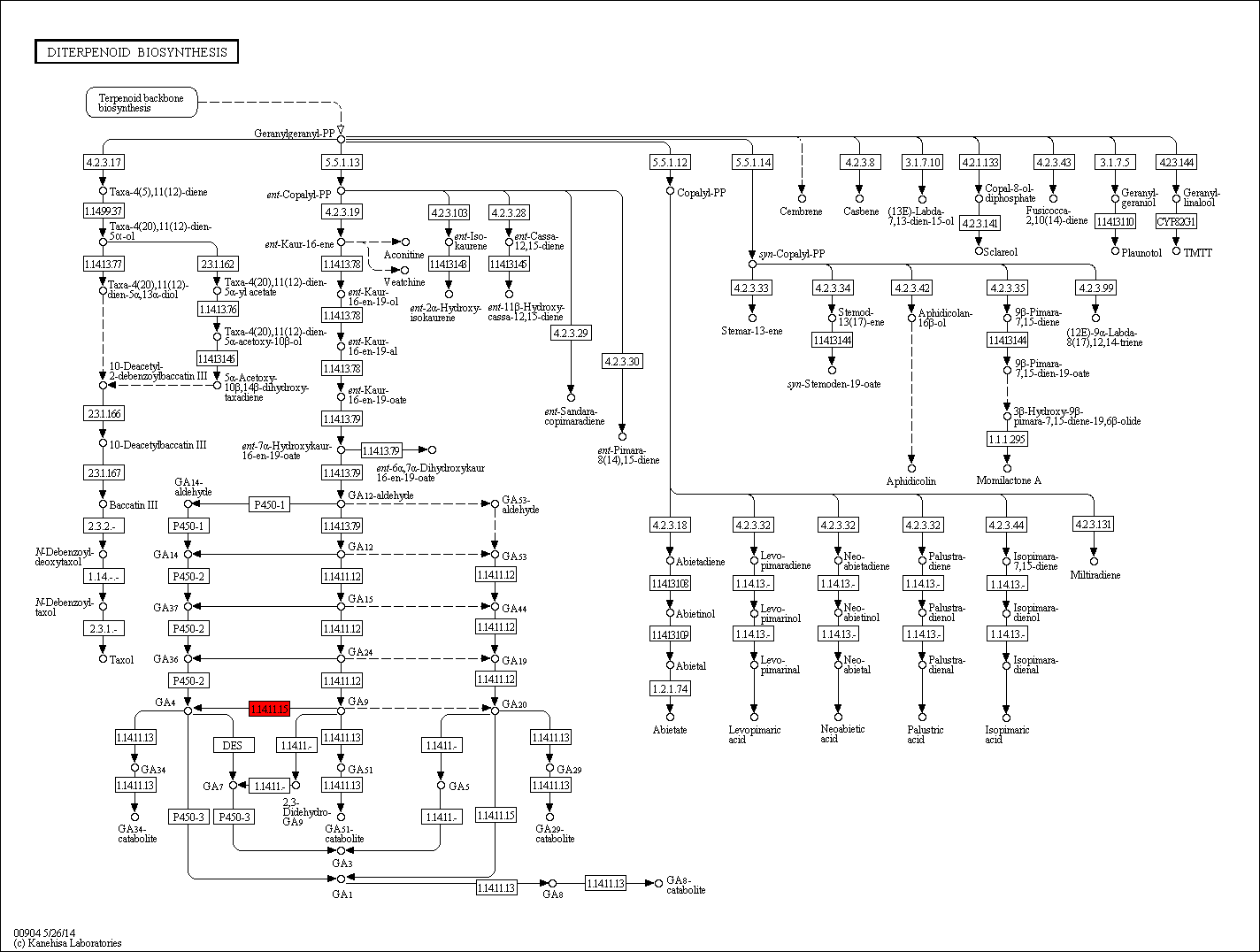

Supplement: S1 File — (ZIP) [file pone.0261403.s011.zip › ED/Anno/GeneAnno/pathway/kegg_map/ko00904.png]

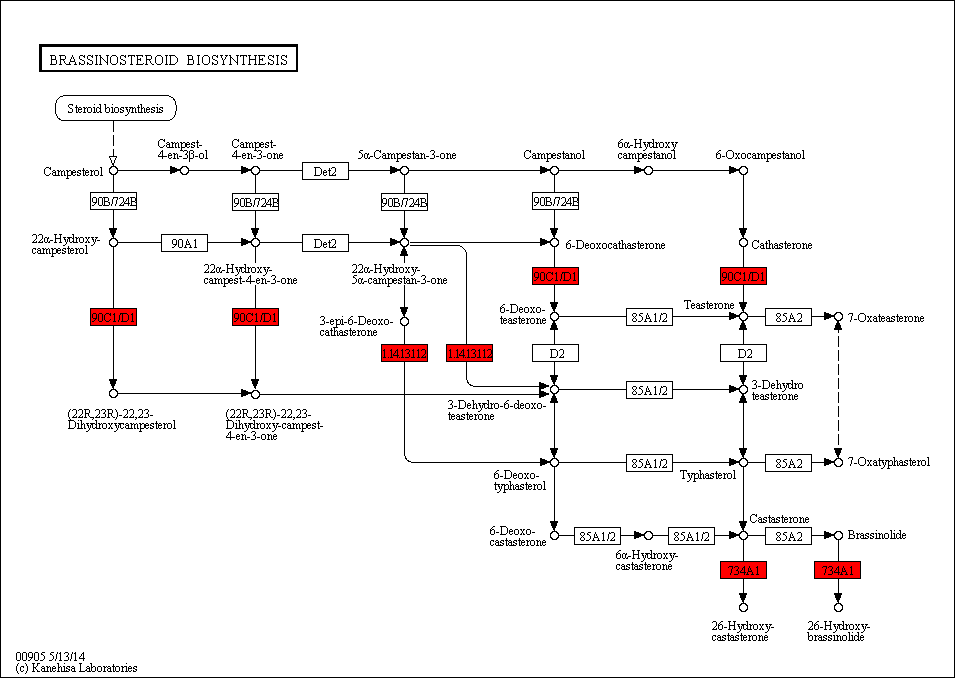

Supplement: S1 File — (ZIP) [file pone.0261403.s011.zip › ED/Anno/GeneAnno/pathway/kegg_map/ko00905.png]

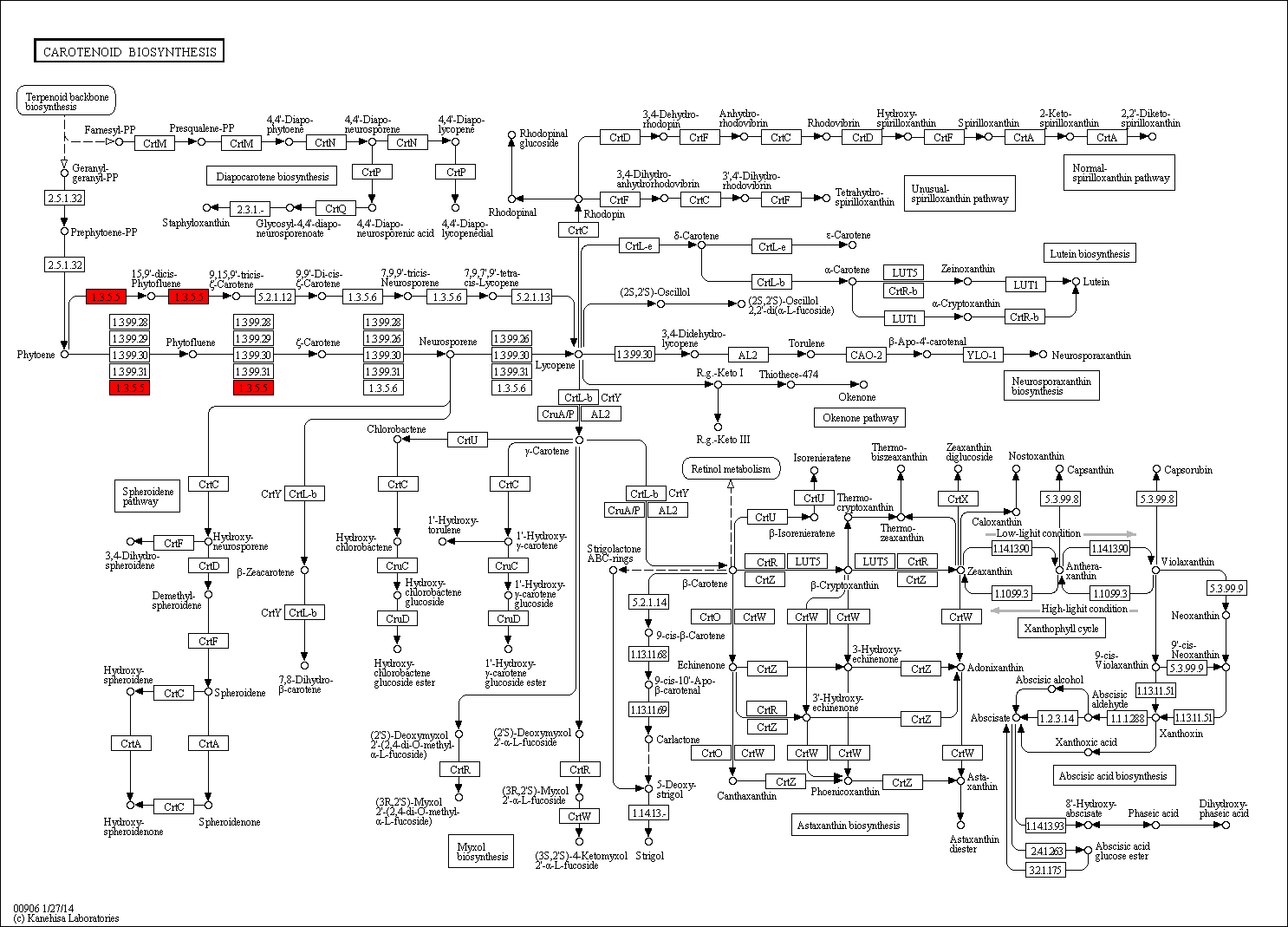

Supplement: S1 File — (ZIP) [file pone.0261403.s011.zip › ED/Anno/GeneAnno/pathway/kegg_map/ko00906.png]

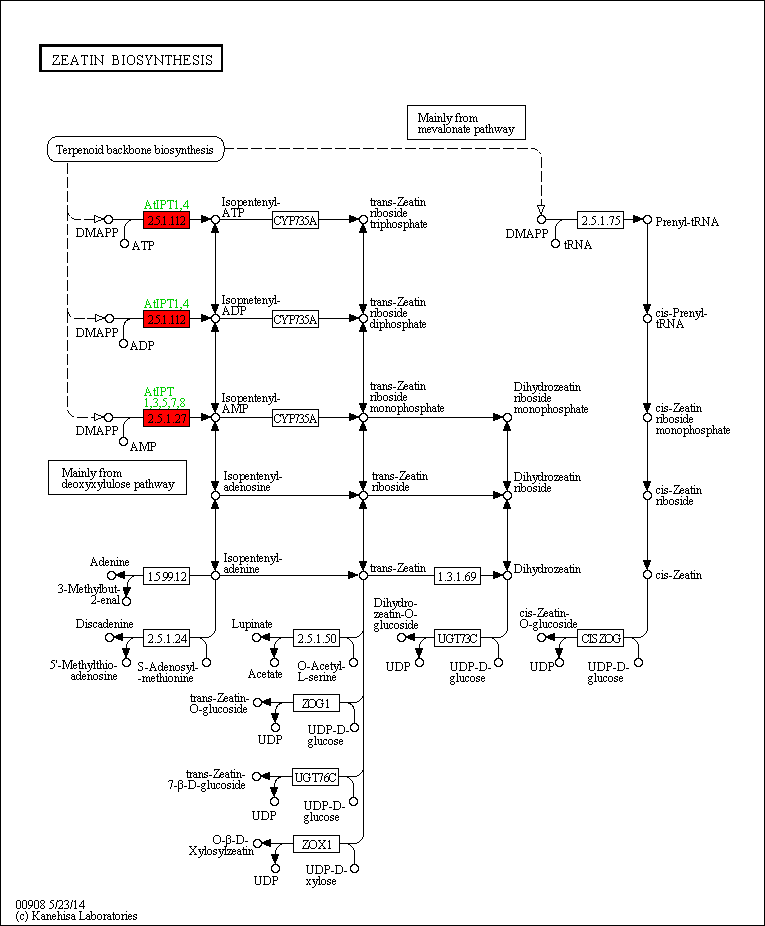

Supplement: S1 File — (ZIP) [file pone.0261403.s011.zip › ED/Anno/GeneAnno/pathway/kegg_map/ko00908.png]

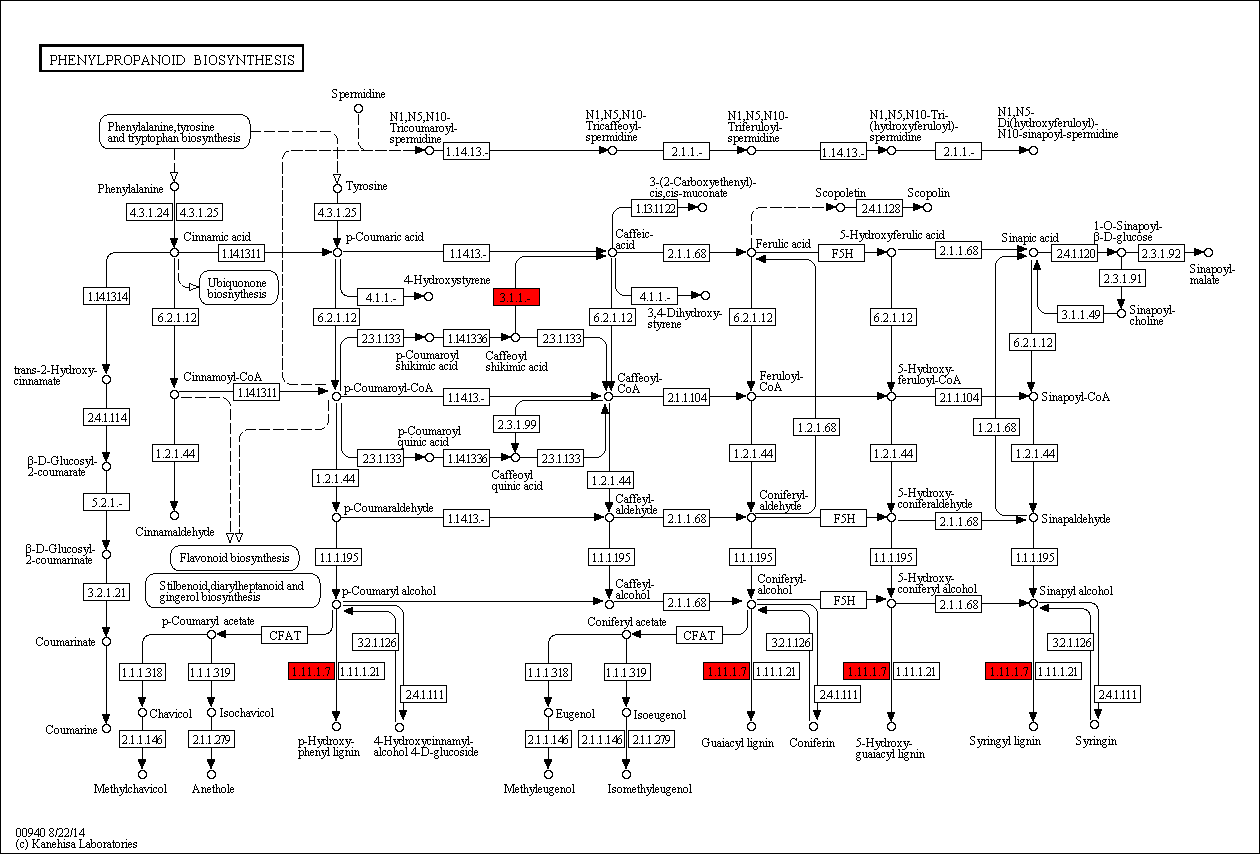

Supplement: S1 File — (ZIP) [file pone.0261403.s011.zip › ED/Anno/GeneAnno/pathway/kegg_map/ko00940.png]

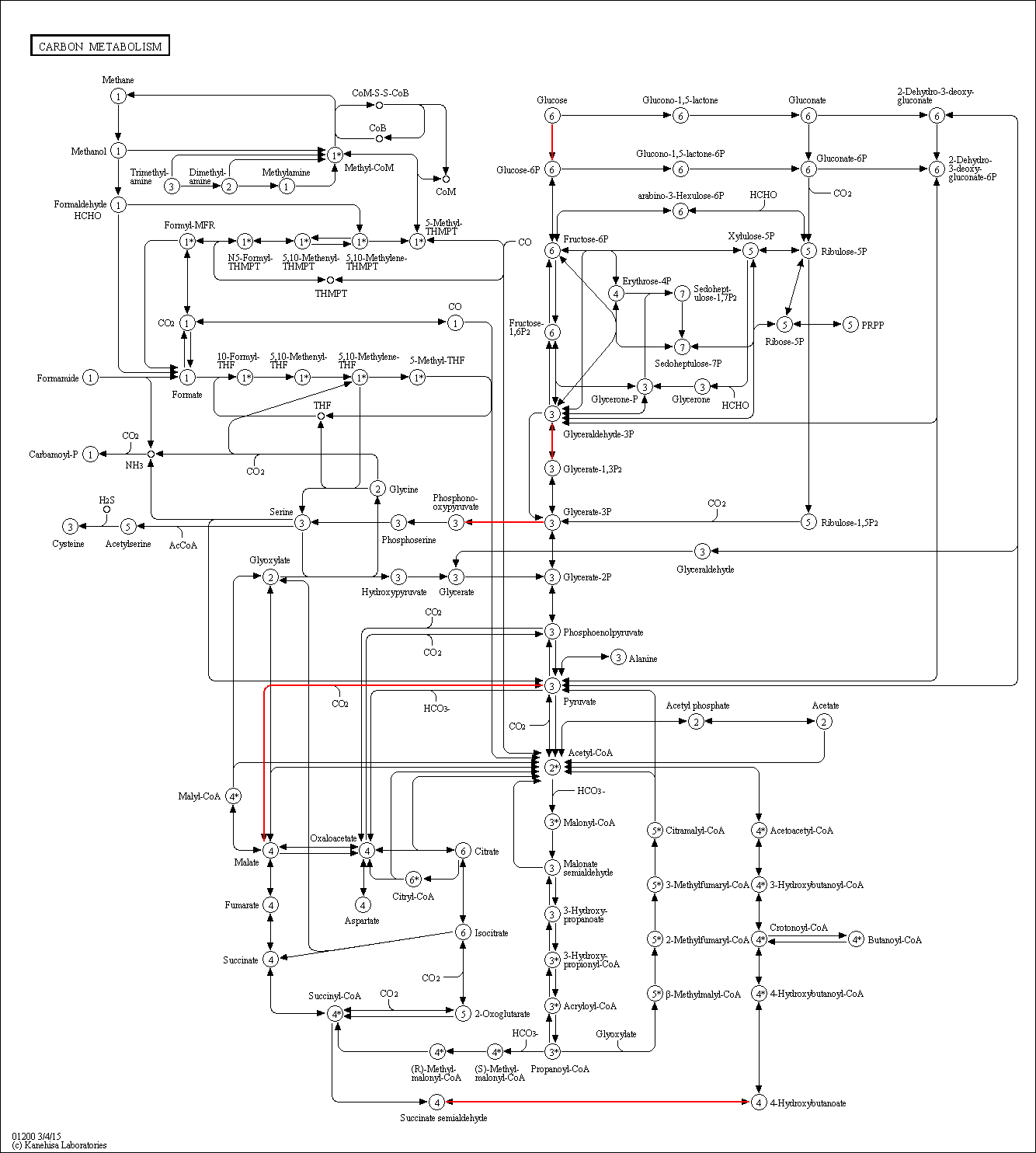

Supplement: S1 File — (ZIP) [file pone.0261403.s011.zip › ED/Anno/GeneAnno/pathway/kegg_map/ko01200.png]

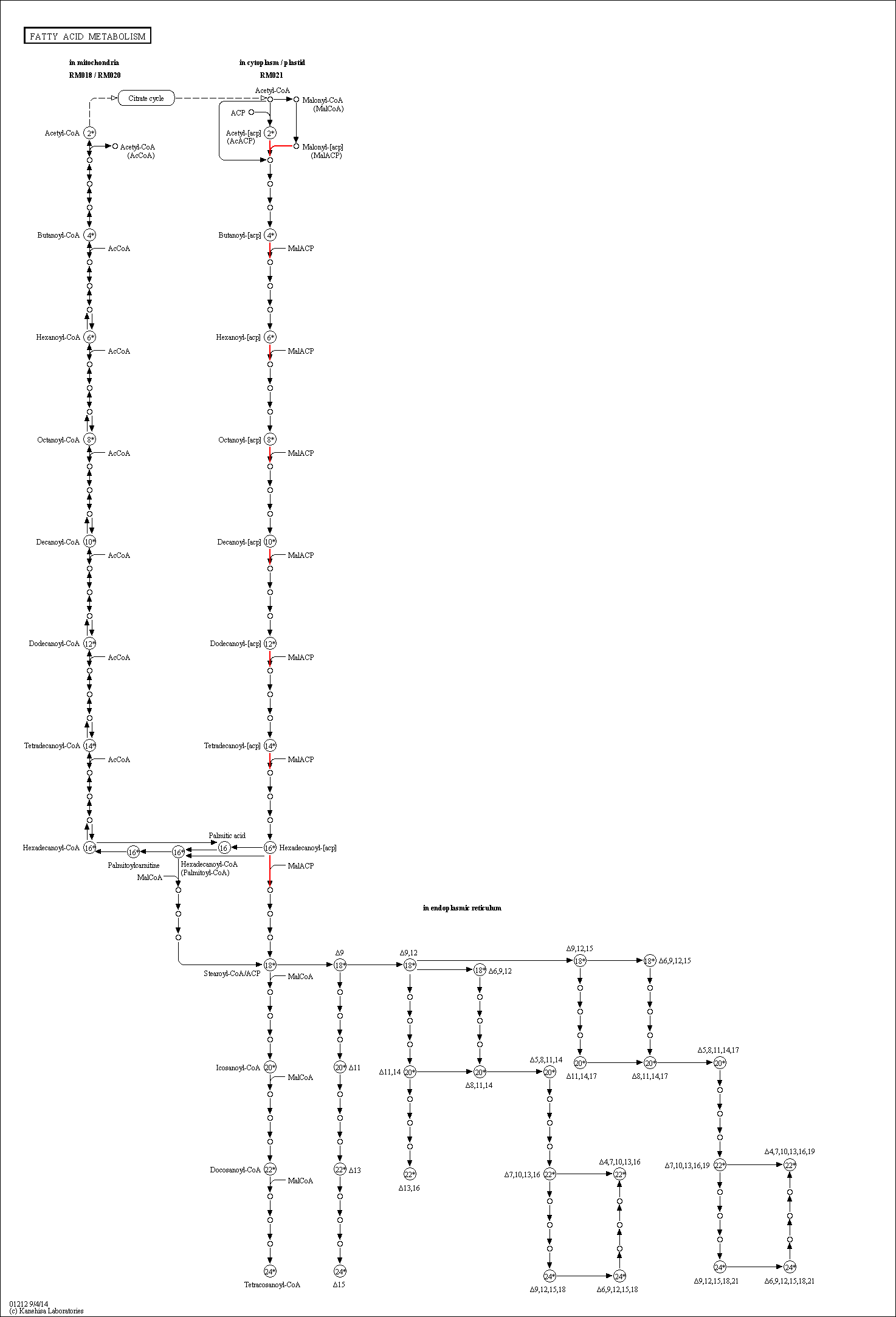

Supplement: S1 File — (ZIP) [file pone.0261403.s011.zip › ED/Anno/GeneAnno/pathway/kegg_map/ko01212.png]

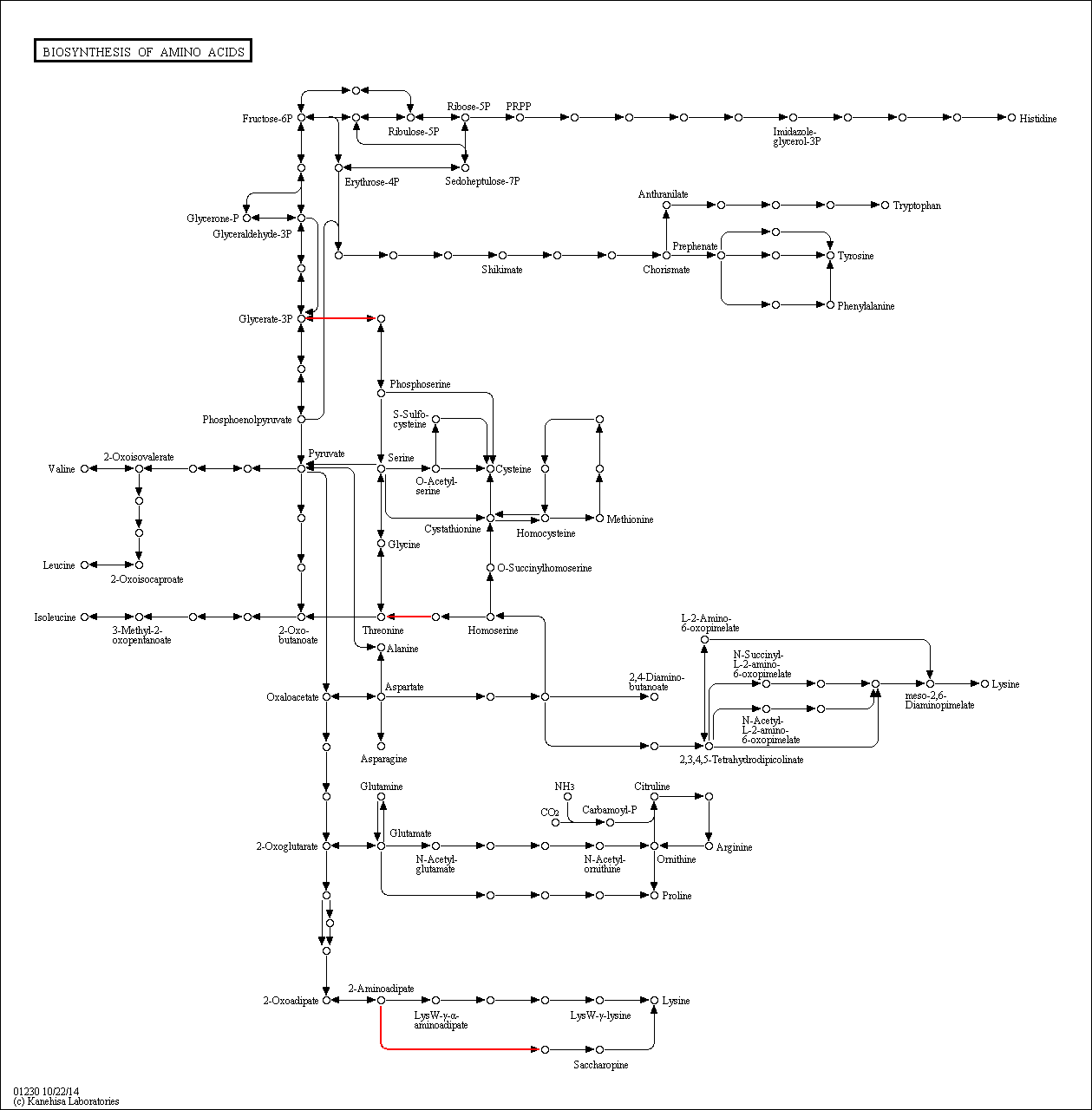

Supplement: S1 File — (ZIP) [file pone.0261403.s011.zip › ED/Anno/GeneAnno/pathway/kegg_map/ko01230.png]

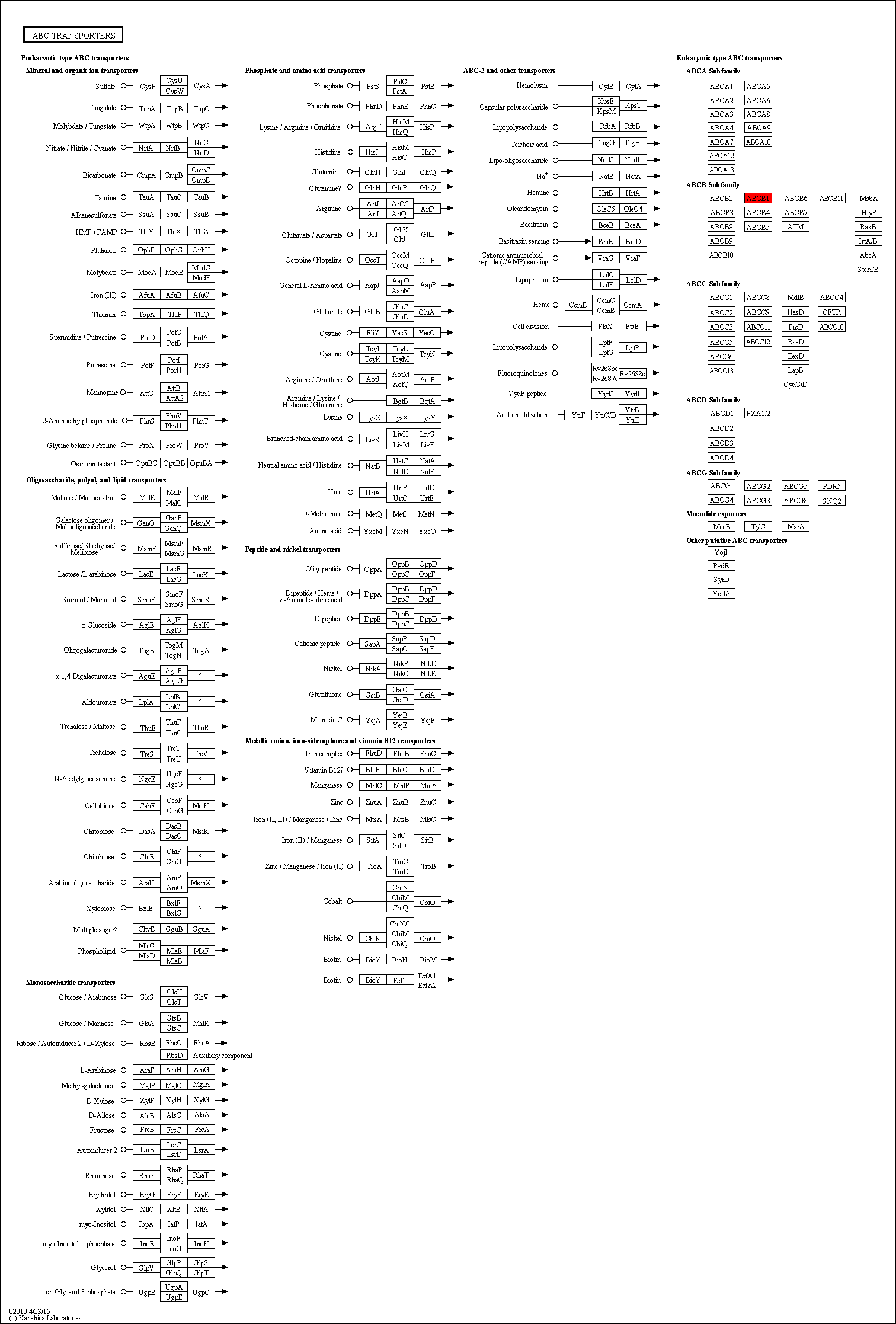

Supplement: S1 File — (ZIP) [file pone.0261403.s011.zip › ED/Anno/GeneAnno/pathway/kegg_map/ko02010.png]

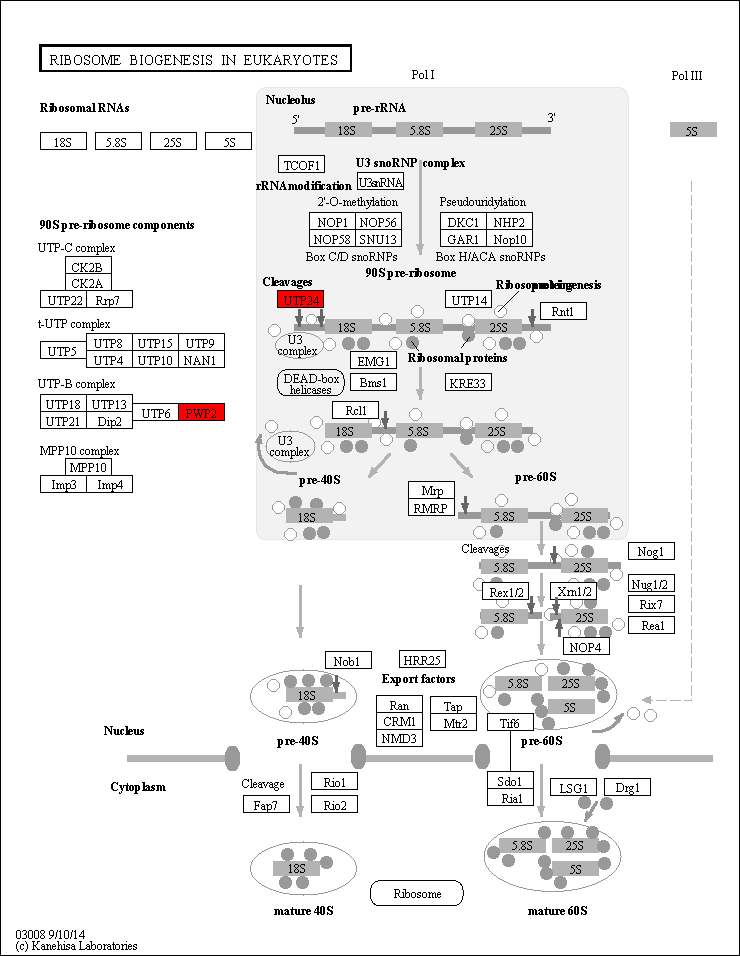

Supplement: S1 File — (ZIP) [file pone.0261403.s011.zip › ED/Anno/GeneAnno/pathway/kegg_map/ko03008.png]

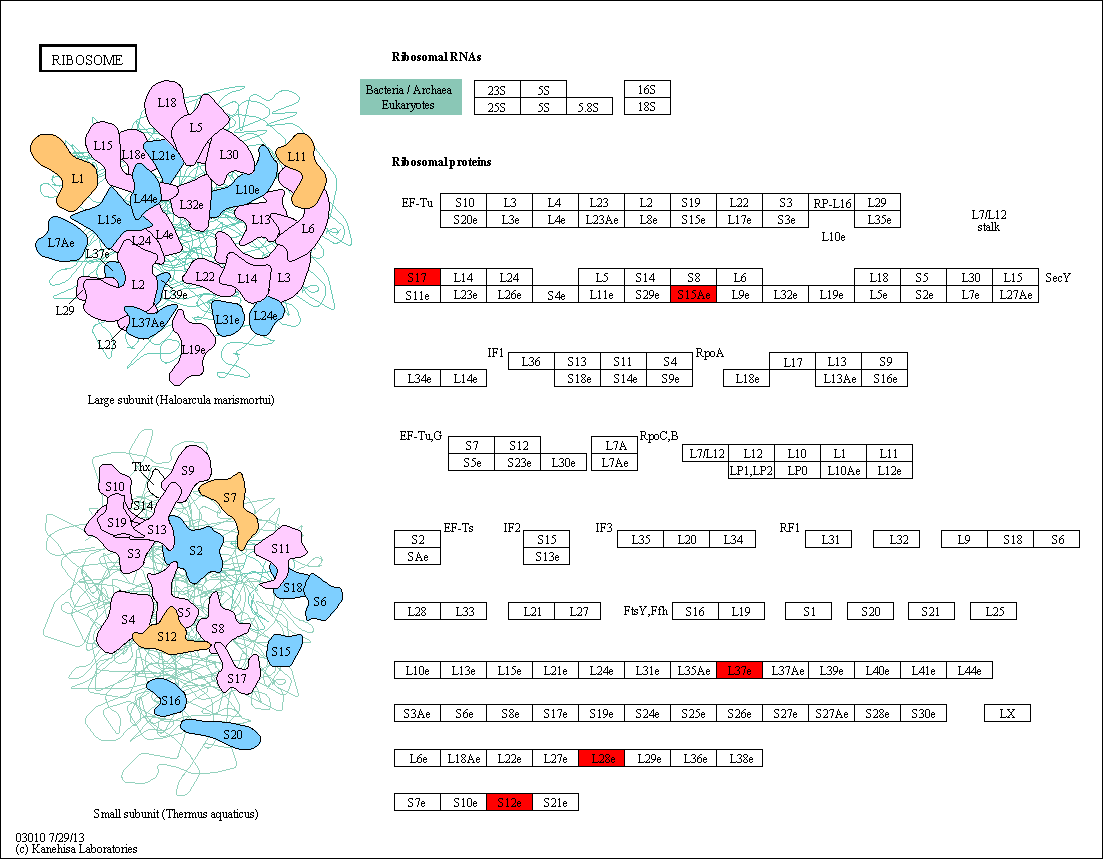

Supplement: S1 File — (ZIP) [file pone.0261403.s011.zip › ED/Anno/GeneAnno/pathway/kegg_map/ko03010.png]

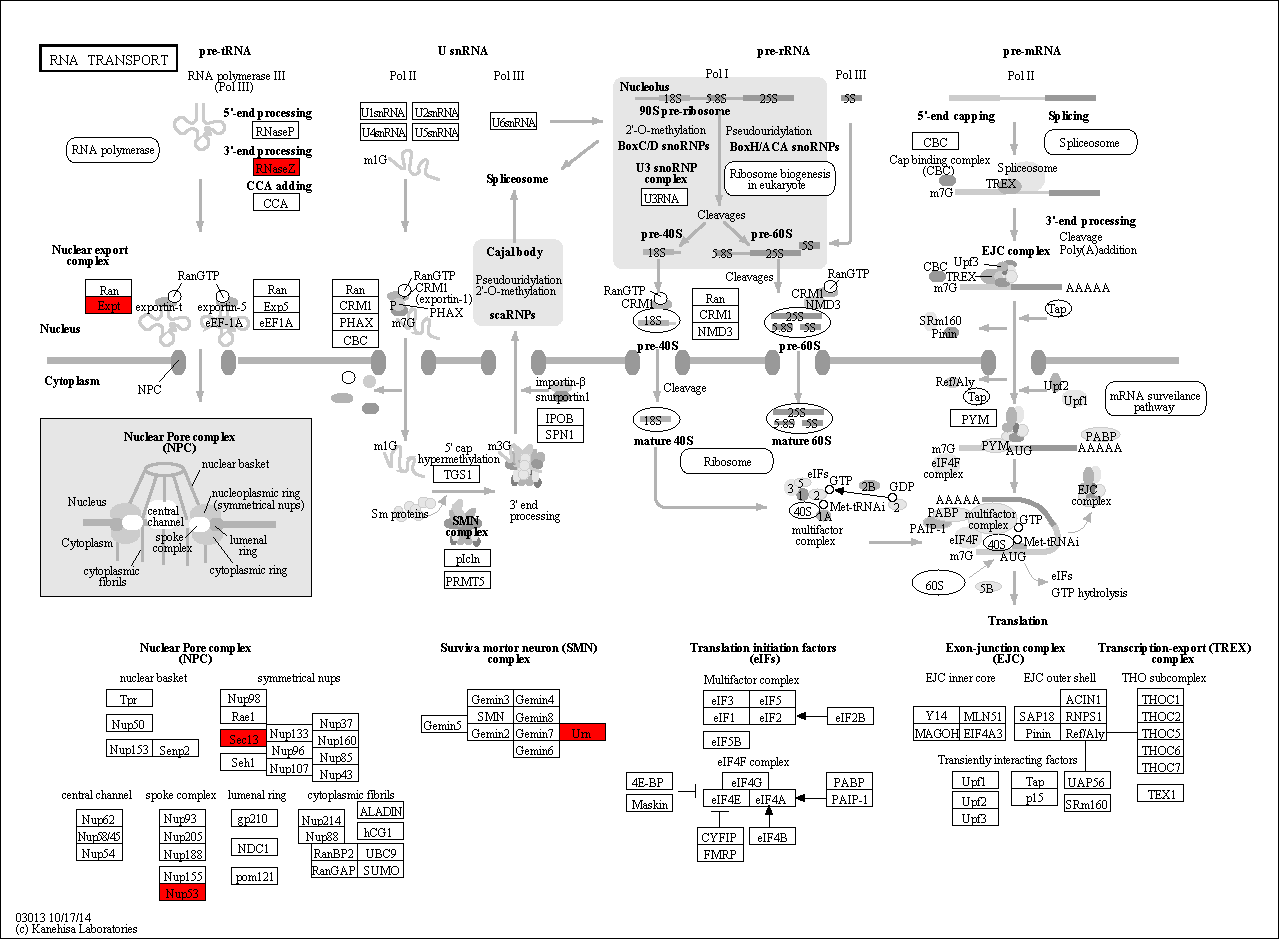

Supplement: S1 File — (ZIP) [file pone.0261403.s011.zip › ED/Anno/GeneAnno/pathway/kegg_map/ko03013.png]

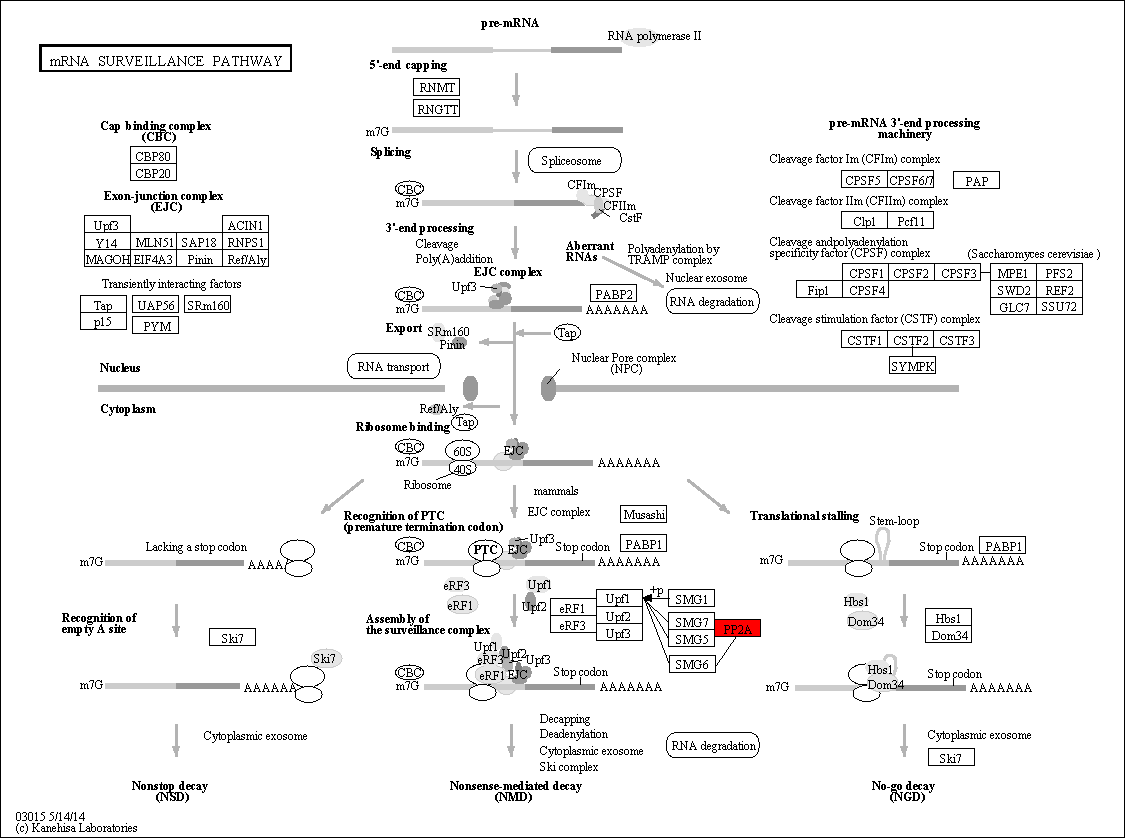

Supplement: S1 File — (ZIP) [file pone.0261403.s011.zip › ED/Anno/GeneAnno/pathway/kegg_map/ko03015.png]

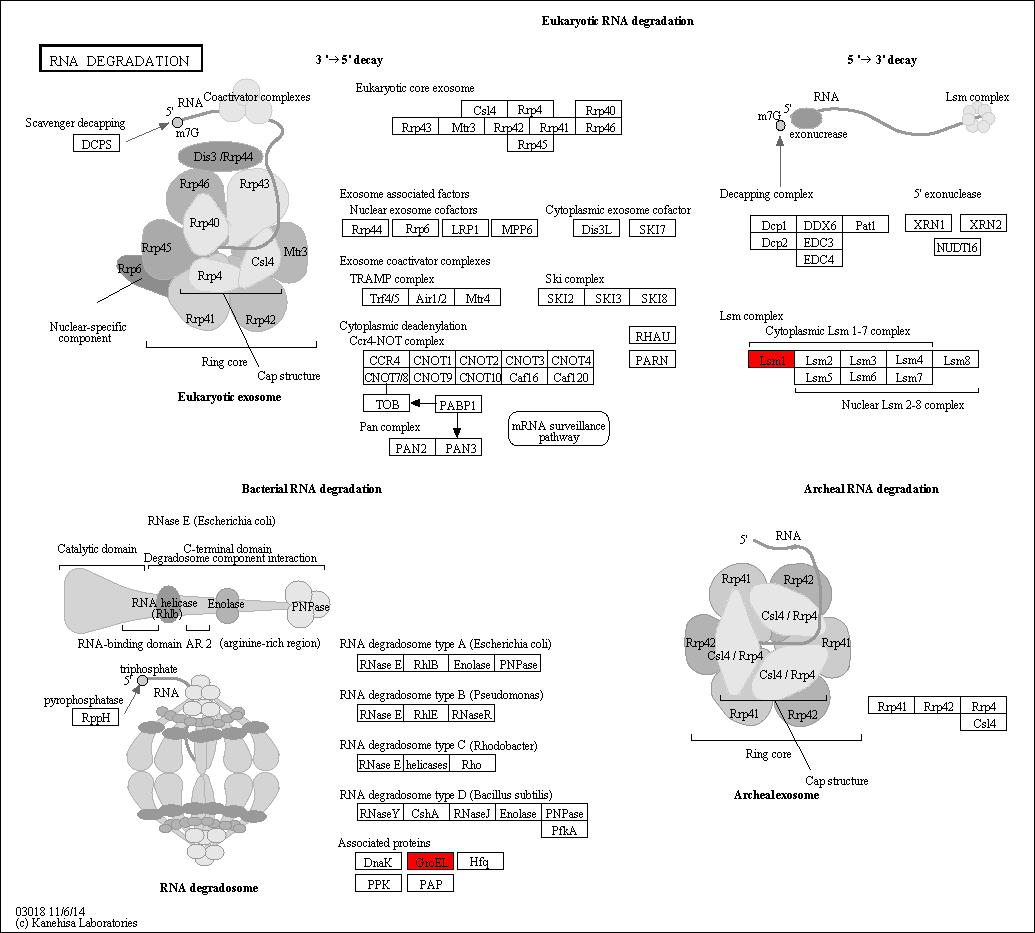

Supplement: S1 File — (ZIP) [file pone.0261403.s011.zip › ED/Anno/GeneAnno/pathway/kegg_map/ko03018.png]

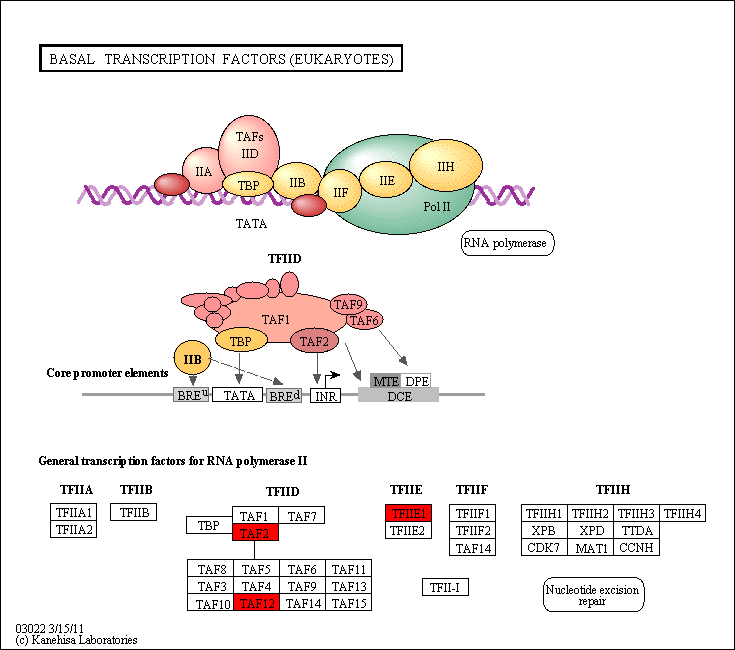

Supplement: S1 File — (ZIP) [file pone.0261403.s011.zip › ED/Anno/GeneAnno/pathway/kegg_map/ko03022.png]

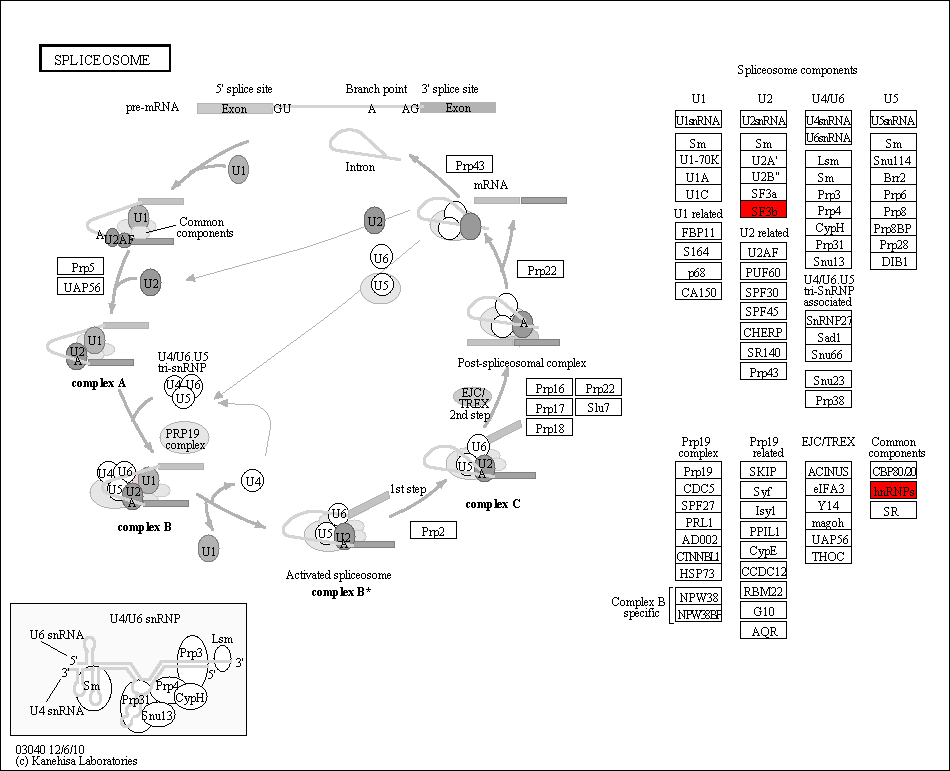

Supplement: S1 File — (ZIP) [file pone.0261403.s011.zip › ED/Anno/GeneAnno/pathway/kegg_map/ko03040.png]

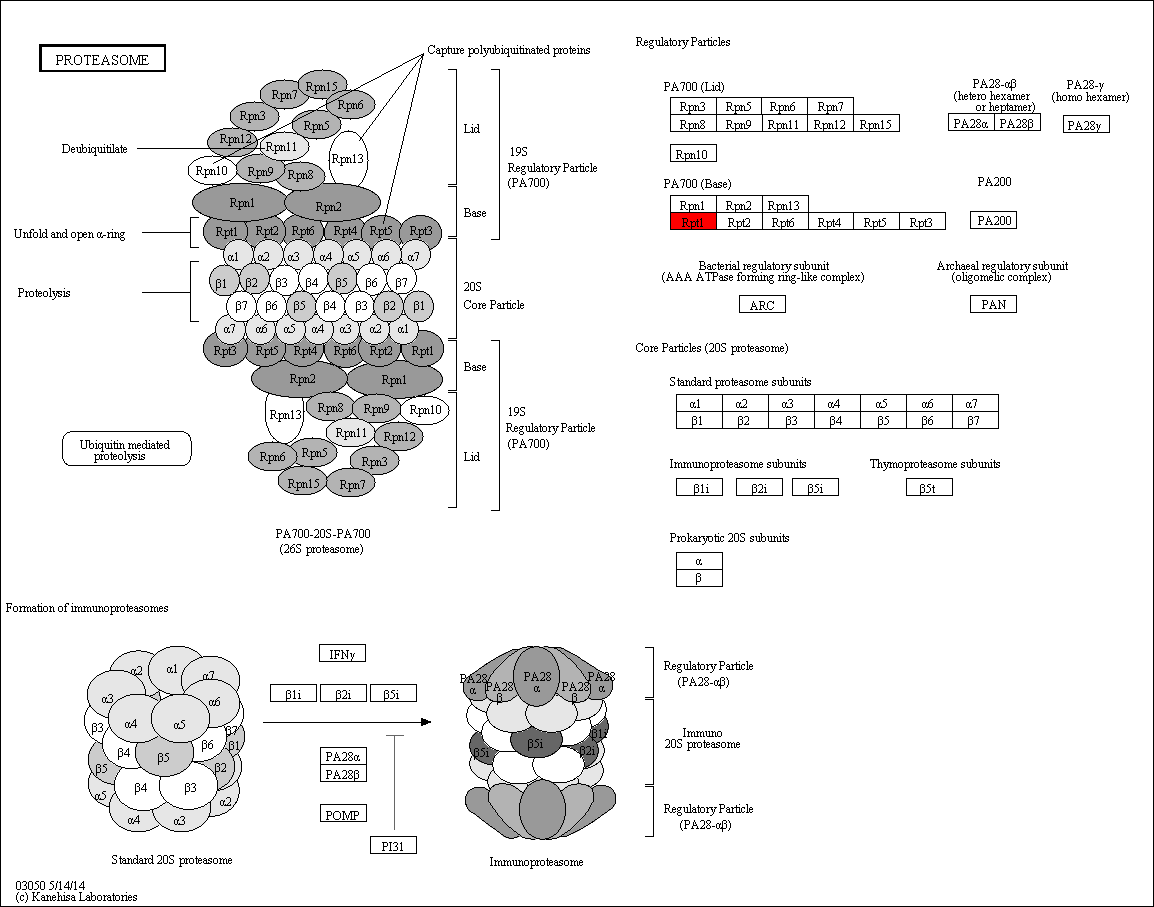

Supplement: S1 File — (ZIP) [file pone.0261403.s011.zip › ED/Anno/GeneAnno/pathway/kegg_map/ko03050.png]

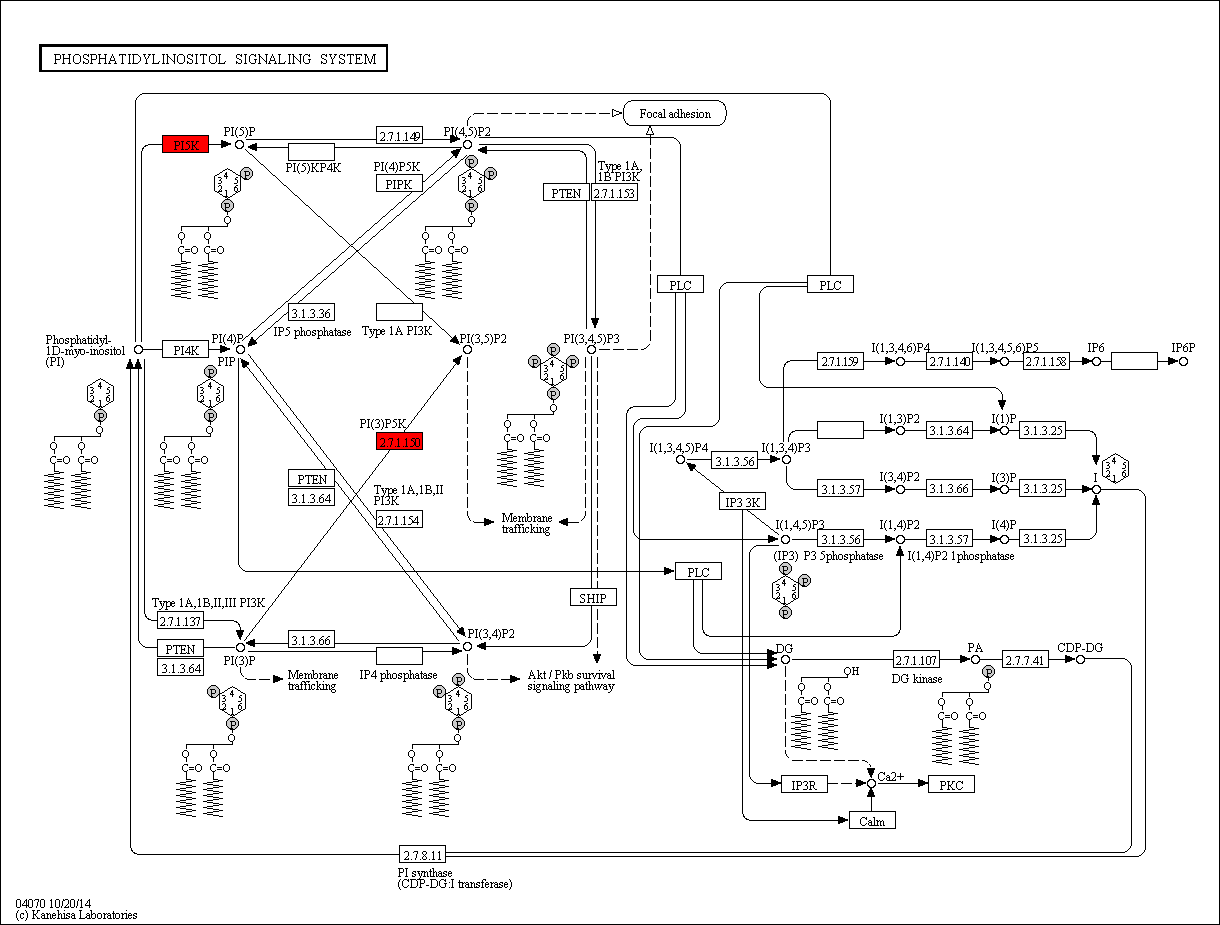

Supplement: S1 File — (ZIP) [file pone.0261403.s011.zip › ED/Anno/GeneAnno/pathway/kegg_map/ko04070.png]

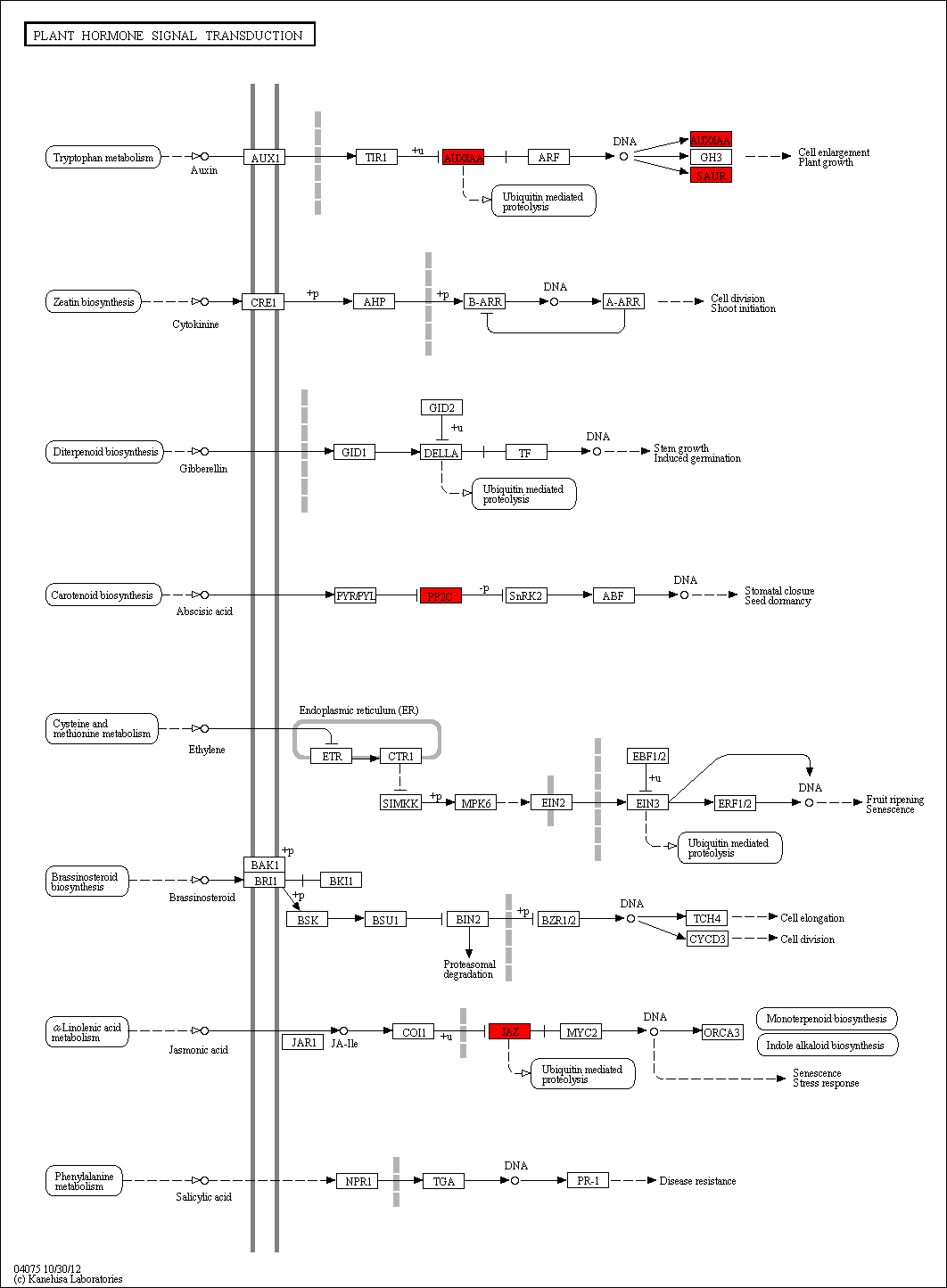

Supplement: S1 File — (ZIP) [file pone.0261403.s011.zip › ED/Anno/GeneAnno/pathway/kegg_map/ko04075.png]

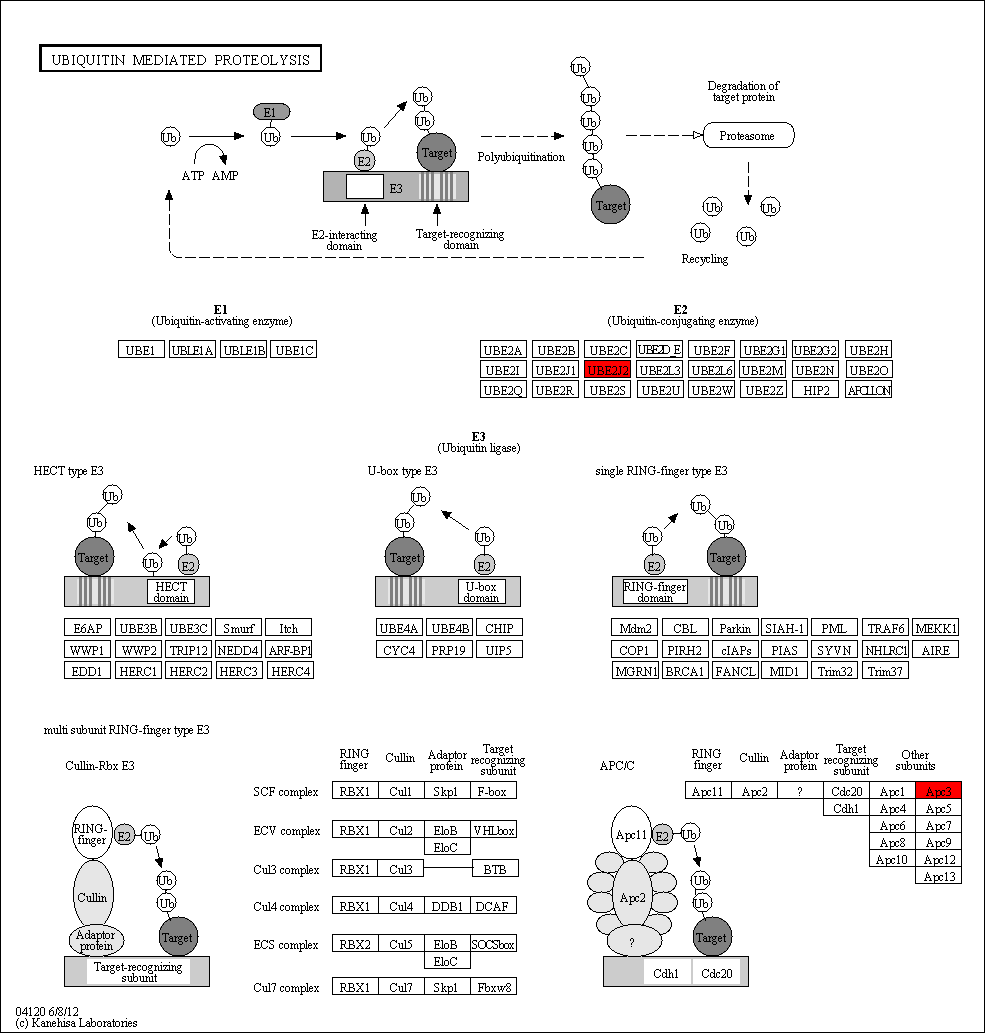

Supplement: S1 File — (ZIP) [file pone.0261403.s011.zip › ED/Anno/GeneAnno/pathway/kegg_map/ko04120.png]

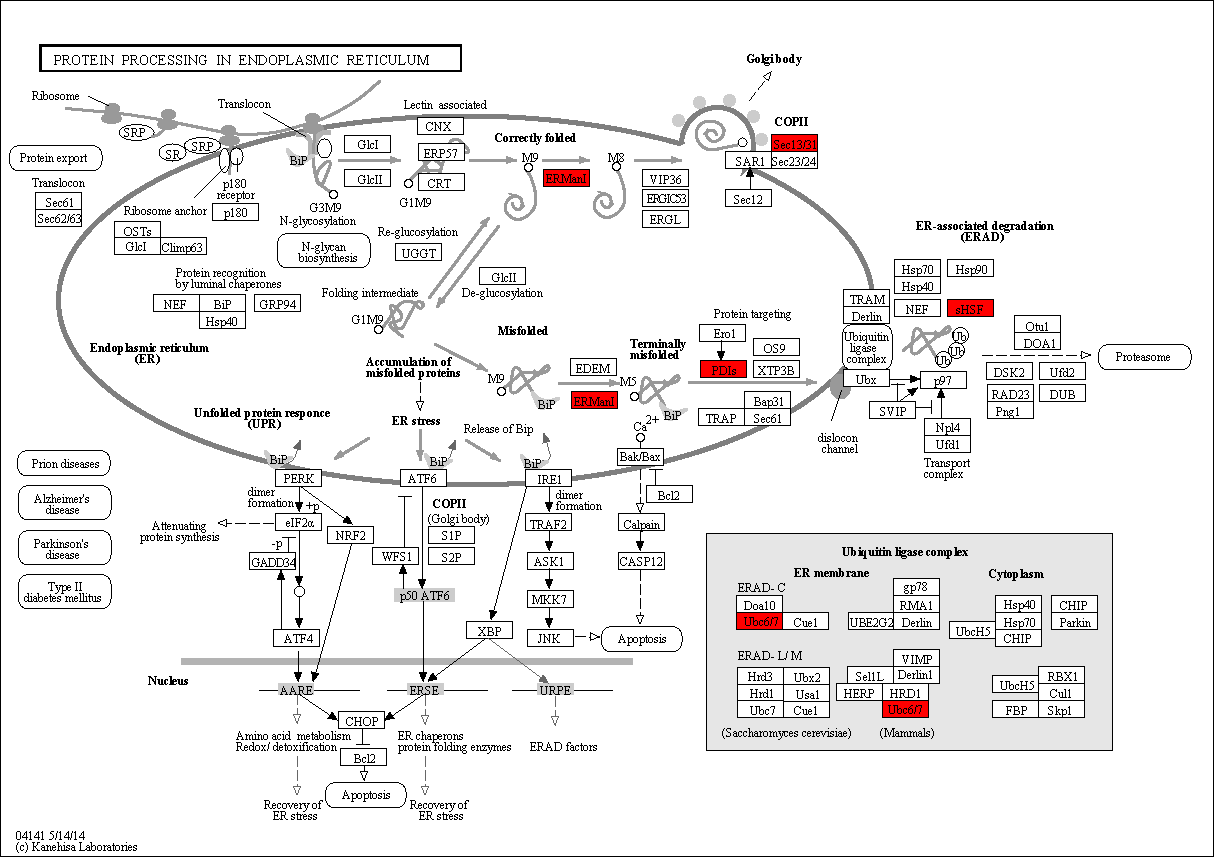

Supplement: S1 File — (ZIP) [file pone.0261403.s011.zip › ED/Anno/GeneAnno/pathway/kegg_map/ko04141.png]

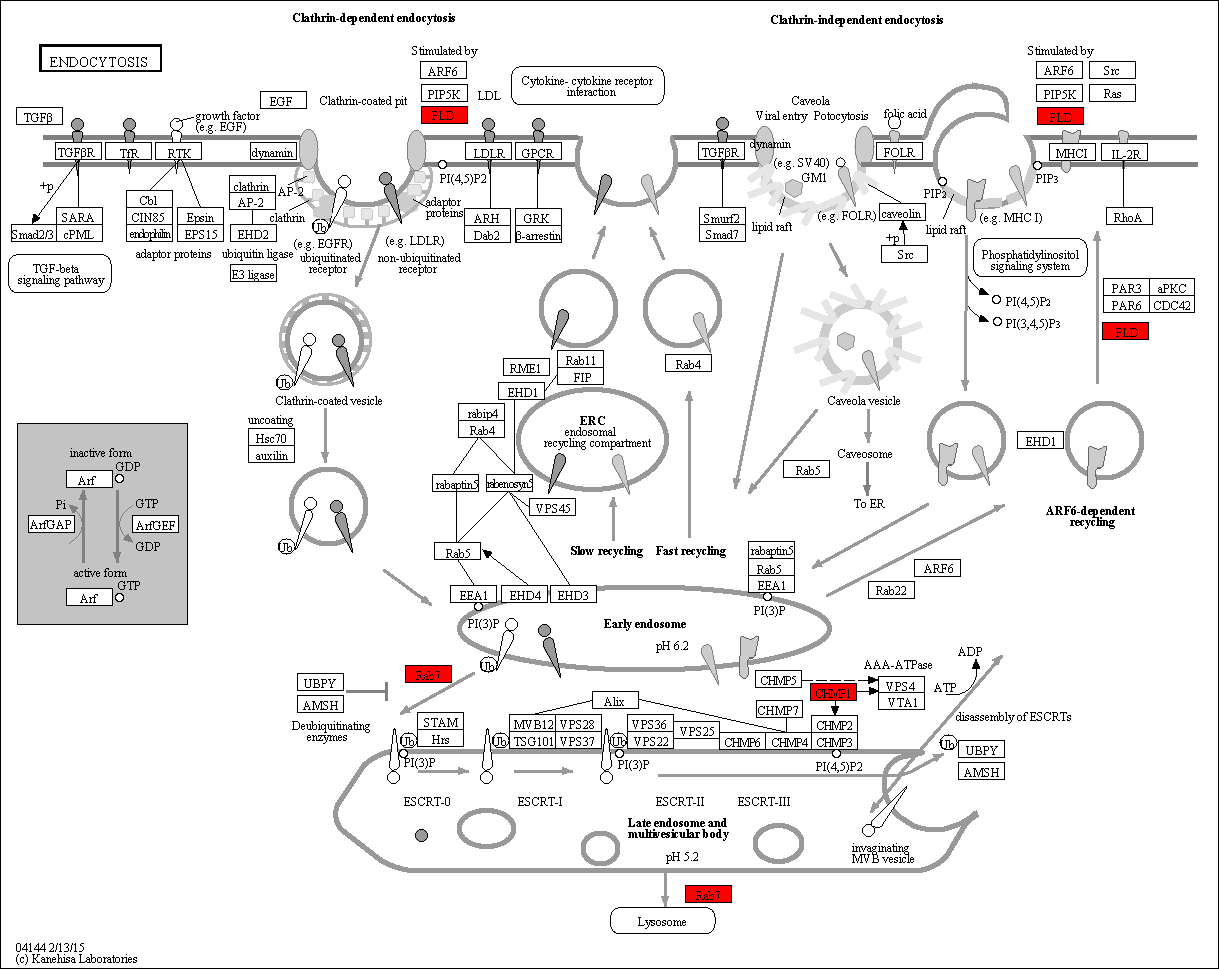

Supplement: S1 File — (ZIP) [file pone.0261403.s011.zip › ED/Anno/GeneAnno/pathway/kegg_map/ko04144.png]

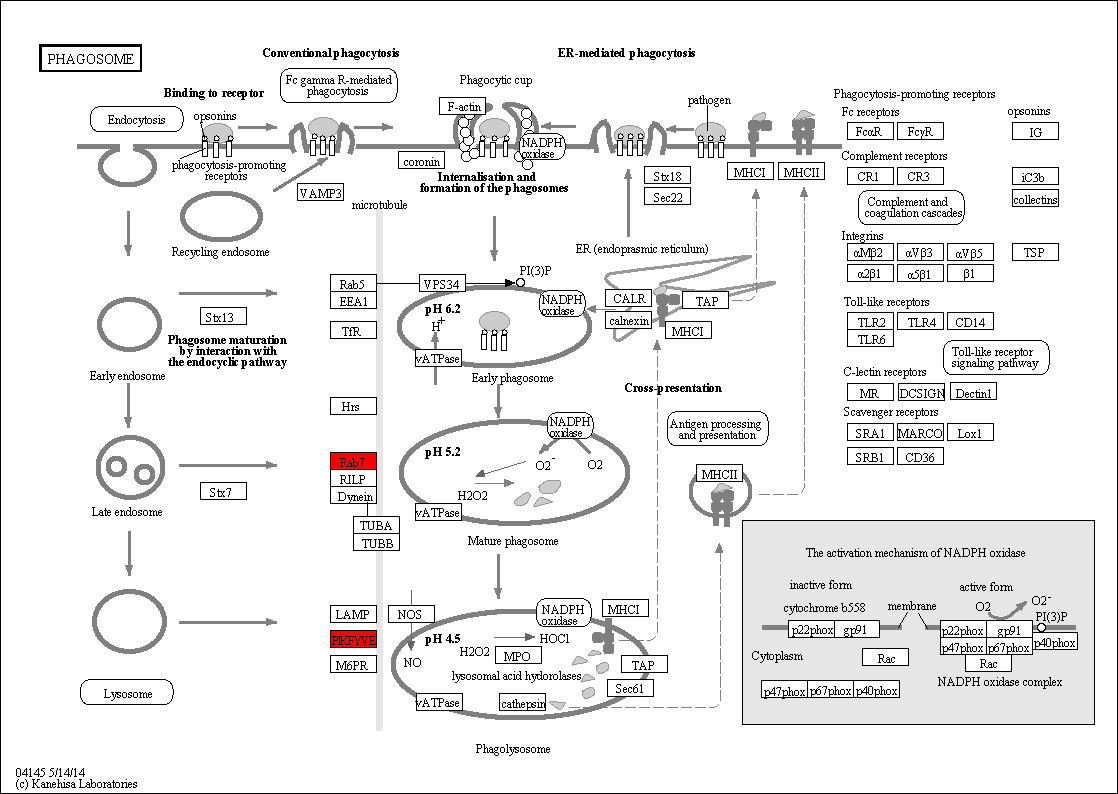

Supplement: S1 File — (ZIP) [file pone.0261403.s011.zip › ED/Anno/GeneAnno/pathway/kegg_map/ko04145.png]

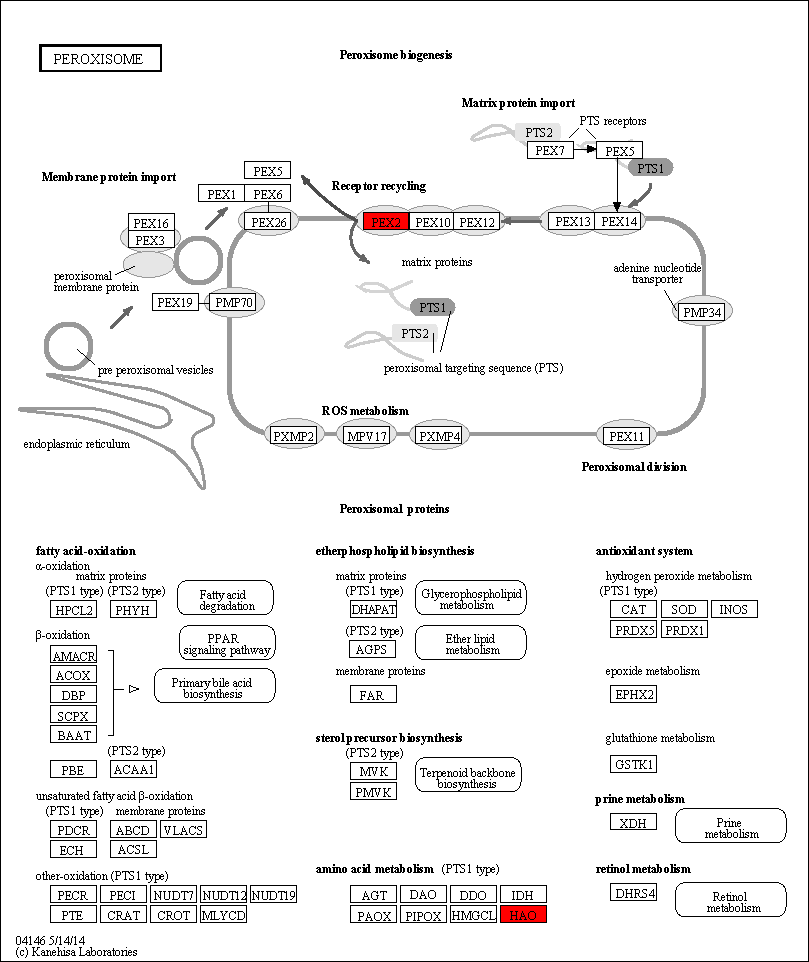

Supplement: S1 File — (ZIP) [file pone.0261403.s011.zip › ED/Anno/GeneAnno/pathway/kegg_map/ko04146.png]

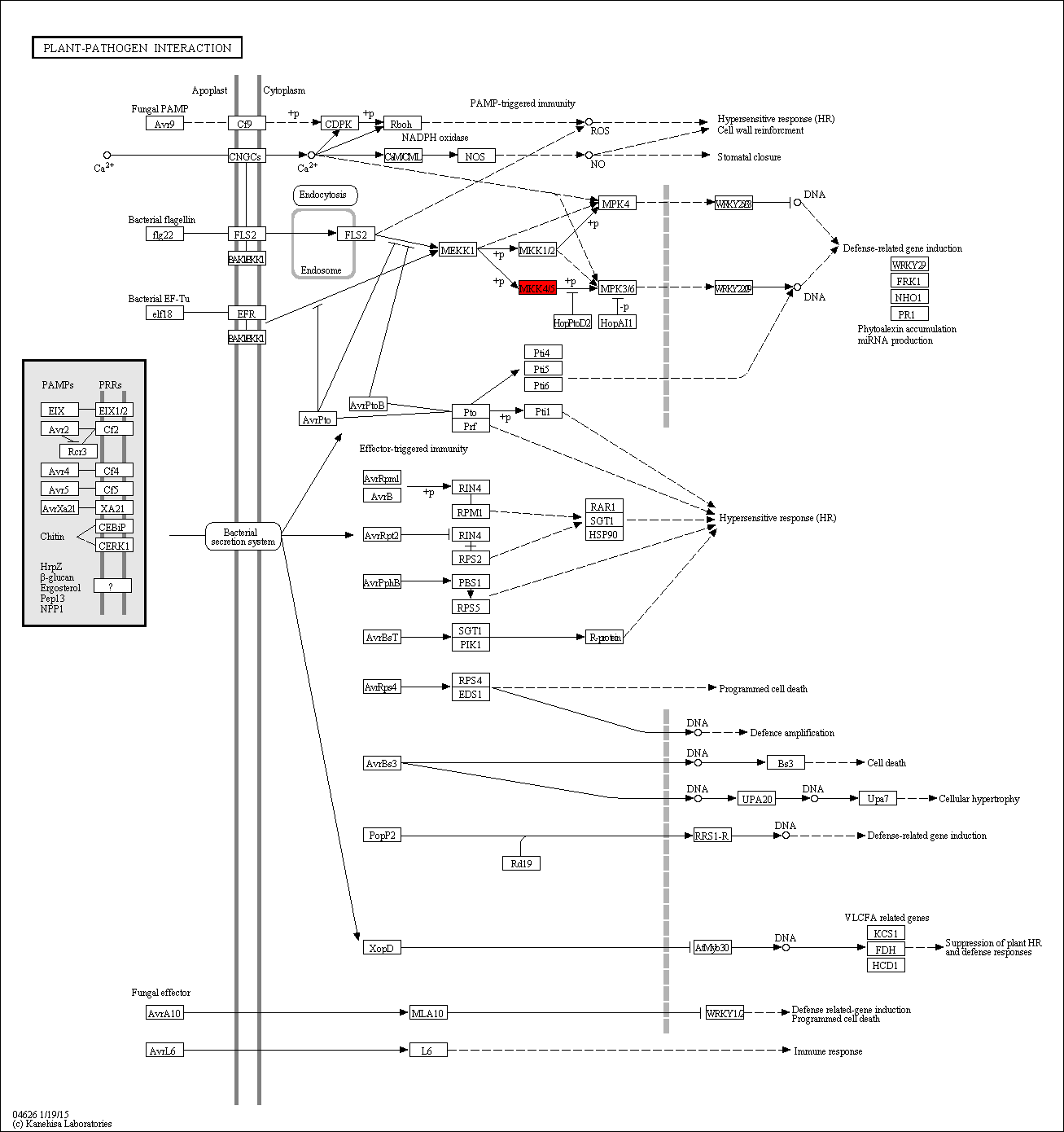

Supplement: S1 File — (ZIP) [file pone.0261403.s011.zip › ED/Anno/GeneAnno/pathway/kegg_map/ko04626.png]

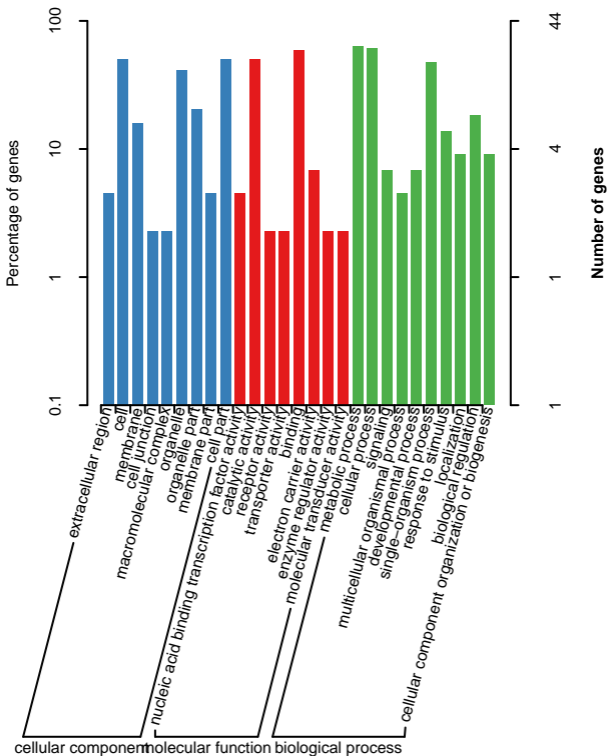

Supplement: S1 File — (ZIP) [file pone.0261403.s011.zip › ED/Anno/SNPAnno/GO_Anno/go_enrichment/Solanum_tuberosum_v4.03.GO.pdf]

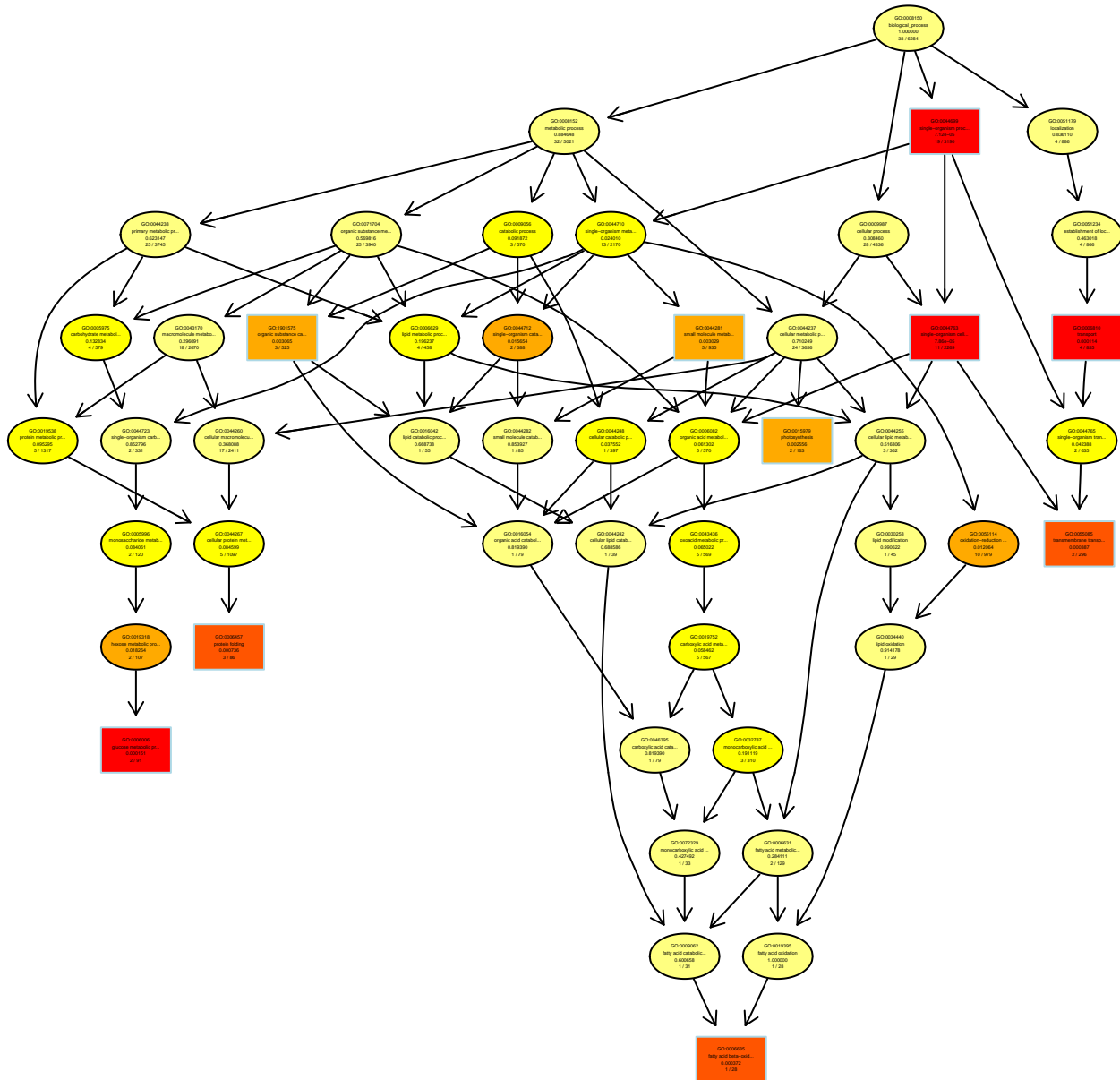

Supplement: S1 File — (ZIP) [file pone.0261403.s011.zip › ED/Anno/SNPAnno/GO_Anno/topGO/Solanum_tuberosum_v4.03.topGO_BP.pdf]

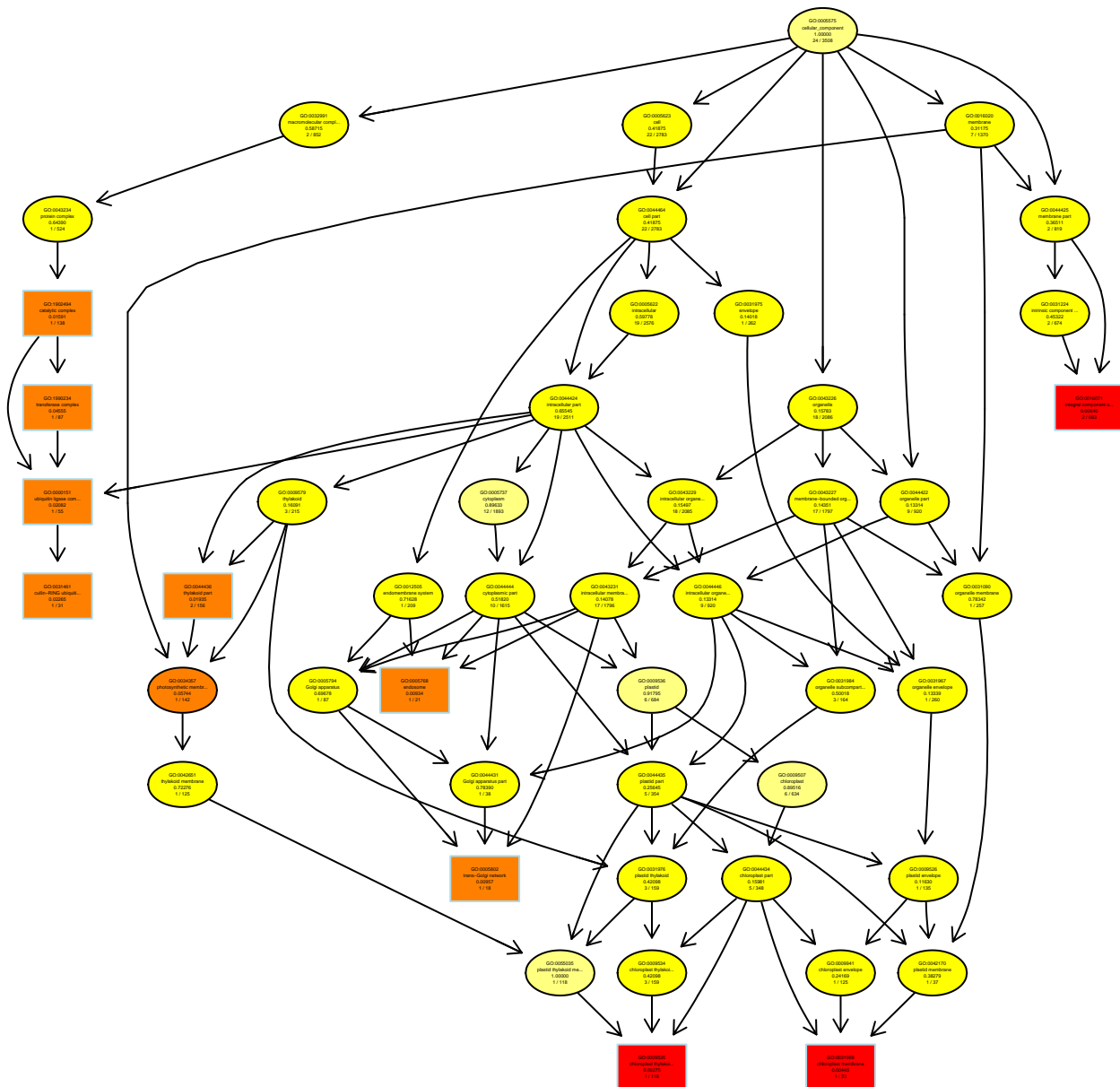

Supplement: S1 File — (ZIP) [file pone.0261403.s011.zip › ED/Anno/SNPAnno/GO_Anno/topGO/Solanum_tuberosum_v4.03.topGO_CC.pdf]

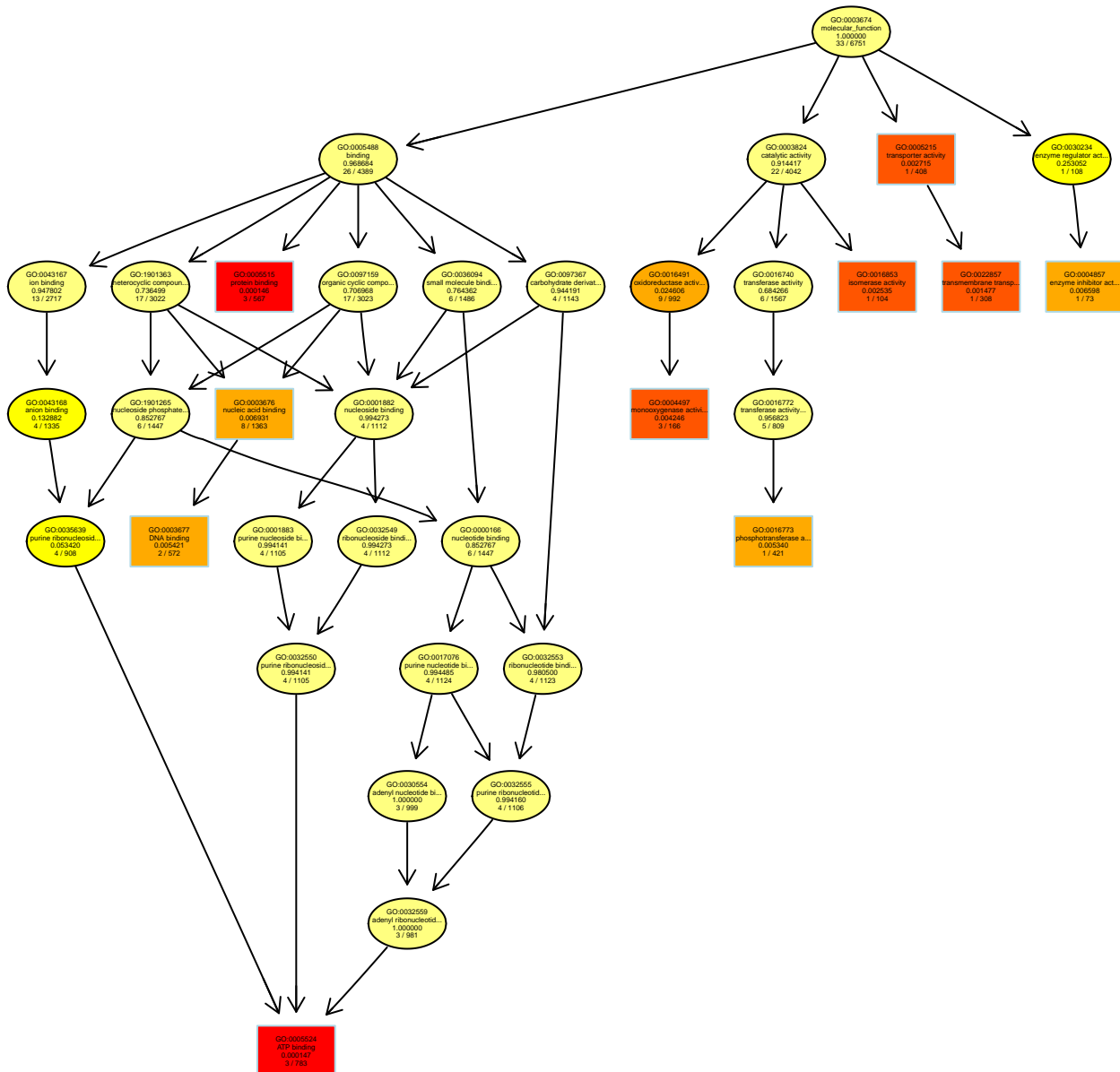

Supplement: S1 File — (ZIP) [file pone.0261403.s011.zip › ED/Anno/SNPAnno/GO_Anno/topGO/Solanum_tuberosum_v4.03.topGO_MF.pdf]

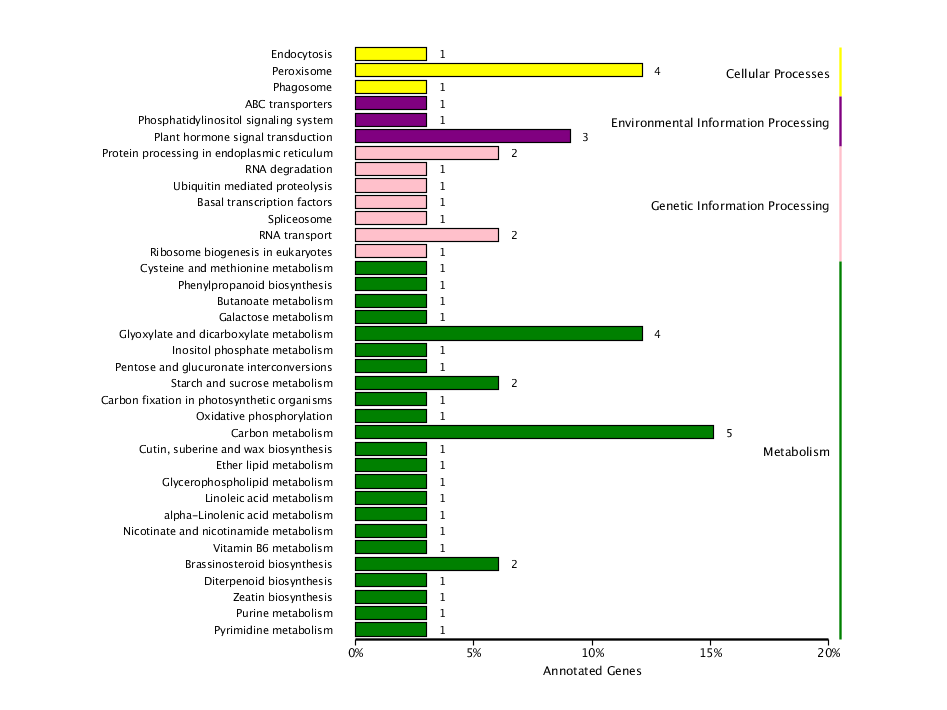

Supplement: S1 File — (ZIP) [file pone.0261403.s011.zip › ED/Anno/SNPAnno/pathway/kegg_enrichment/Solanum_tuberosum_v4.03.KEGG.png]

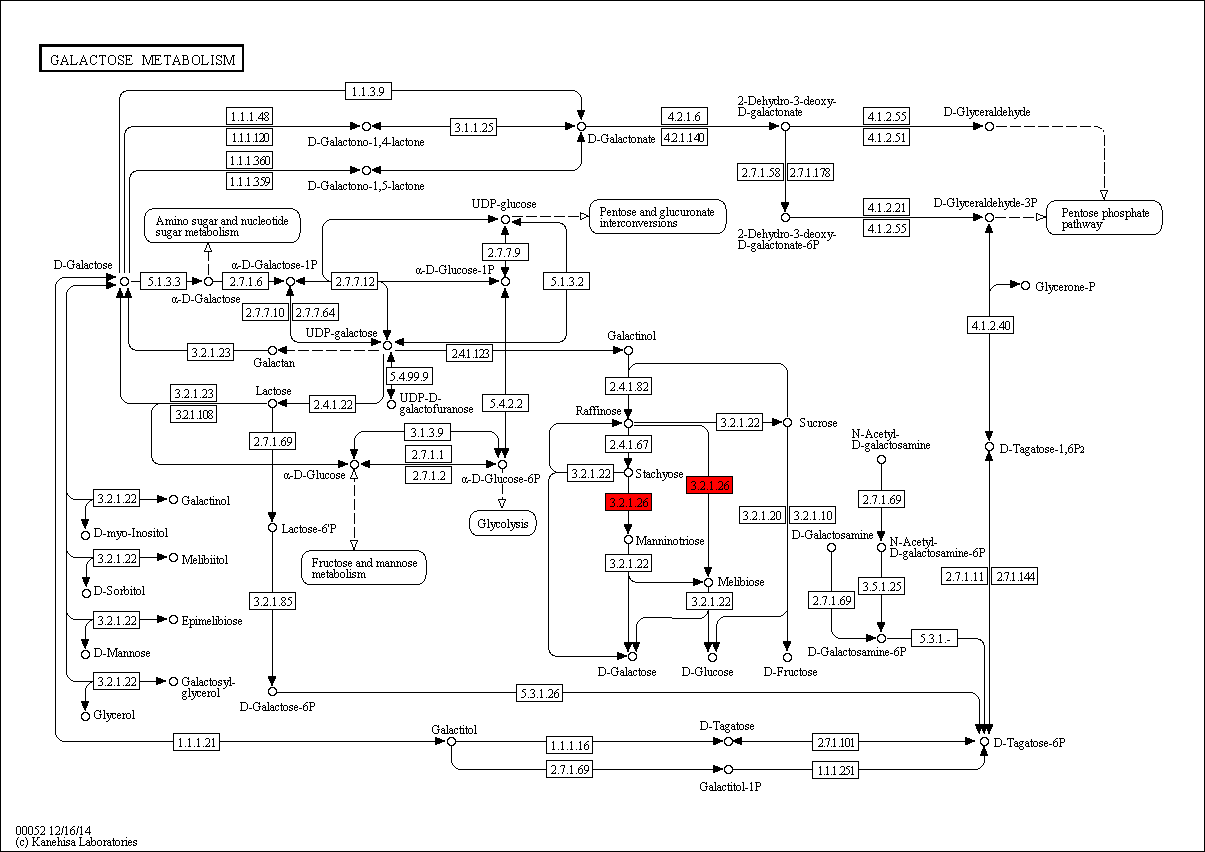

Supplement: S1 File — (ZIP) [file pone.0261403.s011.zip › ED/Anno/SNPAnno/pathway/kegg_map/ko00052.png]

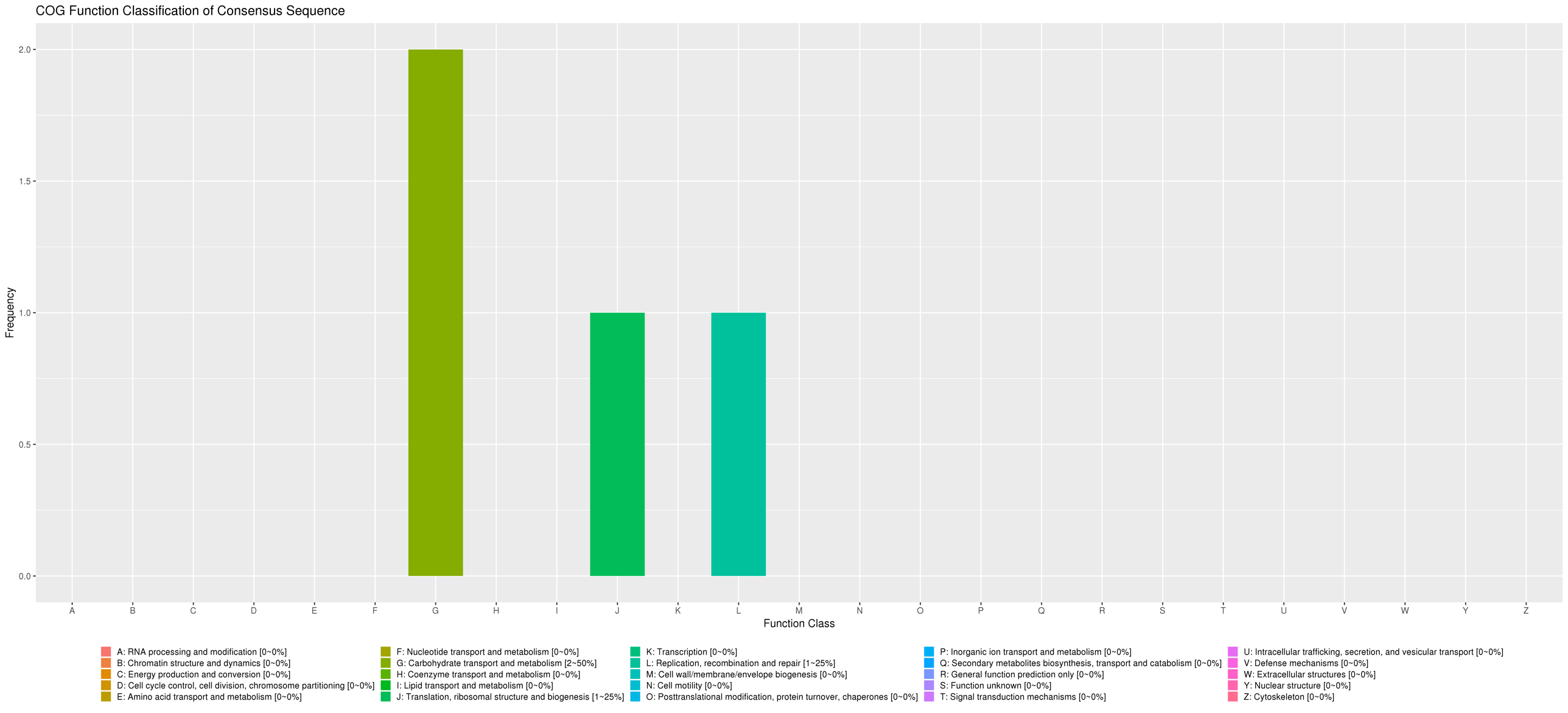

Supplement: S2 File — (ZIP) [file pone.0261403.s012.zip › merge_region/Anno/Cog_Anno/Solanum_tuberosum_v4.03.Cog.classfy.png]

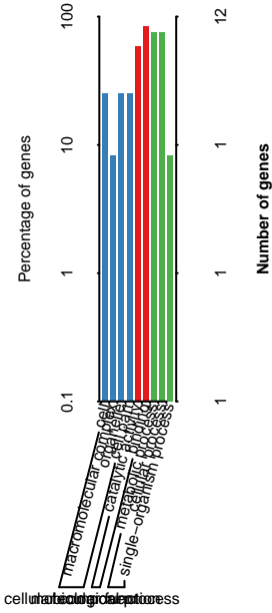

Supplement: S2 File — (ZIP) [file pone.0261403.s012.zip › merge_region/Anno/GO_Anno/go_enrichment/Solanum_tuberosum_v4.03.GO.pdf]

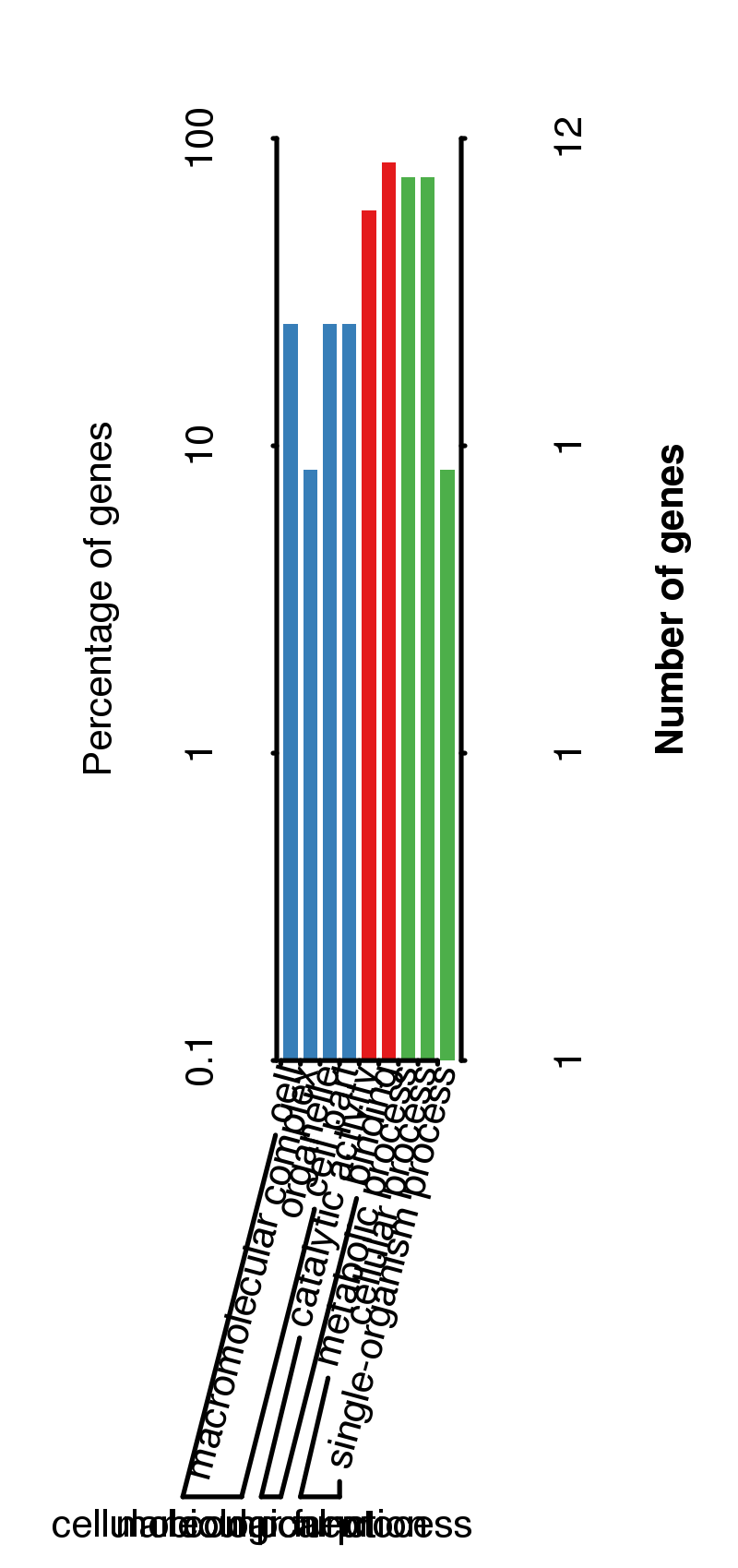

Supplement: S2 File — (ZIP) [file pone.0261403.s012.zip › merge_region/Anno/GO_Anno/go_enrichment/Solanum_tuberosum_v4.03.GO.png]

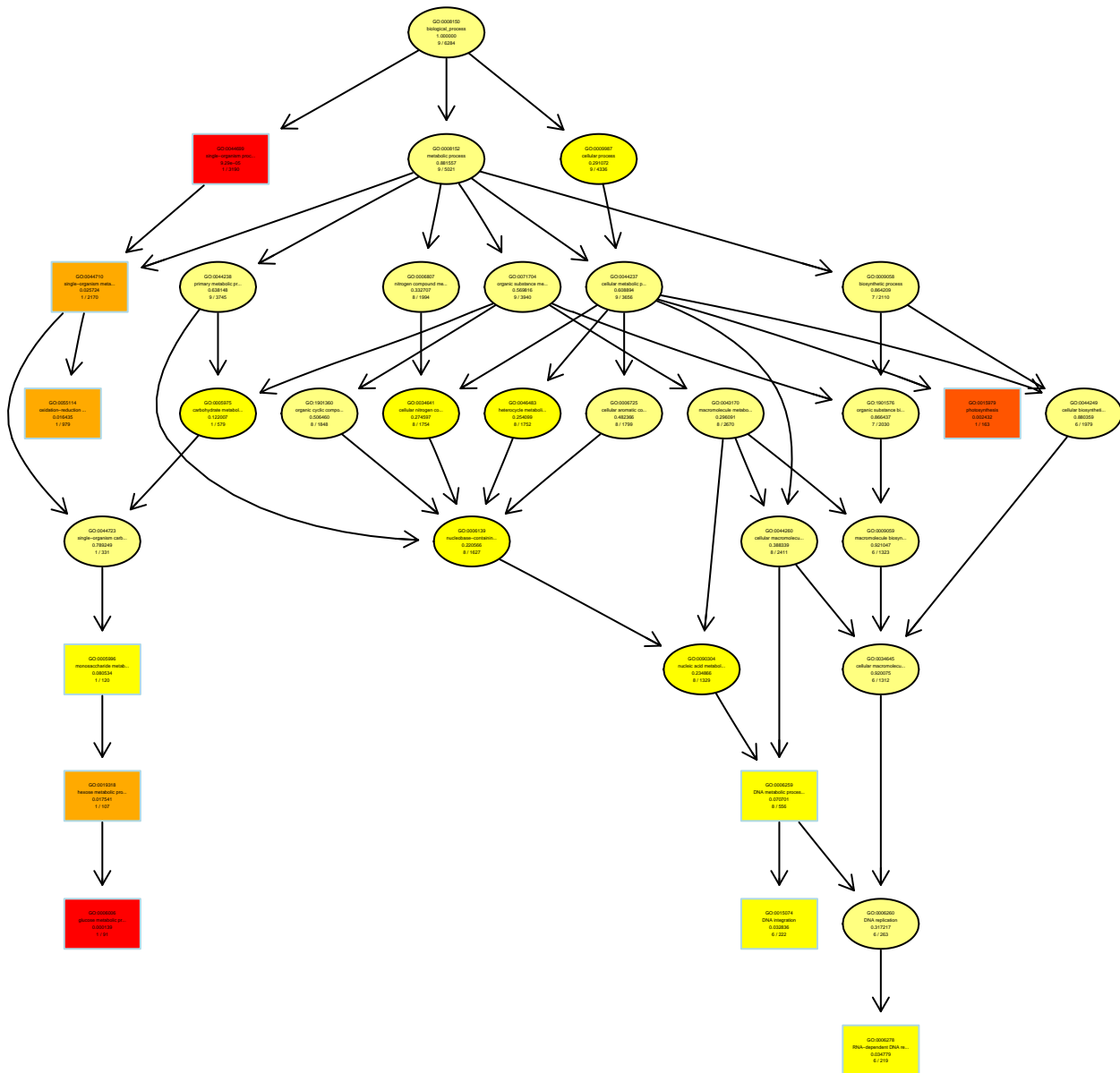

Supplement: S2 File — (ZIP) [file pone.0261403.s012.zip › merge_region/Anno/GO_Anno/topGO/Solanum_tuberosum_v4.03.topGO_BP.pdf]

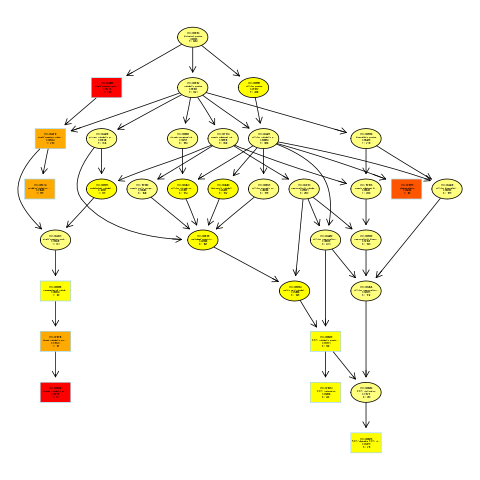

Supplement: S2 File — (ZIP) [file pone.0261403.s012.zip › merge_region/Anno/GO_Anno/topGO/Solanum_tuberosum_v4.03.topGO_BP.png]

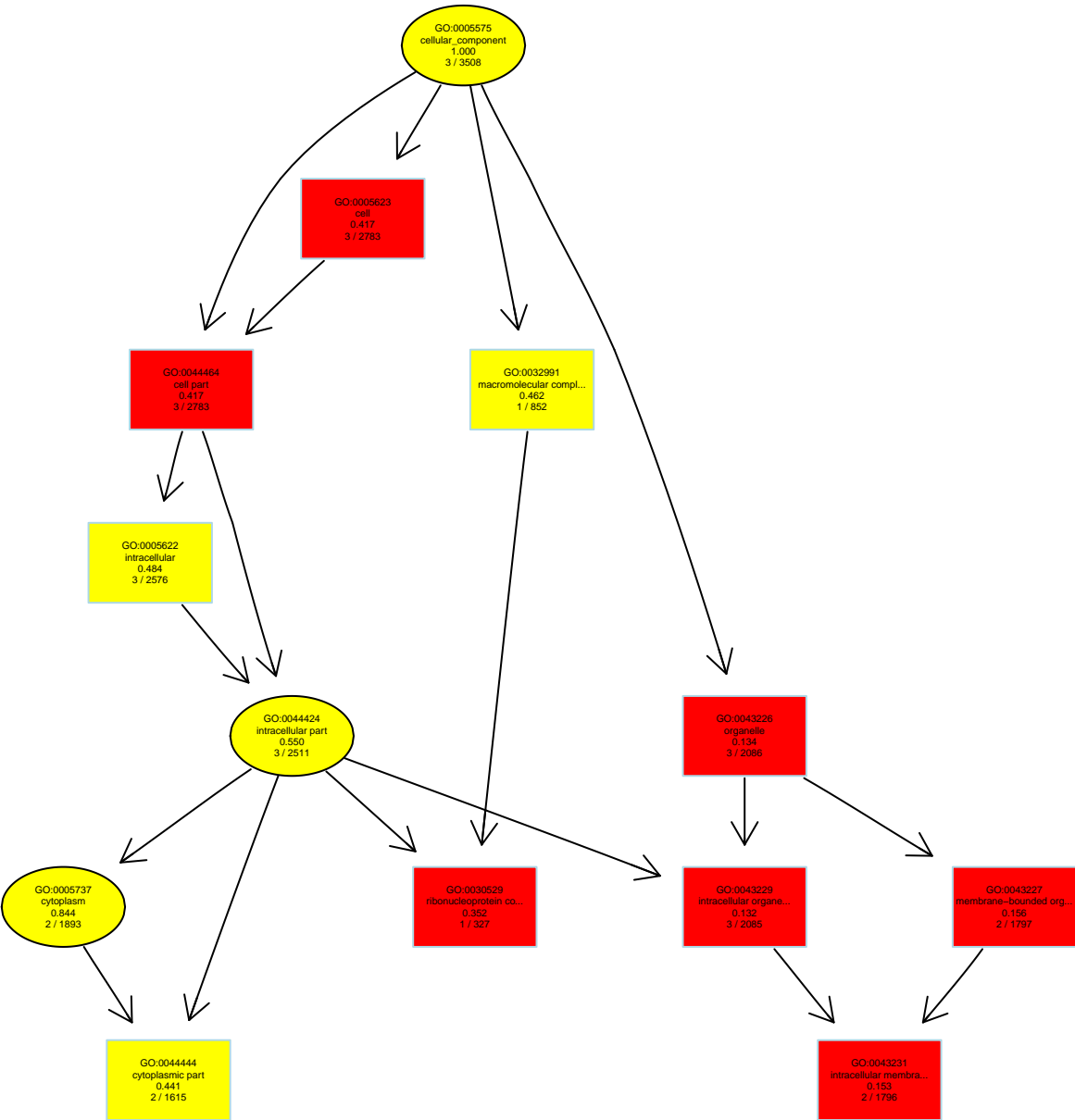

Supplement: S2 File — (ZIP) [file pone.0261403.s012.zip › merge_region/Anno/GO_Anno/topGO/Solanum_tuberosum_v4.03.topGO_CC.pdf]

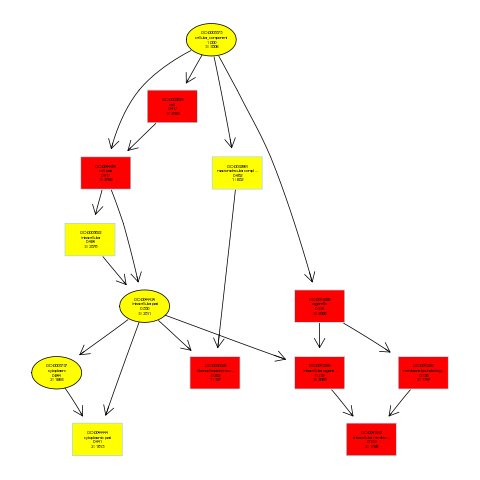

Supplement: S2 File — (ZIP) [file pone.0261403.s012.zip › merge_region/Anno/GO_Anno/topGO/Solanum_tuberosum_v4.03.topGO_CC.png]

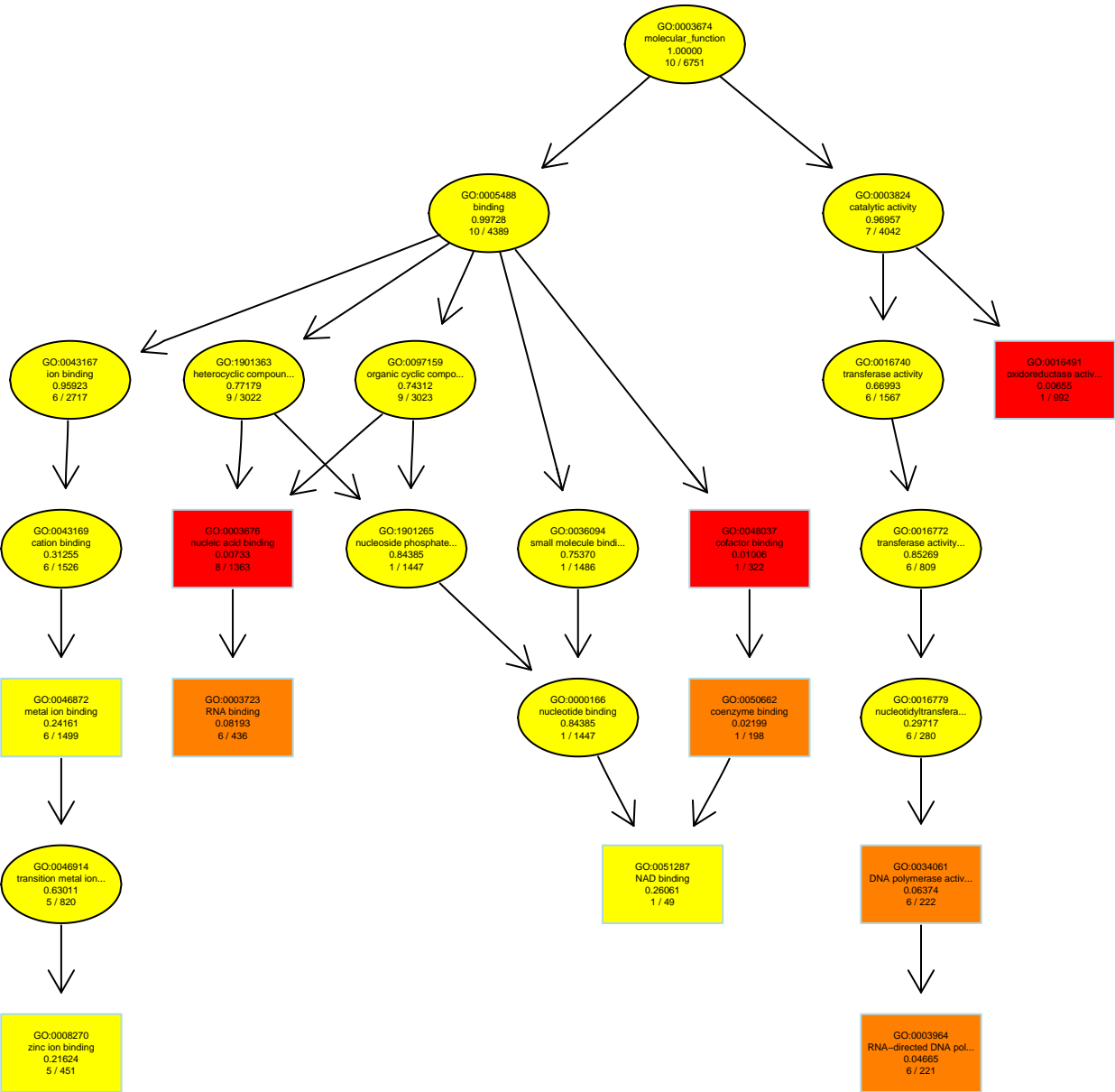

Supplement: S2 File — (ZIP) [file pone.0261403.s012.zip › merge_region/Anno/GO_Anno/topGO/Solanum_tuberosum_v4.03.topGO_MF.pdf]

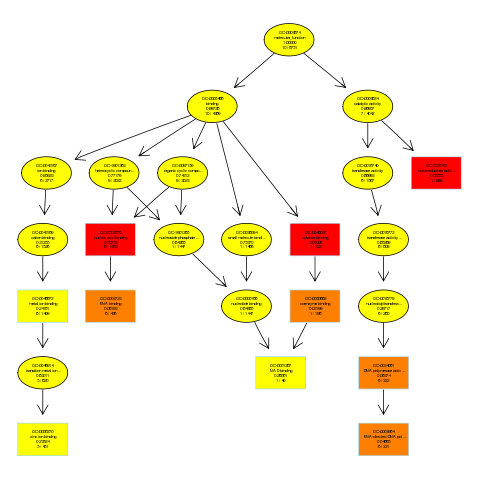

Supplement: S2 File — (ZIP) [file pone.0261403.s012.zip › merge_region/Anno/GO_Anno/topGO/Solanum_tuberosum_v4.03.topGO_MF.png]

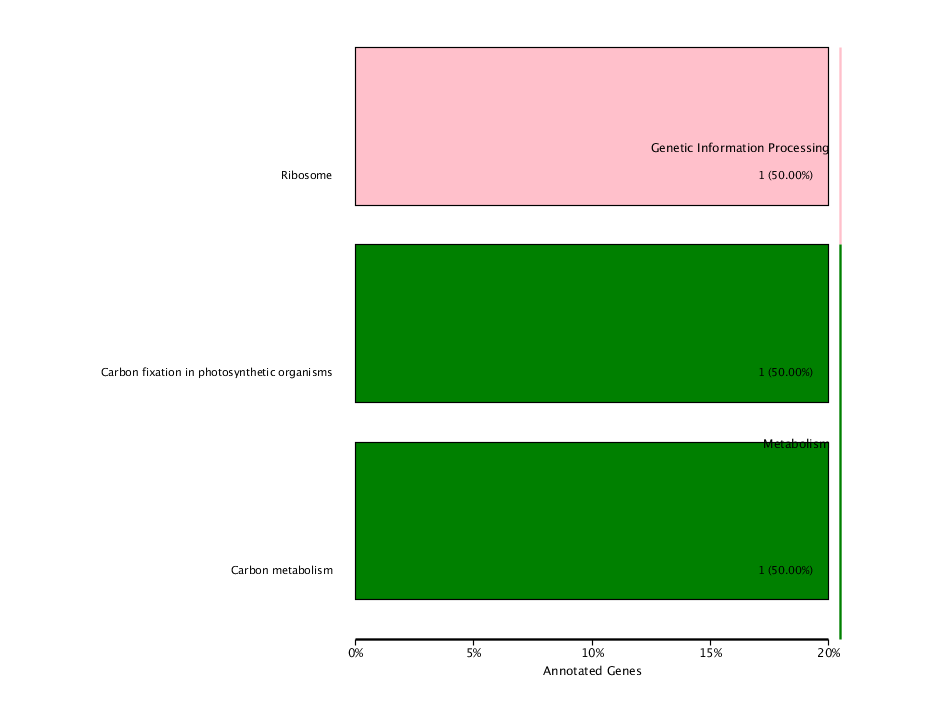

Supplement: S2 File — (ZIP) [file pone.0261403.s012.zip › merge_region/Anno/pathway/kegg_enrichment/Solanum_tuberosum_v4.03.KEGG.png]

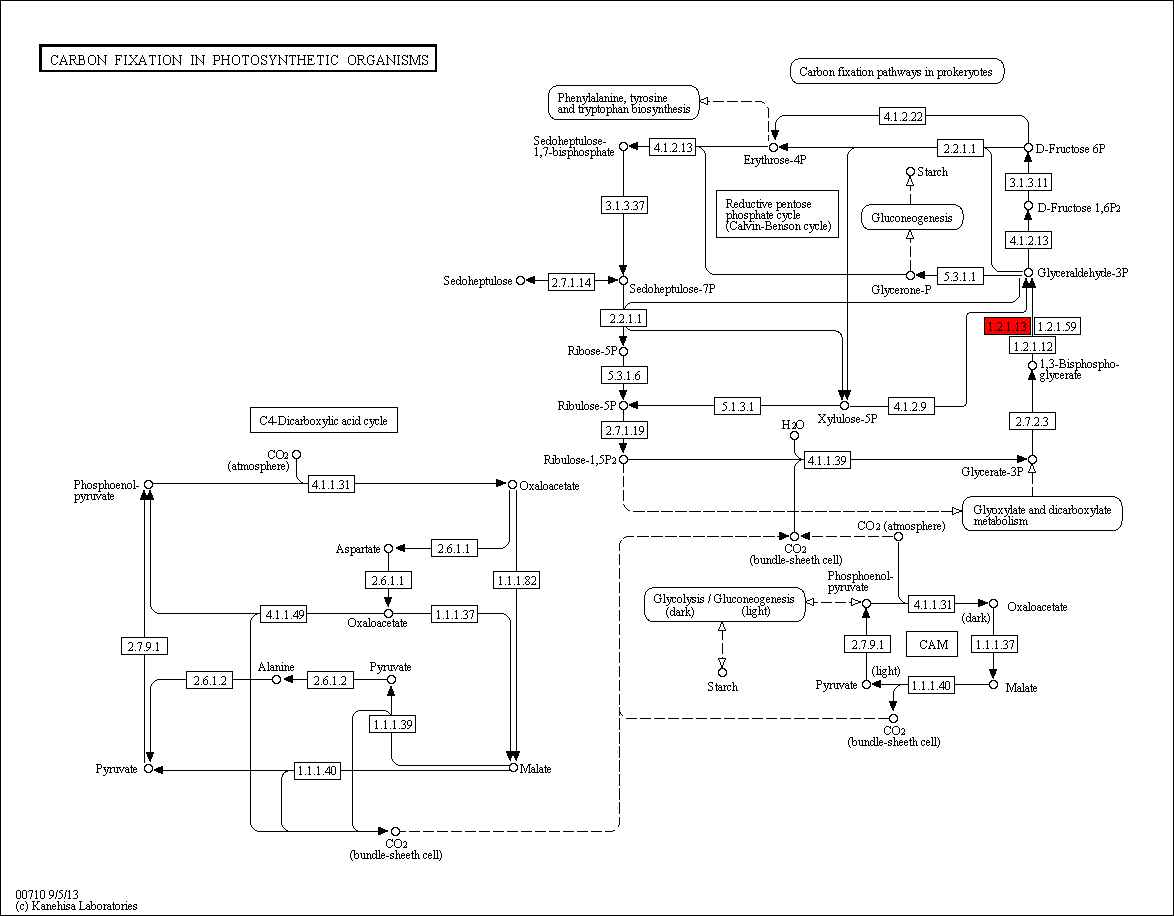

Supplement: S2 File — (ZIP) [file pone.0261403.s012.zip › merge_region/Anno/pathway/kegg_map/ko00710.png]

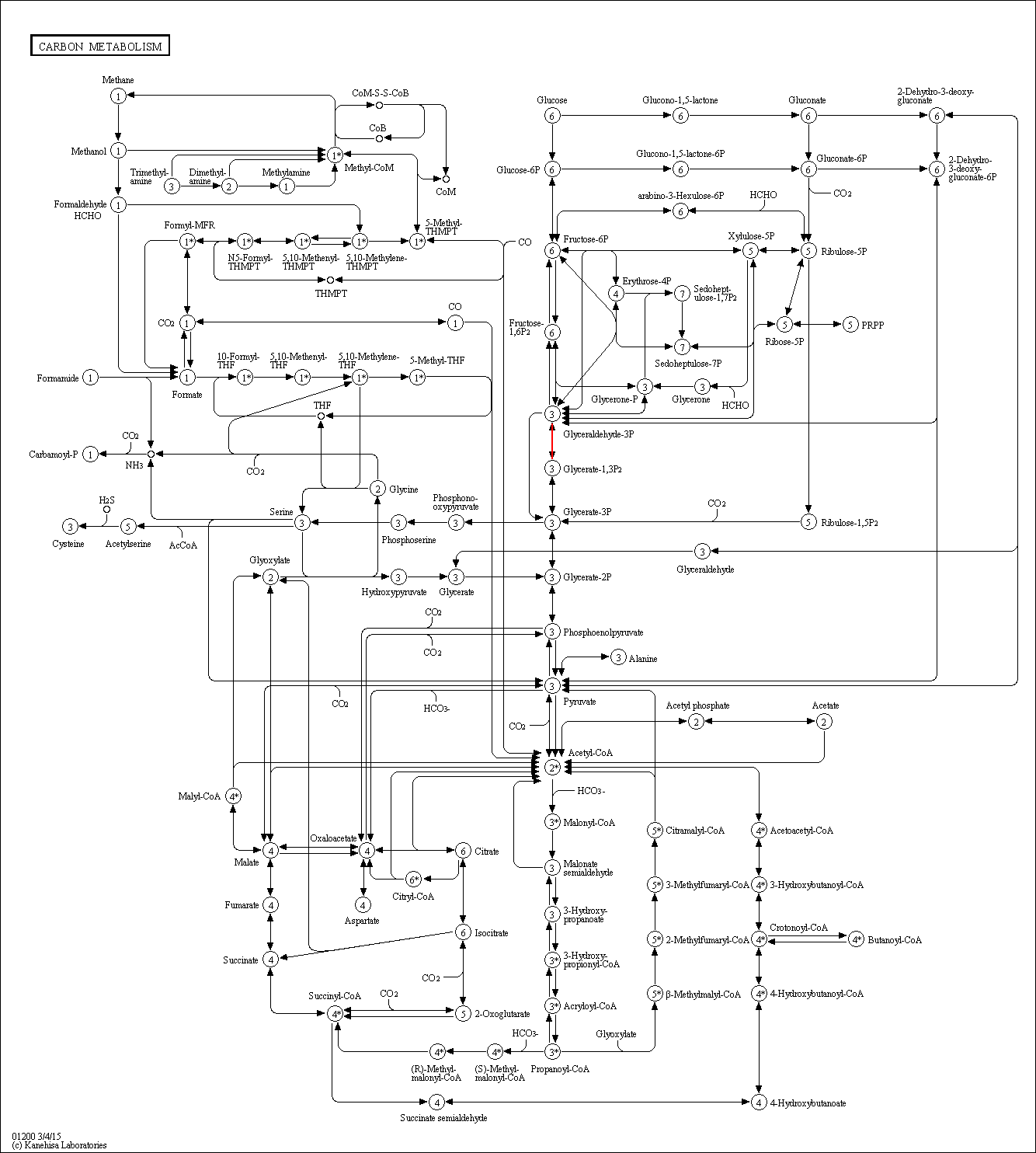

Supplement: S2 File — (ZIP) [file pone.0261403.s012.zip › merge_region/Anno/pathway/kegg_map/ko01200.png]

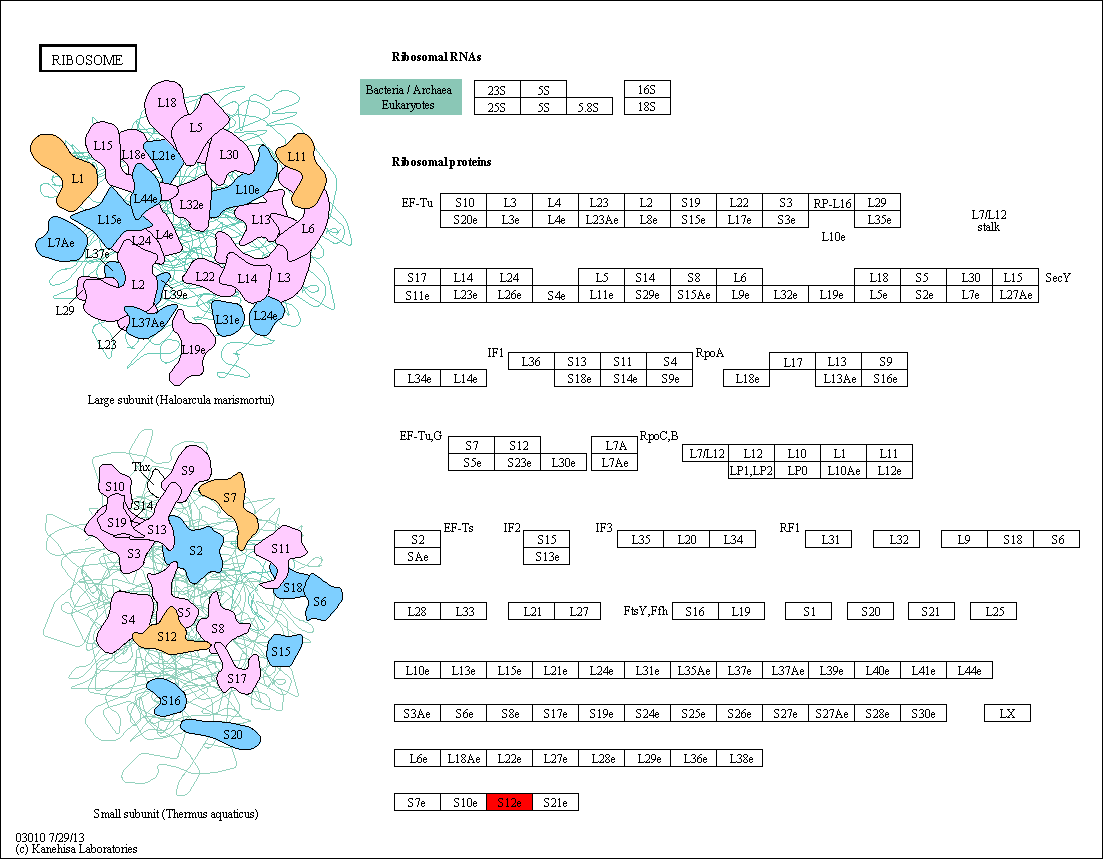

Supplement: S2 File — (ZIP) [file pone.0261403.s012.zip › merge_region/Anno/pathway/kegg_map/ko03010.png]

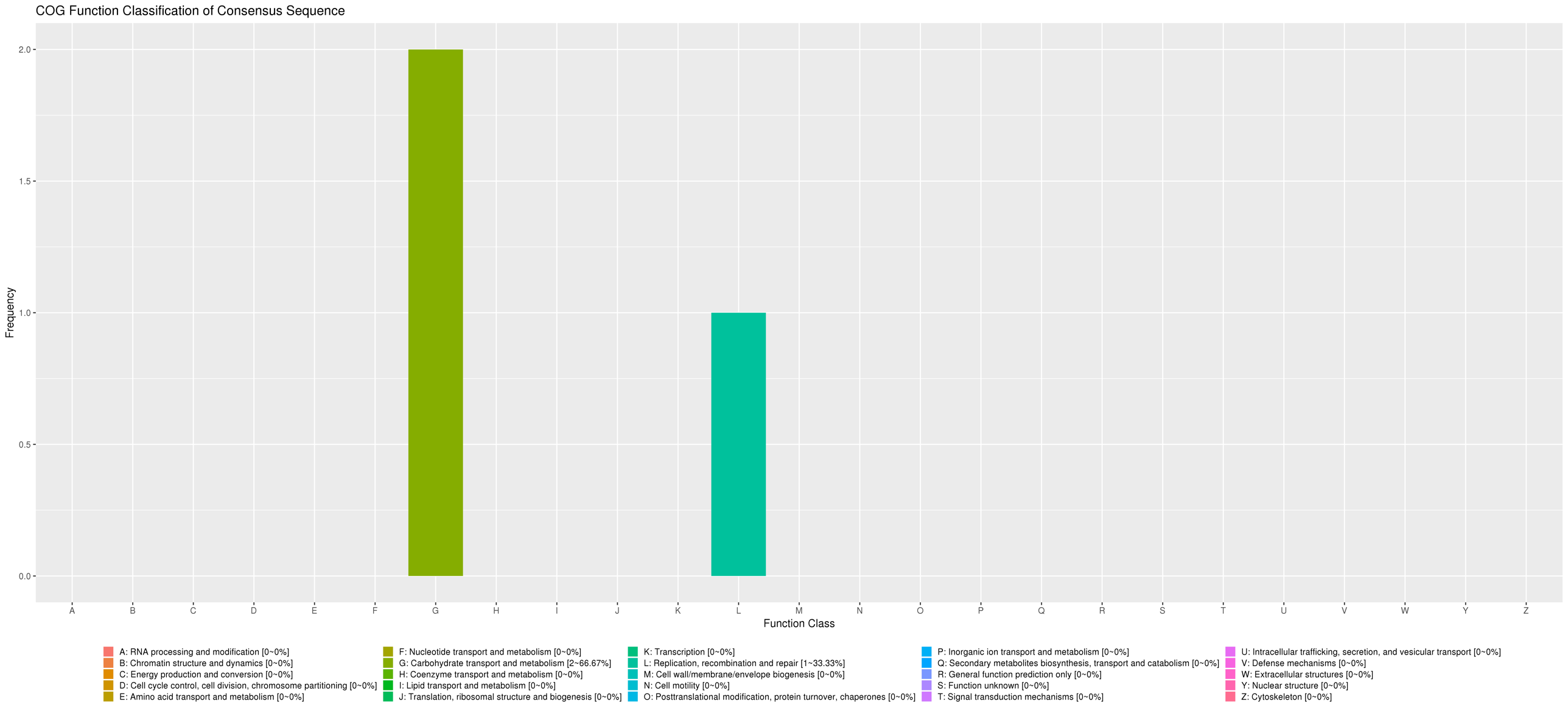

Supplement: S2 File — (ZIP) [file pone.0261403.s012.zip › merge_region/SNPAnno/Cog_Anno/Solanum_tuberosum_v4.03.Cog.classfy.png]

COG Function Classification of Consensus Sequence

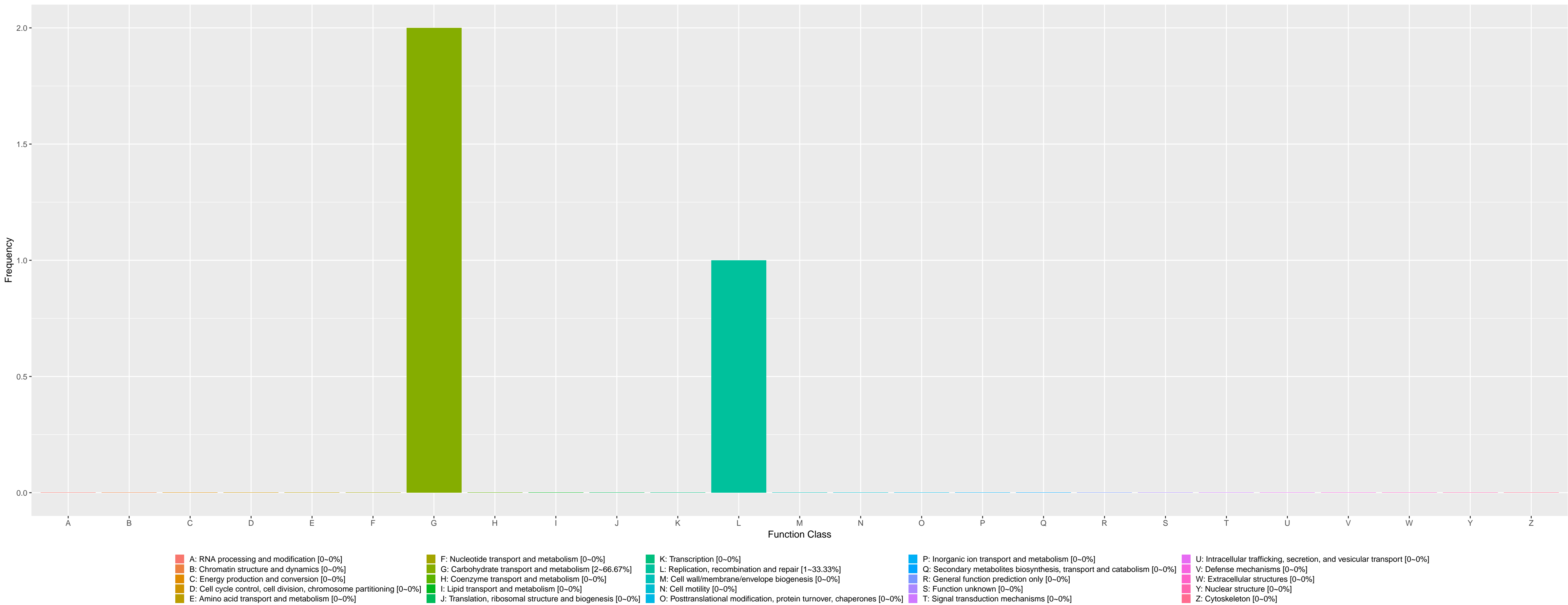

Supplement: S2 File — (ZIP) [file pone.0261403.s012.zip › merge_region/SNPAnno/Cog_Anno/Solanum_tuberosum_v4.03.Cog.classfy.png.pdf]

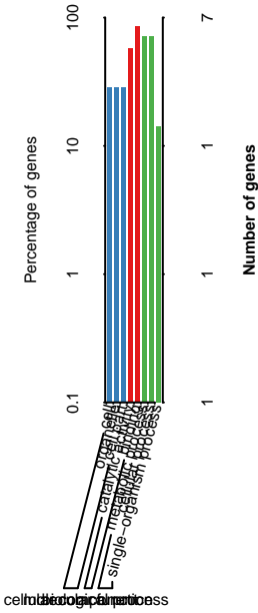

Supplement: S2 File — (ZIP) [file pone.0261403.s012.zip › merge_region/SNPAnno/GO_Anno/go_enrichment/Solanum_tuberosum_v4.03.GO.pdf]

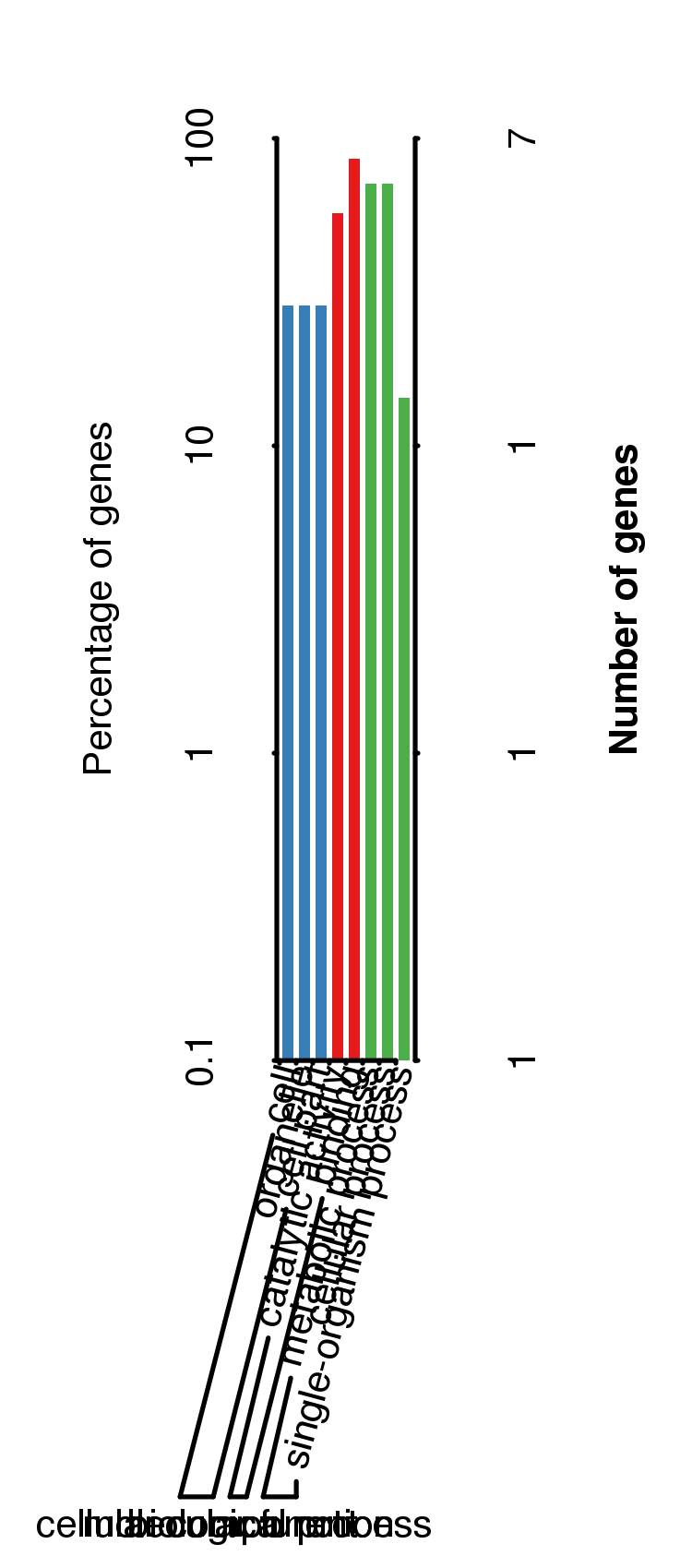

Supplement: S2 File — (ZIP) [file pone.0261403.s012.zip › merge_region/SNPAnno/GO_Anno/go_enrichment/Solanum_tuberosum_v4.03.GO.png]
