# Supplementary material for: Identification of starch candidate genes using SLAF-seq and BSA strategies and development of related SNP-CAPS markers in tetraploid potato
Source: PLoS One. 2021 Dec 21;16(12):e0261403. doi: 10.1371/journal.pone.0261403 (PMC8691606; doi:10.1371/journal.pone.0261403)
Supplement: S1 Table — (DOCX) [file pone.0261403.s009.docx]

**S1 Table. CAPS primers and restriction endonuclease.**

| **CAPS**  **Primers** | **Forward**  **primer (5'-3')** | **Reverse**  **primer (5'-3')** | **Anneal temperature**  **(℃)** | **Size of fragment (bp)** | **SNP loci** | **Restriction enzyme** |
| --- | --- | --- | --- | --- | --- | --- |
| chr2-CAPS1 | 5' GTACCCTATCTCCTTTCAATCT 3' | 5' CCTTCCCATCACCGACTT 3' | 56℃ | 550bp | T/A | ScaI |
| chr2-CAPS2 | 5' GGGTTGAATTTGGAAGTAGGG 3' | 5' GGTGAGGTAAGTGGCGTAGA 3' | 58℃ | 673bp | G/A | ClaI |
| chr2-CAPS3 | 5' GAACTAAATCTGGTCCTATCA 3' | 5' GGTTGCCAAGGTCTCGTA 3' | 56℃ | 502bp | A/G | NcoI |
| chr2-CAPS4 | 5' TCATCTGCGGTGGTAGGC 3' | 5' TGACACGAGGAGGGTTGC 3' | 60℃ | 567bp | C/A | MseI |
| chr2-CAPS5 | 5' AGGGAGAAGGCGGAGTTG 3' | 5' TAGCCGTGAGGGTAGGAC 3' | 58℃ | 664bp | A/T | DraI |
| chr2-CAPS6 | 5' TTGGACTTGTGCCTTTGG 3' | 5' ATCATTCTACGGGTTTGC 3' | 58℃ | 517bp | A/G | ApaLI |
| chr2-CAPS7 | 5' CATCGCCCTCACATTCGT 3' | 5' CGGCTTCCCTTGGTTCTA 3' | 56℃ | 441bp | T/C | NheI |
| chr2-CAPS8 | 5' CCCTTGGTTGGGTTACTT 3' | 5' CATTCCCTTTCACGGTCT 3' | 60℃ | 419bp | A/C | BstUI |
| chr2-CAPS9 | 5' TTAGGTTATCGTGGCAAAT 3' | 5' GAAGATGAATAAGAGGGCTA 3' | 58℃ | 418bp | G/A | MseI |
| chr2-CAPS10 | 5' GCCCAAAGTTCCAATAGC 3' | 5' TTTCGTAACAACCCAAGAC 3' | 58℃ | 610bp | T/C | BstUI |
| chr2-CAPS11 | 5' CGGAAGTTCTATCCTCTGA 3' | 5' TAAGGCTAATGTCGTTGC 3' | 58℃ | 538bp | G/A | XbaI |
| chr2-CAPS12 | 5' ATGCCAGAATGTTGAATGAG 3' | 5' AAATGGGTAAGTTATGGAGGT 3' | 56℃ | 707bp | A/G | BstUI |
| chr2-CAPS13 | 5' GGGACATGAGGAACGACA 3' | 5' CAGAACGACGCCAACACT 3' | 58℃ | 405bp | C/T | HpaI |
| chr2-CAPS14 | 5' TTCTCCTTTCGTCTATGTCC 3' | 5' CCAGCAGGCACTACTCTT 3' | 60℃ | 682bp | A/G | BstUI |
| chr2-CAPS15 | 5' GGGGCAAGAAGGTGAAAT 3' | 5' AAGTTGGAGCGTGAGCAT 3' | 58℃ | 588bp | C/A | MlucI |
| chr2-CAPS16 | 5' CGGATGTCGGTATAAGAGG 3' | 5' GGTCCCACAAGTTCCAGTT 3' | 58℃ | 625bp | T/C | HinP1I |
| chr2-CAPS17 | 5' CACCTATCGTGCCTACTTT 3' | 5' AACTTCATTCACTTTCCCTC 3' | 58℃ | 468bp | C/T | MboI |
| chr2-CAPS18 | 5' ACTGATGAAGCCAAACCA 3' | 5' TTGTAAGCCCAAATAGCG 3' | 58℃ | 624bp | G/A | HpaI |
| chr2-CAPS19 | 5' GCGGCGAGGTATTGGTTC 3' | 5' AAGTTGGAGCGTGAGCAT 3' | 58℃ | 388bp | C/A | MlucI |
| chr2-CAPS20 | 5' GAGCAAGATAGTTGGTGGTG 3' | 5' TCGTAGATTGGTGGAGGA 3' | 56℃ | 599bp | T/C | HpaI |
| chr2-CAPS21 | 5' GCTCTGGTCCTCTGTCTGC 3' | 5' TGTGGTGGGCTATTGTGC 3' | 58℃ | 387bp | A/C | BspHI |
| chr2-CAPS22 | 5' GCATTATCGGAGTGGGTA 3' | 5' CACAGGCAGACAGAGGAC 3' | 56℃ | 420bp | T/A | BglII |
| chr2-CAPS23 | 5' GGAACATCCCACAAAGCC 3' | 5' GCCATCGGAAGTGAGACC 3' | 56℃ | 315bp | T/C | AflII |
| chr2-CAPS24 | 5' ATGATAGAACCAAGGGAAGC 3' | 5' TGAGCCAGAGGATGAGAAA 3' | 54℃ | 729bp | A/G | HpaII |
| chr2-CAPS25 | 5' ACCTCCATAACTTACCCATTTC 3' | 5' TTTCGCTTCCATCCTCTTT 3' | 54℃ | 458bp | C/A | BstUI |

**S1 Table. CAPS primers and restriction endonuclease.** (Continued)

| **CAPS Primers** | **Forward**  **primer (5'-3')** | **Reverse**  **primer (5'-3')** | **Anneal temperature**  **(℃)** | **Size of fragment**  **(bp)** | **SNP loci** | **Restriction enzyme** |
| --- | --- | --- | --- | --- | --- | --- |
| chr2-CAPS26 | 5' ACTGATGAAGCCAAACCA 3' | 5' TTGTAAGCCCAAATAGCG 3' | 56℃ | 624bp | G/A | HpaI |
| chr2-CAPS27 | 5' AATCTGTCCCACAAAGTCC 3' | 5' ACAAGGCAAGCCAAGTAA 3' | 56℃ | 755bp | C/T | PsiI |
| chr2-CAPS28 | 5' TTCTCCTTTCGTCTATGTCC 3' | 5' CCAGCAGGCACTACTCTT 3' | 60℃ | 682bp | A/G | SelI |
| chr2-CAPS29 | 5' CGGAAGTTCTATCCTCTGA 3' | 5' TAAGGCTAATGTCGTTGC 3' | 58℃ | 538bp | G/A | BfaI |
| chr2-CAPS30 | 5' ATGGGAACTGCAATAACC 3' | 5' CACTACCAGGCAAAGAGG 3' | 54℃ | 404bp | C/T | PsiI |
| chr2-CAPS31 | 5' ATGGGAACTGCAATAACC 3' | 5' CACTACCAGGCAAAGAGG 3' | 54℃ | 404bp | C/T | BglII |
| chr2-CAPS32 | 5' GGTTTGCCATAAAGAGGT 3' | 5' TGTGGGCTGGTTAGTGAG 3' | 56℃ | 555bp | T/A | MaeII |
| chr2-CAPS33 | 5' GGTTTGCCATAAAGAGGT 3' | 5' TGTGGGCTGGTTAGTGAG 3' | 56℃ | 555bp | T/A | TaiI |
| chr2-CAPS34 | 5' ATGCCAGAATGTTGAATGAG 3' | 5' AAATGGGTAAGTTATGGAGGT 3' | 56℃ | 707bp | A/G | MluI |
| chr2-CAPS35 | 5' ATGCCAGAATGTTGAATGAG 3' | 5' AAATGGGTAAGTTATGGAGGT 3' | 56℃ | 707bp | A/G | SelI |
| chr2-CAPS36 | 5' TTAGGTTATCGTGGCAAAT 3' | 5' GAAGATGAATAAGAGGGCTA 3' | 58℃ | 418bp | G/A | AseI |
| chr2-CAPS37 | 5' CCTTGGTTGGGTTACTTT 3' | 5' CATTCCCTTTCACGGTCT 3' | 54℃ | 418bp | A/C | BglII |
| chr2-CAPS38 | 5' CCCTTGGTTGGGTTACTT 3' | 5' CATTCCCTTTCACGGTCT 3' | 56℃ | 419bp | C/T | XhoI |
| chr2-CAPS39 | 5' CCCTTGGTTGGGTTACTT 3' | 5' CATTCCCTTTCACGGTCT 3' | 54℃ | 419bp | A/G | HinP1I |
| chr2-CAPS40 | 5' CCCTTGGTTGGGTTACTT 3' | 5' CATTCCCTTTCACGGTCT 3' | 54℃ | 419bp | T/G | HhaI |
| chr2-CAPS41 | 5' CCCTTGGTTGGGTTACTT 3' | 5' CATTCCCTTTCACGGTCT 3' | 54℃ | 419bp | T/G | BsrGI |
| chr2-CAPS42 | 5' CCATCCCATCACCGACAT 3' | 5' AACCGGAGCAGGAACCAT 3' | 58℃ | 556bp | G/A | MscI |
| chr2-CAPS43 | 5' GGGTTGAATTTGGAAGTAGGG 3' | 5' GGTGAGGTAAGTGGCGTAGA 3' | 54℃ | 673bp | C/T | PciI |
| chr2-CAPS44 | 5' CACATCTTTCCTCGGCTCA 3' | 5' TGGTTTGGCTTCATCAGTAG 3' | 58℃ | 741bp | T/C | AluI |
| chr2-CAPS45 | 5' CCTGATTGAGTGGAGGTCG 3' | 5' GCCAAGTTTAAGCCGGTAG 3' | 58℃ | 757bp | G/A | NdeI |
