# Supplementary material for: Identification of starch candidate genes using SLAF-seq and BSA strategies and development of related SNP-CAPS markers in tetraploid potato
Source: PLoS One. 2021 Dec 21;16(12):e0261403. doi: 10.1371/journal.pone.0261403 (PMC8691606; doi:10.1371/journal.pone.0261403)
Supplement: S1 Raw images — (PDF) [file pone.0261403.s010.pdf]

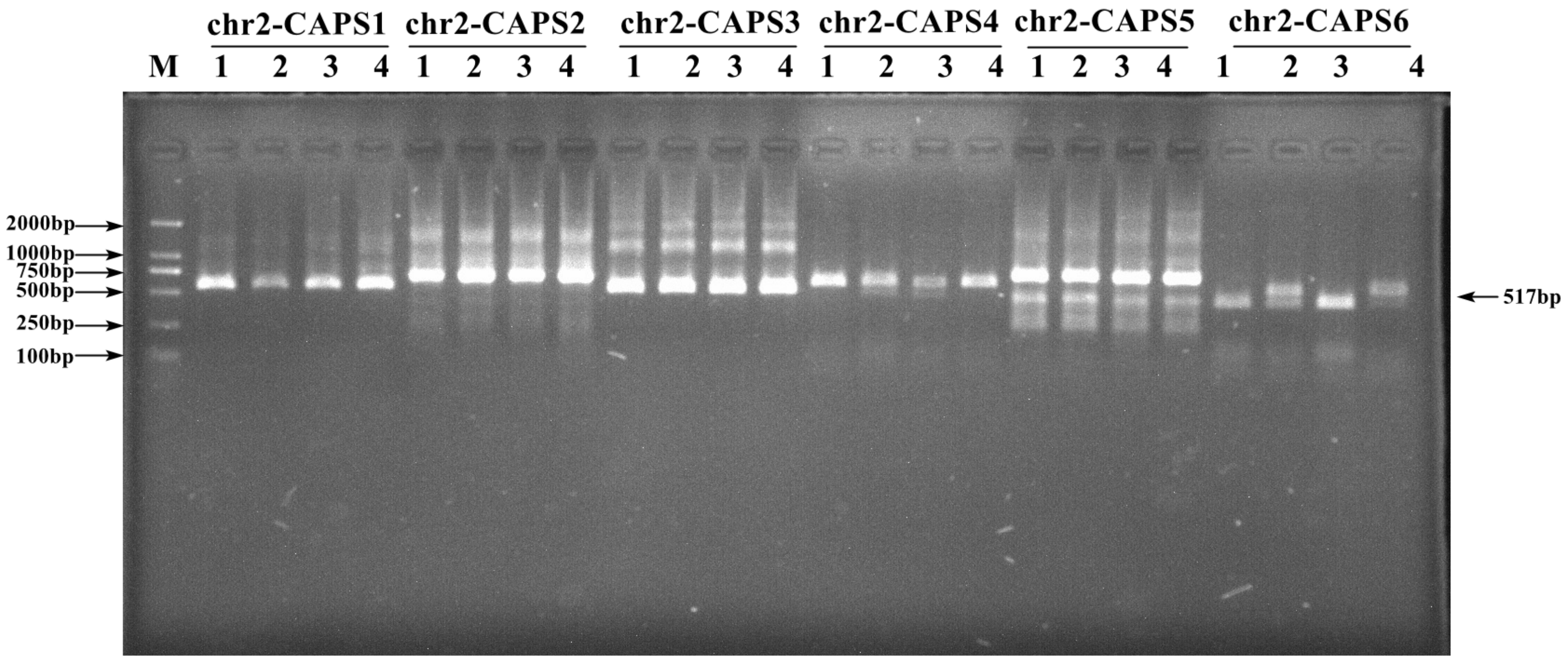

Fig 4A. The screening of partial CAPS primers for potato. The first row in the picture represents different CAPS primers, and the numbers in the second row represent the identities of experimental samples respectively. For example, M represents DL2000Marker, 1 represents female YSP-4, 2 represents male MIN-021, 3 represents high starch mixing pool, and 4 represents low starch mixing pool. The label on the right of the picture represents the molecular weight of the enzyme fragment of chr2-CAPS6. This image was taken using Biometra UVIdoc gel imager.

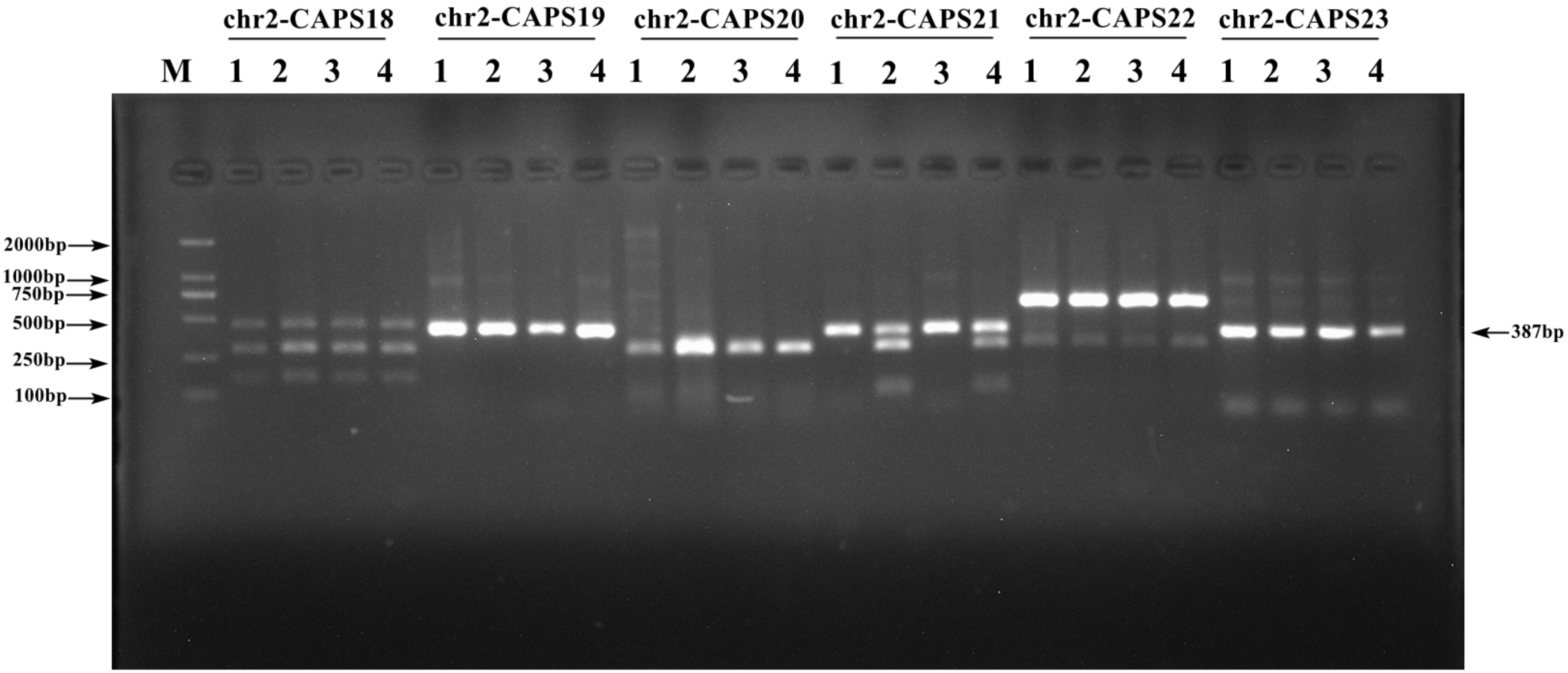

Fig 4B. The screening of partial CAPS primers for potato. The first row in the picture represents different CAPS primers, and the numbers in the second row represent the identities of experimental samples respectively. For example, M represents DL2000Marker, 1 represents female YSP-4, 2 represents male MIN-021, 3 represents high starch mixing pool, and 4 represents low starch mixing pool. The label on the right of the picture represents the molecular weight of the enzyme fragment of chr2-CAPS21. This image was taken using Biometra UVIdoc gel imager.

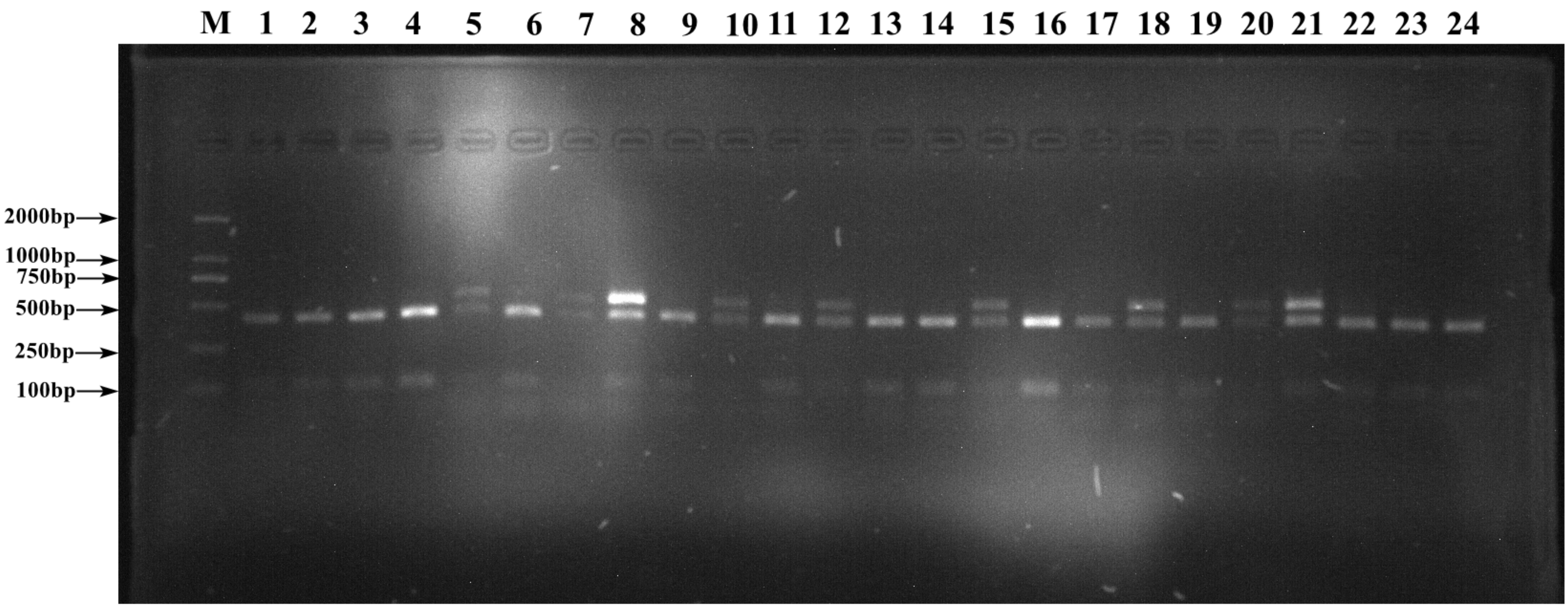

Fig 5A. Partial results of enzyme digestion chr2-CAPS6 marker for F2 population.

M. DL2000 Marker; 1~24. F2 individuals. This image was taken using Biometra UVIdoc gel imager.

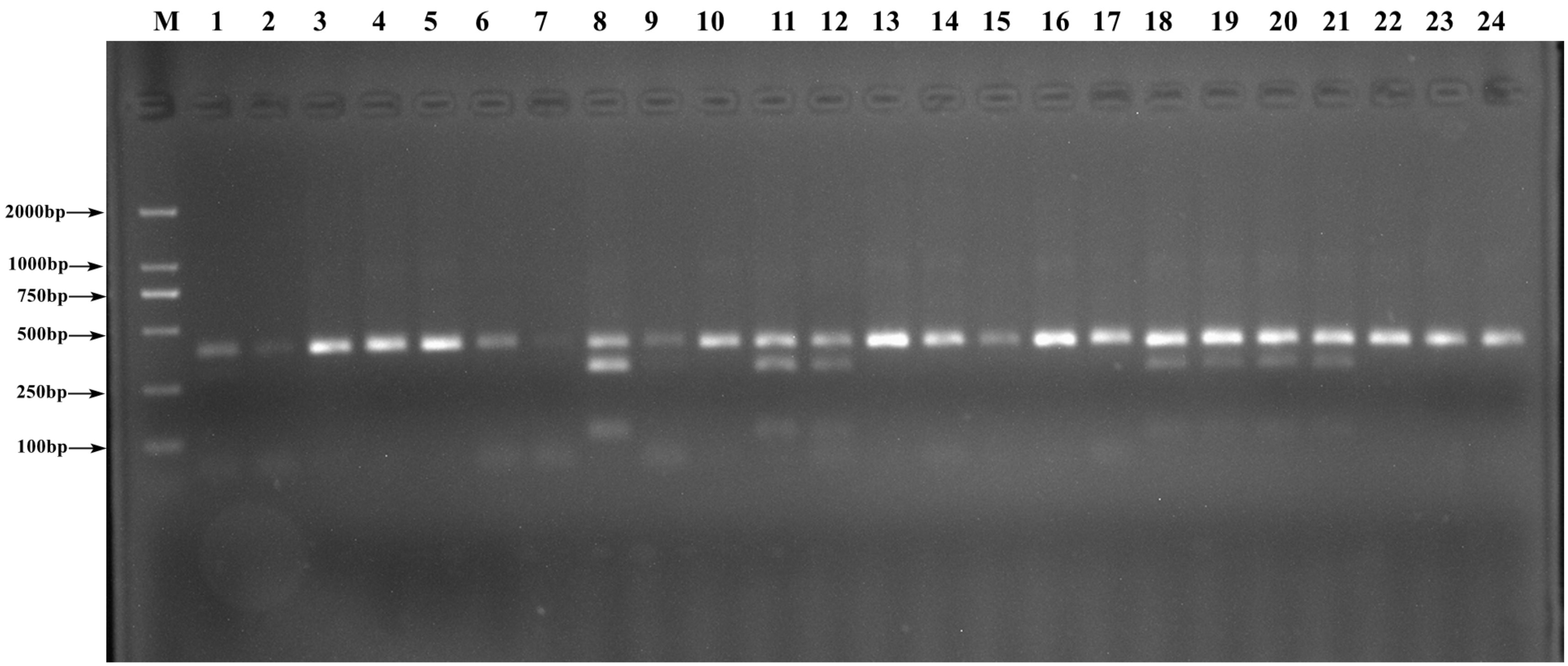

Fig 5B. Partial results of enzyme digestion chr2-CAPS21 marker for F2 population.

M. DL2000 Marker; 1~24. F2 individuals. This image was taken using Biometra UVIdoc gel imager.

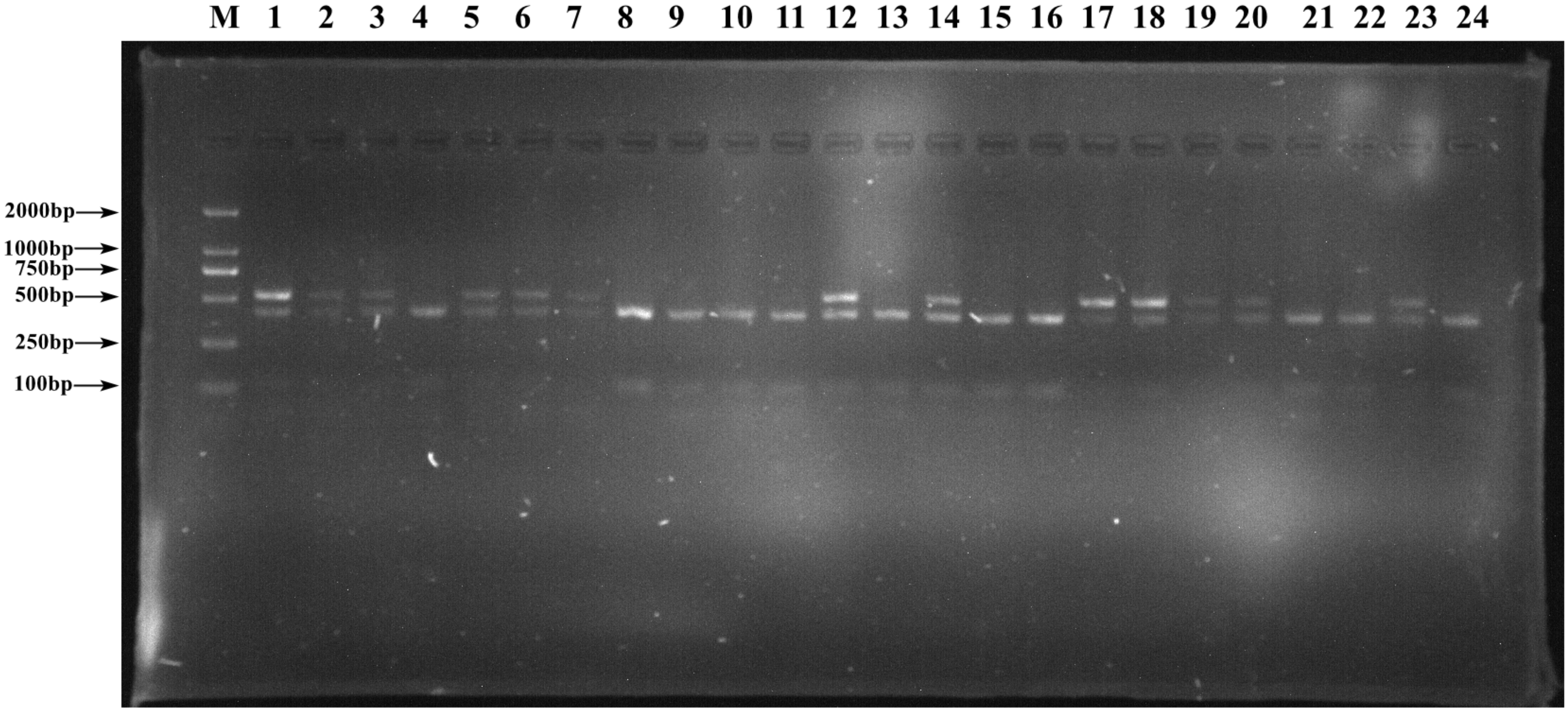

Fig 6A. Enzymatic results chr2-CAPS6 marker in partial tetraploid and strains.

M. DL2000 Marker; 1-24. Tetraploid varieties and strains. This image was taken using Biometra UVIdoc gel imager.

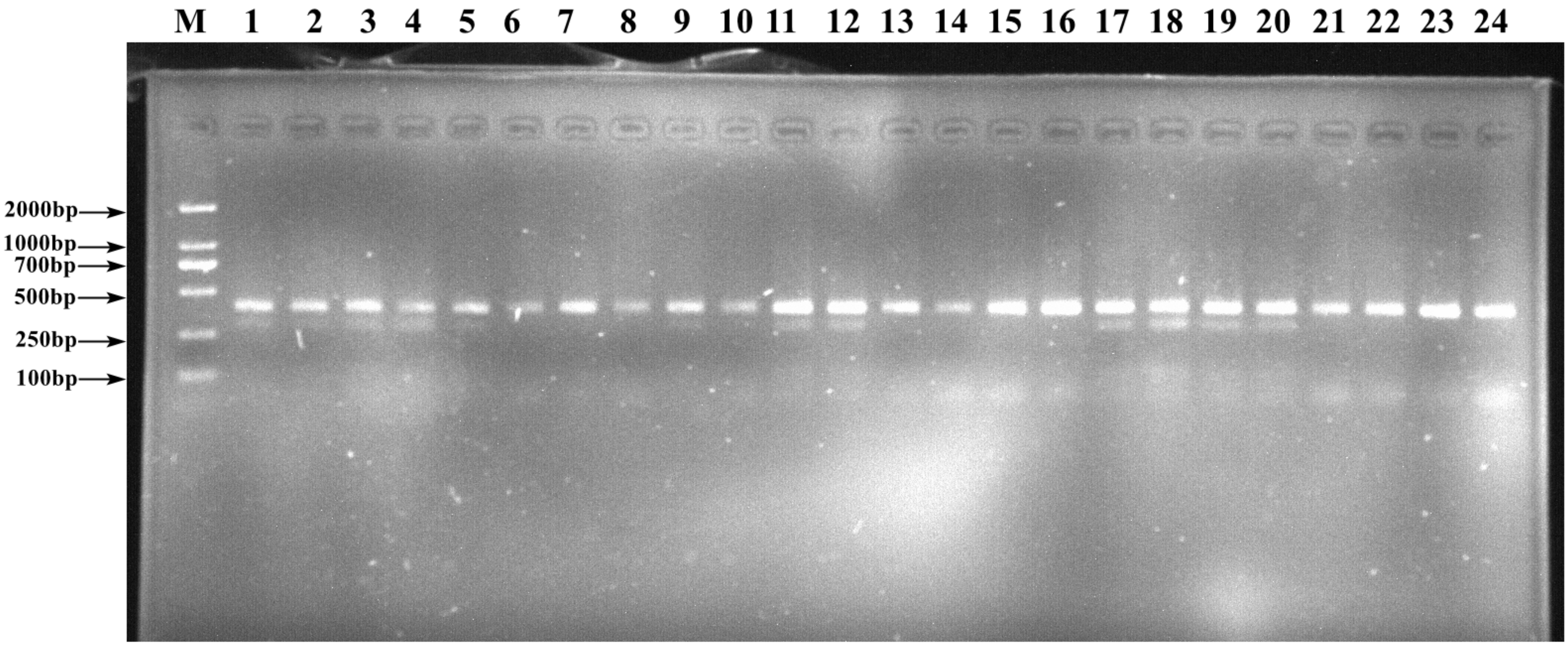

Fig 6B. Enzymatic results chr2-CAPS21 marker in partial tetraploid and strains.

M. DL2000 Marker; 1-24. Tetraploid varieties and strains. This image was taken using Biometra UVIdoc gel imager.
