# Supplementary material for: Identification of starch candidate genes using SLAF-seq and BSA strategies and development of related SNP-CAPS markers in tetraploid potato
Source: PLoS One. 2021 Dec 21;16(12):e0261403. doi: 10.1371/journal.pone.0261403 (PMC8691606; doi:10.1371/journal.pone.0261403)
Supplement: S2 File — (ZIP) [file pone.0261403.s012.zip › merge_region/Anno/Cog_Anno/Solanum_tuberosum_v4.03.Cog.classfy.png.pdf]

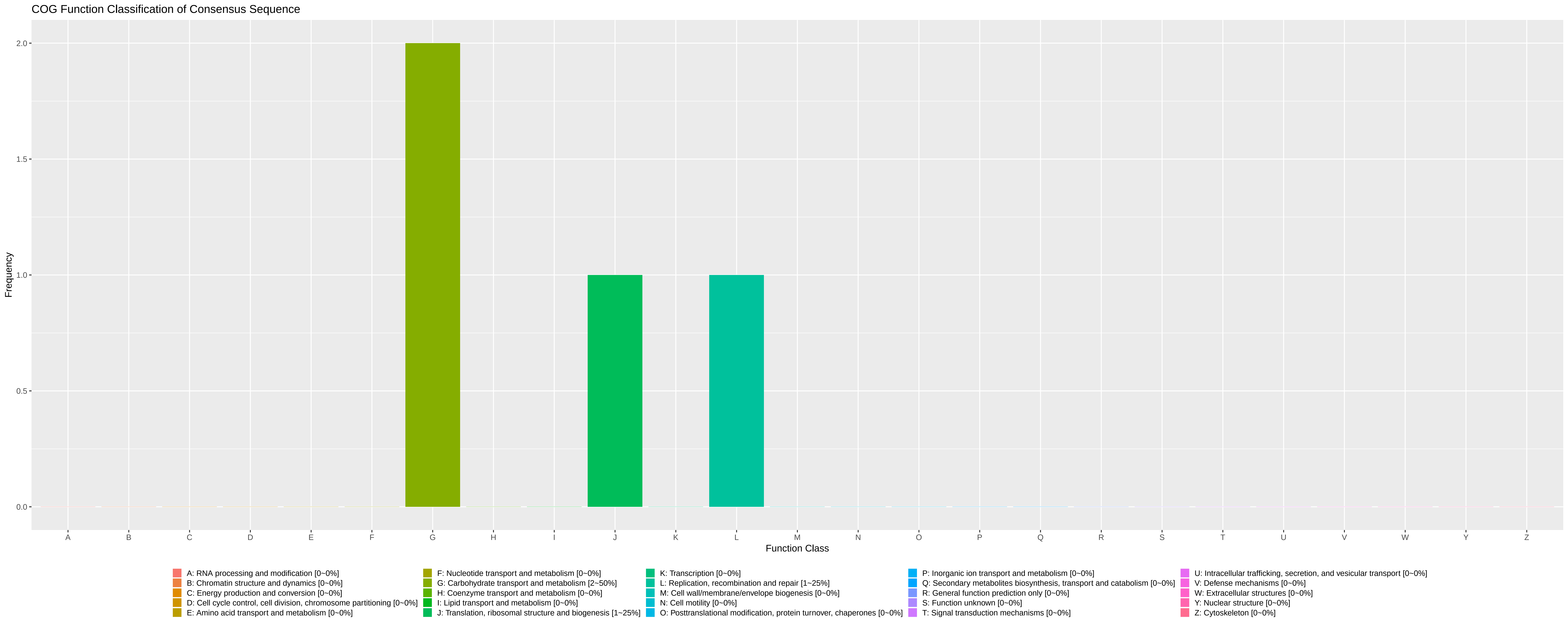
COG Function Classification of Consensus Sequence
Frequency
2.0
1.5
1.0
0.5
0.0
A
B
C
D
E
F
G
H
I
J
K
L
M
N
O
P
Q
R
S
T
U
V
W
Y
Z
Function Class
A: RNA processing and modification [0~0%]
B: Chromatin structure and dynamics [0~0%]
C: Energy production and conversion [0~0%]
D: Cell cycle control, cell division, chromosome partitioning [0~0%]
E: Amino acid transport and metabolism [0~0%]
F: Nucleotide transport and metabolism [0~0%]
G: Carbohydrate transport and metabolism [2~50%]
H: Coenzyme transport and metabolism [0~0%]
I: Lipid transport and metabolism [0~0%]
J: Translation, ribosomal structure and biogenesis [1~25%]
K: Transcription [0~0%]
L: Replication, recombination and repair [1~25%]
M: Cell wall/membrane/envelope biogenesis [0~0%]
N: Cell motility [0~0%]
O: Posttranslational modification, protein turnover, chaperones [0~0%]
P: Inorganic ion transport and metabolism [0~0%]
Q: Secondary metabolites biosynthesis, transport and catabolism [0~0%]
R: General function prediction only [0~0%]
S: Function unknown [0~0%]
T: Signal transduction mechanisms [0~0%]
U: Intracellular trafficking, secretion, and vesicular transport [0~0%]
V: Defense mechanisms [0~0%]
W: Extracellular structures [0~0%]
Y: Nuclear structure [0~0%]
Z: Cytoskeleton [0~0%]
