# Supplementary figures and images for: Identification of starch candidate genes using SLAF-seq and BSA strategies and development of related SNP-CAPS markers in tetraploid potato (part 2 of 2)
Source: PLoS One. 2021 Dec 21;16(12):e0261403. doi: 10.1371/journal.pone.0261403 (PMC8691606; doi:10.1371/journal.pone.0261403)

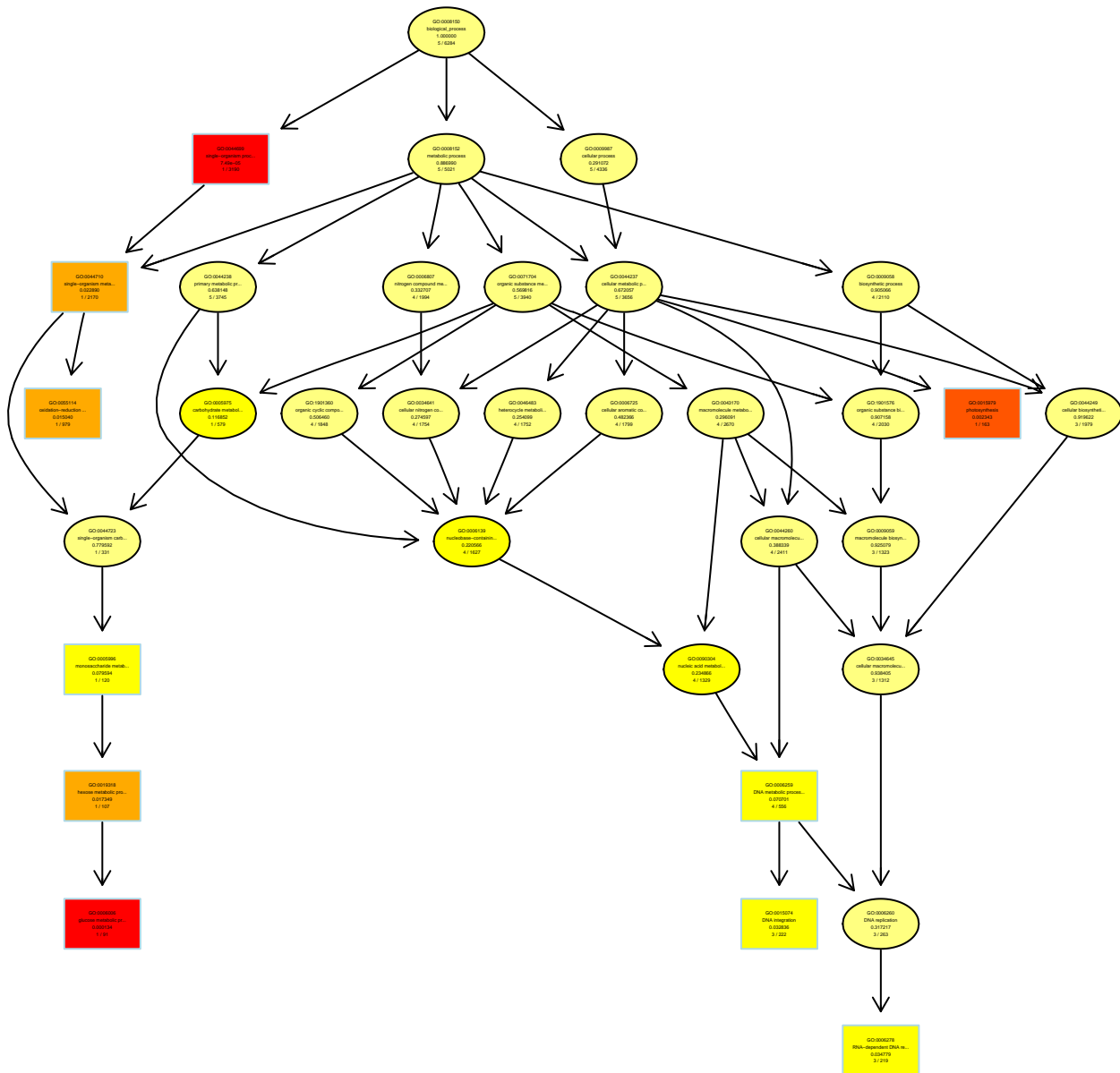

Supplement: S2 File — (ZIP) [file pone.0261403.s012.zip › merge_region/SNPAnno/GO_Anno/topGO/Solanum_tuberosum_v4.03.topGO_BP.pdf]

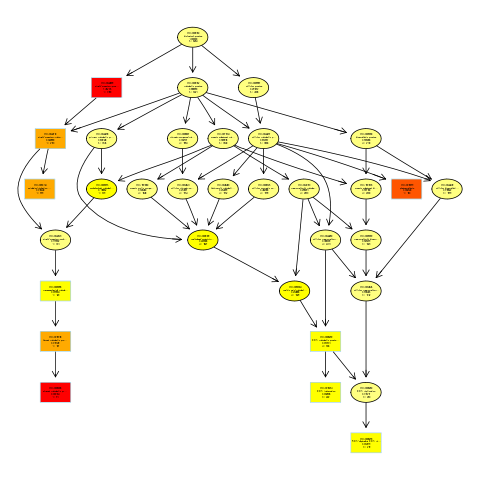

Supplement: S2 File — (ZIP) [file pone.0261403.s012.zip › merge_region/SNPAnno/GO_Anno/topGO/Solanum_tuberosum_v4.03.topGO_BP.png]

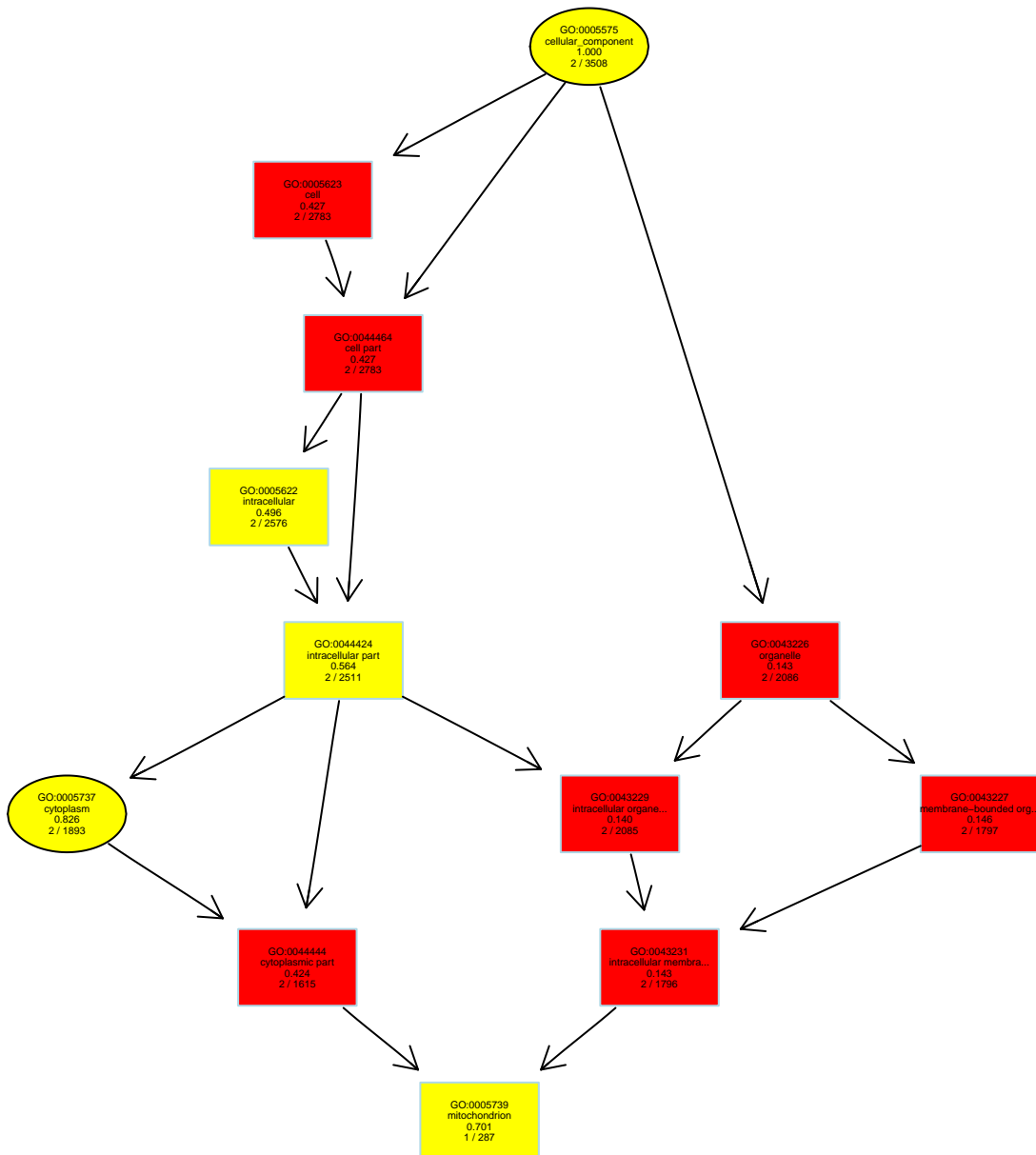

Supplement: S2 File — (ZIP) [file pone.0261403.s012.zip › merge_region/SNPAnno/GO_Anno/topGO/Solanum_tuberosum_v4.03.topGO_CC.pdf]

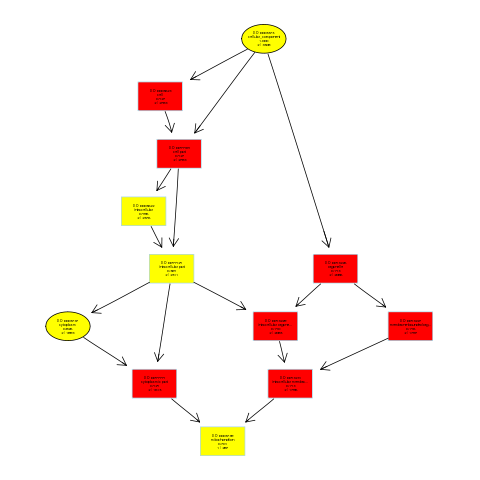

Supplement: S2 File — (ZIP) [file pone.0261403.s012.zip › merge_region/SNPAnno/GO_Anno/topGO/Solanum_tuberosum_v4.03.topGO_CC.png]

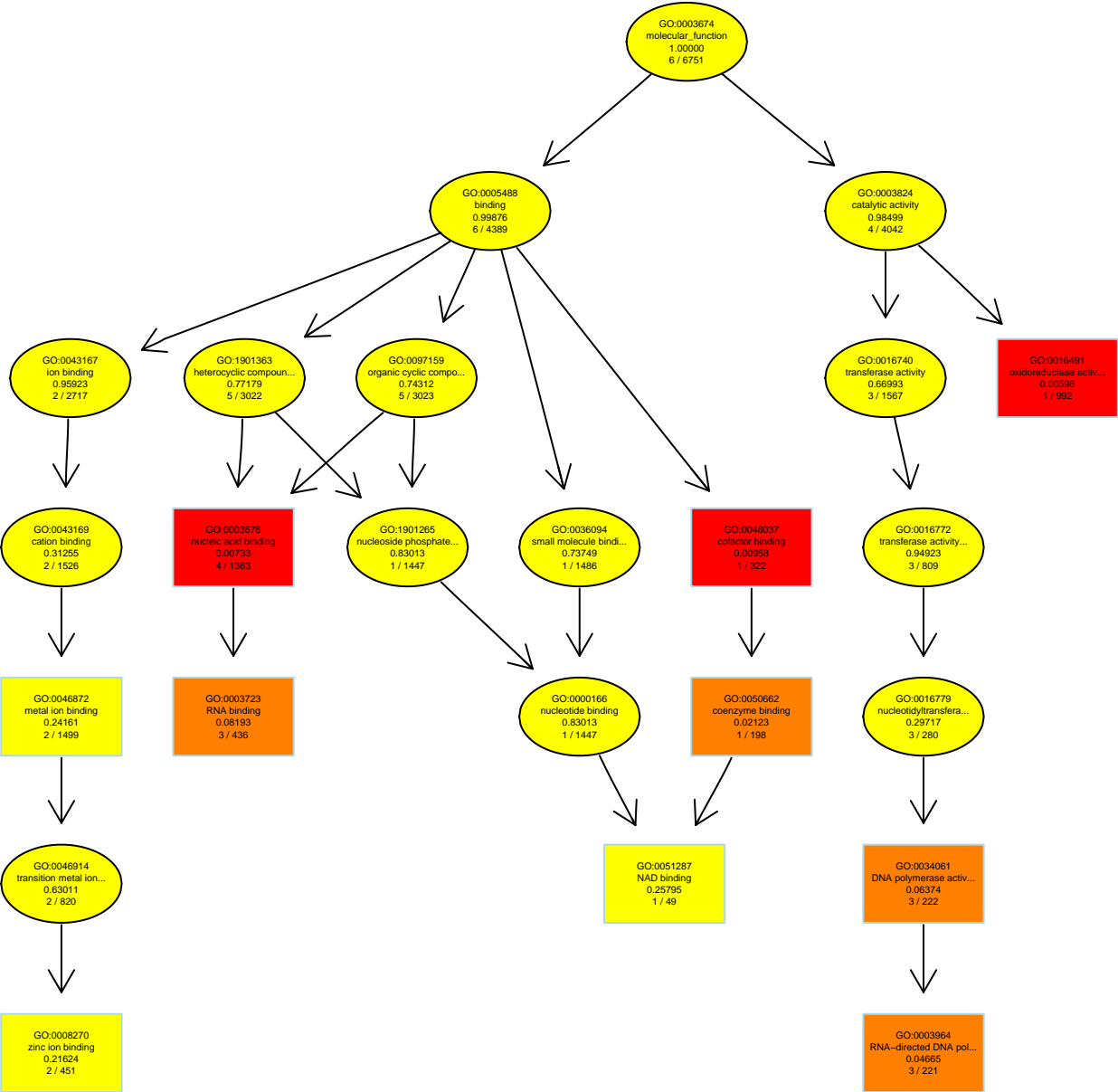

Supplement: S2 File — (ZIP) [file pone.0261403.s012.zip › merge_region/SNPAnno/GO_Anno/topGO/Solanum_tuberosum_v4.03.topGO_MF.pdf]

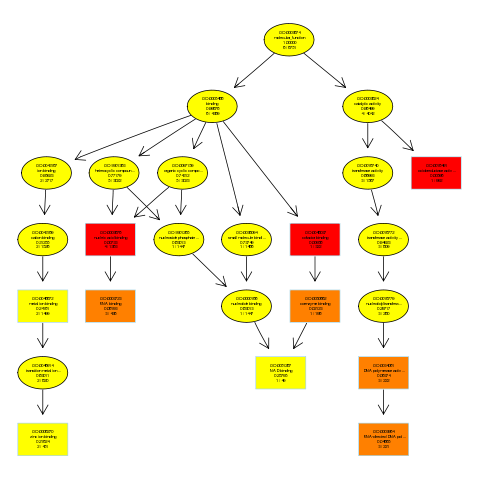

Supplement: S2 File — (ZIP) [file pone.0261403.s012.zip › merge_region/SNPAnno/GO_Anno/topGO/Solanum_tuberosum_v4.03.topGO_MF.png]

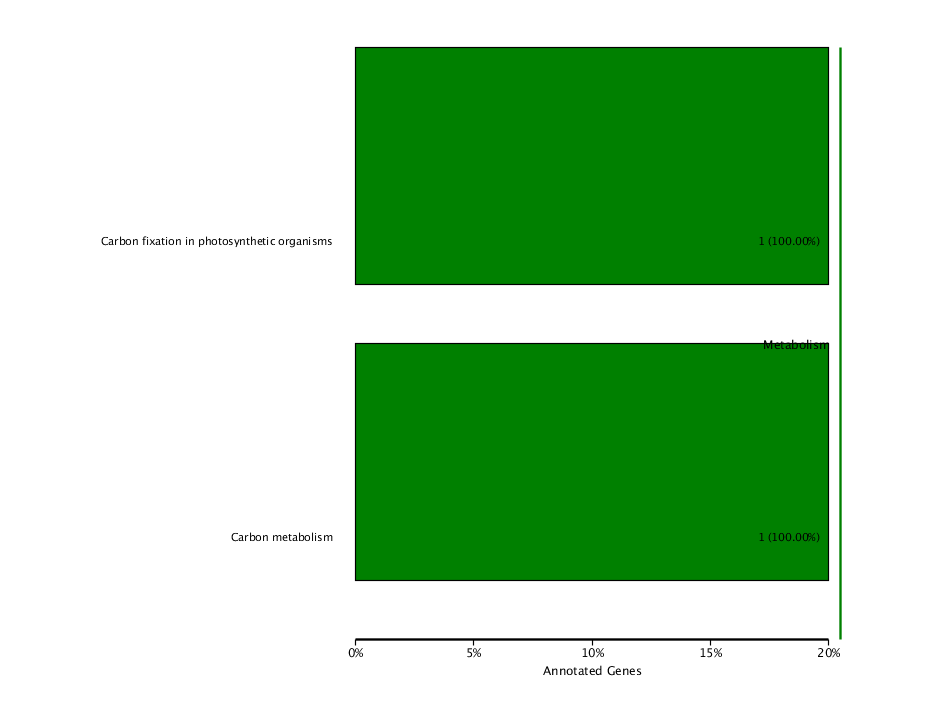

Supplement: S2 File — (ZIP) [file pone.0261403.s012.zip › merge_region/SNPAnno/pathway/kegg_enrichment/Solanum_tuberosum_v4.03.KEGG.png]

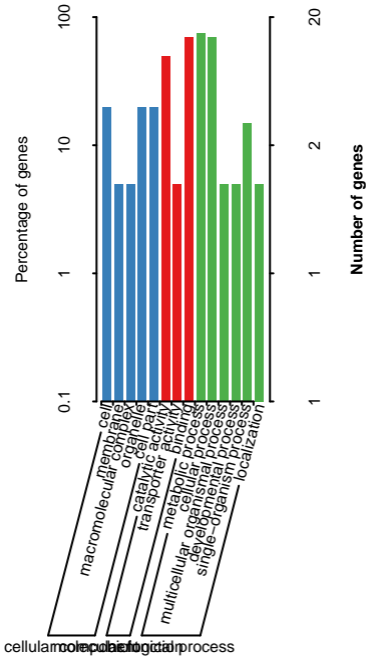

Supplement: S3 File — (ZIP) [file pone.0261403.s013.zip › SNP-index/Anno/GeneAnno/GO_Anno/go_enrichment/Solanum_tuberosum_v4.03.GO.pdf]

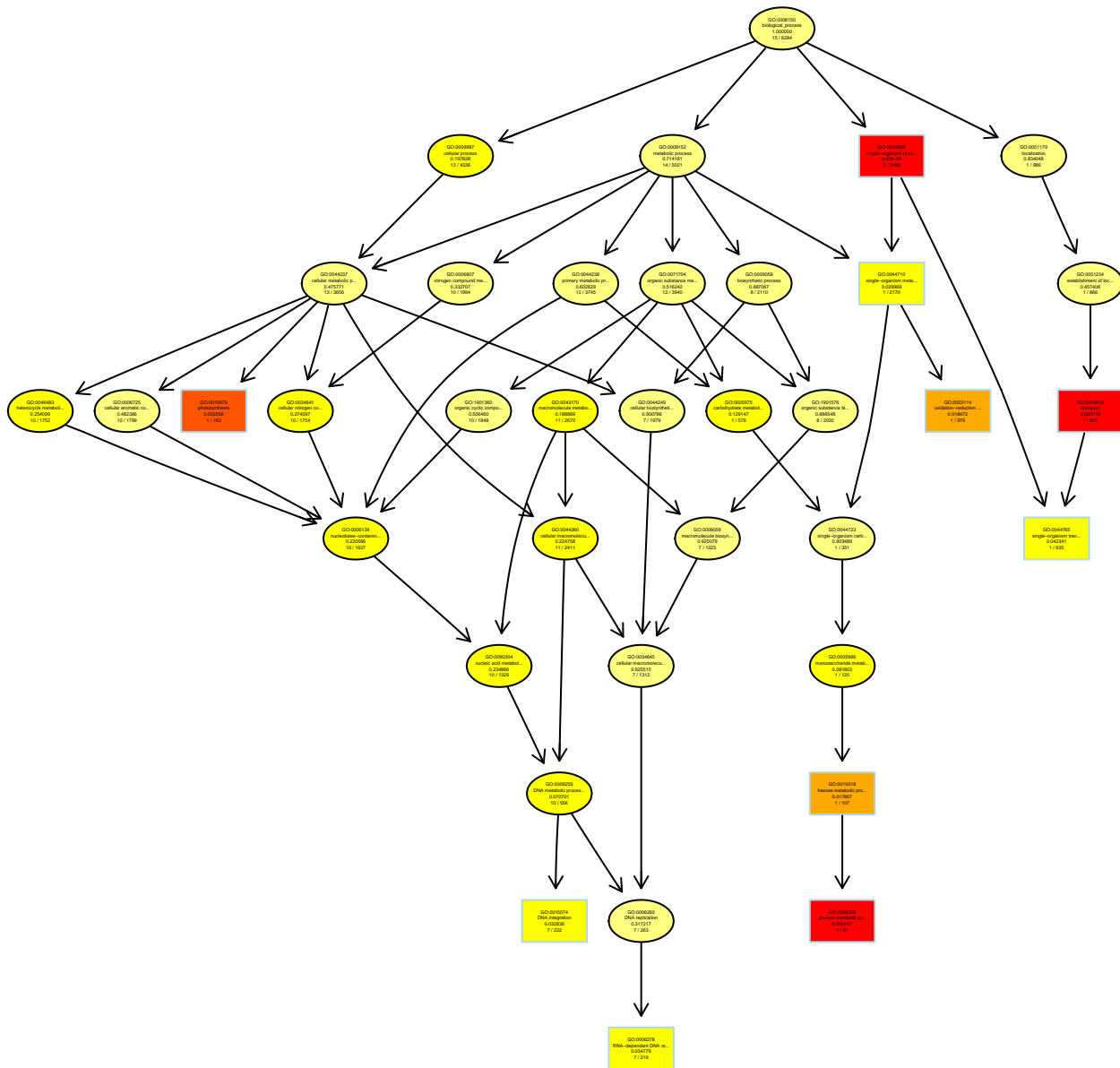

Supplement: S3 File — (ZIP) [file pone.0261403.s013.zip › SNP-index/Anno/GeneAnno/GO_Anno/topGO/Solanum_tuberosum_v4.03.topGO_BP.pdf]

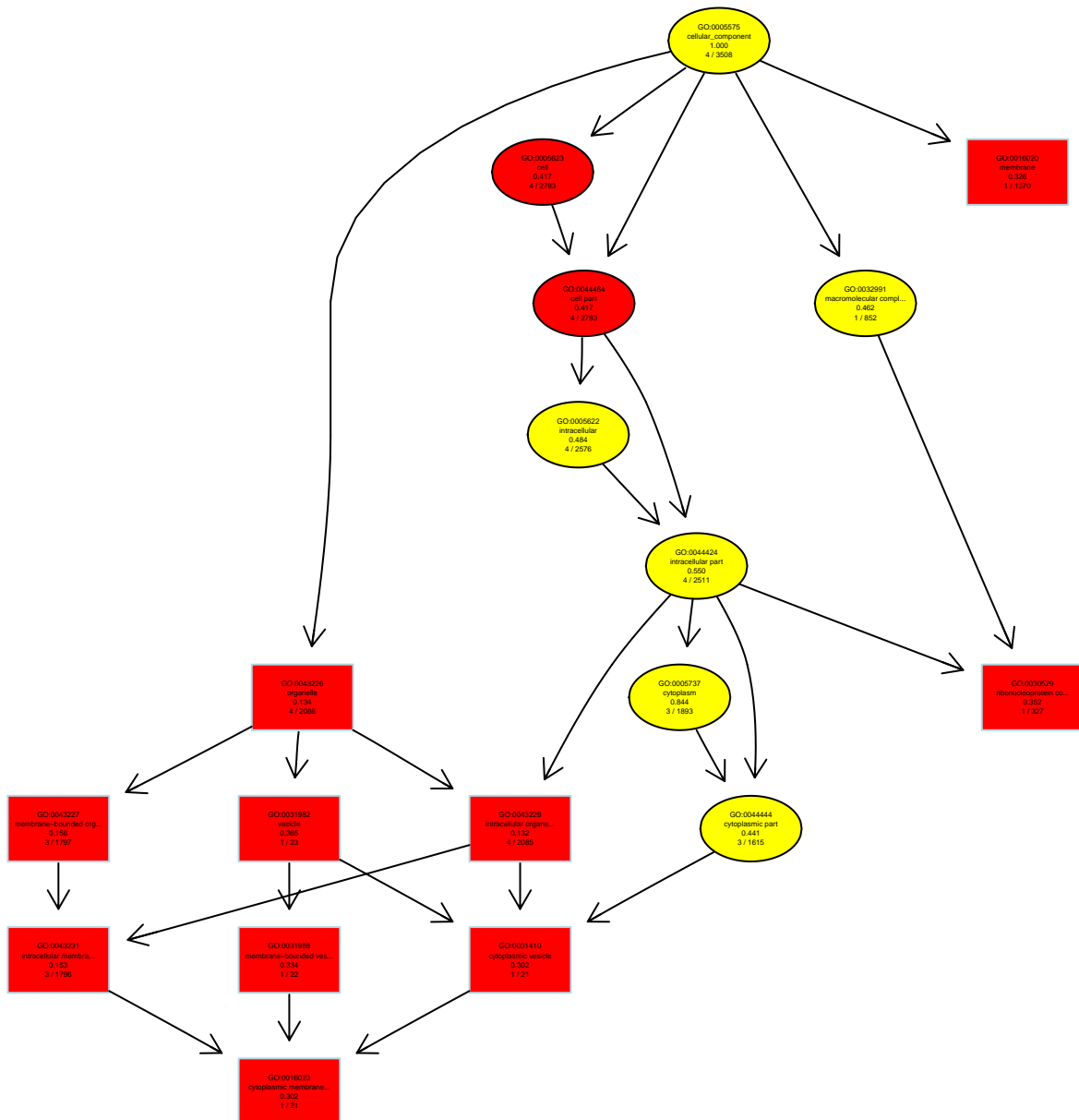

Supplement: S3 File — (ZIP) [file pone.0261403.s013.zip › SNP-index/Anno/GeneAnno/GO_Anno/topGO/Solanum_tuberosum_v4.03.topGO_CC.pdf]

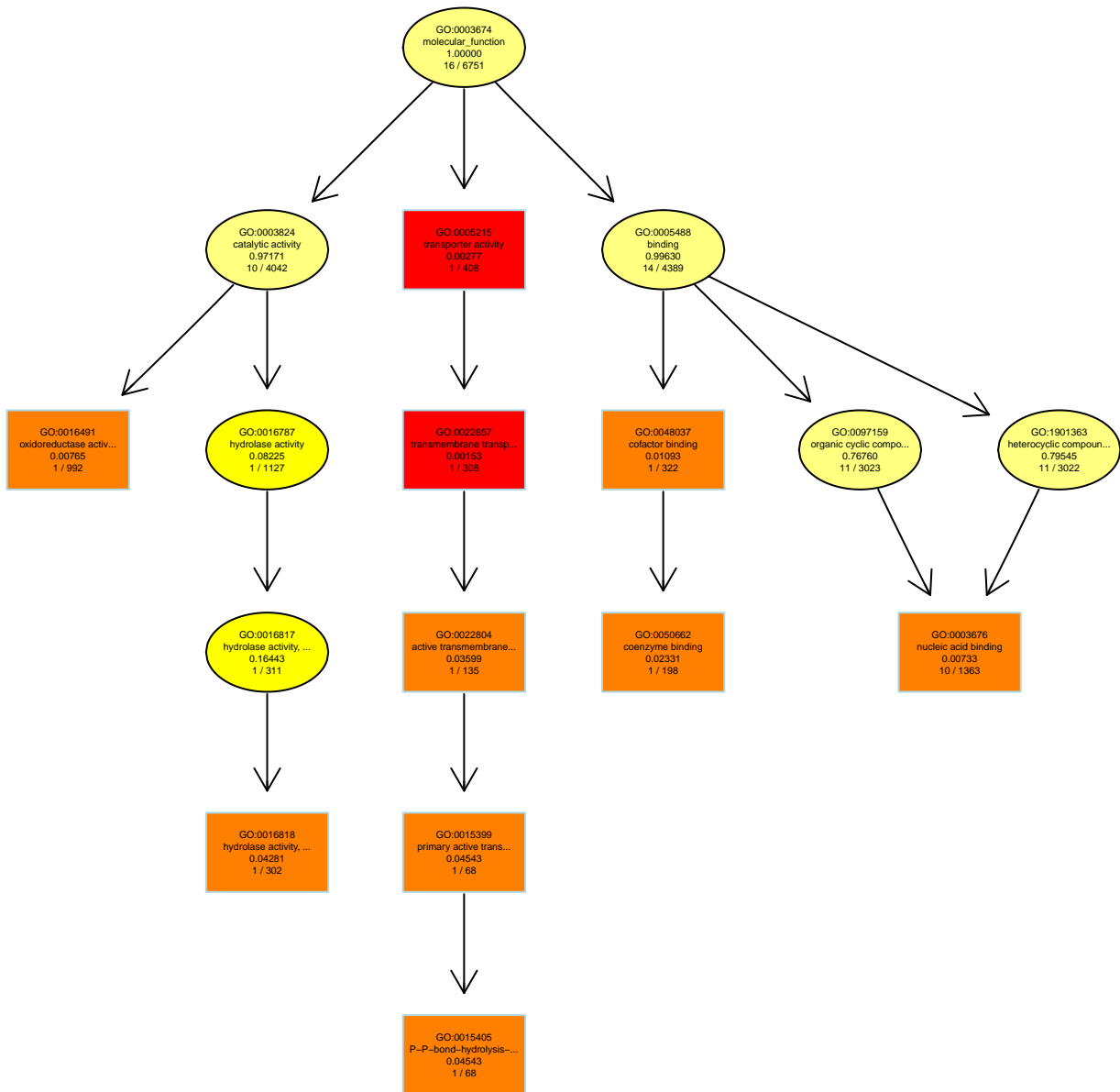

Supplement: S3 File — (ZIP) [file pone.0261403.s013.zip › SNP-index/Anno/GeneAnno/GO_Anno/topGO/Solanum_tuberosum_v4.03.topGO_MF.pdf]

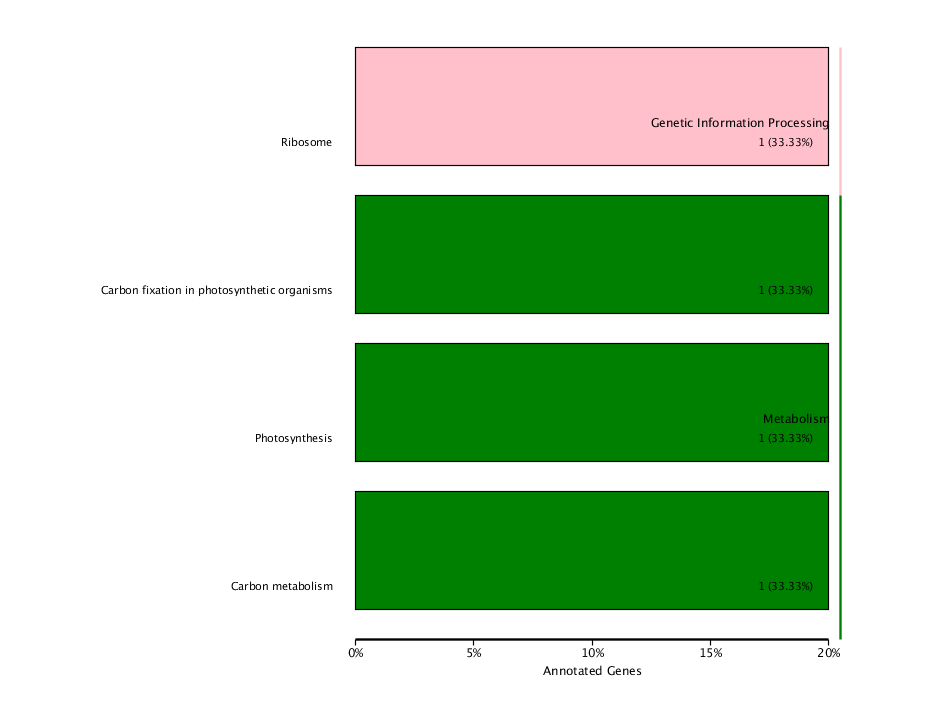

Supplement: S3 File — (ZIP) [file pone.0261403.s013.zip › SNP-index/Anno/GeneAnno/pathway/kegg_enrichment/Solanum_tuberosum_v4.03.png]

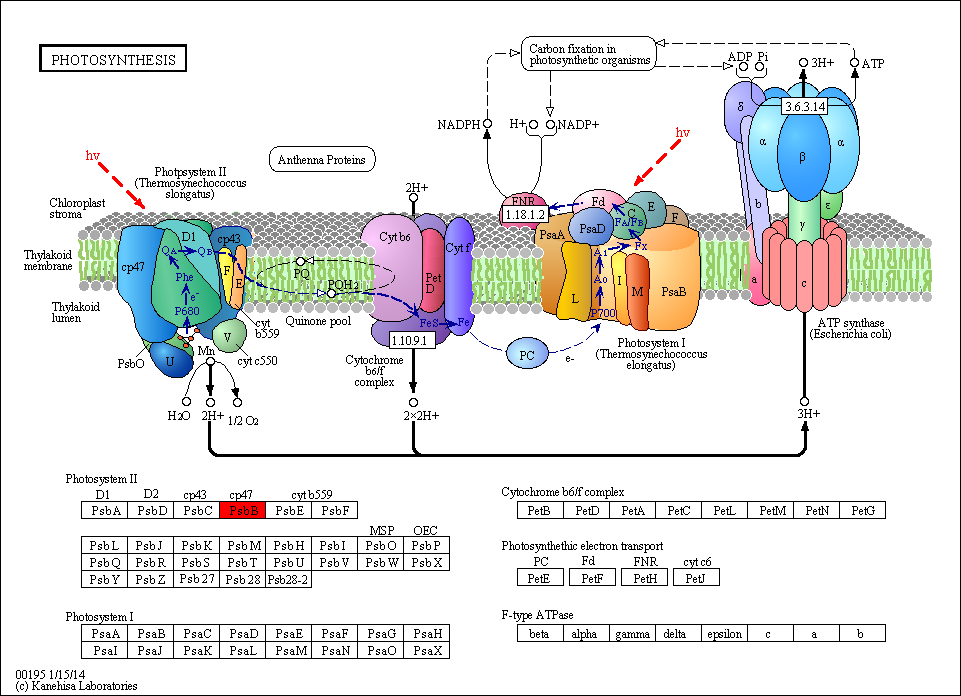

Supplement: S3 File — (ZIP) [file pone.0261403.s013.zip › SNP-index/Anno/GeneAnno/pathway/kegg_map/ko00195.png]

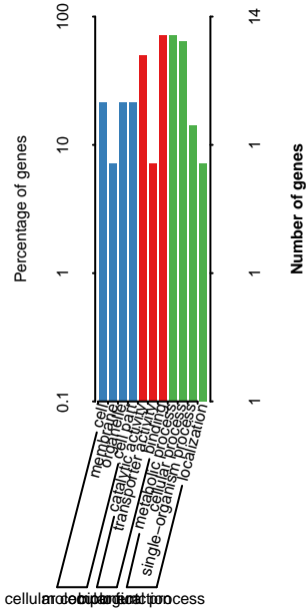

Supplement: S3 File — (ZIP) [file pone.0261403.s013.zip › SNP-index/Anno/SNPAnno/GO_Anno/go_enrichment/Solanum_tuberosum_v4.03.GO.pdf]

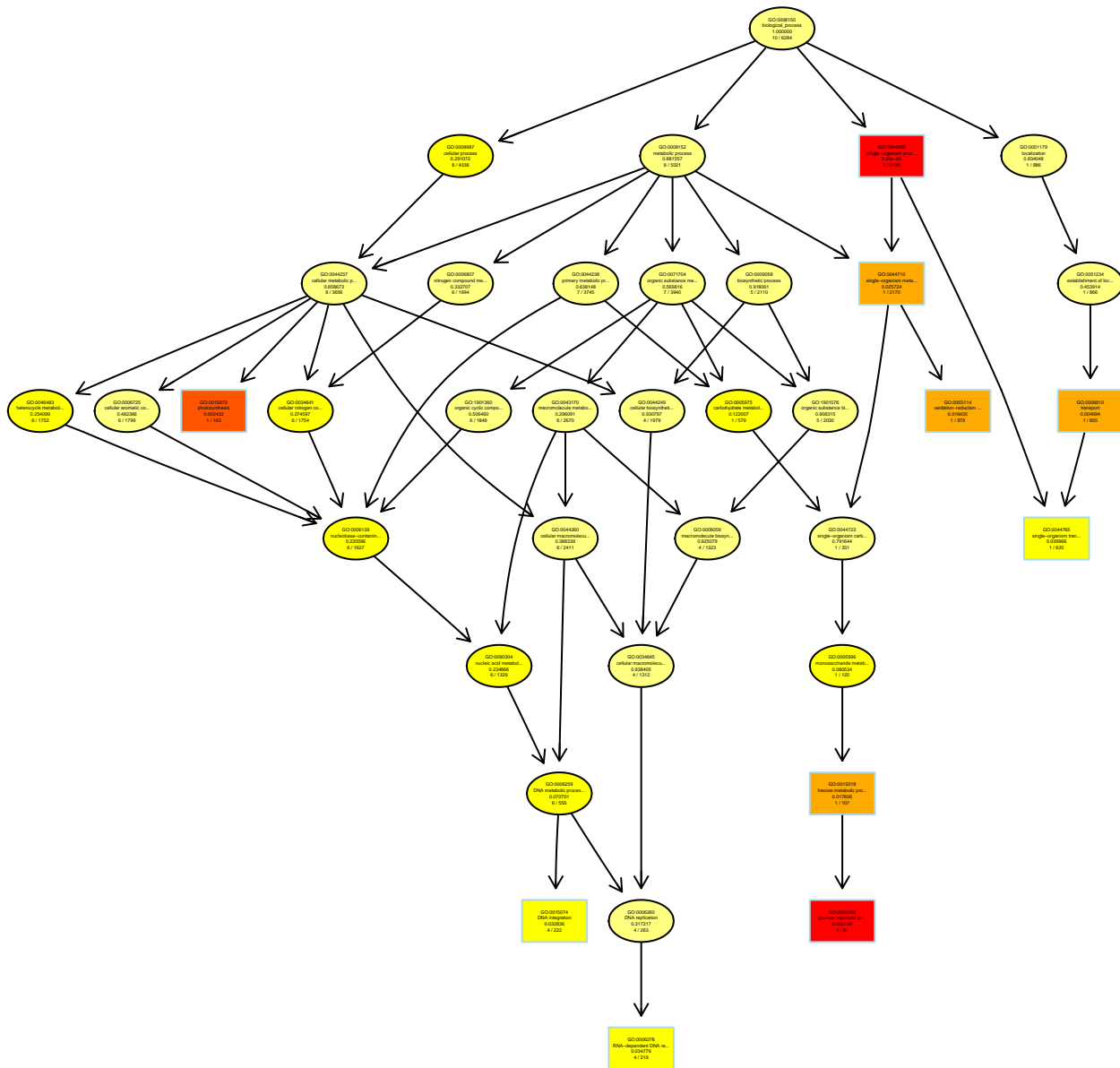

Supplement: S3 File — (ZIP) [file pone.0261403.s013.zip › SNP-index/Anno/SNPAnno/GO_Anno/topGO/Solanum_tuberosum_v4.03.topGO_BP.pdf]

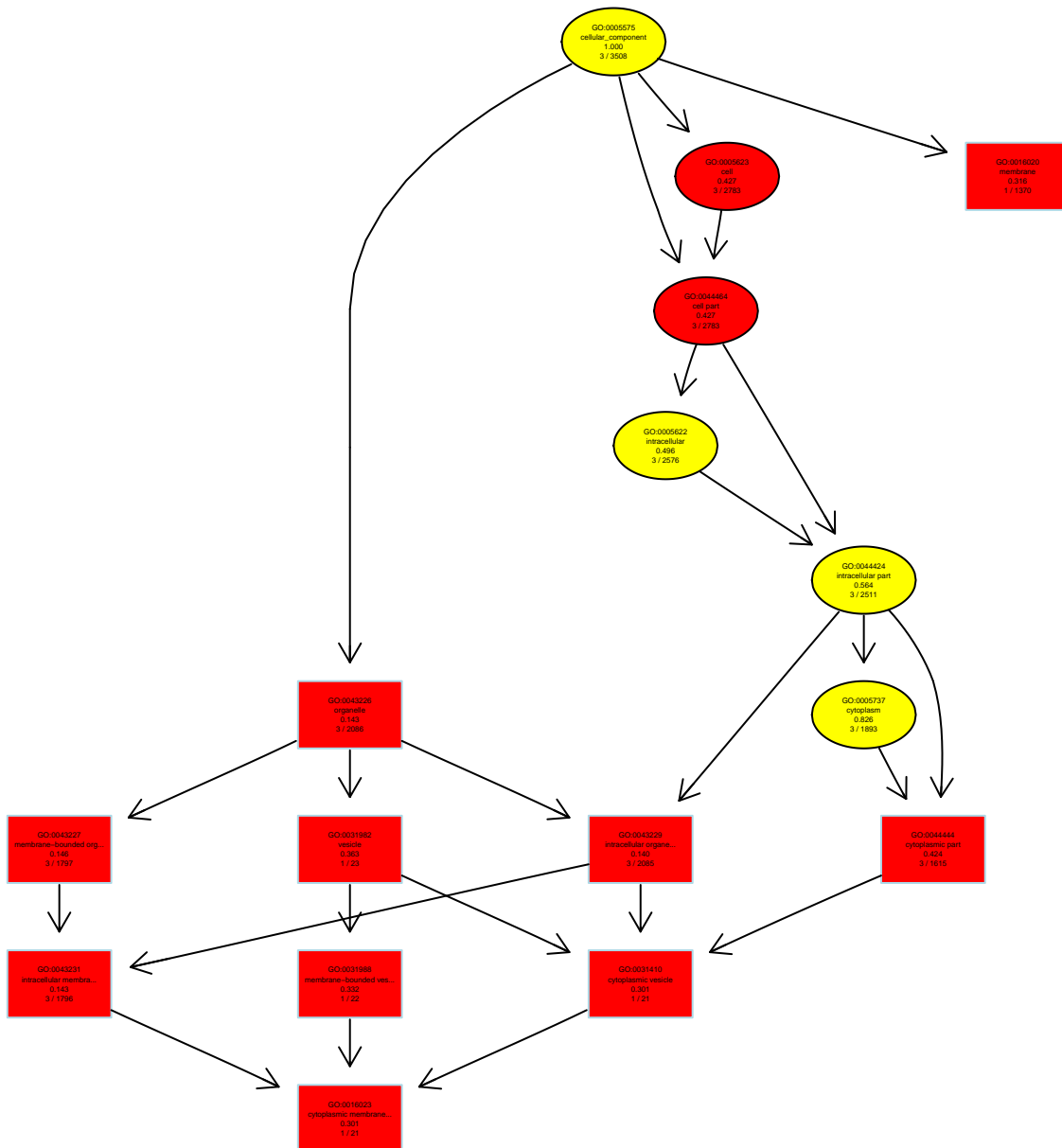

Supplement: S3 File — (ZIP) [file pone.0261403.s013.zip › SNP-index/Anno/SNPAnno/GO_Anno/topGO/Solanum_tuberosum_v4.03.topGO_CC.pdf]

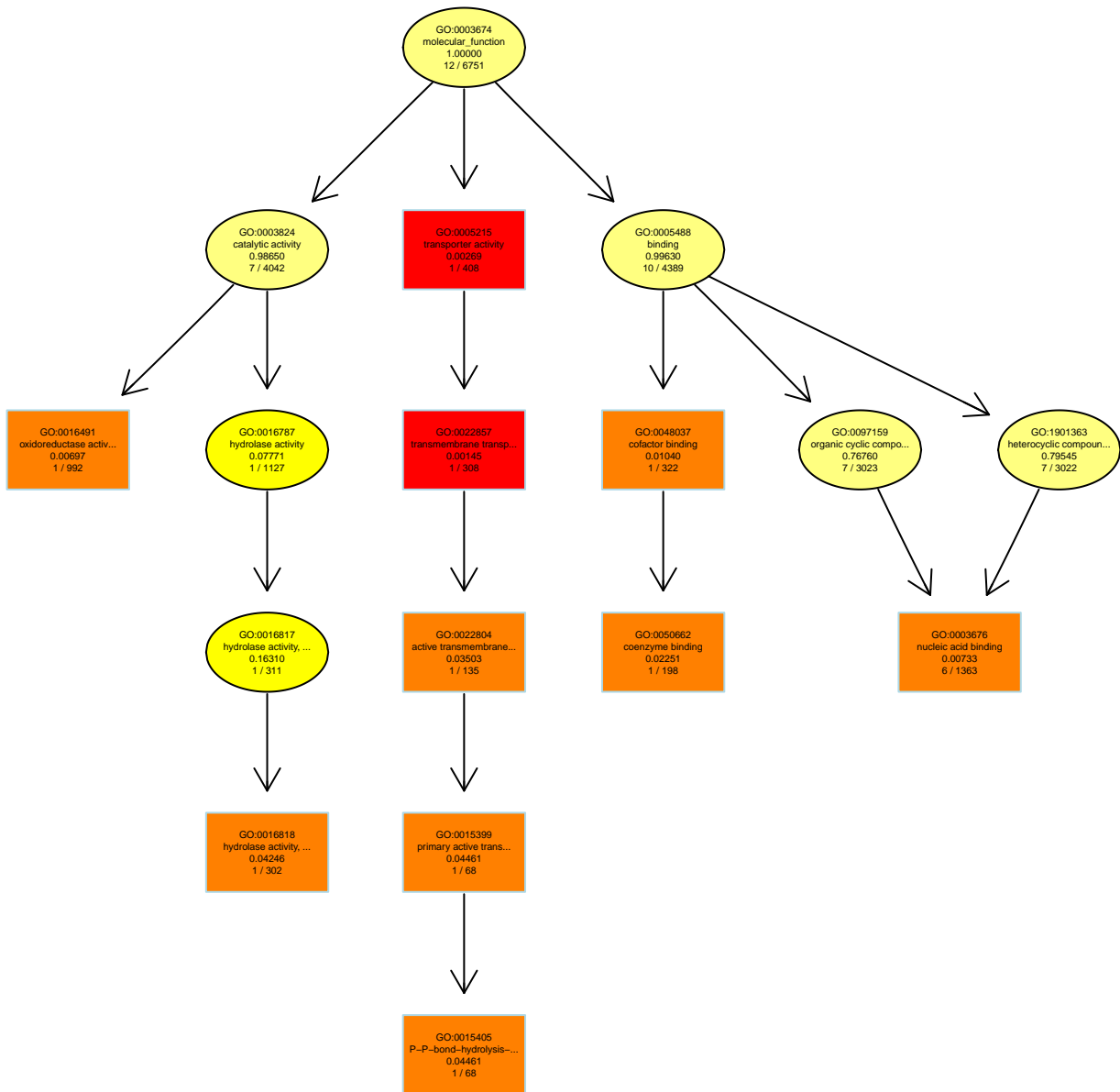

Supplement: S3 File — (ZIP) [file pone.0261403.s013.zip › SNP-index/Anno/SNPAnno/GO_Anno/topGO/Solanum_tuberosum_v4.03.topGO_MF.pdf]

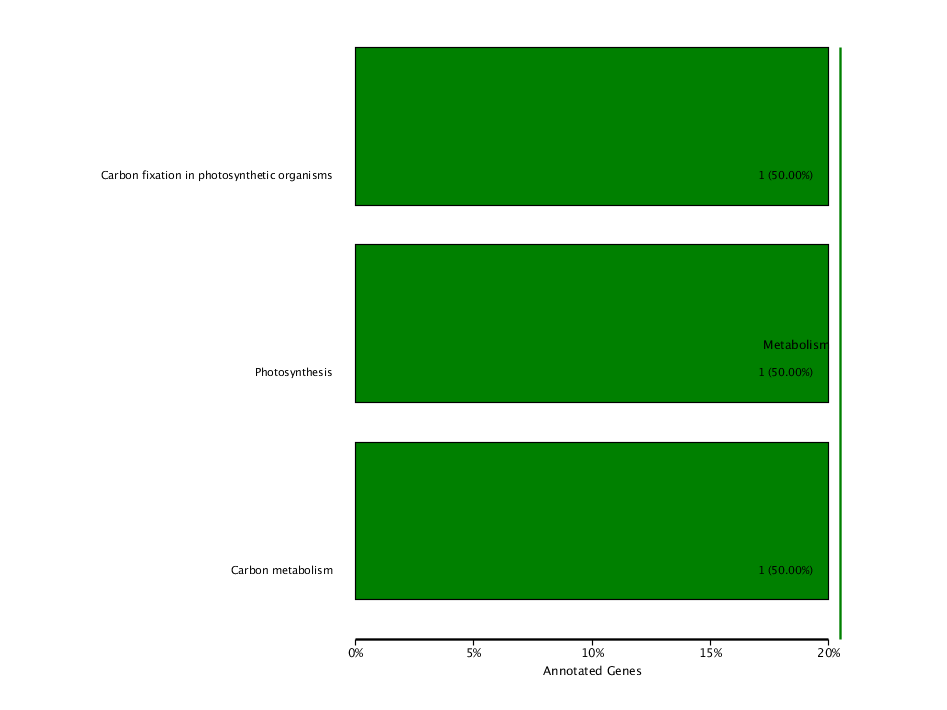

Supplement: S3 File — (ZIP) [file pone.0261403.s013.zip › SNP-index/Anno/SNPAnno/pathway/kegg_enrichment/Solanum_tuberosum_v4.03.KEGG.png]

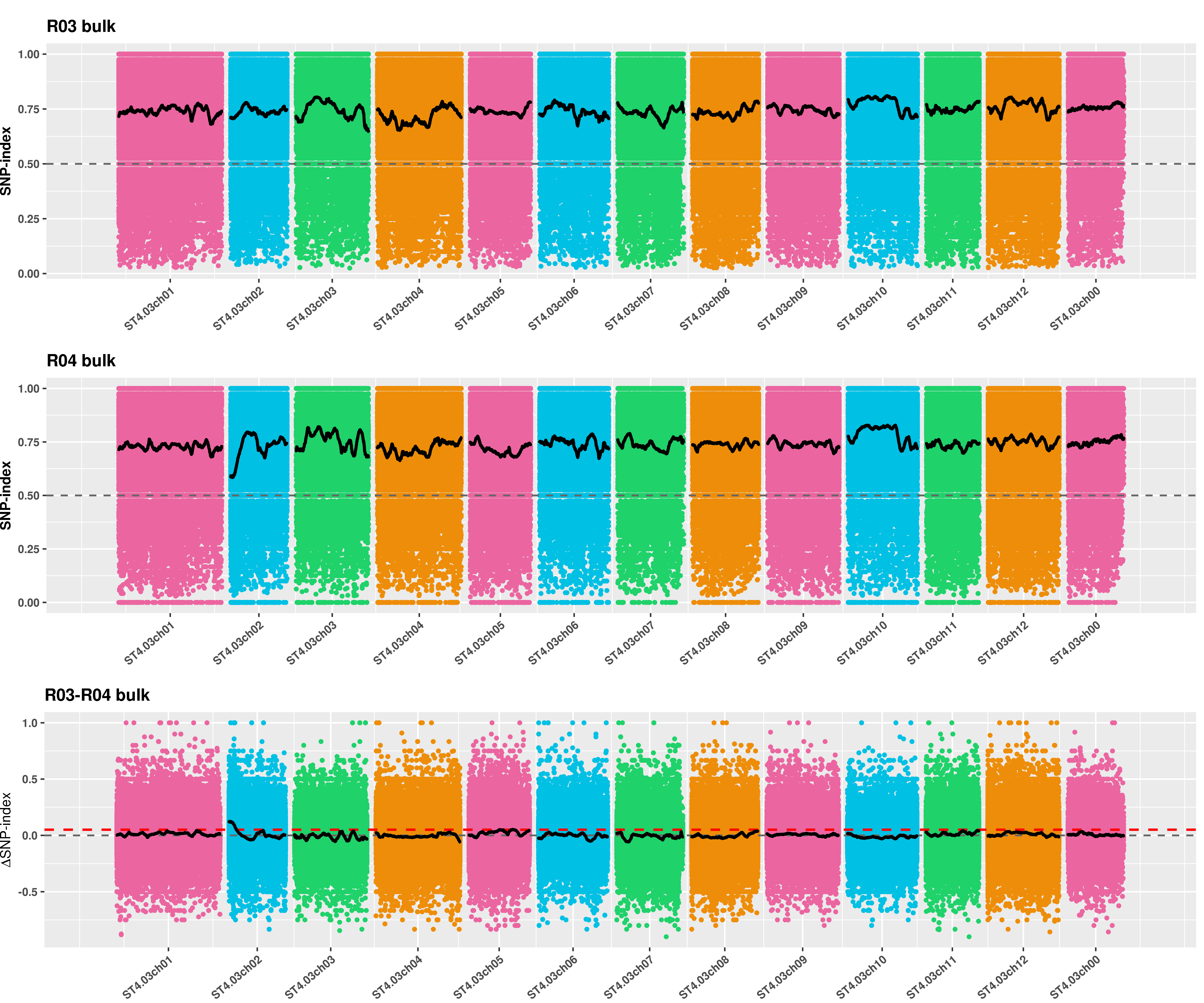

Supplement: S3 File — (ZIP) [file pone.0261403.s013.zip › SNP-index/png/quantile99_3/total.plot.png]
