# Supplementary material for: Identification of starch candidate genes using SLAF-seq and BSA strategies and development of related SNP-CAPS markers in tetraploid potato
Source: PLoS One. 2021 Dec 21;16(12):e0261403. doi: 10.1371/journal.pone.0261403 (PMC8691606; doi:10.1371/journal.pone.0261403)

COG Function Classification of Consensus Sequence

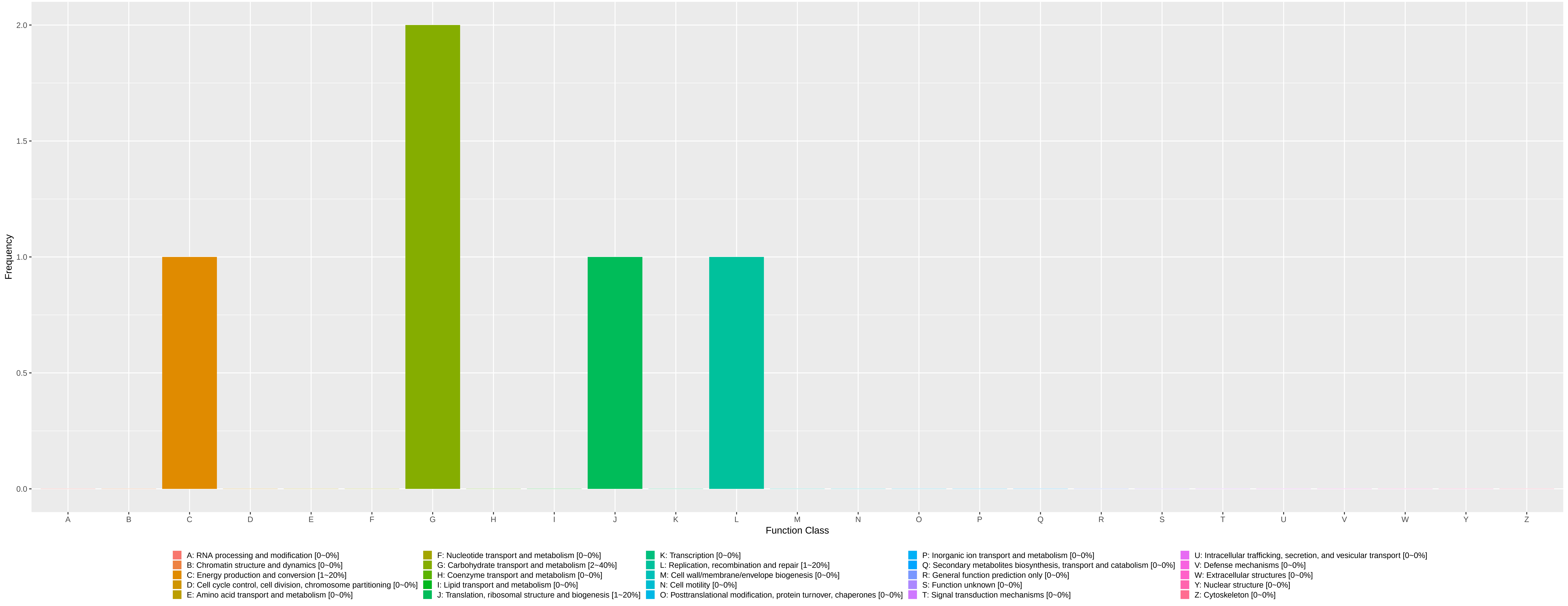

Supplement: S3 File — (ZIP) [file pone.0261403.s013.zip › SNP-index/Anno/GeneAnno/Cog_Anno/Solanum_tuberosum_v4.03.Cog.classfy.png.pdf]
