# Supplementary material for: Identification of starch candidate genes using SLAF-seq and BSA strategies and development of related SNP-CAPS markers in tetraploid potato
Source: PLoS One. 2021 Dec 21;16(12):e0261403. doi: 10.1371/journal.pone.0261403 (PMC8691606; doi:10.1371/journal.pone.0261403)
Supplement: S1 File — (ZIP) [file pone.0261403.s011.zip › ED/png/total.plot.png.pdf]

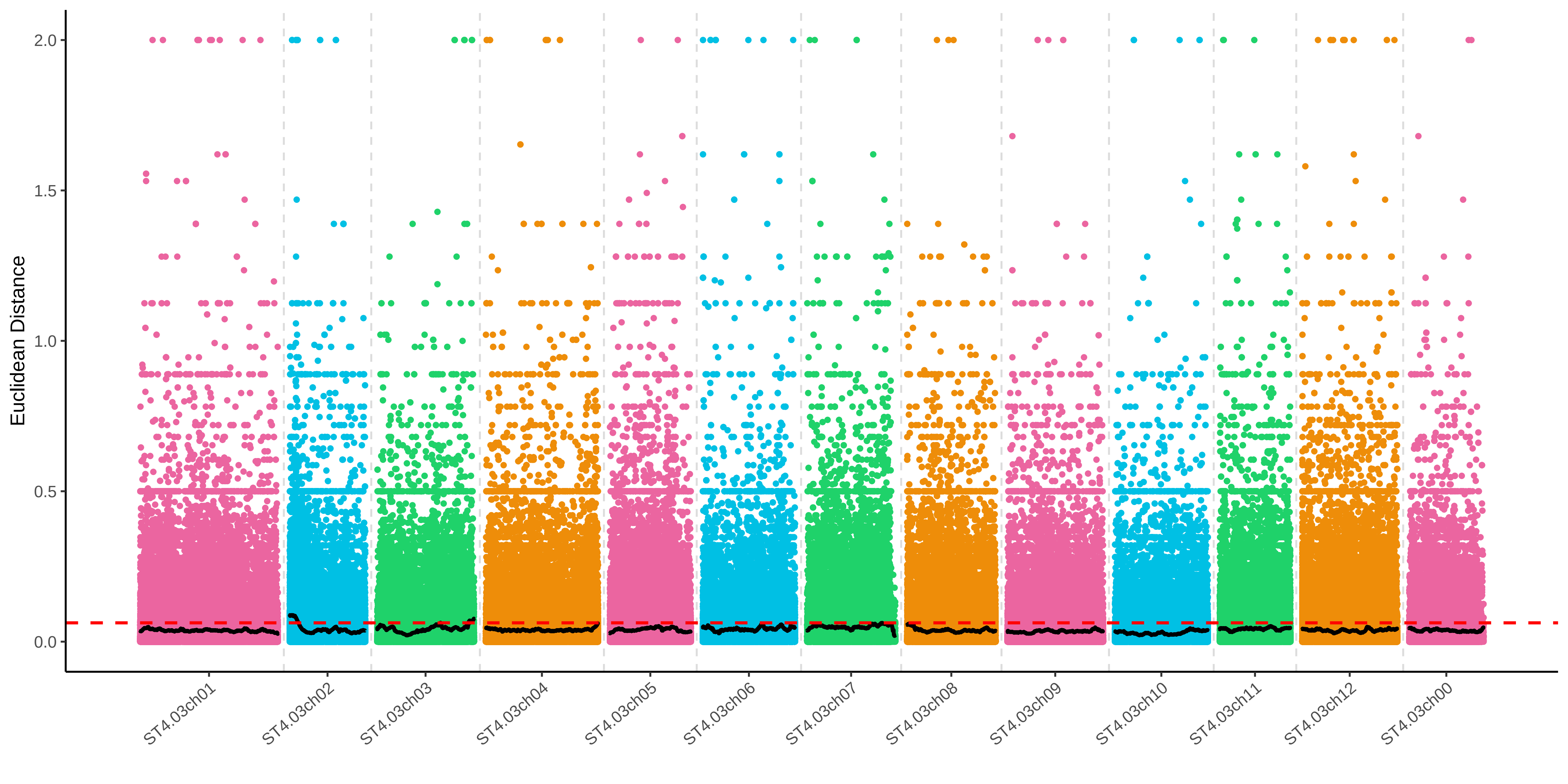

Euclidean Distance
2.0
1.5
1.0
0.5
0.0
ST4.03ch01
ST4.03ch02
ST4.03ch03
ST4.03ch04
ST4.03ch05
ST4.03ch06
ST4.03ch07
ST4.03ch08
ST4.03ch09
ST4.03ch10
ST4.03ch11
ST4.03ch12
ST4.03ch00
